# A Paramagnetic NMR Spectroscopy Toolbox for the Characterisation of Paramagnetic/Spin‐Crossover Coordination Complexes and Metal–Organic Cages

**DOI:** 10.1002/anie.202008439

**Published:** 2020-08-26

**Authors:** Marc Lehr, Tobias Paschelke, Eicke Trumpf, Anna‐Marlene Vogt, Christian Näther, Frank D. Sönnichsen, Anna J. McConnell

**Affiliations:** ^1^ Otto Diels Institute of Organic Chemistry Christian-Albrechts-Universität zu Kiel Otto-Hahn-Platz 4 Kiel 24098 Germany; ^2^ Institute of Inorganic Chemistry Christian-Albrechts-Universität zu Kiel Max-Eyth-Straße 2 Kiel 24118 Germany

**Keywords:** metal–organic cage, NMR spectroscopy, paramagnetic complex, spin-crossover, supramolecular chemistry

## Abstract

The large paramagnetic shifts and short relaxation times resulting from the presence of a paramagnetic centre complicate NMR data acquisition and interpretation in solution. As a result, NMR analysis of paramagnetic complexes is limited in comparison to diamagnetic compounds and often relies on theoretical models. We report a toolbox of 1D (^1^H, proton‐coupled ^13^C, selective ^1^H‐decoupling ^13^C, steady‐state NOE) and 2D (COSY, NOESY, HMQC) paramagnetic NMR methods that enables unprecedented structural characterisation and in some cases, provides more structural information than would be observable for a diamagnetic analogue. We demonstrate the toolbox's broad versatility for fields from coordination chemistry and spin‐crossover complexes to supramolecular chemistry through the characterisation of Co^II^ and high‐spin Fe^II^ mononuclear complexes as well as a Co_4_L_6_ cage.

## Graphical abstract

The paramagnetic NMR toolbox of 1D and 2D NMR techniques now enables in depth structural analysis of paramagnetic mononuclear complexes and metal–organic cages comparable to that of diamagnetic analogues. The robustness and broad versatility of the toolbox is demonstrated by the characterisation of Co^II^ and high spin Fe^II^ mononuclear complexes and a Co^II^_4_L_6_ cage.

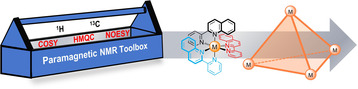

## Introduction

NMR spectroscopy is indispensable for the solution structural characterisation of macromolecules from proteins^1^ to supramolecular architectures,^2^ including interlocked structures,^3^ metal‐organic cages^4^ and topologically complex molecules.^5^ With the standard suite of 1D and 2D NMR methods, structural assignment of diamagnetic compounds and complexes is straightforward but NMR spectroscopy in the presence of paramagnetic centres is more difficult.

Paramagnetic NMR spectroscopy^6^ is central to many fields from chemical and structural biology for studying the structure, dynamics and interactions of proteins1b, 1c, 7 to probing spin‐state populations in spin‐crossover compounds^8^ and the structural characterisation of paramagnetic complexes^9^ and supramolecular architectures.4b, 10 However, NMR data acquisition and interpretation in the presence of a paramagnetic centre presents a number of challenges due to the large paramagnetic shifts, short relaxation times and broad linewidths: pulse programs with long or multiple pulses are not suitable since relaxation can occur before data acquisition takes place;6b uniform excitation is more difficult over the larger spectral range; some signals may be lost completely in the case of very short relaxation times and broad linewidths;6e structural information usually extracted from the chemical shift and *J*‐coupling is lost.6e, 9a, 10a Furthermore, there are limitations to current methods for spectral assignment; they often rely on the availability of accurate theoretical models6b, 9b, 11 or single crystal X‐ray structures for correlation of *T*_1_ relaxation times to metal‐proton distances using the Solomon equation.4b, 10b, 10d As a result, solution characterisation is, in many cases, limited to a ^1^H NMR spectrum only where complete and unambiguous assignment may not be possible.

Nevertheless, paramagnetic NMR spectroscopy also has advantages compared to diamagnetic NMR spectroscopy: the large paramagnetic shifts result in reduced likelihood of signal overlap from dispersion of the NMR signals over a wider chemical shift range;10c the fast relaxation times in comparison to diamagnetic compounds enables reduction of the acquisition times and recycle delays, thereby significantly reducing the demand on instrument time.4b, 6b, 12 Alternatively, this can be exploited for a greater sensitivity of detection through extensive scan averaging. In supramolecular chemistry this has allowed detection of guest binding within paramagnetic cages,4b, 10c even when the guest is present as a trace impurity.4b

Despite these advantages, the full potential of paramagnetic NMR spectroscopy is still to be realised; in comparison to the wealth of 1D and 2D NMR methods for diamagnetic compounds, the number of methods suitable for paramagnetic complexes is limited by fast relaxation although paramagnetic DOSY has been recently reported.6e, 13 Furthermore, the data acquisition/interpretation difficulties6c still need to be overcome to enable straightforward structural characterisation by paramagnetic NMR spectroscopy.

**Figure 1:**
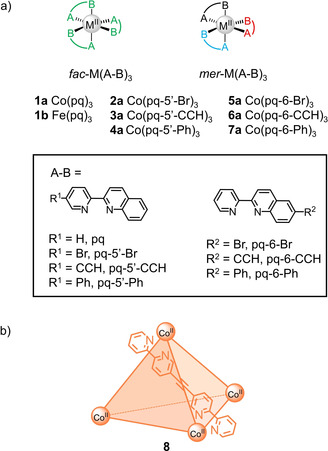
Paramagnetic a) mononuclear Co^II^ and Fe^II^ complexes based on sterically bulky 2‐pyridylquinoline (pq) motifs and b) Co_4_L_6_ cage for characterisation by the toolbox of paramagnetic NMR techniques.

We report a toolbox of 1D (^1^H, proton‐coupled ^13^C, selective ^1^H‐decoupling ^13^C, steady‐state NOE) and 2D (COSY, NOESY, HMQC) paramagnetic NMR methods that have proven particularly robust towards fast relaxation and enable unprecedented in‐depth structural analysis of paramagnetic complexes in solution. We demonstrate the general applicability of this selection of robust experiments by characterising paramagnetic complexes from various fields of chemistry: Co^II^ mononuclear complexes **1 a**–**7 a** (Figure 1 a) as representative examples of paramagnetic coordination complexes; Fe^II^ mononuclear complex **1 b** (Figure 1 a), whose perchlorate and tetrafluoroborate salts are known to undergo spin‐crossover in the solid state,^14^ to represent the high‐spin state of a spin‐crossover complex; and metal‐organic cage **8** (Figure 1 b) as an example of a paramagnetic supramolecular architecture.

## Results and Discussion

We initially investigated the mononuclear complexes to optimise the toolbox and test its limits for spectral assignment. Since the mononuclear complexes could form a mixture of meridional (*mer*) and facial (*fac*) isomers, up to four sets of NMR signals could, in principle, be observed based on symmetry considerations: 1 set for the *fac* isomer (represented as green ligands in Figure 1 a) and 3 sets for the ligand in the three different environments in the *mer* isomer (represented as black, red and blue ligands, Figure 1 a). The 2‐pyridylquinoline (pq) coordination motif was chosen to investigate the influence of steric bulk on the *fac/mer* ratio since its coordination chemistry with labile octahedral metal ions has been underexplored in solution.11b

**Figure 2:**
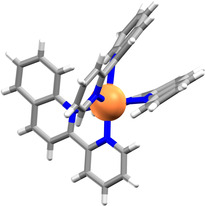
Single crystal X‐ray structure of [Co(pq)_3_](BF_4_)_2_ (**1 a**) with counteranions omitted for clarity.

Co^II^ complex **1 a** with the parent pq^15^ ligand (SI, Section 2.1) was studied first rather than the literature‐known Fe^II^ complex **1 b**,^14^ which could undergo spin‐crossover complicating NMR analysis. Complex **1 a** was prepared either in situ or as crystals by mixing Co(BF_4_)_2_⋅6 H_2_O and three equivalents of the ligand in CD_3_CN or EtOH, respectively. Single crystal X‐ray analysis revealed *mer*‐**1 a** crystallised (Figure 2 and SI, Section 3.1.1.1).^18^ Like the analogous crystal structure of [Fe(pq)_3_](BF_4_)_2_ (**1 b**),14a adoption of the *mer* configuration is attributed to the ligands’ steric bulk and π‐π stacking interactions are observed between two of the ligands (black and red ligand environments in Figure 1 a) causing distortion of the N‐Co^II^‐N angles from the ideal 90° octahedral geometry (Table S3).

In solution, the ^1^H NMR spectra of the complex prepared in situ and by redissolving the crystals in CD_3_CN were similar (Figure S32). The ^1^H signals were spread over a 250 ppm range with 24 relatively sharp signals of equal intensity (linewidths up to 70 Hz, Table S5) and 4 broader signals (Figure S34). This suggests that the *mer* isomer is not only the solid‐state structure but also the only structure in solution; the deviation from the expected 30 signals is attributed to either overlapping or broad signals.

**Figure 3:**
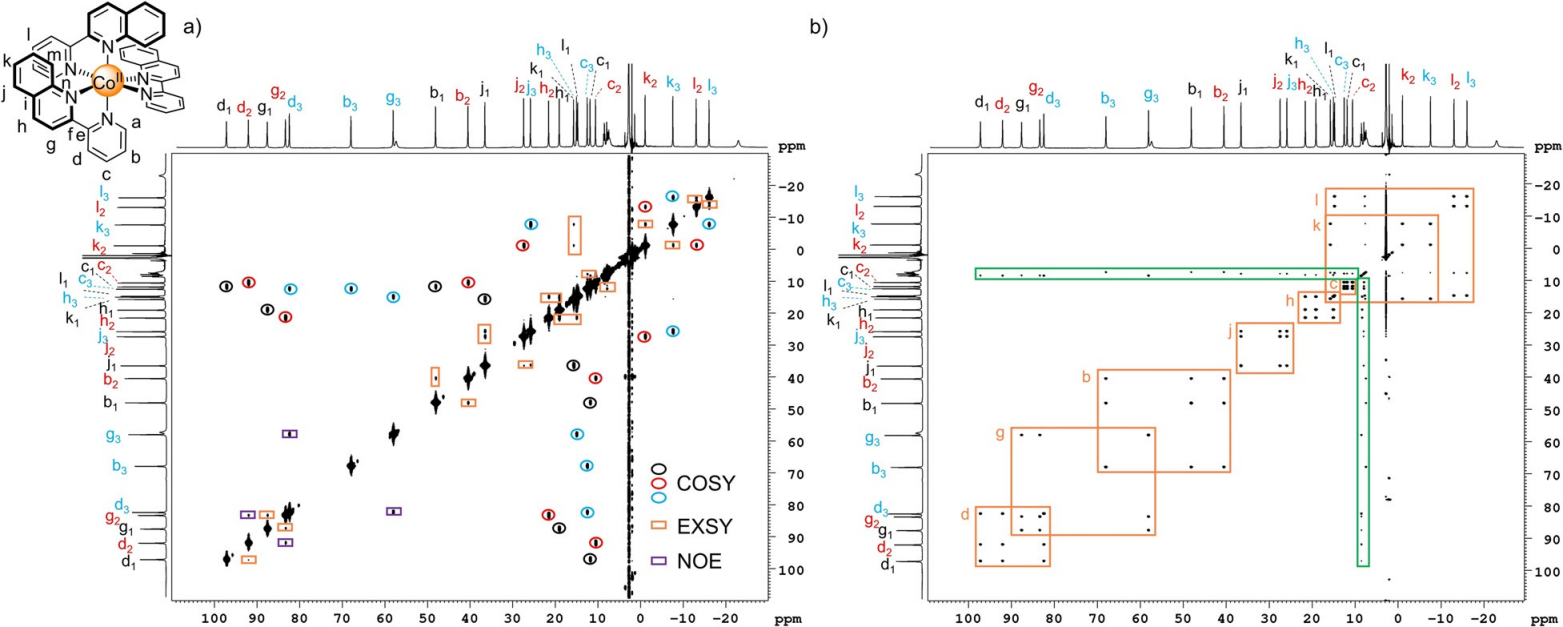
a) ^1^H‐^1^H COSY NMR spectrum (600 MHz, CD_3_CN, 298 K) of *mer*‐[Co(pq)_3_](BF_4_)_2_ (**1 a**) where through‐bond (COSY) cross‐peaks within the three ligand environments are represented by the red, black and blue circles, respectively. Additional structural information in the form of exchange (EXSY) cross‐peaks (orange boxes) and through‐space (NOE) cross‐peaks (purple boxes) is present due to chemical exchange and cross‐correlation,^16^ respectively. Note: the numbers 1, 2, 3 on the proton labels represent the three sets of coupled protons within a single spin system (i.e. protons *b‐d*). The absence of NOE cross‐peaks between protons *h* and *j* prevented assignment of these spin systems to a particular ligand environment and therefore, spin system *j‐l* was arbitrarily labelled with black, red and blue labels in decreasing chemical shift order of proton *j* to represent the three ligand environments. b) ^1^H‐^1^H NOESY NMR spectrum (600 MHz, CD_3_CN, 298 K) of *mer*‐[Co(pq)_3_](BF_4_)_2_ (**1 a**). Exchange (EXSY) cross‐peaks are represented by the orange boxes and exchange cross‐peaks between the complex and excess ligand are shown by the green boxes.

Assignment of the ^1^H spectrum was initially attempted using the Solomon equation, which has been successfully applied to assign the spectra of highly symmetric cages by correlating *T*_1_ relaxation times to the metal‐proton distances from the single crystal X‐ray structure.4b, 10b, 10d For complex **1 a** the *T*_1_ relaxation times varied from 0.7 to 80 ms (Table S4) but a limitation of this currently available assignment method was highlighted during analysis; only partial assignment was possible since not only protons *d* and *g* (Figure 3 a) but also, and more importantly, the three different ligand environments cannot be distinguished on the basis of *T*_1_ relaxation times/Co^II^‐proton distances alone. Ward also encountered this limitation in the assignment of a lower symmetry metal‐organic cage.10c Therefore, we sought to remove the reliance of assignment on the Solomon equation by optimising a toolbox of paramagnetic NMR experiments with broad applicability for the straightforward characterisation of a variety of paramagnetic complexes. The following description of the structural assignment of complex **1 a** and related mononuclear complexes is used to illustrate how the paramagnetic NMR toolbox was optimised to overcome commonly encountered data acquisition and interpretation difficulties. An instruction manual for application of the toolbox to the characterisation of other paramagnetic complexes and cages is provided in Section 1.1.1 of the SI.

We initially turned our attention to COSY since this would allow grouping of the resonances within the different ligand environments. Encouraged by the observation of cross‐peaks in the COSY spectra of several paramagnetic complexes9a and supramolecular architectures4b, 10a, 10b despite the broad linewidths and short relaxation times, we tested and optimised several COSY variants (SI, Section 1.1.1.2). Reduction of the recycle delays and acquisition times due to the fast relaxation times enabled the acquisition of more data in a shorter amount of time using these optimised paramagnetic COSY parameters (typically 5.5 mins for 4 scans, Table S1) compared to the standard COSY parameters (8 min for 1 scan, Table S1).

Cross‐peaks were observed in spectra recorded using the pulse programs cosygpqf (Figure 3 a), cosyqf90 and cosygpmfqf (Figure S35), although intense diagonal peaks6b and commonly observed artefacts, such as *T*_1_ noise streaks and anti‐diagonal peaks, were present to varying degrees dependent on the pulse program. The availability of three suitable paramagnetic COSY pulse programs will enable broad applicability to a variety of paramagnetic complexes and facilitate the implementation of paramagnetic COSY as a standard characterisation method.

The three COSY spectra of complex **1 a** display a large number of cross‐peaks (Figure 3 a, Figure S35). Notably, COSY^19^ cross‐peaks expected on the basis of ^3^ *J* coupling were observable facilitating identification of neighbouring protons and thus, grouping of the proton resonances to one of the three ligand environments (black, red and blue circles, Figure 3 a). Unexpectedly, additional cross‐peaks were observed (orange and purple boxes in Figure 3 a, Figure S35), although the COSY spectrum of [Co(bpy)_3_](BF_4_)_2_ as a reference complex showed only the two expected cross‐peaks arising from ^3^ *J* coupling (Figure S96). The origin of these additional cross‐peaks was investigated using NOESY since Wimperis and Bodenhausen16c, 16d as well as Bertini16a, 16b have reported the presence of additional relaxation‐allowed cross‐peaks in the COSY spectra of paramagnetic complexes that result not from through‐bond coupling but rather cross‐correlation from through‐space (NOE) coupling between the nuclei as well as between the nuclei and the paramagnetic centre.

The standard NOESY pulse program was modified for application to paramagnetic complexes (SI, Section 1.1.1.3). For an initial experiment, a mixing time of 20 ms was chosen as a compromise between the short relaxation times and comparatively long NOE cross‐relaxation rates. Pleasingly, cross‐peaks for the sharp signals (linewidth <70 Hz) were observed (Figure 3 b). Given the range of *T*_1_ relaxation times within the complex, optimisation of the mixing time was investigated. A series of NOESY spectra were measured varying the mixing time from 1 ms to 20 ms and the cross‐peaks were integrated (Figure S36). For protons with *T*_1_ relaxation times significantly longer than the mixing time, the exchange integral approached a maximum as the mixing time increased. However, for protons with shorter *T*_1_ relaxation times (e.g. protons *d* and *g*), the exchange integral reached a maximum before decreasing as relaxation began competing with exchange when the mixing time increased. A mixing time of 10 ms was found to be a good compromise for maximising the exchange cross‐peak of all protons despite their differing *T*_1_ relaxation times.

Analysis of the NOESY spectrum revealed groups of three cross‐peaks (orange boxes, Figure 3 b), corresponding to chemical exchange between the three different ligand environments of the *mer* isomer. Thus, the spectrum has no NOE cross‐peaks but is an EXSY spectrum since the mixing time was so short. Furthermore, EXSY cross‐peak intensities can be close to 100 %,6c whereas NOE intensities are, in general, small for small molecules and reduced even further by the fast relaxation from coupling to the paramagnetic center.6d Since some excess ligand was also present in the complex solution, exchange cross‐peaks were also observed between the excess ligand and ligand in the complex (green boxes, Figure 3 b), enabling assignment of protons *b‐l* in the complex using the free ligand assignments (Table S6) despite broadening and small shifts between free and excess ligand signals due to the presence of Co^II^.

The COSY spectrum (Figure 3 a) was then reanalysed with the proton assignments to determine the origin of the additional cross‐peaks beyond the expected COSY cross‐peaks (black, red and blue circles). These additional cross‐peaks correspond to structural information that is not typically observable in the COSY spectra of diamagnetic compounds; relaxation‐allowed through‐space (NOE) cross‐peaks between protons *d* and *g* (purple squares) were observed due to cross‐correlation^16^ and EXSY cross‐peaks (orange boxes) were observed due to exchange between the three ligand environments, as confirmed by the exchange cross‐peaks in the NOESY spectrum (Figure 3 b).

Thus, almost complete assignment of the ^1^H spectrum of **1 a** was possible using COSY and NOESY with the exceptions of the assignment of: i) spin systems *g‐h* and *j‐l* to a particular ligand environment since relaxation‐allowed (NOE) cross‐peaks were not observed between protons *h* and *j* in the COSY spectrum; ii) the broad signals, which are proposed to be protons *a* and *m* due to their close proximity to the paramagnetic Co^II^ centre. TOCSY (Figure S37) and steady‐state NOE experiments (Figures S38, S39) were carried out but exchange cross‐peaks rather than long‐range coupling and NOE cross‐peaks, respectively, dominated these experiments. Therefore, the unambiguous assignment of spin‐system containing protons *j‐l* to a particular ligand environment was not possible and the spin system was arbitrarily labelled with black, red and blue labels according to decreasing chemical shift of proton *j* to represent the three different ligand environments. Steady‐state NOE experiments, however, did allow assignment of the broad signals above and below −22 ppm as protons *a* and *m*, respectively (Figure S40). The complete proton assignment of complex **1 a** was independently corroborated by *T*_1_ relaxation measurements (Table S4) as well as exchange cross‐peaks between the excess ligand present in the sample and the complex.

Having successfully assigned the ^1^H NMR spectrum of **1 a**, we investigated assignment of the ^13^C NMR spectrum using paramagnetic analogues of heteronuclear 1D and 2D techniques (e.g. HSQC and HMBC). The proton‐coupled ^13^C spectrum contained signals over almost a 900 ppm range (Figure S41), making uniform excitation over this very wide spectral range difficult. Therefore, overlapping spectra of smaller spectral widths were acquired to cover the entire range. The quaternary carbons could be distinguished from the tertiary carbons on the basis of the multiplicity and initially, the tertiary carbons were assigned using selective ^1^H‐decoupling ^13^C experiments where one after another, each proton signal was selectively irradiated during repeated acquisitions of the ^13^C NMR data (Figures S42–S46). However, this method is time‐consuming due to the number of signals and the sensitivity of ^13^C NMR measurements and therefore, we instead chose to investigate 2D heteronuclear experiments.

**Figure 4:**
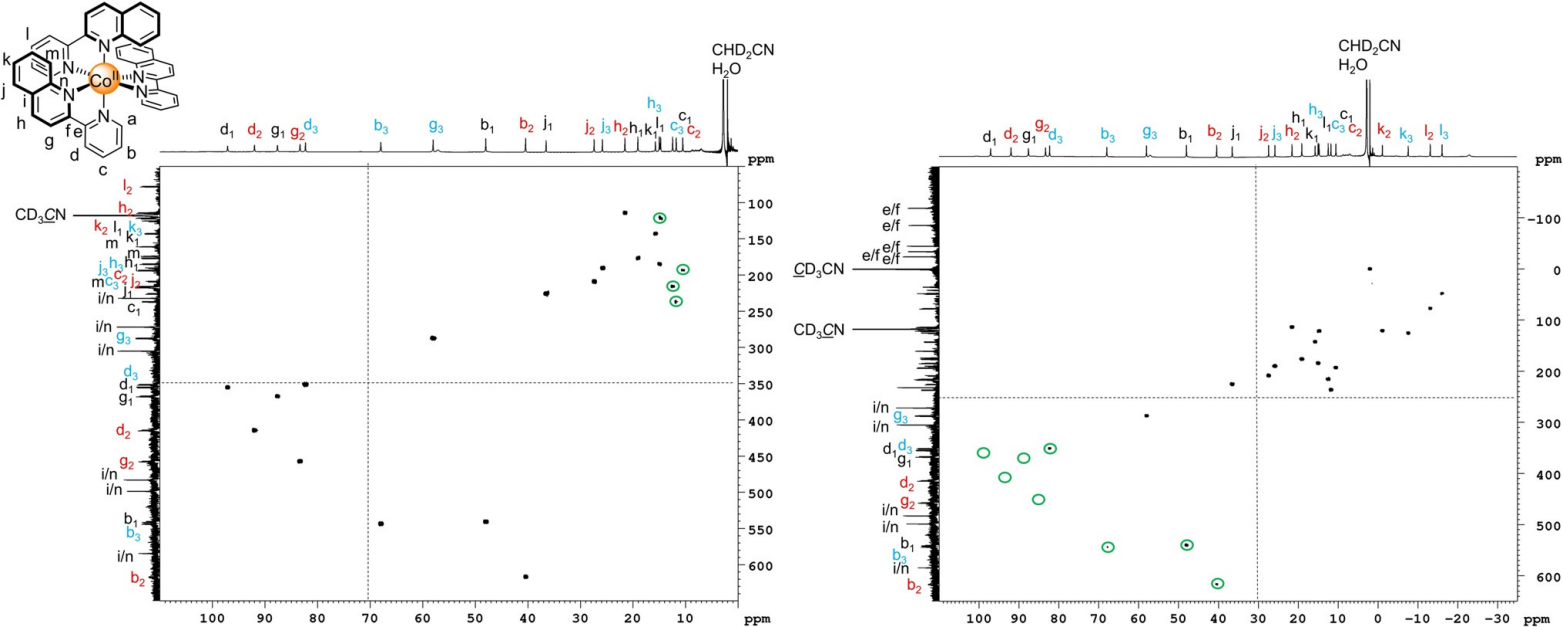
^1^H‐^13^C HMQC NMR spectra (600 MHz/151 MHz, CD_3_CN, 298 K) of *mer*‐[Co(pq)_3_](BF_4_)_2_ (**1 a**). Two spectra with differing offsets (represented by the dashed lines) were recorded since uniform excitation could not be achieved with standard square pulses over the large spectral range resulting in absent cross‐peaks or cross‐peaks with reduced intensities (green circles) at the extremes of the spectral range.

We focused on the HMQC pulse program as an alternative to HSQC because of its simple four pulse sequence and pleasingly, cross‐peaks were observed for the sharp ^1^H signals (linewidth <70 Hz, Figures S47–S49). However, the acquisition of two HMQC spectra was necessary to cover the large spectral range in both dimensions as non‐uniform excitation resulted in a decrease in the intensity or complete loss of cross‐peaks at the extremes of the spectral range (Figure 4). Nevertheless, an overlay of the two HMQC spectra confirmed the assignments made using the selective ^1^H‐decoupling ^13^C experiments (Figures S42–S46). HMBC spectra were also acquired in an attempt to assign the quaternary carbons and the three spin systems within a particular ligand environment; however, no cross‐peaks were observed due to the long delays resulting from the small magnitude of ^3^ *J* coupling constants in contrast to the large ^1^ *J* coupling constants utilised in the HMQC experiments. Therefore, the quaternary carbons and carbon *a* were tentatively assigned through comparison to the reference complex [Co(bpy)_3_](BF_4_)_2_ (Figures S97–S99).

Thus, we successfully assigned the ^1^H and ^13^C NMR spectra of complex *mer*‐**1 a** using a combination of 1D (^1^H, proton‐coupled ^13^C, selective ^1^H‐decoupling ^13^C, steady‐state NOE) and 2D (COSY, NOESY, HMQC) NMR paramagnetic experiments. We then sought to test the limits of this paramagnetic NMR toolbox in complexes where proton coupling within a spin system is interrupted by substitution as well as complexes with broader linewidths. We prepared a series of 2‐pyridylquinoline ligands substituted in the 5′‐ or 6‐positions (Figure 1 a) and their corresponding Co^II^ complexes **2 a**–**7 a** in situ by mixing Co(BF_4_)_2_⋅6 H_2_O and three equivalents of the ligand in CD_3_CN.

These complexes fulfil both criteria, as revealed by their NMR spectra (Figures S93–S94, Table S5), and they also allowed investigation of the influence of substitution on the *fac/mer* isomerism. Based on comparison to the ^1^H NMR spectrum of the parent complex **1 a**, it appeared that the *mer* isomer also predominated for complexes **3 a**–**7 a** but additional species were also present (Figures S93–S94). In the case of the complex with pq‐5′‐Br, a set of broader signals consistent with only one ligand environment was also a significant species within the mixture (Figure S51).

Initially, assignment of the proposed *mer* isomer in the ^1^H and ^13^C NMR spectra of complexes **2 a**–**7 a** was investigated. In comparison to the parent complex **1 a**, the ^1^H signals of complexes **2 a**–**4 a** with 5′‐substituted ligands had the largest linewidths (>90 Hz, Table S5) followed by complexes **5 a**–**7 a** containing 6‐substituted ligands (<130 Hz, Table S5). Nevertheless, cross‐peaks were still observable in the 2D spectra (COSY, NOESY and HMQC) including exchange cross‐peaks in the COSY spectra to varying degrees for complexes **3 a**–**7 a** (Figures S59, S66, S72, S79, S87). The ^1^H assignments for these complexes confirmed that in each case the major species in solution is the *mer* isomer (Figures S93–S94). ^1^H assignment of *mer*‐**2 a** was also possible despite the absence of many cross‐peaks in the COSY spectrum since exchange cross‐peaks were still observable in the NOESY spectrum. We attribute the incompleteness of cross‐peaks in the COSY spectrum to the presence of broad linewidths (>200 Hz, Table S5). Assignment of the ^13^C NMR spectra of complexes **2 a**–**7 a** was not as straightforward as the ^1^H spectra, most likely due to the lower sensitivity of ^13^C NMR spectroscopy compared to ^1^H NMR spectroscopy, the broadness of the signals and the presence of multiple species. Thus, in some cases only partial assignment was possible (Figures S62–S63, S68–S69, S74–S75, S81–S83, S89–S91).

A comparison of the ^1^H assignments for *mer*‐**2 a**–**7 a** to those of the parent complex *mer*‐**1 a** showed that the signals of the 2‐pyridylquinoline backbone do not shift significantly upon substitution and as expected, the ^1^H signals for the 5′‐ and 6‐positions are not present in the spectra of complexes **2 a**–**4 a** and **5 a**–**7 a**, respectively, due to substitution (Figures S93–S94). The protons in these spin systems with substituents could be assigned by the exchange peaks in the NOESY spectra but not to a particular ligand environment, due to the disruption of proton coupling by substitution in the COSY spectrum and absence of suitable TOCSY and HMBC pulse programs for paramagnetic complexes.

We then investigated the assignment of the species other than the *mer* isomer in the spectra of **2 a**–**7 a**. A set of sharp signals consistent with only one ligand environment was visible in the spectra of complexes **3 a** and **5 a** (Figures S93–S94), and another set of broader signals, also consistent with only one ligand environment, was visible for all complexes with the exception of **6 a** (Figures S93–S94). We propose these two species to be the *fac* isomer and a symmetric CoL_2_‐based species. While these species cannot be distinguished on the basis of the number of NMR signals, we attribute the set of broader signals to a symmetric CoL_2_‐based species; this set of broader signals was significant for the complex with pq‐5′‐Br yet decreased in intensity upon addition of a fourth equivalent of ligand while the *mer*‐**2 a** signals increased (Figure S51). Furthermore, in two samples of the complex with pq‐6‐Ph, the chemical shifts of the proposed symmetric CoL_2_‐based species were sensitive to the differing water content of the samples whereas those corresponding to *mer*‐**7 a** were not (Figure S86). We attribute the observation of a symmetric CoL_2_‐based species as well as *mer*‐**2 a** to the steric bulk of pq‐5′‐Br from not only the quinoline ring but also the bromine substituent since this could reduce the efficiency of π‐π stacking interactions between two of the ligands. In contrast, the predominance of *mer*‐**5 a** based on the pq‐6‐Br ligand is likely due to the reduced steric influence of the bromine substituent in the 6‐position compared with the 5′‐position in complex **2 a**.

Assignment of these species was more difficult than the *mer* isomer since cross‐peaks for these species were not typically observable in the COSY spectra, most likely due to the broadness of the signals in the case of the CoL_2_‐based species and low concentration in the case of the *fac* isomer (estimated to constitute less than 1 % of the complex mixture). However, NOESY appears to be less sensitive to signal broadness than COSY since exchange cross‐peaks between the CoL_2_‐based species and the *mer* complex were still observed (Figures S54, S60, S67, S73). Furthermore, these cross‐peaks were even visible when the CoL_2_‐based species was not detectable in the ^1^H NMR spectrum due to signal broadness and/or the low concentration of this species as seen in the spectrum of complex **6 a** (Figures S78, S80).

To further investigate the applicability of the paramagnetic NMR toolbox we extended our studies to the characterisation of a high spin/spin‐crossover Fe^II^ complex. Complex **1 b** was prepared in a glovebox by mixing Fe(OTf)_2_ and three equivalents of the pq ligand in dry CD_3_CN. At room temperature the ^1^H NMR spectra of the complex contained broad signals for the complex and therefore, detailed analysis was not possible. We attributed the broad signals to fast ligand exchange processes and therefore, variable temperature experiments were carried at lower temperatures where ligand exchange would be slower.

Upon cooling the solutions from 298 K to 248 K, the signals sharpened and displayed Curie–Weiss behaviour (Figure S106–S109).^17^ There was no evidence of spin‐crossover over this temperature range, consistent with previous studies on the tetrafluoroborate salt of complex **1 b** in acetone.14b At 248 K almost complete assignment of the ^1^H and ^13^C NMR spectra was possible for *mer*‐**1 b** since the signals were relatively sharp and cross‐peaks were observable in the COSY, NOESY and HMQC spectra (Figures S101–S105). However, the carbon signals for *d* and *g* could not be assigned on the basis of the HMQC spectrum since the cross‐peaks were absent or very weak, attributed to the increased influence of the paramagnetic Fe^II^ ion on the relaxation times at lower temperature. In addition, at least one other species was present at equilibrium as broader signals were also seen in the ^1^H NMR spectrum but they could not be assigned in the absence of cross‐peaks in the 2D NMR spectra.

Given the large change in the linewidth of the ^1^H NMR signals between 248 K and 298 K (Table S7), we carried out temperature‐dependent COSY, NOESY and HMQC experiments to investigate whether cross‐peaks were also observable at higher temperatures (Figures S110–112). HMQC and COSY appeared to be more sensitive to temperature than NOESY since by 266 K most cross‐peaks were no longer observable (Figures S110, S112). Nevertheless, assignment of the ^1^H signals was still possible exploiting the exchange cross‐peaks in the NOESY spectra (Figure S111) and the assignments from lower temperatures to assign coupled protons within a single spin system when COSY cross‐peaks were not observable at that temperature (Figures S110).

Finally, we applied the paramagnetic NMR toolbox to the characterisation of paramagnetic metal‐organic cages. The rational design of lower symmetry metal‐organic cages is challenging and therefore, we prepared and characterised instead the highly symmetric tetrahedral Co^II^_4_L_6_ cage **8** as proof‐of‐principle for paramagnetic cage characterisation (Figures S113–S117). A solution of four equivalents of Co(NTf_2_)_2_ and six equivalents of ligand was heated at 50 °C in acetonitrile and the cage was isolated by precipitation with diethyl ether.

The ^1^H NMR spectrum of the redissolved cage in CD_3_CN contained 5 sharp and 2 broad signals, reflecting the presence of one ligand environment due to *fac* coordination around the metal centres. Only the expected through‐bond cross‐peaks were observed in the COSY spectrum (Figure S114). Full and unambiguous assignment of the ^1^H and ^13^C NMR spectra was possible using the paramagnetic NMR toolbox, with the exception of the quaternary carbons and the broad signals corresponding to *a* and *j* (Figures S113, S115).

**Figure 5:**
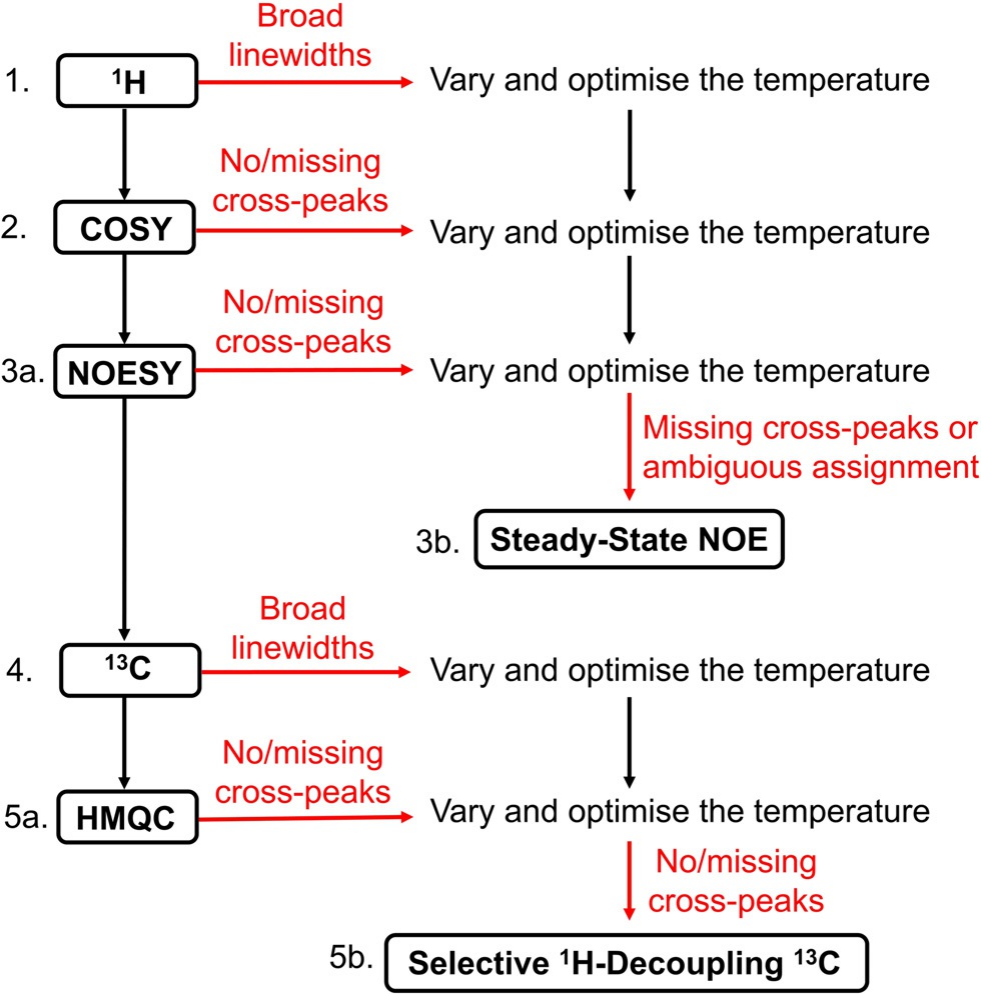
Proposed workflow including troubleshooting experiments (red arrows) for the application of the paramagnetic NMR toolbox to the structural characterisation of paramagnetic complexes and cages.

Following the characterisation of mononuclear complexes **1 a**–**7 a**, **1 b** and cage **8**, we propose a workflow including troubleshooting experiments for the application of the paramagnetic NMR toolbox to the structural characterisation of other paramagnetic complexes and cages (Figure 5). A more detailed instruction manual for each toolbox experiment is provided in the SI (Section 1.1.1). The workflow begins with the acquisition of a ^1^H NMR spectrum to establish the spectral width as well as the linewidths of the signals since large spectral widths often necessitate the acquisition of several 2D spectra with smaller spectral widths and the observation of cross‐peaks in 2D spectra is, in many cases, dependent on the linewidth. With broad linewidths (typically >100 Hz), variation and optimisation of the temperature is recommended in an attempt to reduce the linewidths and increase the likelihood of cross‐peak observation in 2D experiments.

COSY is recommended as the second toolbox experiment to identify coupled protons within each spin system. However, additional relaxation‐allowed exchange and NOE cross‐peaks may be observable complicating assignment and therefore, either NOESY (toolbox experiment 3a) or steady‐state NOE (toolbox experiment 3b) experiments are recommended to complete the ^1^H NMR spectrum assignment. These two experiments provide similar structural information but NOESY has the advantage that the exchange and NOE cross‐peaks can be observed in a single 2D spectrum compared with multiple 1D spectra for steady‐state NOE experiments. For this reason, steady‐state NOE experiments may only be necessary in the case of troubleshooting (Sections 1.1.1.3 and 1.1.1.4).

The assignment of the ^13^C NMR spectrum has a similar workflow beginning with acquisition of the ^13^C NMR spectrum (toolbox experiment 4) followed by HMQC (toolbox experiment 5a) and/or selective ^1^H‐decoupling ^13^C experiments (toolbox experiment 5b) to identify the ^1^ *J*_CH_ coupling. Again, the 2D HMQC experiment is preferable to a series of selective ^1^H‐decoupling ^13^C experiments but these 1D experiments may be useful in troubleshooting (Sections 1.1.1.6 and 1.1.1.7).

## Conclusion

We report a toolbox of 1D (^1^H, proton‐coupled ^13^C, selective ^1^H‐decoupling ^13^C, steady‐state NOE) and 2D (COSY, NOESY, HMQC) paramagnetic NMR methods that enables the straightforward characterisation of paramagnetic complexes. This toolbox overcomes the data acquisition challenges due to the presence of paramagnetic centres, such as large paramagnetic shifts and short relaxation times, and also removes the reliance of data interpretation on theoretical models6b, 9b, 11 or the Solomon equation.4b, 10b, 10d We demonstrated the general applicability of this toolbox for fields from coordination chemistry to spin‐crossover complexes and supramolecular chemistry through the characterisation of Co^II^ and high‐spin Fe^II^ mononuclear complexes as well as a Co_4_L_6_ cage. Furthermore, we demonstrated the toolbox can be successfully applied to structural characterisation in a variety of situations: the assignment of complexes with multiple ligand environments (e.g. *mer* complexes), complexes with a range of signal linewidths (including broad signals in the case of the CoL_2_‐based species) and mixtures of complexes (e.g. *mer*‐ and *fac*‐CoL_3_ isomers as well as CoL_2_‐based species).

This study also shows the advantages of paramagnetic versus diamagnetic NMR spectroscopy; the short relaxation times of paramagnetic complexes enable reduction of the repetition delays and acquisition times, thereby significantly reducing the experiment times or enabling the acquisition of more data in a similar time (Table S1). In addition to reduced signal overlap and increased sensitivity, structural information can be observed by paramagnetic NMR spectroscopy that would not be observable in the diamagnetic analogue; in the COSY spectra of the Co^II^ *mer* mononuclear complexes, relaxation‐allowed through‐space (NOE) cross‐correlation peaks and exchange (EXSY) cross‐peaks were observed in addition to the expected through‐bond (COSY) cross‐peaks. Furthermore, the sensitivity of the exchange NOESY technique enabled the identification of additional species present at equilibrium that were not visible in the ^1^H NMR spectra due to broad linewidths and/or their low concentration.

While solution characterisation of paramagnetic complexes and cages was previously typically limited to a ^1^H NMR spectrum only, we demonstrate that in‐depth structural analysis comparable to that for diamagnetic compounds is now possible using the paramagnetic NMR toolbox. We are now extending the use of this toolbox to the characterisation of more complex as well as mixtures of supramolecular architectures.

## Conflict of interest

The authors declare no conflict of interest.

## Supporting information

As a service to our authors and readers, this journal provides supporting information supplied by the authors. Such materials are peer reviewed and may be re‐organized for online delivery, but are not copy‐edited or typeset. Technical support issues arising from supporting information (other than missing files) should be addressed to the authors.

Supplementary
