# Supplementary material for: A Paramagnetic NMR Spectroscopy Toolbox for the Characterisation of Paramagnetic/Spin‐Crossover Coordination Complexes and Metal–Organic Cages
Source: Angew Chem Int Ed Engl. 2020 Aug 26;59(43):19344–51. doi: 10.1002/anie.202008439 (PMC7590057; doi:10.1002/anie.202008439)
Supplement: Supplementary file 1 — Supplementary [file ANIE-59-19344-s001.pdf]

Angewandte Chemie
Eine Zeitschrift der Gesellschaft Deutscher Chemiker

Supporting Information

A Paramagnetic NMR Spectroscopy Toolbox for the Characterisation of Paramagnetic/Spin-Crossover Coordination Complexes and Metal–Organic Cages

*Marc Lehr, Tobias Paschelke, Eicke Trumpf, Anna-Marlene Vogt, Christian Näther, Frank D. Sönnichsen, and Anna J. McConnell**

anie_202008439_sm_miscellaneous_information.pdf

Author Contributions

M.L. Conceptualization: Lead; Formal analysis: Lead; Investigation: Lead; Methodology: Lead; Resources: Lead; Supervision: Supporting; Visualization: Supporting; Writing - Review & Editing: Equal
T.P. Conceptualization: Equal; Formal analysis: Equal; Investigation: Equal; Methodology: Equal; Resources: Equal; Supervision: Supporting; Writing - Review & Editing: Equal
E.T. Formal analysis: Equal; Investigation: Equal; Methodology: Equal; Resources: Equal; Writing - Review & Editing: Supporting
A.V. Investigation: Supporting; Resources: Supporting
C.N. Formal analysis: Supporting; Investigation: Supporting; Resources: Supporting; Writing - Review & Editing: Supporting
F.S. Conceptualization: Equal; Investigation: Lead; Methodology: Lead; Resources: Equal; Writing - Review & Editing: Equal
A.M. Conceptualization: Lead; Formal analysis: Lead; Funding acquisition: Lead; Methodology: Equal; Project administration: Lead; Resources: Equal; Supervision: Lead; Visualization: Lead; Writing - Original Draft: Lead; Writing - Review & Editing: Lead.

Table of Contents

1 Materials and Methods S3
1.1 NMR Spectroscopy S3
1.1.1 Instruction Manual for the Paramagnetic NMR Toolbox S4
1.1.1.1 ^{1}H NMR spectroscopy S4
1.1.1.2 COSY S5
1.1.1.3 NOESY S6
1.1.1.4 Steady-State NOE Experiments S7
1.1.1.5 ^{13}C NMR Spectroscopy S8
1.1.1.6 HMQC S9
1.1.1.7 Selective ^{1}H-Decoupling ^{13}C NMR Experiments S10
1.1.1.8 TOCSY S10
1.2 Mass Spectrometry S10
1.3 X-Ray Crystallography S10
2 Ligand Synthesis S10
2.1 Unsubstituted Ligand S11
2.1.1 2-(2-Pyridyl)quinoline S11
2.2 5'-Substituted Pyridylquinoline Ligand Derivatives S12
2.2.1 1-(5-Bromopyridin-2-yl)ethanone S13
2.2.2 2-(5'-Bromopyridin-2'-yl)quinoline S14
2.2.3 2-(5'-Ethynylpyridin-2'-yl)quinoline S17
2.2.4 2-(5'-Phenylpyridin-2'-yl)quinoline S20
2.3 6-Substituted Pyridylquinoline Ligand Derivatives S23
2.3.1 6-Bromo-2-(pyridin-2'-yl)quinoline S24
2.3.2 6-Ethynyl-2-(pyridin-2'-yl)quinoline S26
2.3.3 6-Phenyl-2-(pyridin-2'-yl)quinoline S29
2.4 Ligand for Cage 8 ..... S32
2.4.1 5-Ethynyl-2,2'-bipyridine S33
3 Mononuclear Complexes S33
3.1 Mononuclear Cobalt Complexes S33
3.1.1 *mer*-$[Co(pq)_3](BF_4)_2$ (**1a**) S33
3.1.1.1 X-ray crystal structure determination of $[Co(pq)_3](BF_4)_2$ (**1a**) S34
3.1.1.2 Proton Assignment through T_1 Measurements S36
3.1.1.3 Characterisation using Paramagnetic NMR Spectroscopy and Mass Spectrometry S38
3.1.2 *mer*-$[Co(pq\text{-}5'\text{-}Br)_3](BF_4)_2$ (**2a**) S51

3.1.2.1 Characterisation using Paramagnetic NMR Spectroscopy and Mass Spectrometry S51

3.1.3 *mer*-[Co(pq-5'-CCH)$_3$](BF$_4$)$_2$ (**3a**) S56

3.1.3.1 Characterisation using Paramagnetic NMR Spectroscopy and Mass Spectrometry S56

3.1.4 *mer*-[Co(pq-5'-Ph)$_3$](BF$_4$)$_2$ (**4a**) S61

3.1.4.1 Characterisation using Paramagnetic NMR Spectroscopy and Mass Spectrometry S61

3.1.5 *mer*-[Co(pq-6-Br)$_3$](BF$_4$)$_2$ (**5a**) S65

3.1.5.1 Characterisation using Paramagnetic NMR Spectroscopy and Mass Spectrometry S66

3.1.6 *mer*-[Co(pq-6-CCH)$_3$](BF$_4$)$_2$ (**6a**) S70

3.1.6.1 Characterisation using Paramagnetic NMR Spectroscopy and Mass Spectrometry S71

3.1.7 *mer*-[Co(pq-6-Ph)$_3$](BF$_4$)$_2$ (**7a**) S75

3.1.7.1 Characterisation using Paramagnetic NMR Spectroscopy and Mass Spectrometry S76

3.1.8 Comparison of Complexes **1a**-**7a** S81

3.1.9 [Co(bpy)$_3$](BF$_4$)$_2$ S83

3.1.9.1 Characterisation using Paramagnetic NMR Spectroscopy and Mass Spectrometry S84

3.2 Mononuclear Iron Complex S87

3.2.1 *mer*-[Fe(pq)$_3$](OTf)$_2$ (**1b**) S87

3.2.1.1 Characterisation at 248 K using Paramagnetic NMR Spectroscopy S87

3.2.1.2 Variable Temperature Studies S91

4 Co$_4$L$_6$ Cage 8 .... S100

5 References S103

1 Materials and Methods

Reagents and solvents were purchased from commercial suppliers and used without further purification, unless otherwise specified. Where anhydrous deuterated acetonitrile is specified, the deuterated acetonitrile was distilled over calcium hydride and stored in a glovebox. Column chromatography was carried out on an Isolera One from Biotage using Biotage SNAP Ultra columns, unless otherwise specified. Centrifugation of mononuclear complexes and cages was carried out using a Grant-Bio LMC-3000 low speed benchtop centrifuge.

1.1 NMR Spectroscopy

NMR spectra were recorded on a Bruker Avance 200, a Bruker AvanceNeo 500, or a Bruker Avance 600 spectrometer, the latter being equipped with a cryogenically cooled triple-resonance probe head. Chemical shifts for ^{1}H, ^{13}C, and ^{19}F spectra are expressed in parts per million (ppm) and coupling constants (*J*) are reported in Hertz (Hz). ^{1}H and ^{13}C spectra of the diamagnetic compounds were referenced to TMS at 0.0 ppm and the chemical shifts of the paramagnetic complexes/cage are reported relative to the resonance of the residual methyl proton and carbon of CD_3CN (δ_H = 1.94 ppm, δ_C = 1.32 ppm). ^{19}F spectra were referenced to C_6F_6 at -164.9 ppm. All measurements were carried out at 298 K unless reported otherwise. The following abbreviations are used to describe signal multiplicity for ^{1}H, ^{13}C and ^{19}F NMR spectra: s: singlet, d: doublet, t: triplet, m: multiplet, b: broad.

The following pulse programs were used for diamagnetic compounds: zg30 (^{1}H), cosygpmfppqf (COSY), hsqcedetgpsp.3 (HSQC), hmbcgplpndqf (HMBC), zgpg30 (^{13}C).

The following standard Bruker pulse programs were used for the paramagnetic complexes: zg30 (^{1}H), zg (^{1}H), zgfhigqn (^{19}F), cosyqf90 (COSY), cosygpqf (COSY), cosyqpmfqf (COSY), hmqcgpqf (HMQC), zg (^{13}C) and used without modifications. The standard phase-sensitive NOESY experiment (noesyph) was modified, delaying data acquisition in the indirect F1-dimension by two (noesyph-lsw) or four data points (noesyph-2lsw) to facilitate spectral widths of up to 140 or >150 ppm, respectively. Optimisation of the parameters for paramagnetic complexes is described in the following sections and typical parameters are given as a starting point for application of the toolbox to other types of paramagnetic complexes and cages.

Table S1 Comparison of typical experiment times for the paramagnetic versus diamagnetic NMR experiments used in this work.

Pulse Program	Paramagnetic[c]		Diamagnetic	
	Time (min)	Scans	Time (min)	Scans
^{1}H	1.25	1024	0.83	8
^{13}C	17.28	8000	9.35	256
COSY	5.5	4	7.85	1
NOESY	8.5	8	170	8
HMQC[a]/HSQC[b]	8.27[a]	16	15.5[b]	2

[a] It should be noted that at least two HMQC spectra, each typically 8.27 min, need to be acquired to cover the entire spectral range for the paramagnetic complexes. [b] The HSQC pulse program was used for diamagnetic compounds. [c] The paramagnetic NMR experiments were typically carried out with concentrations of 20-100 mM for the mononuclear complexes and 3 mM for the cages.

1.1.1 Instruction Manual for the Paramagnetic NMR Toolbox

The proposed workflow for use of the paramagnetic NMR toolbox is illustrated in Figure 5 of the main text. An instruction manual is provided in the following sections for its application to the characterisation of paramagnetic complexes and cages.

The instruction manual for each toolbox experiment consists of a flowchart demonstrating the suggested workflow for optimising NMR data acquisition (blue text) and troubleshooting NMR data acquisition and interpretation (red text). Where relevant, alternative experiments are suggested for troubleshooting and each flowchart ends with next toolbox experiment in the suggested workflow from Figure 5. A more detailed explanation of optimisation and troubleshooting strategies is found in the text accompanying each flowchart. Typical parameters are given for the complexes and cages studied in this work and these can be used as a starting point for application of the toolbox to other types of paramagnetic complexes and cages.

1.1.1.1 ^{1}H NMR spectroscopy

Flowchart 1. Workflow for the optimisation (blue text) and troubleshooting (red text) of paramagnetic ^{1}H NMR experiments (Toolbox experiment 1).

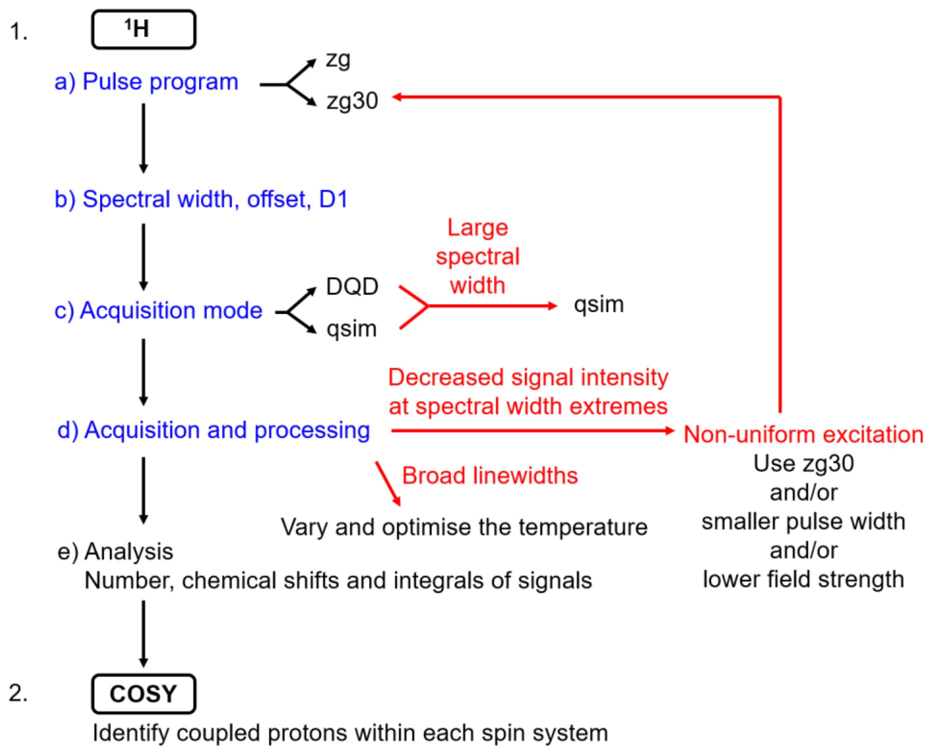

Paramagnetic ^{1}H NMR spectra were recorded using either the zg or the zg30 pulse program in either DQD or qsim acquisition mode, depending on the spectral width. With the qsim acquisition mode an artefact was introduced at the offset frequency but this mode was necessary for larger spectral widths (with the exact values depending on the generation of the spectrometer console). The NMR spectra were recorded with a typical spectral width of approximately 300 ppm centred at -40 ppm, an acquisition time of 0.04-0.07 s and a typical D1 value of 1 ms. The spectra were processed applying a line broadening of 1-15 Hz. Prior to the recording of ^{1}H NMR spectra, the 90° pulse width was determined via zero excitation for a 180° on-resonance pulse of a selected signal. Due to the large spectral width intensities in the 1D 1H NMR spectra suffered from non-uniform excitation; this reduced the

accuracy of signal integration for the spectra recorded using the zg pulse program/90° excitation pulse at 600 MHz. More uniform excitation was achieved using the zg30 pulse program, even smaller pulse widths and/or the use of lower field strengths and the integrals could be used as a guide during signal assignment.

1.1.1.2 COSY

Flowchart 2. Workflow for the optimisation (blue text) and troubleshooting (red text) of paramagnetic COSY NMR experiments (Toolbox experiment 2).

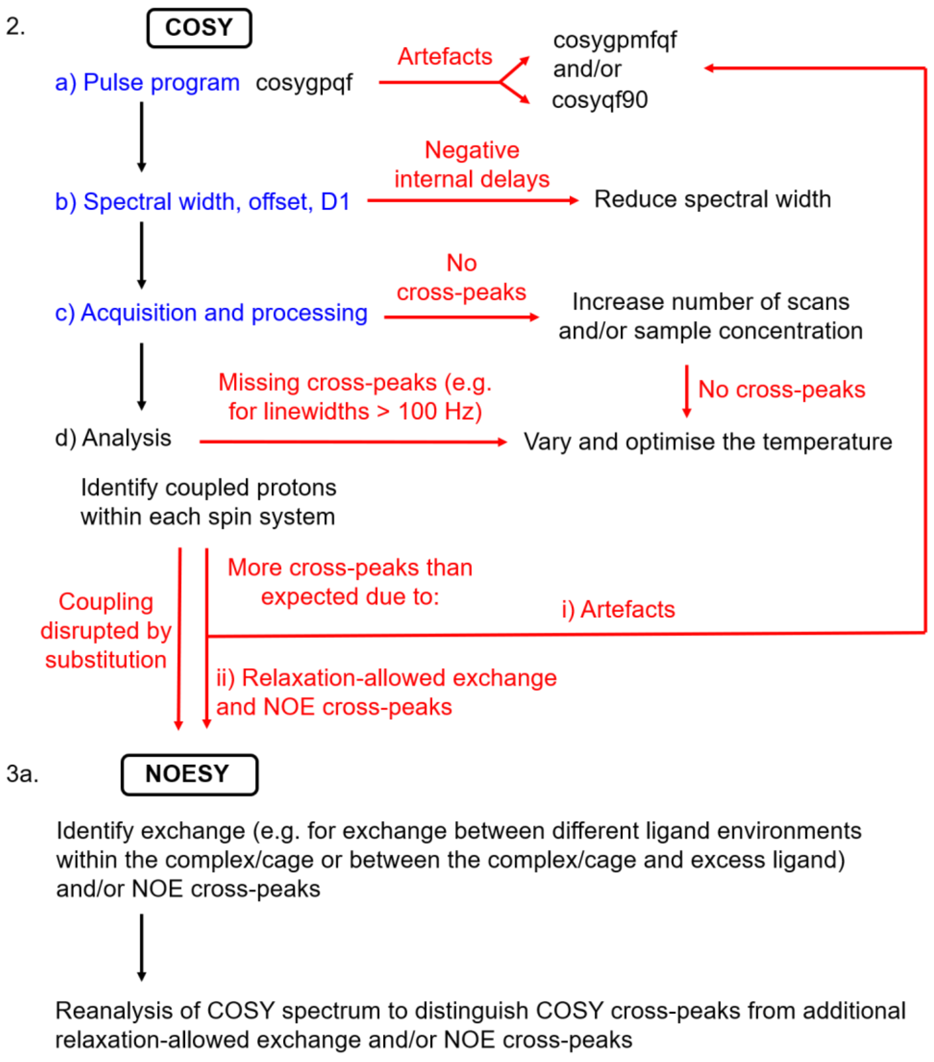

COSY NMR spectra were recorded using either the cosyqf90, cosygpqf or cosyqpmfqf pulse program with a 140-167 ppm spectral width centred at 40-45 ppm using 4 scans, 2048 and 256/512/2048 increments and acquisition times of 0.012 s and 0.003/0.005/0.024 s in the F2 and F1 dimensions, respectively. The COSY spectra were processed using a line broadening of 1 Hz in the F2 dimension and sinebell weighting in both dimensions.

The paramagnetic COSY spectra often contain artefacts to varying degrees and this can complicate analysis. In cases of a spectrum with a significant number of artefacts, the availability of multiple pulse programs in the paramagnetic NMR toolbox has the advantage that other pulse programs can be screened to investigate whether the number of artefacts can be reduced. The non-gradient pulse program cosyqf90 typically contained the most artefacts but allowed the fastest scanning of the three pulse programs. The gradient

sequences cosygpqf and cosyqpmfqf performed better and the cosygpqf pulse program typically gave COSY spectra with the least number of artefacts. However, gradient duty cycle limits prohibited the use of fast scan rates principally facilitated by the very fast relaxation.

1.1.1.3 NOESY

Flowchart 3. Workflow for the optimisation (blue text) and troubleshooting (red text) of paramagnetic NOESY NMR experiments (Toolbox experiment 3a).

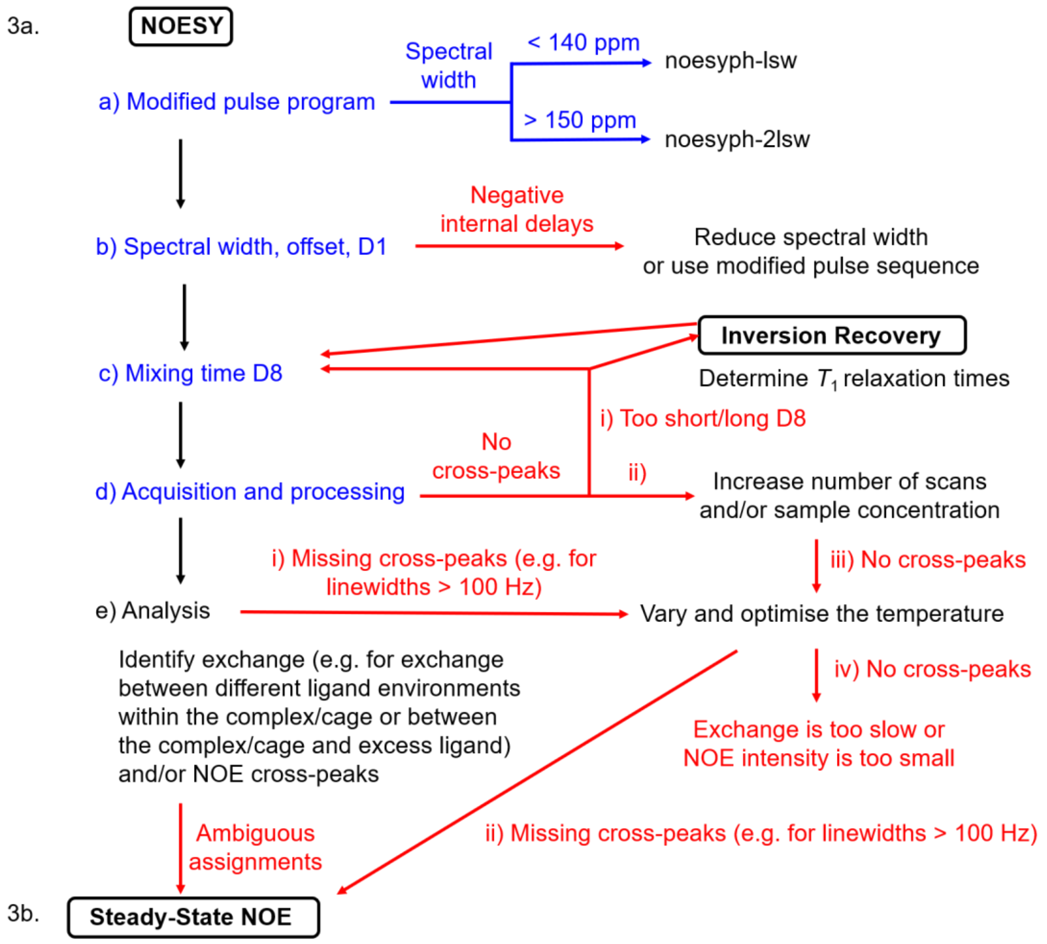

NOESY spectra were recorded using either the modified noesyph-lsw or noesyph-2lsw pulse program with a spectral width of 140/170 and 140 ppm with an offset at 40 ppm, typical acquisition times of 0.005-0.05 and 0.006 s, increments of 1024 and 1024 in the F2 and F1 dimensions, respectively. A repetition delay D1 of 0.04 s and a mixing time D8 of 1-20 ms was typically used with a D8 of 10 ms being a good compromise for maximising the exchange cross-peak for all protons despite their differing T_1 relaxation times.

1.1.1.4 Steady-State NOE Experiments

Flowchart 4. Workflow for the optimisation (blue text) and troubleshooting (red text) of paramagnetic steady-state NOE NMR experiments (Toolbox experiment 3b).

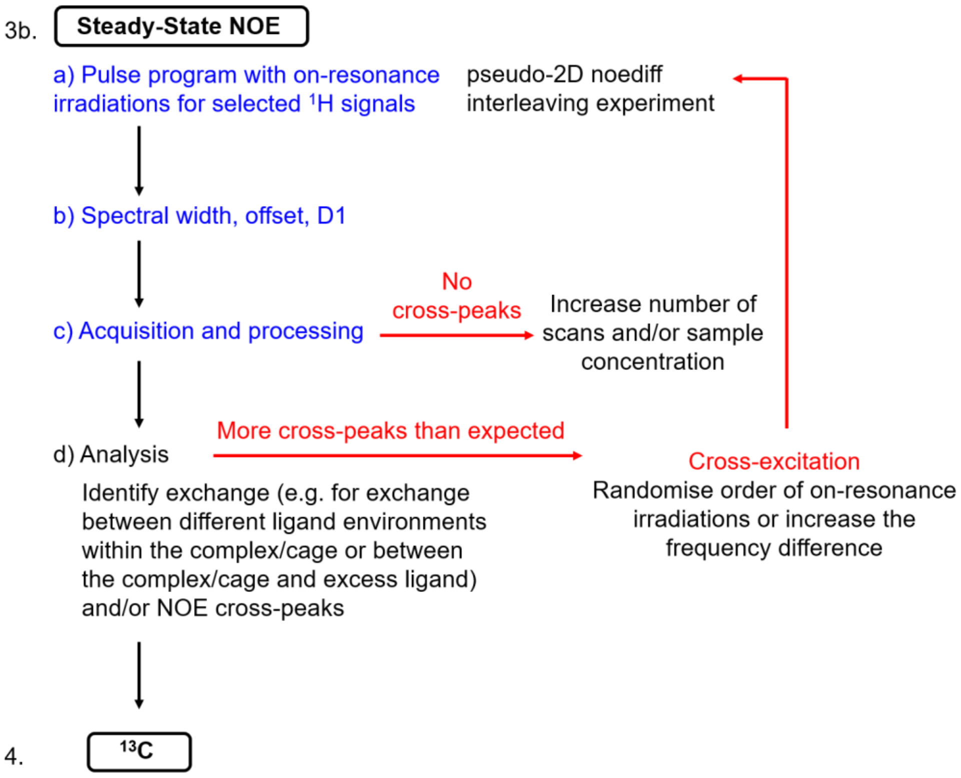

In a pseudo-2D noediff interleaving experiment, selected ^{1}H signals were irradiated and off-resonance irradiations were included at regular intervals as well as between on-resonance irradiations with similar chemical shifts to prevent cross-excitation. The spectra were recorded with 128 scans, 64 dummy scans, a spectral width of 140 ppm with an offset of 40 ppm, an acquisition time of 0.5 s and a repetition delay and saturation time (D1) of 2 s. Difference spectra were obtained by subtracting an off-resonance from an on-resonance spectrum.

1.1.1.5 ^{13}C NMR Spectroscopy

Flowchart 5. Workflow for the optimisation (blue text) and troubleshooting (red text) of paramagnetic ^{13}C NMR experiments (Toolbox experiment 4).

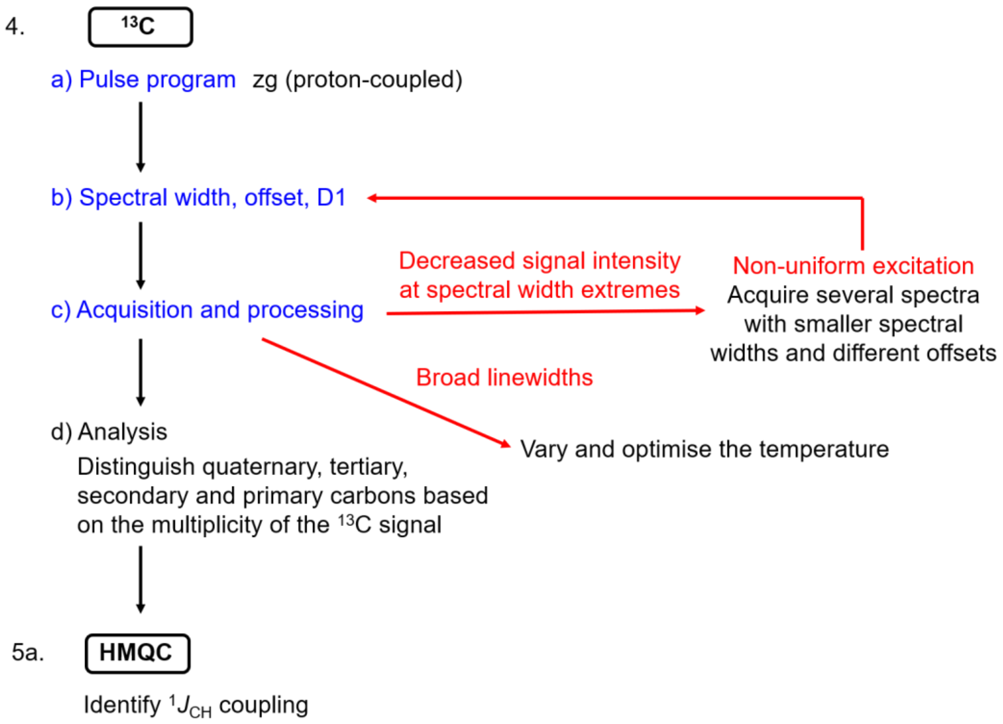

As the bandwidth of decoupling schemes is usually significantly smaller than the proton dispersion present in the analysed compounds, proton-coupled ^{13}C NMR spectra were recorded with the benefit that tertiary and quaternary carbons could be distinguished on the basis of their multiplicity (doublet vs singlet, respectively). Due to non-uniform excitation over the almost 900 ppm ^{13}C spectral width, two to three spectra with different offsets (e.g. -150, 220 and 600 ppm) were acquired to cover the whole range in smaller spectral widths. Acquisition times of 0.04-0.08 s and D1 values of 0.02-0.05 s were typically used.

1.1.1.6 HMQC

Flowchart 6. Workflow for the optimisation (blue text) and troubleshooting (red text) of paramagnetic HMQC NMR experiments (Toolbox experiment 5a).

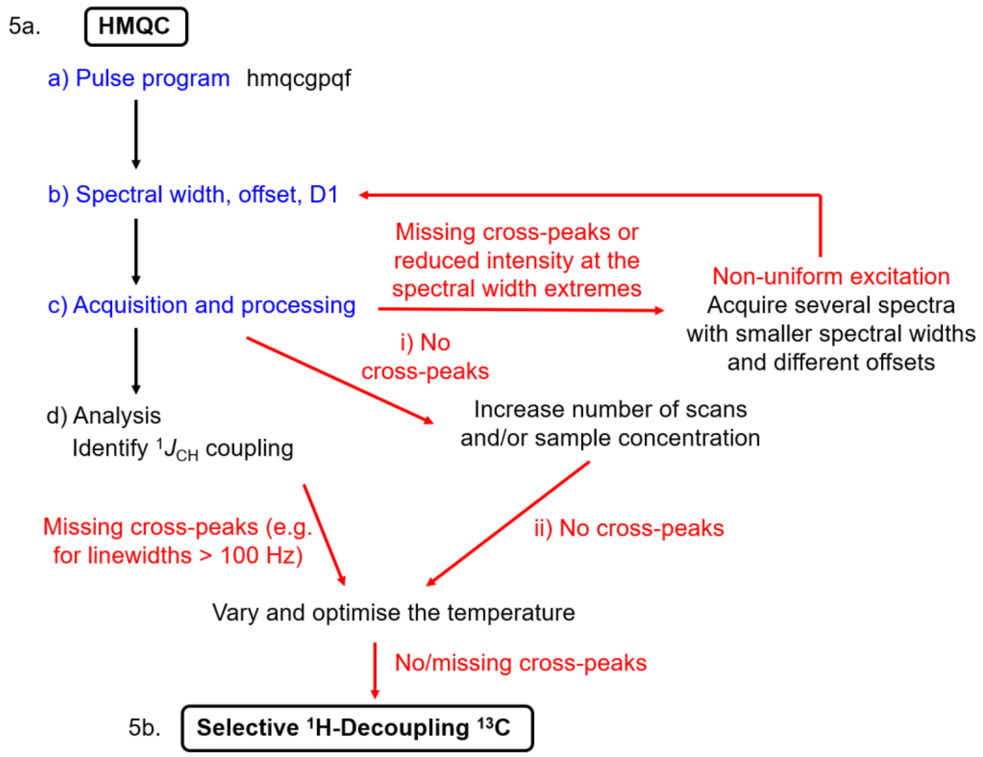

HMQC spectra were recorded using the hmqcgpqf pulse program with a D1 of 0.1 s, acquisition times of 0.008 and 0.002 s, increments of 2048 and 256 and a spectral width of 200 and 800 ppm for the F2 and F1 dimensions, respectively. At least two HMQC spectra were recorded with offsets typically at 20 ppm F2/350 ppm F1 and -60 ppm F2/250 ppm F1 to cover the entire spectral range.

1.1.1.7 Selective ^{1}H-Decoupling ^{13}C NMR Experiments

Flowchart 7. Workflow for the optimisation (blue text) and troubleshooting (red text) of paramagnetic selective ^{1}H-decoupling ^{13}C NMR experiments (Toolbox experiment 5b).

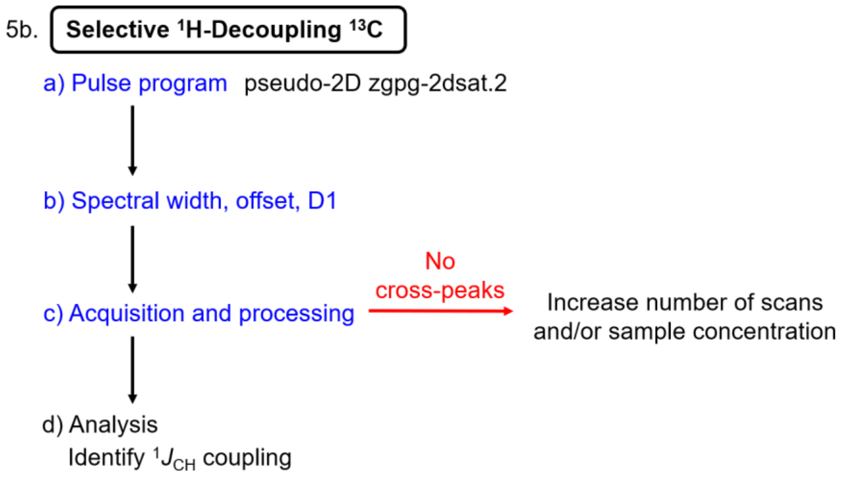

In a pseudo-2D zgpg-2dsat.2 experiment, the ^{1}H signals were individually decoupled with WALTZ decoupling in a series of acquired ^{13}C NMR spectra. $^{1}J_{CH}$ coupling was identified by the presence of a singlet for the proton-decoupled carbon signal rather than a doublet that was observed in the proton-coupled ^{13}C NMR spectrum (Section 1.1.1.5).

The pseudo-2D experiment was recorded with a repetition delay D1 of 0.1 s and acquisition time of 0.05 s. The spectra were processed using a line broadening of 30 Hz in the F2 dimension.

1.1.1.8 TOCSY

Spin lock based experiments such as TOCSY are difficult to adapt for paramagnetic complexes since they typically use longer low power pulses but the high power pulses required for uniform excitation over the large spectral range result in overheating of the sample and lock instabilities.[6, 16, 20] A TOCSY spectrum of complex **1a** was recorded using a mlevph pulse program and lock instabilities were observed during the acquisition. Furthermore, exchange rather than long-range coupling cross-peaks were observed and therefore, further optimisation of the parameters was not investigated.

1.2 Mass Spectrometry

Electron Ionisation (EI) mass spectrometry was carried out on a Jeol AccuTOF. High resolution electrospray ionisation mass spectrometry (ESI-MS) was carried out on a Thermo Scientific Q Exactive Plus (spray voltage 3-4 eV, capillary temperature 40-50 °C) infused from a Harvard syringe pump at a rate of 5-10 µL per minute.

1.3 X-Ray Crystallography

Data collection was performed with an Imaging Plate Diffraction System (IPDS-2) from STOE & CIE, Darmstadt, Germany using MoKα_1 radiation. Structure solution was performed with SHELXT[1] and structure refinement was done with SHELXL-2018.[2] A numerical absorption correction was performed using programs X-RED and X-SHAPE of the program package X-Area.[3]

2 Ligand Synthesis

In order to enable comparison between the complexes with the various 2-(2-pyridyl)quinoline derivatives, the following labelling scheme has been used throughout for the ligands and

complexes. Labelling of the protons and carbons begins on the 6-position of the 2-pyridyl spin system (*a*-*e*) before continuing on the 2-quinoline (*f*-*n*) spin system and the protons and carbons on the substituent in the 5'- (*b*) or 6-position (*k*) are labelled with *o* onwards.

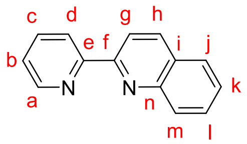

2.1 Unsubstituted Ligand

2-(2-Pyridyl)quinoline was synthesised according to Scheme S1 by adapting the literature procedure of Shim *et al.*[4]

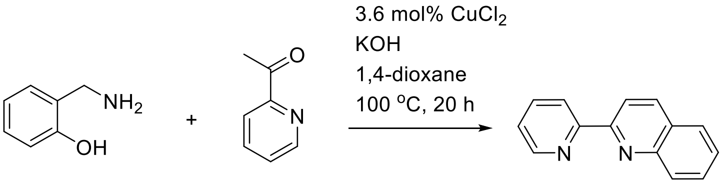

Scheme S1. Synthesis of 2-(2-pyridyl)quinoline.

2.1.1 2-(2-Pyridyl)quinoline

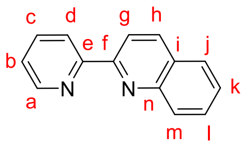

2-Aminobenzyl alcohol (0.25 g, 2.0 mmol), 2-acetylpyridine (0.28 mL, 2.5 mmol), $CuCl_2$ (0.0136 g, 0.07 mmol, 3.6 mol%), and KOH (0.34 g, 6.0 mmol) were suspended in dry 1,4-dioxane (10 mL) and the reaction mixture was heated at 100 °C under air for 20 h. The reaction mixture was cooled to room temperature, diluted with water (50 mL) and extracted with EtOAc (3 x 20 mL). The organic extracts were combined, dried over $MgSO_4$, filtered and the solvent was removed *in vacuo*. The residue was dry loaded onto silica gel and purified by column chromatography (4:1 cyclohexane/EtOAc) to give the desired product as a white solid.

Yield: 254 mg (1.23 mmol, 62%)

The analytical data was consistent with literature data.[5]

^{1}H NMR (500 MHz, $CDCl_3$, 298 K) δ (ppm): 8.74 (ddd, ^3J = 4.7 Hz, ^4J = 1.7 Hz, ^5J = 0.9 Hz, 1H, H_a), 8.66 (dt, ^3J = 7.7 Hz, ^4J = 1.1 Hz 1H, H_d), 8.57 (d, ^3J = 8.6 Hz, 1H, H_g), 8.29 (d, ^3J = 8.6 Hz, 1H, H_h), 8.19 (d, ^3J = 8.5 Hz, 1H, H_m), 7.89 (td, ^3J = 7.7 Hz, ^4J =1.7 Hz, 1H, H_c), 7.85 (dd, ^3J = 8.2 Hz, ^4J = 1.1 Hz, 1H, H_j), 7.74 (unresolved ddd, ^3J = 8.4 Hz, ^3J = 6.9 Hz, ^4J = 1.6 Hz, 1H, H_l), 7.55 (unresolved ddd, ^3J = 8.1 Hz, ^3J = 6.9 Hz, ^4J = 1.2 Hz, 1H, H_k), 7.36 (ddd, ^3J = 7.7 Hz, ^3J = 4.7 Hz, ^4J = 1.2 Hz, 1H, H_b).

^{13}C NMR (121 MHz, $CDCl_3$, 298 K) δ (ppm): 156.3 (C_e), 156.1(C_f), 149.1 (C_a), 147.9 (C_n), 137.0 (C_c), 136.9 (C_h), 129.8 (C_m), 129.6 (C_l), 128.3 (C_i), 127.6 (C_j), 126.8 (C_k), 124.1 (C_b), 121.9 (C_d), 119.0 (C_g).

HRMS (EI, 70 eV) *m/z*: 206.08479 $[M]^+$ (calculated: 206.08440 for $C_{14}H_{10}N_2$).

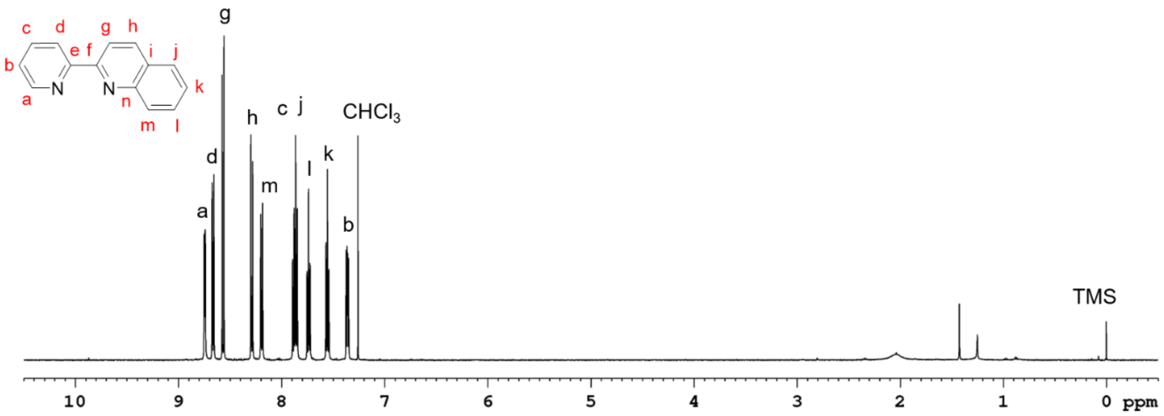

Figure S1. ^{1}H NMR spectrum (500 MHz, $CDCl_3$, 298 K) of 2-(2-pyridyl)quinoline.

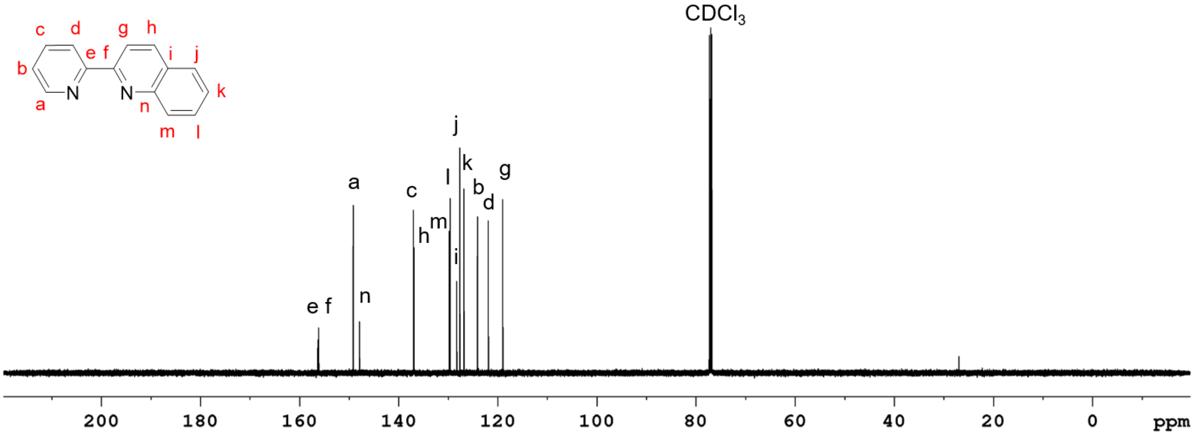

Figure S2. ^{13}C NMR spectrum (121 MHz, $CDCl_3$, 298 K) of 2-(2-pyridyl)quinoline.

2.2 5'-Substituted Pyridylquinoline Ligand Derivatives

2-(5'-Bromopyridin-2'-yl)quinoline was synthesised in two steps according to Scheme S2 by adapting literature procedures.[4, 6]

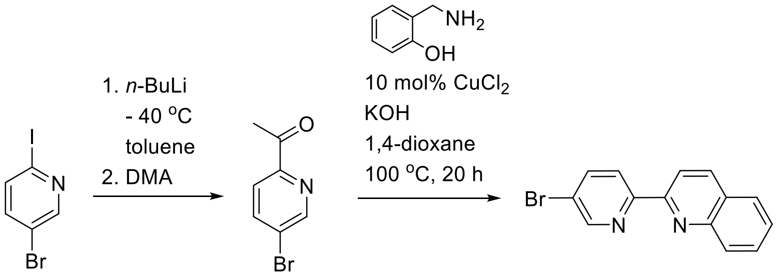

Scheme S2. Synthesis of 2-(5'-bromopyridin-2'-yl)quinoline.

The remaining ligands were prepared by palladium-catalysed Sonogashira or Suzuki coupling reactions (Schemes S3-S4).

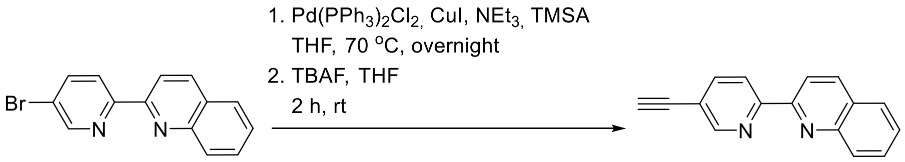

Scheme S3. Synthesis of 2-(5'-ethynylpyridin-2'-yl)quinoline.

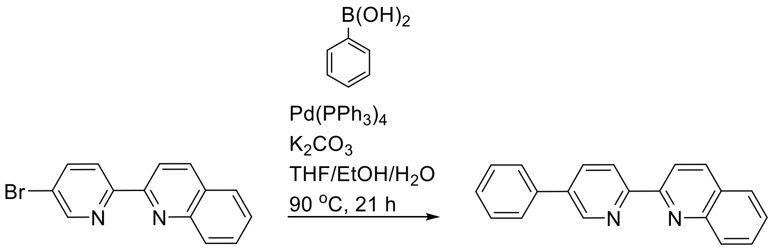

Scheme S4. Synthesis of 2-(5'-phenylpyridin-2'-yl)quinoline.

2.2.1 1-(5-Bromopyridin-2-yl)ethanone

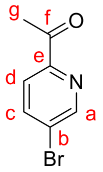

2-Iodo-5-bromopyridine (1.80 g, 6.33 mmol) was dissolved in anhydrous toluene (60 mL) and cooled to -40 °C. *n*-Butyllithium (2.50 mL, 6.33 mmol, 2.5 M in hexane) was added dropwise to the solution. The solution was stirred for 1 h at -40 °C then *N,N*-dimethylacetamide (1.00 mL, 10.8 mmol) was added dropwise to the solution. After stirring for another 30 min at -40 °C, the solution was allowed to warm up to room temperature over 30 min. The reaction was quenched with NH_4Cl solution (20 mL). The organic phases were separated and the aqueous phase was extracted twice with dichloromethane (30 mL). The organic layers were combined, washed with sat. NaCl solution (10 mL), dried over $MgSO_4$ and the solvent was removed *in vacuo* to give a brown solid. The product was used without further purification.

Yield: 1.16 g (5.78 mmol, 91%)

R_f (cyclohexane/EtOAc 4:1): 0.68

^{1}H NMR (500 MHz, $CDCl_3$, 298 K) δ (ppm): 8.73 (dd, ^4J = 2.2 Hz, ^5J = 0.7 Hz, 1H, H_a), 7.98 – 7.91 (m, 2H, H_c, H_d), 2.70 (s, 3H, H_g).

^{13}C NMR (125 MHz, $CDCl_3$, 298 K) δ (ppm): 199.2 (C_f), 151.8 (C_e), 150.2 (C_a), 139.5 (C_c), 125.3 (C_b), 122.9 (C_d), 25.7 (C_g).

HRMS (EI, 70 eV) *m/z*: 198.96331 $[M]^+$ (calculated: 198.96328 for $C_7H_6{}^{79}BrNO$), 200.96146 $[M]^+$ (calculated: 200.96123 for $C_7H_6{}^{81}BrNO$).

M. p.: 112 °C Lit.[6]: 109-111 °C

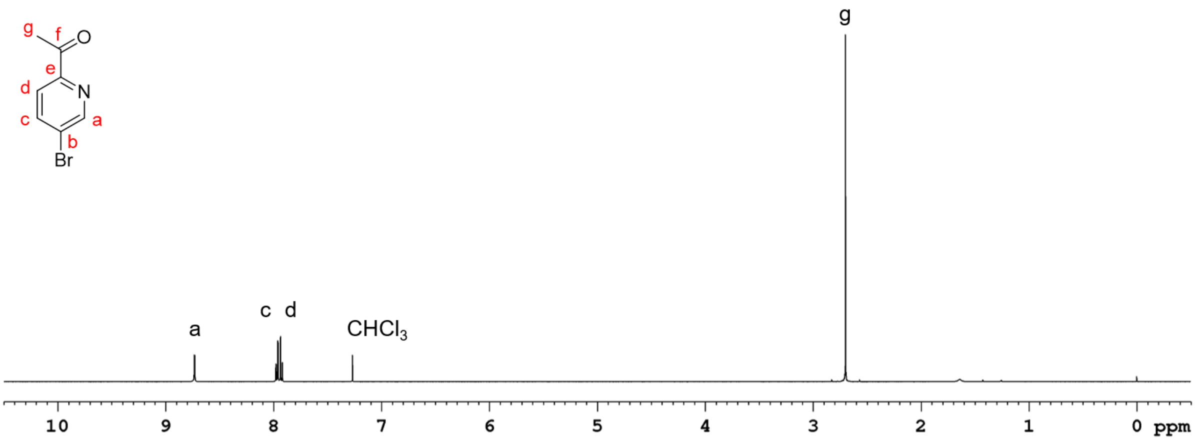

Figure S3. ^{1}H NMR spectrum (500 MHz, $CDCl_3$, 298 K) of 2-(5-bromopyridin-2-yl)ethanone.

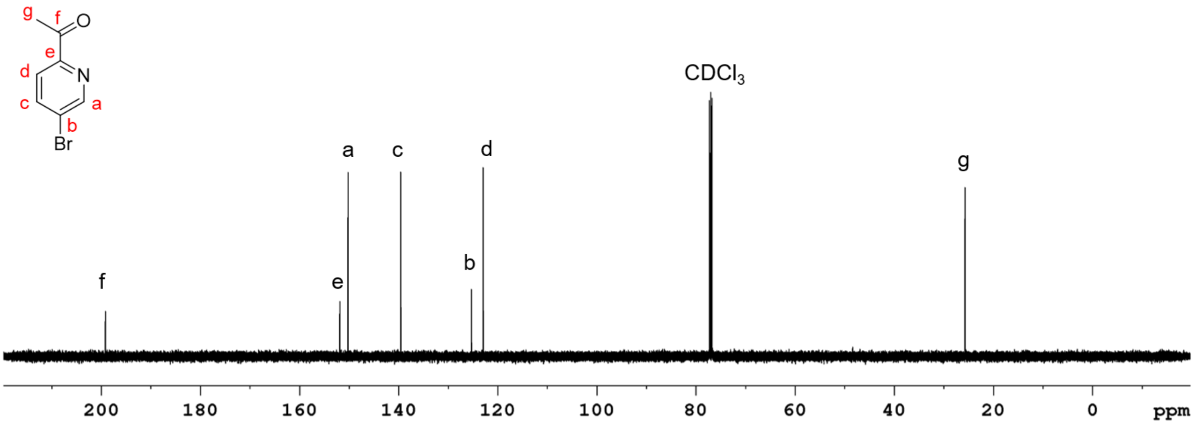

Figure S4. ^{13}C NMR spectrum (125 MHz, $CDCl_3$, 298 K) of 2-(5-bromopyridin-2-yl)ethanone.

2.2.2 2-(5'-Bromopyridin-2'-yl)quinoline

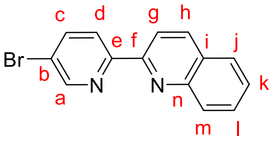

2-Aminobenzyl alcohol (514 mg, 4.17 mmol), 1-(5-bromopyridin-2-yl)ethanone (1.00 g, 5.00 mmol), $CuCl_2$ (56.1 mg, 0.42 mmol, 10 mol%) and KOH (737 mg, 13.1 mmol) were suspended in anhydrous 1,4-dioxane (20 mL) and the reaction mixture was heated at 100 °C for 20 h. The solution was filtered through Celite® and washed with EtOAc (200 mL). The organic layer was washed with sat. NH_4Cl solution (100 mL) and water (100 mL). The layers were separated and the combined aqueous layer was extracted with EtOAc (3 x 50 mL). The combined organic layers were dried over $MgSO_4$ and the solvent was removed *in vacuo*. The crude product was purified by column chromatography (silica gel, 10-20% EtOAc/cyclohexane) to give a white solid.

Yield: 573 mg (2.02 mmol, 48%)

R_f (cyclohexane/EtOAc 4:1): 0.65

^{1}H NMR (500 MHz, $CDCl_3$, 298 K) δ (ppm): 8.78 (dd, ^{4}J = 2.3 Hz, ^{5}J = 0.6 Hz, 1H, H_a), 8.58 (d, ^{3}J = 8.5 Hz, 1H, H_d), 8.53 (d, ^{3}J = 8.6 Hz, 1H, H_g), 8.28 (d, ^{3}J = 8.6 Hz, 1H, H_h), 8.17 (d, ^{3}J = 8.5 Hz, 1H, H_m), 7.99 (dd, ^{3}J = 8.5 Hz, ^{4}J = 2.3 Hz, 1H, H_c), 7.85 (dd,

^3J = 8.1 Hz, ^4J = 1.3 Hz, 1H, H_j), 7.74 (ddd, ^3J = 8.5 Hz, ^3J = 6.9 Hz, ^4J = 1.3 Hz, 1H, H_l), 7.54 (ddd, ^3J = 8.1 Hz, ^3J = 6.9 Hz, ^4J = 1.1 Hz, 1H, H_k).

^{13}C NMR (125 MHz, $CDCl_3$, 298 K) δ (ppm): 155.1 (C_f), 154.7 (C_e), 150.2 (C_a), 147.8 (C_n), 139.5 (C_c), 137.0 (C_h), 129.8 (C_l), 129.7 (C_m), 128.3 (C_i), 127.6 (C_j), 127.0 (C_k), 123.1 (C_d), 121.6 (C_b), 118.7 (C_g).

HRMS (EI, 70 eV) *m/z*: 283.99509 $[M]^+$ (calculated: 283.99491 for $C_{14}H_9{}^{79}BrN_2$), 285.99253 $[M]^+$ (calculated: 285.99286 for $C_{14}H_9{}^{81}BrN_2$).

M. p.: 146 °C

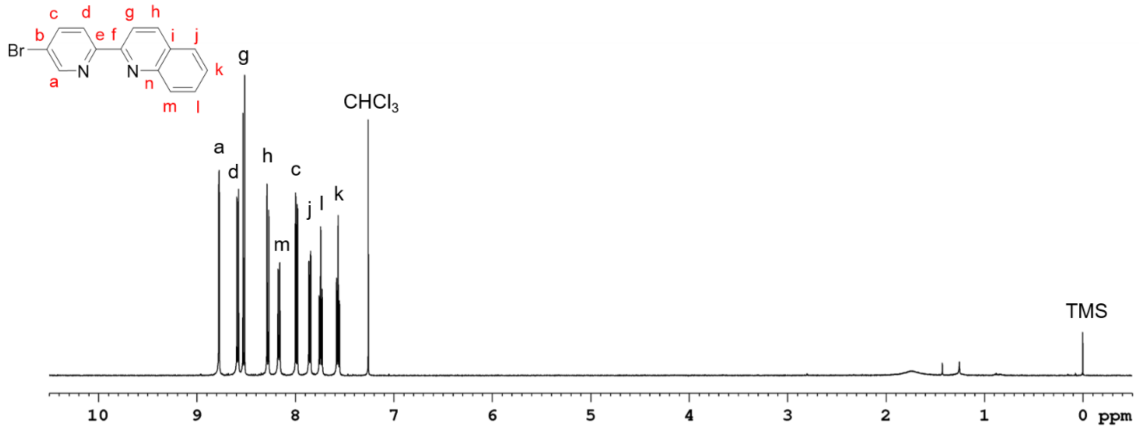

Figure S5. ^{1}H NMR spectrum (500 MHz, $CDCl_3$, 298 K) of 2-(5'-bromopyridin-2'-yl)quinoline.

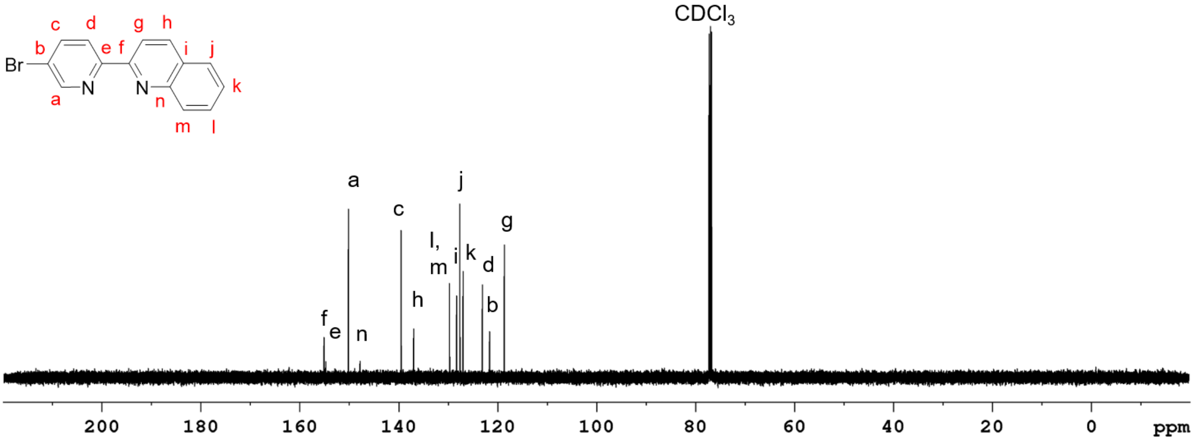

Figure S6. ^{13}C NMR spectrum (125 MHz, $CDCl_3$, 298 K) of 2-(5'-bromopyridin-2'-yl)quinoline.

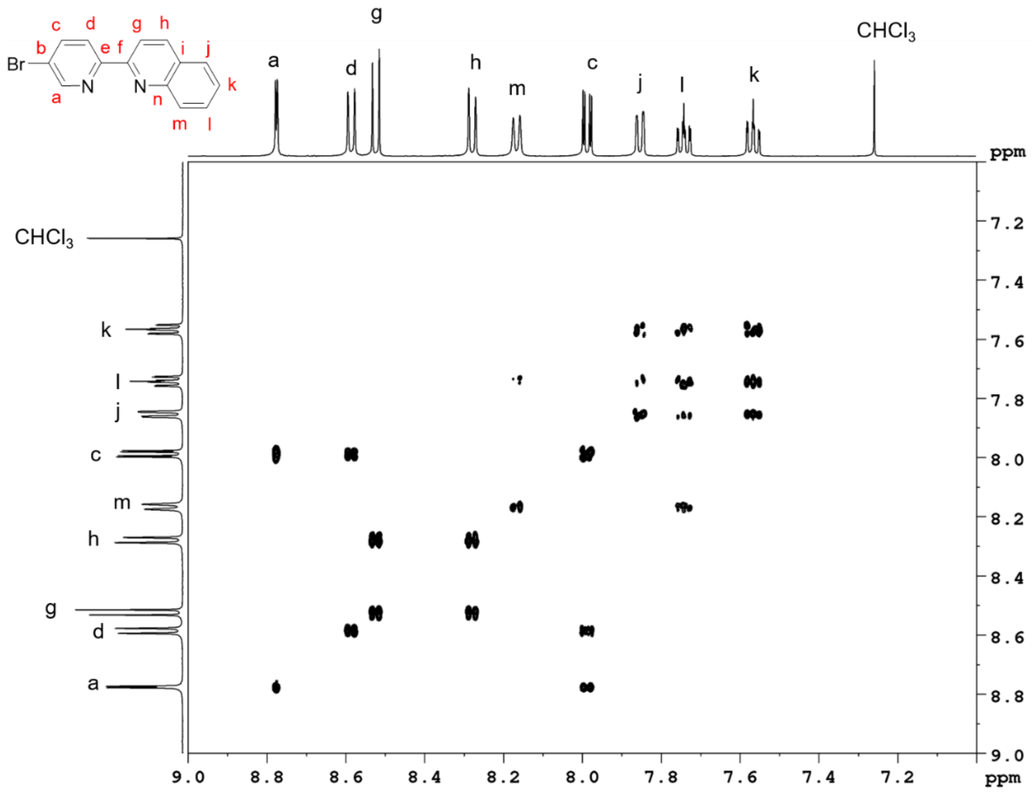

Figure S7. ^{1}H-^{1}H COSY NMR spectrum (500 MHz, $CDCl_3$, 298 K) of 2-(5'-bromopyridin-2'-yl)quinoline.

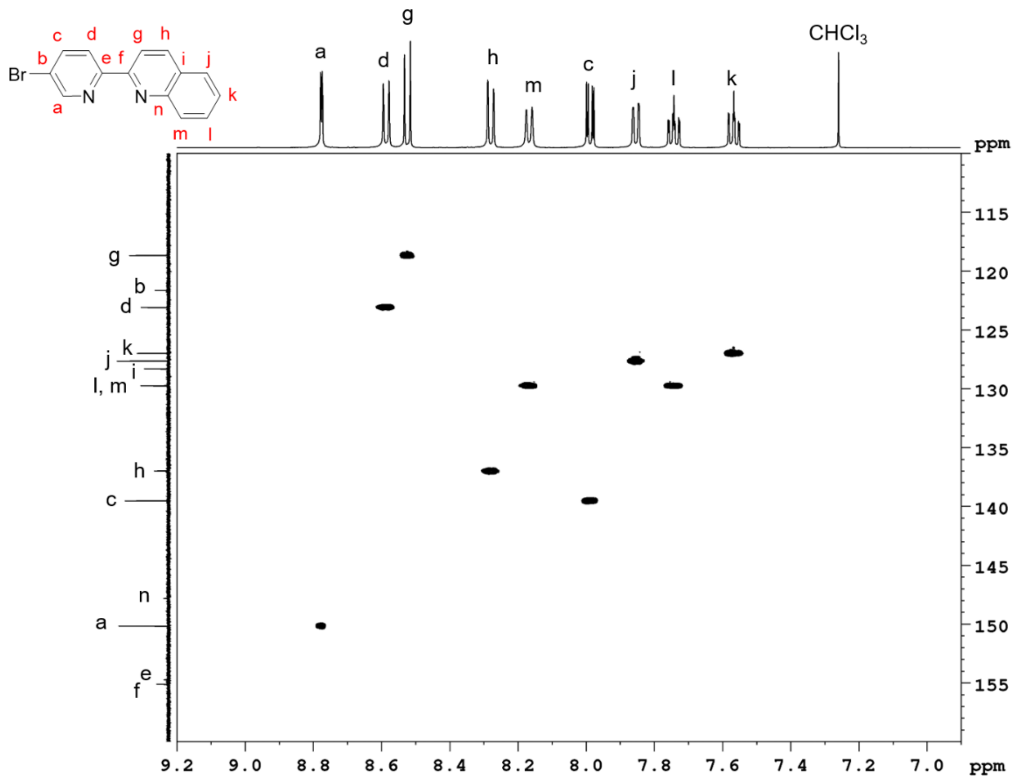

Figure S8. ^{1}H-^{13}C HSQC NMR spectrum (500 MHz/125 MHz, $CDCl_3$, 298 K) of 2-(5'-bromopyridin-2'-yl)quinoline.

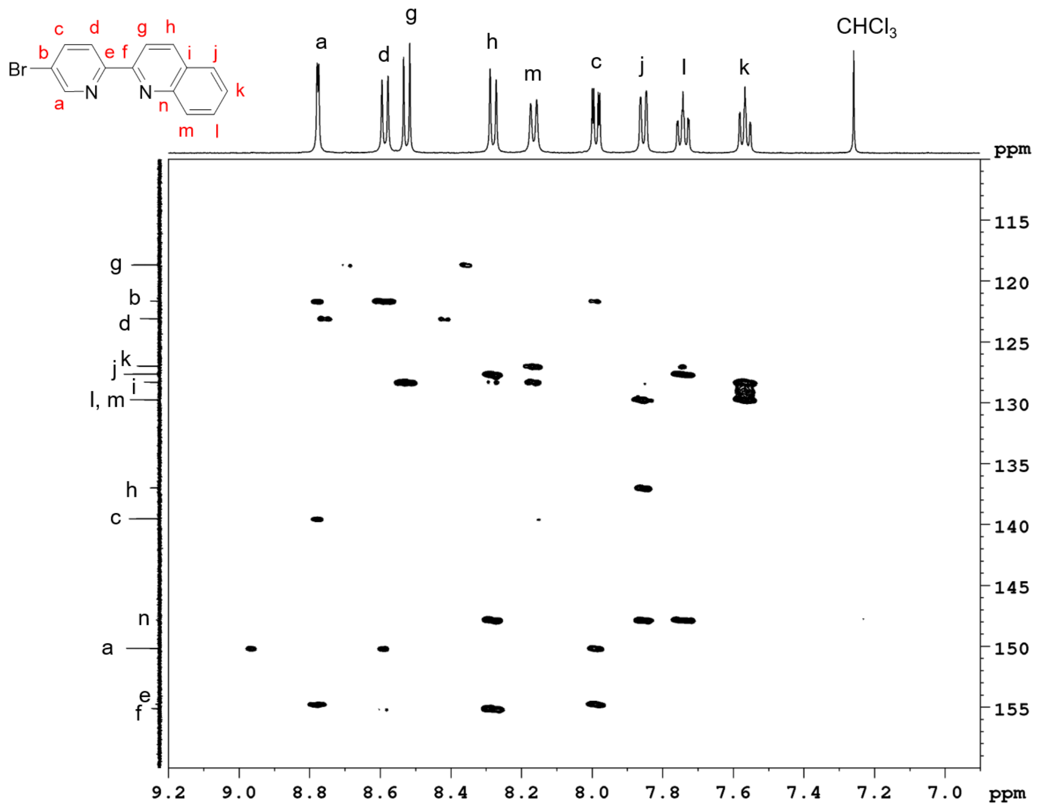

Figure S9. ^{1}H-^{13}C HMBC NMR spectrum (500 MHz/125 MHz, $CDCl_3$, 298 K) of 2-(5'-bromopyridin-2'-yl)quinoline.

2.2.3 2-(5'-Ethynylpyridin-2'-yl)quinoline

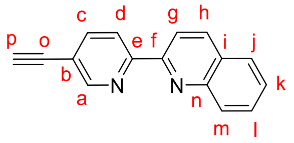

2-(5'-Bromopyridin-2'-yl)quinoline (0.116 g, 0.407 mmol), CuI (7.80 mg, 40.9 µmol) and $Pd(PPh_3)_2Cl_2$ (14.4 mg, 20.5 µmol) were dissolved in anhydrous THF (5 mL) under a nitrogen atmosphere. TMSA (1.0 mL, 0.708 mmol) and 1.0 mL NEt_3 were added to the solution and the reaction mixture was stirred at 70 °C overnight. After cooling to room temperature, TBAF solution (1 mL, 1 M in THF) was added and the reaction mixture was stirred for an additional 2 h at room temperature. The solvent was removed under reduced pressure and the crude material was purified by flash column chromatography (9:1 Cyclohexane/EtOAc). The product was obtained as a colourless solid.

Yield: 56.0 mg (0.244 mmol, 69 %)

R*f* (9:1 Cyclohexane/EtOAc): 0.36

^{1}H NMR (500 MHz, $CDCl_3$, 298 K) δ (ppm): 8.83 (dd, ^{4}J = 2.1 Hz, ^{5}J = 0.8 Hz, 1H, H_a), 8.67 (dd, ^{3}J = 8.2 Hz, ^{5}J = 0.8 Hz, 1H, H_d), 8.57 (d, ^{3}J = 8.6 Hz, 1H, H_g), 8.30 (d, ^{3}J = 8.6 Hz, 1H, H_h), 8.18 (d, ^{3}J = 8.4 Hz, 1H, H_m), 7.96 (dd, ^{3}J = 8.2 Hz, ^{4}J = 2.1 Hz, 1H, H_c), 7.86 (d, ^{3}J = 8.1 Hz, 1H, H_j), 7.75 (ddd, ^{3}J = 8.4 Hz, ^{3}J = 6.9 Hz, ^{4}J = 1.4 Hz, 1H, H_l), 7.57 (ddd, ^{3}J = 8.1 Hz, ^{3}J = 6.9 Hz, ^{4}J = 1.2 Hz, 1H, H_k), 3.32 (s, 1H, H_p).

^{13}C NMR (125 MHz, $CDCl_3$, 298 K) δ (ppm): 155.6 (C_f), 155.3 (C_e), 152.2 (C_a), 147.9 (C_n), 140.0 (C_c), 136.9 (C_h), 129.8 (C_m), 129.7 (C_l), 128.3 (C_i), 127.6 (C_j), 127.0 (C_k), 121.0 (C_d), 119.4 (C_b), 119.0 (C_g), 81.5 (C_p), 80.7 (C_o).

HRMS (EI, 70 eV) *m/z*: 230.08383 (calculated for $C_{16}H_{10}N_2$: 230.08440)

M. p.: 158 °C

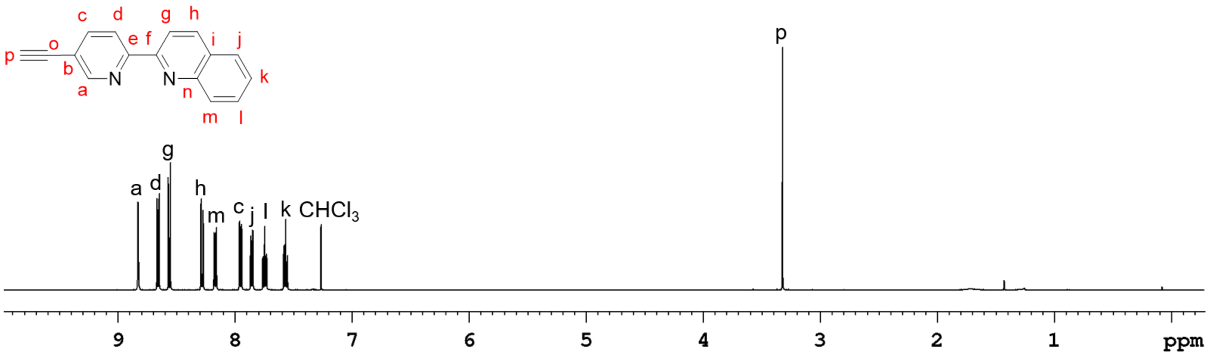

Figure S10. ^{1}H NMR spectrum (500 MHz, $CDCl_3$, 298 K) of 2-(5'-ethynylpyridin-2'-yl)quinoline.

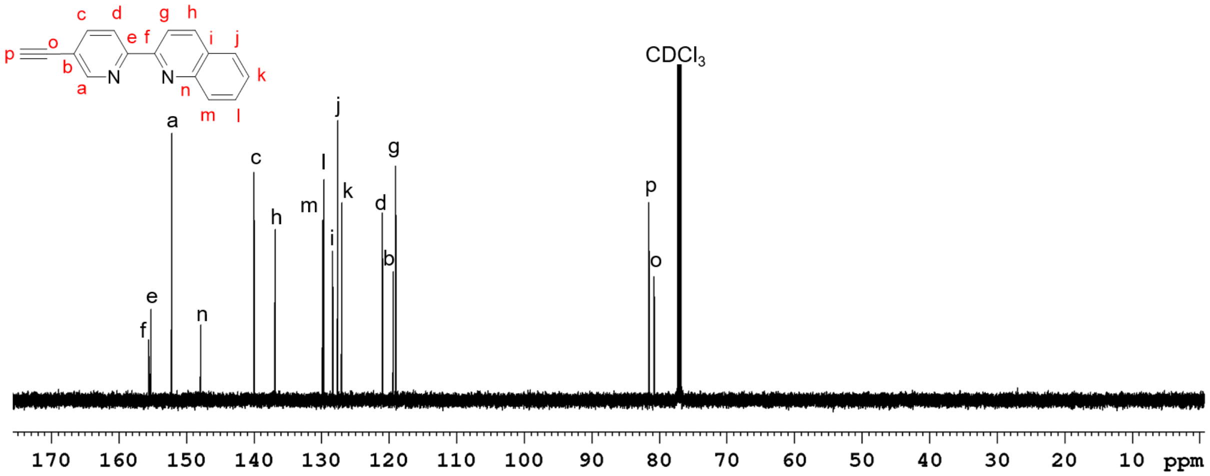

Figure S11. ^{13}C NMR spectrum (125 MHz, $CDCl_3$, 298 K) of 2-(5'-ethynylpyridin-2'-yl)quinoline.

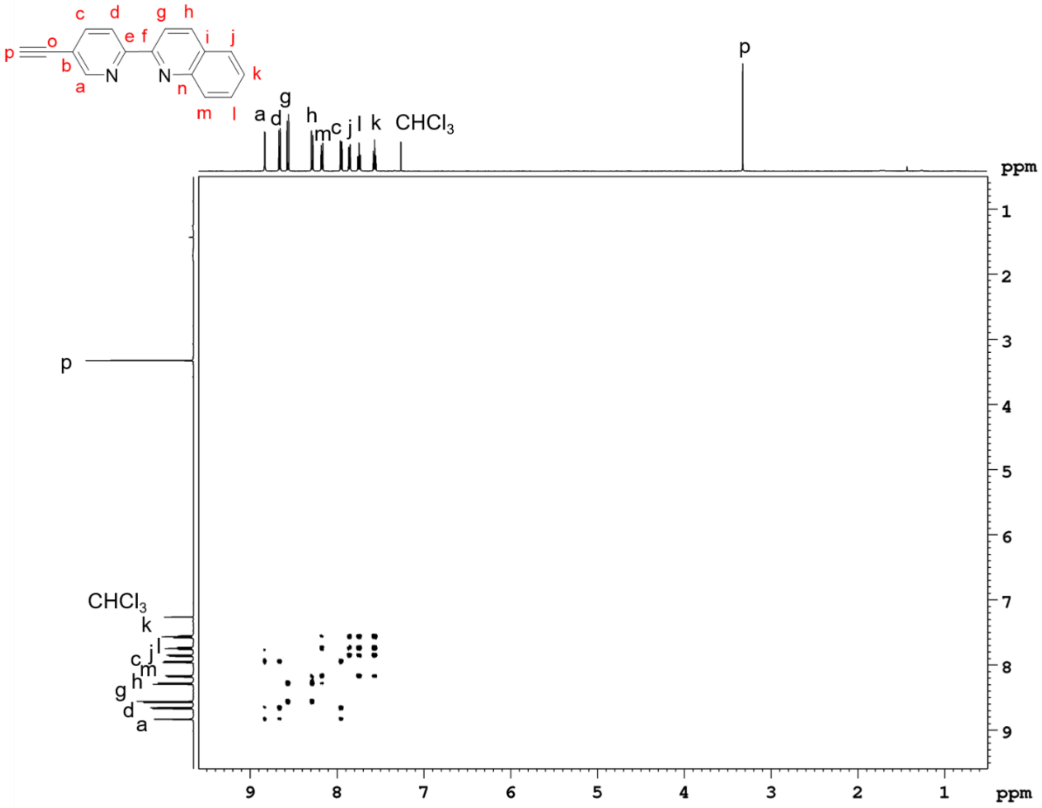

Figure S12. ^{1}H-^{1}H COSY NMR spectrum (500 MHz MHz, $CDCl_3$, 298 K) of 2-(5'-ethynylpyridin-2'-yl)quinoline.

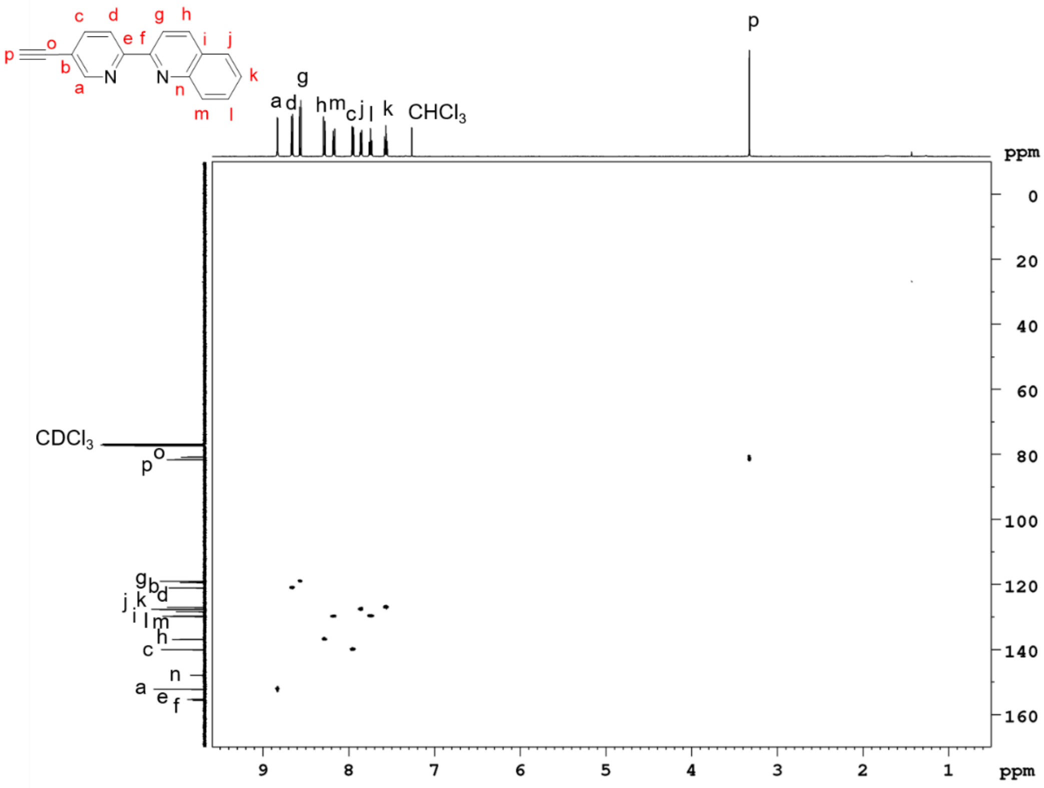

Figure S13. ^{1}H-^{13}C HSQC NMR spectrum (500 MHz/125 MHz, $CDCl_3$, 298 K) of 2-(5'-ethynylpyridin-2'-yl)quinoline.

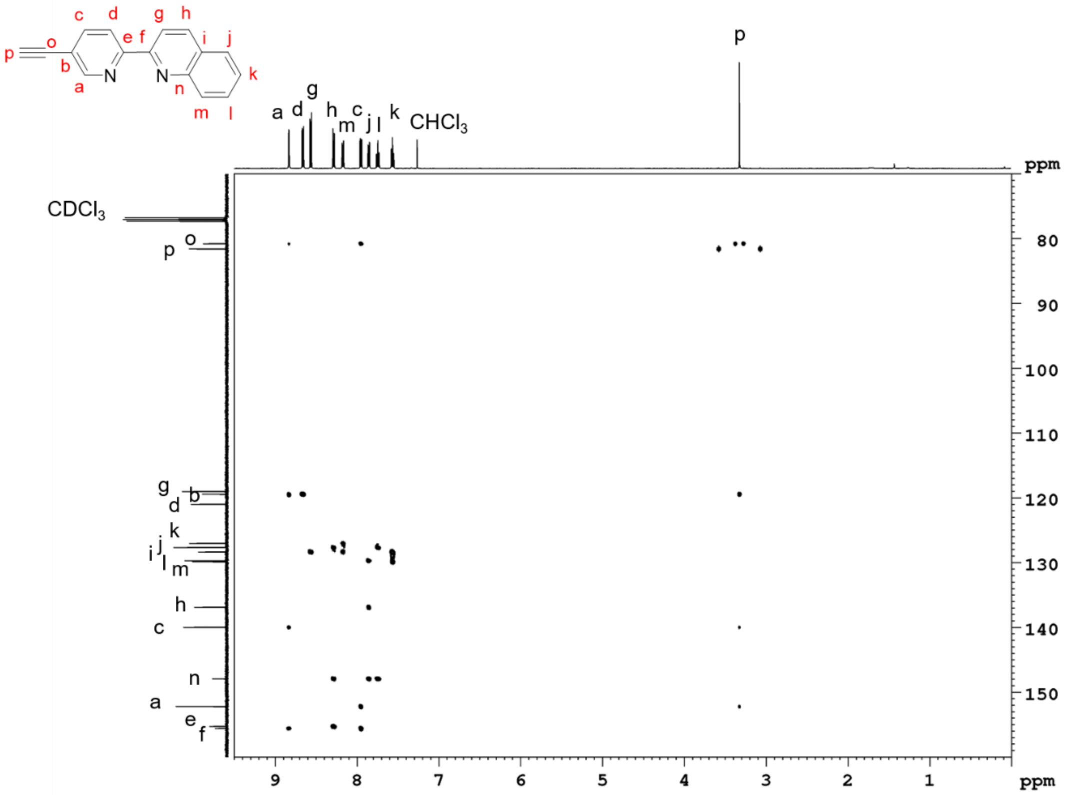

Figure S14. ^{1}H-^{13}C HMQC NMR spectrum (500 MHz/125 MHz, $CDCl_3$, 298 K) of 2-(5'-ethynylpyridin-2'-yl)quinoline.

2.2.4 2-(5'-Phenylpyridin-2'-yl)quinoline

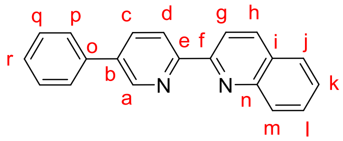

2-(5'-Bromopyridin-2'-yl)quinoline (100 mg, 350 µmol), phenylboronic acid (43 mg, 350 µmol) and K_2CO_3 (122 mg, 880 µmol) were dissolved in a mixture of THF (8 mL), water (4 mL) and ethanol (6.5 mL) in a three-neck flask and the mixture was degassed by three freeze-pump-thaw cycles. To this solution was added $Pd(PPh_3)_4$ (20.0 mg, 5 mol%) and the reaction mixture was heated at 90 °C for 21 h under a nitrogen atmosphere. After cooling to room temperature, the solution was washed with water (30 mL), half-concentrated K_2CO_3 solution (15 mL sat. K_2CO_3 solution diluted with 15 mL H_2O) and water (30 mL). The organic extracts were combined, dried over $MgSO_4$ and the solvent was removed *in vacuo*. The crude product was purified by column chromatography (silica gel, 6-10% EtOAc/cyclohexane) to give a crystalline, colourless solid.

Yield: 64 mg (0.226 mmol, 66%)

R_f (cyclohexane/EtOAc 4:1): 0.29

^{1}H NMR (500 MHz, $CDCl_3$, 298 K) δ (ppm): 8.98 (dd, ^{4}J = 2.4 Hz, ^{5}J = 0.7 Hz, 1H, H_a), 8.74 (dd, ^{3}J = 8.2 Hz, ^{5}J = 0.7 Hz, 1H, H_d), 8.61 (d, ^{3}J = 8.6 Hz, 1H, H_g), 8.30 (d, ^{3}J = 8.6 Hz, 1H, H_h), 8.21 (d, ^{3}J = 8.5 Hz, 1H, H_m), 8.08 (dd, ^{3}J = 8.2 Hz, ^{4}J = 2.4 Hz, 1H, H_c), 7.86 (d, ^{3}J = 8.1 Hz, ^{4}J = 1.4 Hz, 1H, H_j), 7.75 (ddd, ^{3}J = 8.5 Hz, ^{3}J = 6.9 Hz, ^{4}J = 1.4 Hz, 1H, H_l), 7.70 – 7.67 (m, 2H, H_p), 7.56 (ddd, ^{3}J = 8.1 Hz, ^{3}J = 6.9 Hz, ^{4}J = 1.2 Hz, 1H, H_k), 7.54 – 7.50 (m, 2H, H_q), 7.46 – 7.42 (m, 1H, H_r).

^{13}C NMR (125 MHz, $CDCl_3$, 298 K) δ (ppm): 155.8 (C_f), 155.1 (C_e), 148.0 (C_n), 147.6 (C_a), 137.6 (C_o), 136.9 (C_h), 136.8 (C_b), 135.3 (C_c), 129.8 (C_m), 129.6 (C_l), 129.2 (C_q), 128.3 (C_r, C_i), 127.6 (C_j), 127.2 (C_p), 126.8 (C_k), 121.8 (C_d), 119.0 (C_g).

HRMS (EI, 70 eV) *m/z*: 282.11545 $[M]^+$ (calculated: 282.11570 for $C_{20}H_{14}N_2$).

M. p.: 147 °C

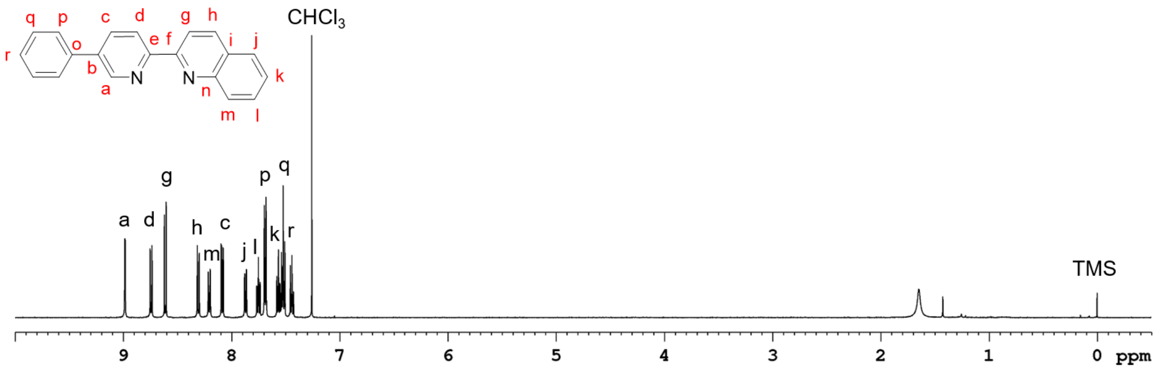

Figure S15. ^{1}H NMR spectrum (500 MHz, $CDCl_3$, 298 K) of 2-(5'-phenylpyridin-2'-yl)quinoline.

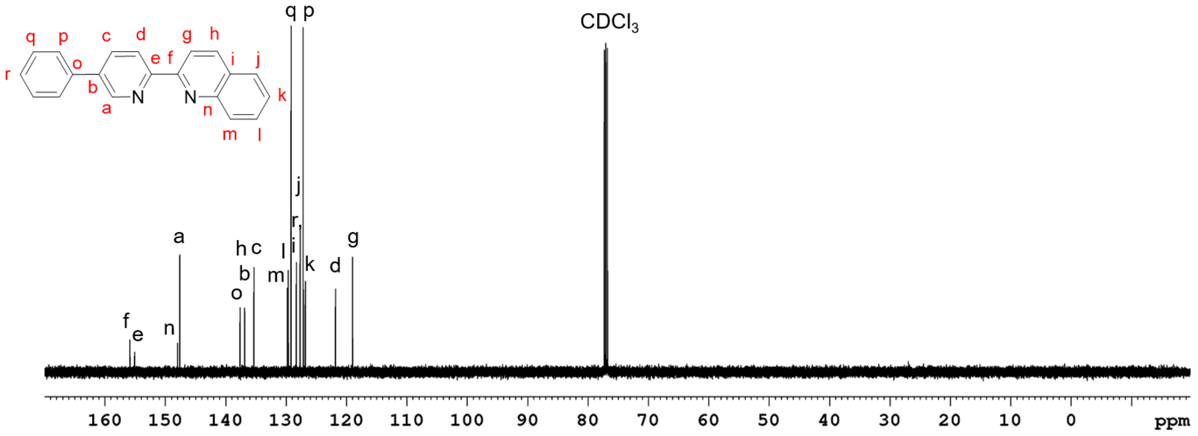

Figure S16. ^{13}C NMR spectrum (125 MHz, $CDCl_3$, 298 K) of 2-(5'-phenylpyridin-2'-yl)quinoline.

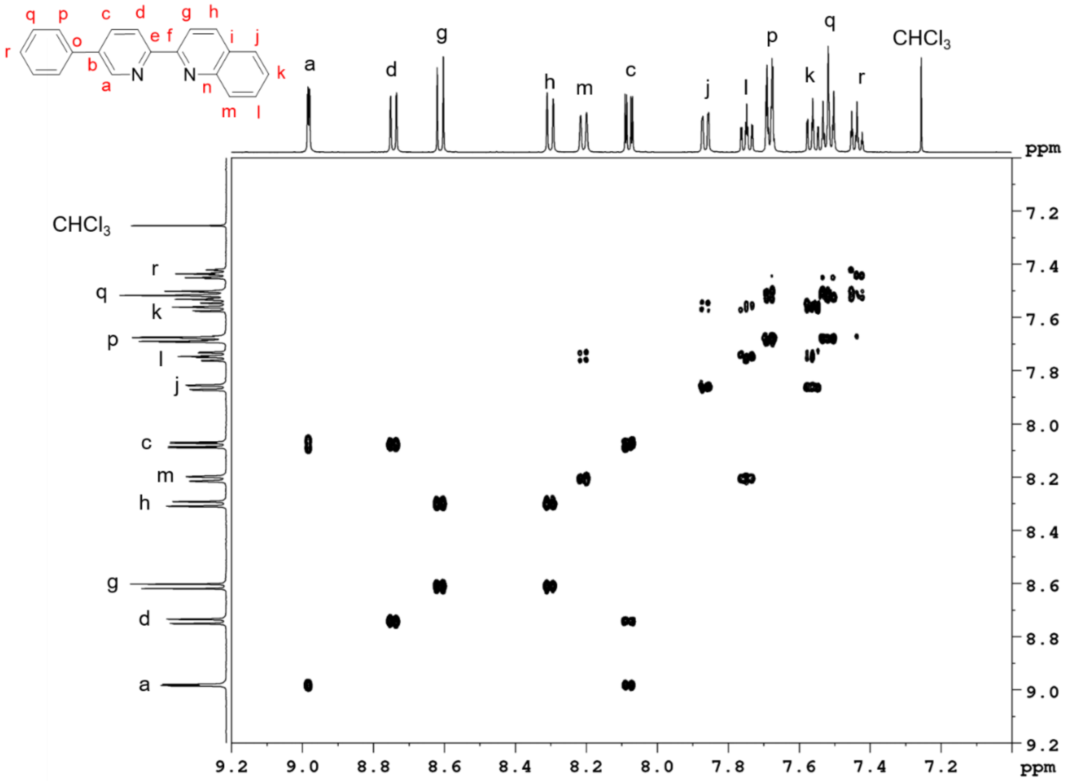

Figure S17. ^{1}H-^{1}H COSY NMR spectrum (500 MHz, $CDCl_3$, 298 K) of 2-(5'-phenylpyridin-2'-yl)quinoline.

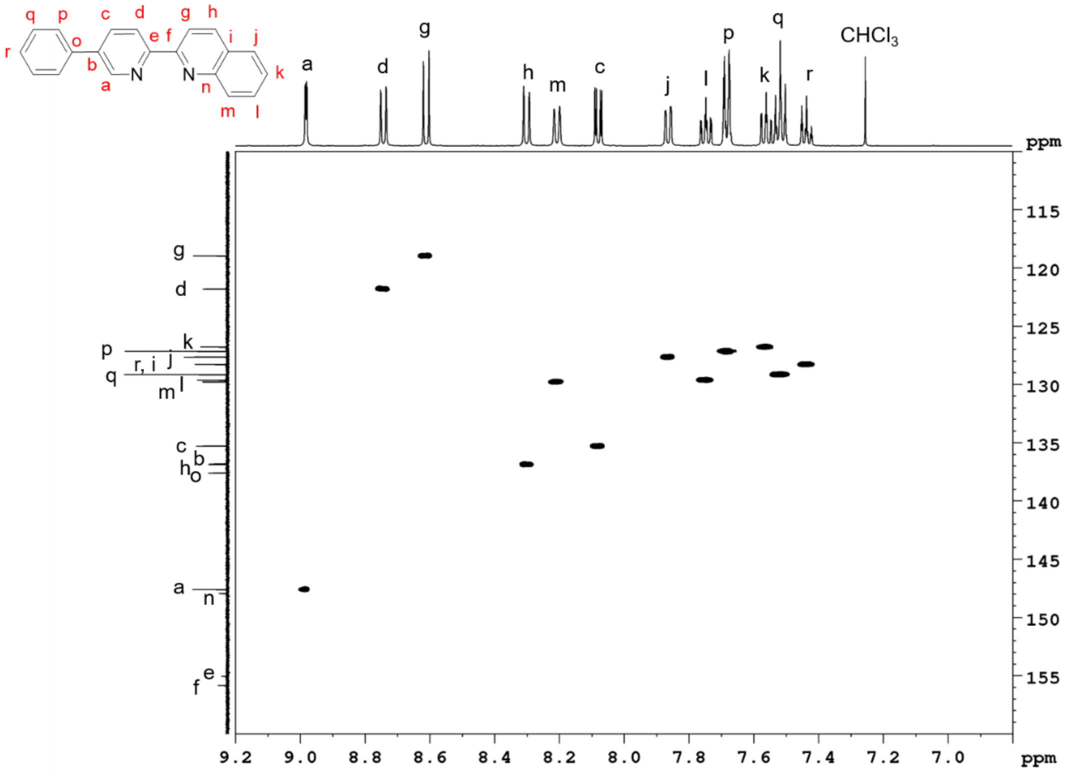

Figure S18. ^{1}H-^{13}C HSQC NMR spectrum (500 MHz/125 MHz, $CDCl_3$, 298 K) of 2-(5'-phenylpyridin-2'-yl)quinoline.

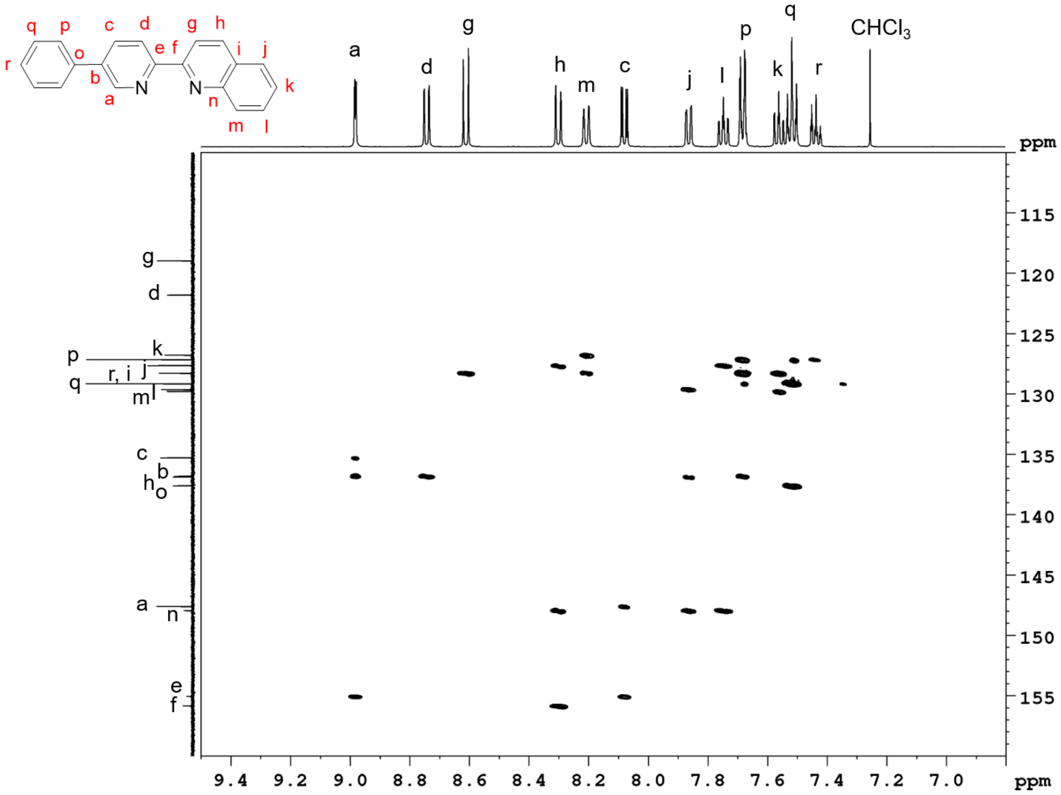

Figure S19. ^{1}H-^{13}C HMBC NMR spectrum (500 MHz/125 MHz, $CDCl_3$, 298 K) of 2-(5'-phenylpyridin-2'-yl)quinoline.

2.3 6-Substituted Pyridylquinoline Ligand Derivatives

6-Bromo-2-(pyridin-2'-yl)quinoline was synthesised in two steps using two different methods (Schemes S5 and S6).

Method A: A mixture of 2-amino-5-bromobenzaldehyde and 2-amino-5-bromobenzyl alcohol was obtained from the reduction of 2-nitro-5-bromobenzaldehyde.[7] The 2-amino-5-bromobenzaldehyde was isolated from this mixture and used in a subsequent Friedländer reaction with 2-acetylpyridine (Scheme S5).

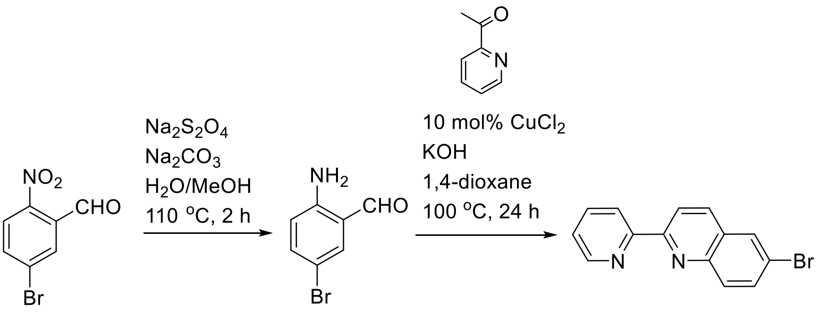

Scheme S5. Synthesis of 6-bromo-2-(pyridin-2'-yl)quinoline using method A.

Method B: The mixture of 2-amino-5-bromobenzaldehyde and 2-amino-5-bromobenzyl alcohol obtained from the reduction of 2-nitro-5-bromobenzaldehyde was used without further purification in the subsequent Friedländer reaction since the alcohol could be oxidised to the aldehyde *in situ* in the presence of copper(II) chloride (Scheme S6).

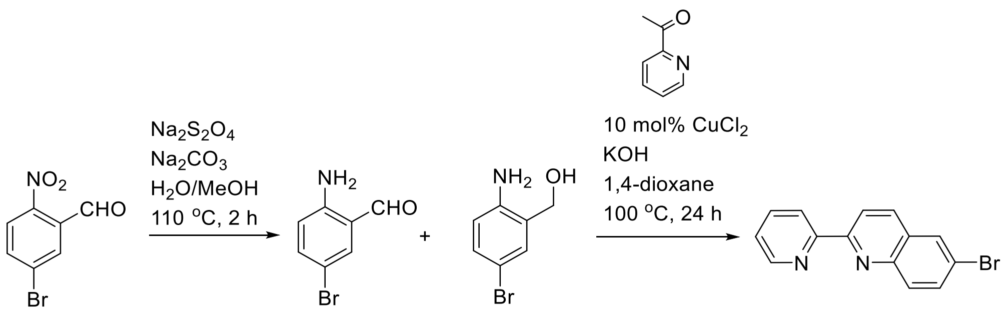

Scheme S6. Synthesis of 6-bromo-2-(pyridin-2'-yl)quinoline using method B.

The remaining ligands were prepared by palladium-catalysed Sonogashira or Suzuki coupling reactions (Schemes S7-S8).

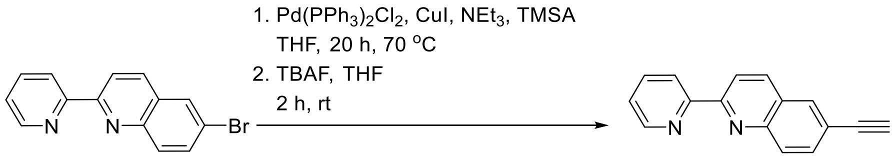

Scheme S7. Synthesis of 6-ethynyl-2-(pyridin-2'-yl)quinoline.

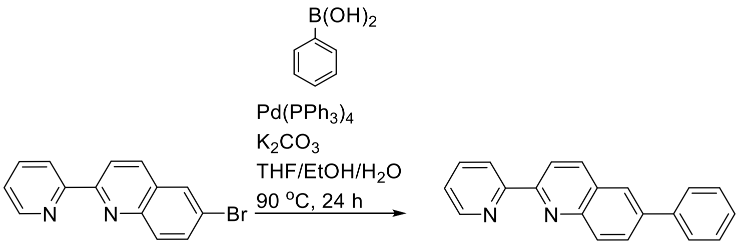

Scheme S8. Synthesis of 6-phenyl-2-(pyridin-2'-yl)quinoline.

2.3.1 6-Bromo-2-(pyridin-2'-yl)quinoline

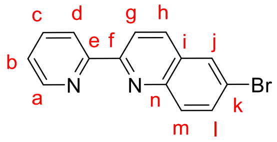

Method A: adapted from 7

A solution of $Na_2S_2O_4$ (11.8 g, 67.8 mmol) and K_2CO_3 (5.8 g, 54.7 mmol) in water (230 mL) was heated to 90 °C. 5-Bromo-2-nitrobenzaldehyde (2.80 g, 12.2 mmol) in methanol (50 mL) was heated to 50 °C for 5 min and added dropwise to the aqueous solution over 30 min. The reaction mixture was stirred for 2 h at 110 °C. After cooling down to room temperature the aqueous solution was placed in a freezer. The yellow crystals were separated from the solution and washed with water giving 2-amino-5-bromobenzaldehyde (190 mg, 0.95 mmol, 8%). The filtrate was extracted three times with diethyl ether (100 mL), the organic extracts were combined, dried over $MgSO_4$ and the solvent was removed *in vacuo* to give a mixture of 2-amino-5-bromobenzaldehyde and 2-amino-5-bromobenzyl alcohol. The mixture was recrystallised from *n*-pentane/dichloromethane and the solid was separated. The solvent of the filtrate was removed *in vacuo* to give 2-amino-5-bromobenzaldehyde (720 mg, 3.60 mmol, 30%).

2-Amino-5-bromobenzaldehyde (168 mg, 0.84 mmol), $CuCl_2$ (11.0 mg, 84.0 µmol, 10 mol%) and KOH (147 mg, 2.60 mmol) were added to a three-neck flask and dissolved in

1,4-dioxane (5 mL). 2-Acetylpyridine (113 µmL, 1.01 mmol) was added and the mixture was heated at 100 °C for 24 h. After cooling to room temperature, the mixture was filtered through Celite® and washed with EtOAc (10 mL). The organic layer was washed with sat. NH_4Cl solution (10 mL) and water (10 mL). The organic layer was separated and the aqueous layer was extracted twice with EtOAc (5 mL). The organic extracts were combined, dried over $MgSO_4$ and the solvent was removed *in vacuo*. The crude mixture was purified using column chromatography (silica gel, 5-10% EtOAc/cyclohexane) to give a yellow powder.

Yield: 182 mg (0.64 mmol, 77 %)

Method B:

A solution of $Na_2S_2O_4$ (11.8 g, 67.8 mmol) and K_2CO_3 (5.8 g, 54.7 mmol) in water (230 mL) was heated to 90 °C. 5-Bromo-2-nitrobenzaldehyde (2.80 g, 12.2 mmol) in methanol (50 mL) was heated to 50 °C for 5 min and added dropwise to the aqueous solution over 30 min. The reaction mixture was stirred for 2 h at 110 °C. After cooling down to room temperature the aqueous solution was extracted three times with diethyl ether (100 mL), the organic extracts were combined, dried over $MgSO_4$ and the solvent was removed *in vacuo* to give a mixture of 2-amino-5-bromobenzaldehyde and 2-amino-5-bromobenzyl alcohol, which was used in the second step without further purification. This mixture (1.33 g, 6.65 mmol, based on 2-amino-5-bromobenzaldehyde), $CuCl_2$ (89.4 mg, 665 µmol, 10 mol%) and KOH (1.18 g, 20.9 mmol) were added to a three-neck flask and dissolved in 1,4-dioxane (15 mL). 2-Acetylpyridine (0.89 mL, 7.89 mmol) was added and the mixture was heated at 100 °C for 24 h. After cooling to room temperature, the mixture was filtered through Celite® and washed with EtOAc (100 mL). The organic layer was washed with sat. NH_4Cl solution (50 mL) and water (50 mL). The organic layer was separated and the aqueous layer was extracted twice with EtOAc (50 mL). The organic extracts were combined, dried over $MgSO_4$ and the solvent was removed *in vacuo*. The crude was purified using column chromatography (silica gel, 5-10% EtOAc/cyclohexane) to give a yellow powder.

Yield: 1.17 g (4.10 mmol, 33% over two steps from 5-bromo-2-nitrobenzaldehyde)

R_f (cyclohexane/EtOAc 4:1): 0.36

The analytical data was consistent with literature data.[7]

^{1}H NMR (500 MHz, $CDCl_3$, 298 K) δ (ppm): 8.74 (ddd, ^3J = 4.8 Hz, ^4J = 1.7 Hz, ^5J = 1.0 Hz, 1H, H_a), 8.64 (dt, ^3J = 8.0 Hz, ^4J = 1.0 Hz, ^5J = 1.0 Hz, 1H, H_d), 8.60 (d, ^3J = 8.7 Hz, 1H, H_g), 8.19 (d, ^3J = 8.7 Hz, 1H, H_h), 8.04 (d, ^3J = 9.0 Hz, 1H, H_m), 8.01 (d, ^4J = 2.2 Hz, 1H, H_j), 7.88 (ddd, ^3J = 8.0 Hz, ^3J = 7.5 Hz, ^4J = 1.7 Hz, 1H, H_c), 7.79 (dd, ^3J = 9.0 Hz, ^4J = 2.2 Hz, 1H, H_l), 7.38 (ddd, ^3J = 7.5 Hz, ^3J = 4.8 Hz, ^4J = 1.0 Hz, 1H, H_b).

^{13}C NMR (125 MHz, $CDCl_3$, 298 K) δ (ppm): 156.4 (C_f), 155.8 (C_e), 149.2 (C_a), 146.5 (C_n), 137.1 (C_c), 135.8 (C_h), 133.1 (C_l), 131.5 (C_m), 129.7 (C_j), 129.3 (C_i), 124.3 (C_b), 121.9 (C_d), 120.7 (C_k), 119.8 (C_g).

HRMS (EI, 70 eV) *m/z*: 283.99530 $[M]^+$ (calculated: 283.99491 for $C_{14}H_9{}^{79}BrN_2$), 285.99326 $[M]^+$ (calculated: 285.99286 for $C_{14}H_9{}^{81}Br_1N_2$).

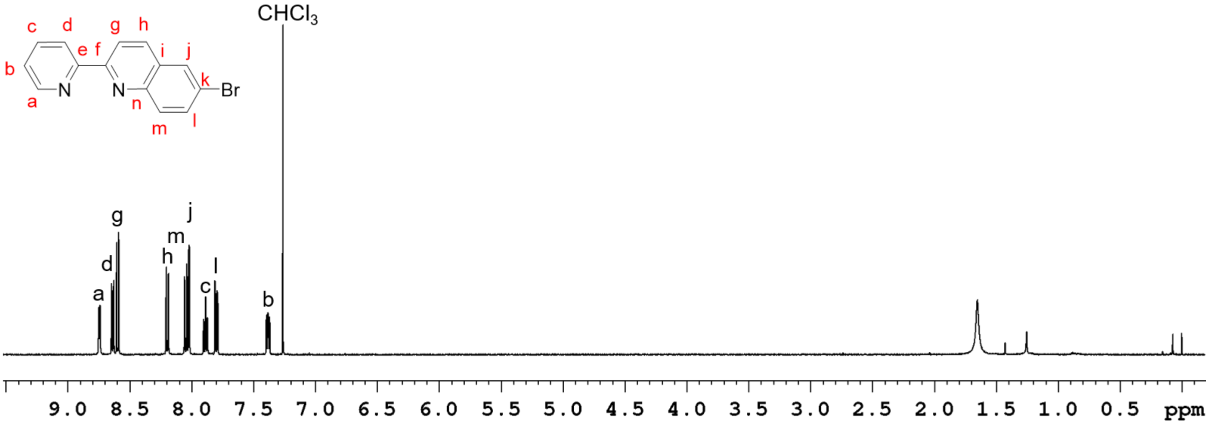

Figure S20. ^{1}H NMR spectrum (500 MHz, $CDCl_3$, 298 K) of 6-bromo-2-(pyridin-2'-yl)quinoline.

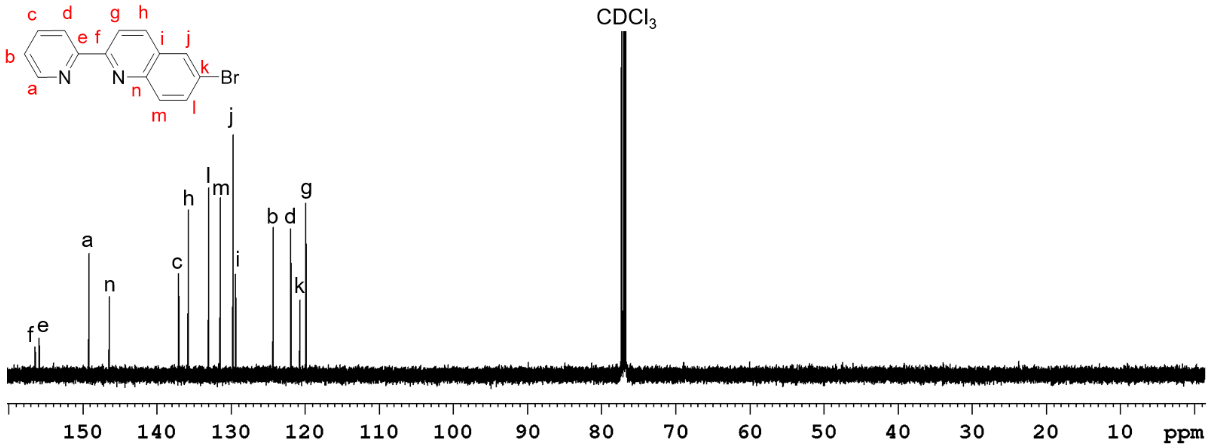

Figure S21. ^{13}C NMR spectrum (125 MHz, $CDCl_3$, 298 K) of 6-bromo-2-(pyridin-2'-yl)quinoline.

2.3.2 6-Ethynyl-2-(pyridin-2'-yl)quinoline

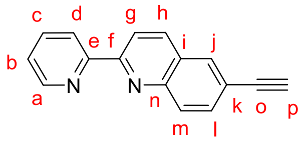

6-Bromo-2-(pyridin-2'-yl)quinoline (754 mg, 2.64 mmol), $Pd(PPh_3)_2Cl_2$ (92.6 mg, 132 µmol, 5 mol%) and CuI (50.3 mg, 264 µmol, 10 mol%) were added to a three-neck flask and dissolved in THF (20 mL). TMSA (0.45 mL, 3.17 mmol) was added to the solution, which was stirred at 70 °C for 20 h. After the solution was cooled to room temperature, TBAF (3.2 mL, 1 M in THF) was added and the solution was stirred for another 2 h. Dichloromethane (50 mL) was added and the mixture was washed with brine (50 mL) and water (50 mL). The organic phase was separated and the aqueous phase was extracted twice with dichloromethane (30 mL). The combined organic extracts were dried over $MgSO_4$ and the solvent was removed *in vacuo*. The crude was purified by column chromatography (silica gel, 10-20% EtOAc/cyclohexane) to give a yellow powder.

Yield: 411 mg (1.96 mmol, 74%)

R_f (cyclohexane/EtOAc 4:1): 0.35

^{1}H NMR (600 MHz, $CDCl_3$, 298 K) δ (ppm): 8.75 (d, ^3J = 4.6 Hz, 1H, H_a), 8.65 (d, ^3J = 7.8 Hz, 1H, H_d), 8.59 (d, ^3J = 8.6 Hz, 1H, H_g), 8.23 (d, ^3J = 8.6 Hz, 1H, H_h), 8.12 (d, ^3J = 8.6 Hz, 1H, H_m), 8.02 (d, ^4J = 1.5 Hz, 1H, H_j), 7.88 (td, ^3J = 7.8 Hz, ^4J = 1.7 Hz, 1H, H_c), 7.77 (dd, ^3J = 8.6 Hz, ^4J = 1.5 Hz, 1H, H_l), 7.38 (ddd, ^3J = 7.8 Hz, ^3J = 4.6 Hz, ^4J = 0.7 Hz, 1H, H_b), 3.21 (s, 1H, H_p).

^{13}C NMR (151 MHz, $CDCl_3$, 298 K) δ (ppm): 156.8 (C_f), 155.9 (C_e), 149.2 (C_a), 147.5 (C_n), 137.1 (C_c), 136.6 (C_h), 132.4 (C_l), 131.9 (C_j), 129.9 (C_m), 127.8 (C_i), 124.3 (C_b), 122.0 (C_d), 120.7 (C_k), 119.7 (C_g), 83.4 (C_o), 78.5 (C_p).

HRMS (EI, 70 eV) *m/z*: 230.08432 $[M]^+$ (calculated: 230.08440 for $C_{16}H_{10}N_2$).

M. p.: 139 °C

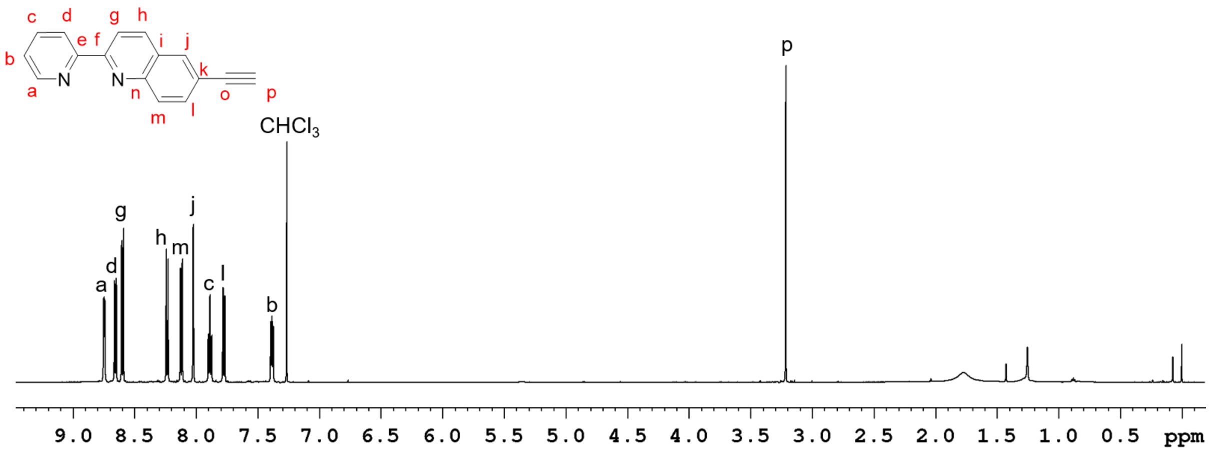

Figure S22. ^{1}H NMR spectrum (600 MHz, $CDCl_3$, 298 K) of 6-ethynyl-2-(pyridine-2'-yl)quinoline.

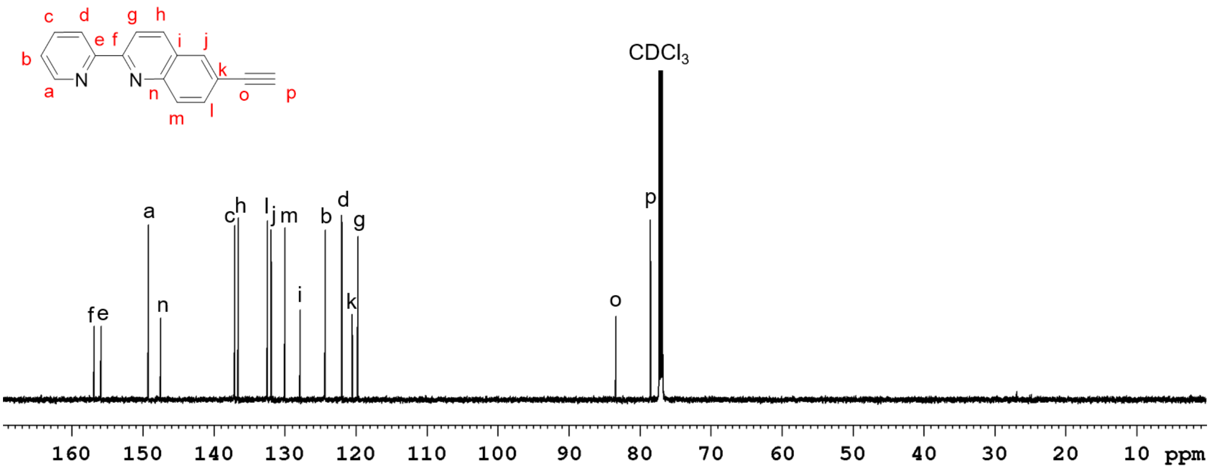

Figure S23. ^{13}C NMR spectrum (151 MHz, $CDCl_3$, 298 K) of 6-ethynyl-2-(pyridine-2'-yl)quinoline.

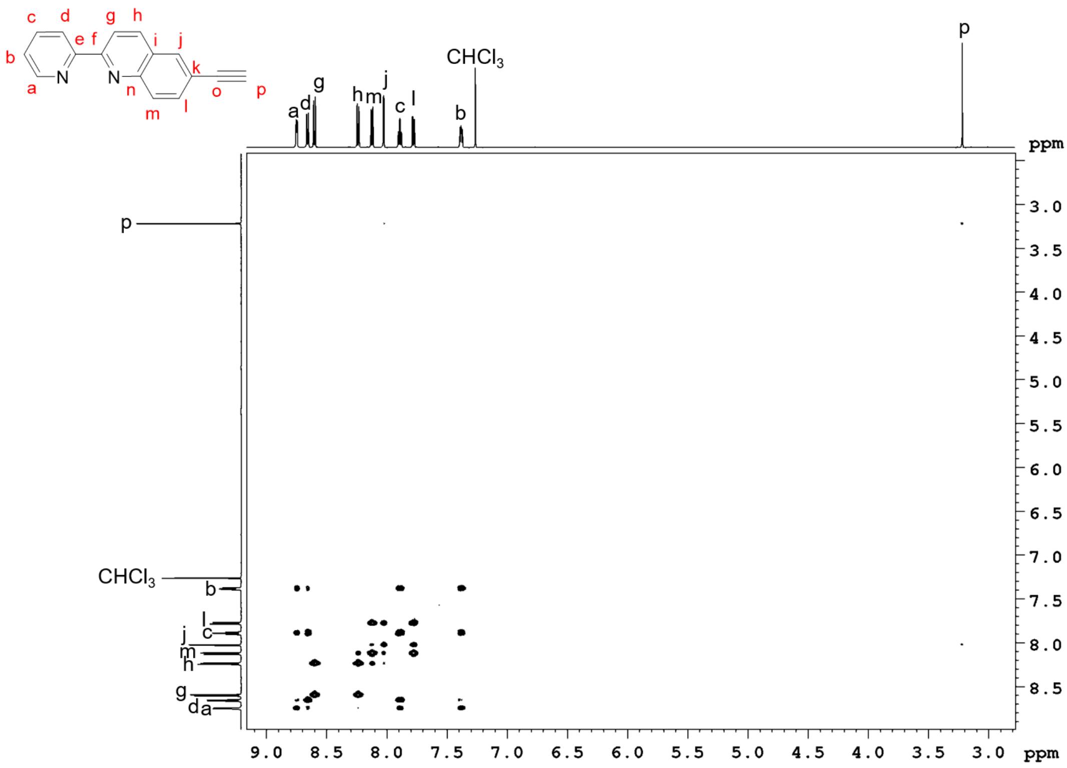

Figure S24. ^{1}H-^{1}H COSY NMR spectrum (600 MHz, $CDCl_3$, 298 K) of 6-ethynyl-2-(pyridine-2’-yl)quinoline.

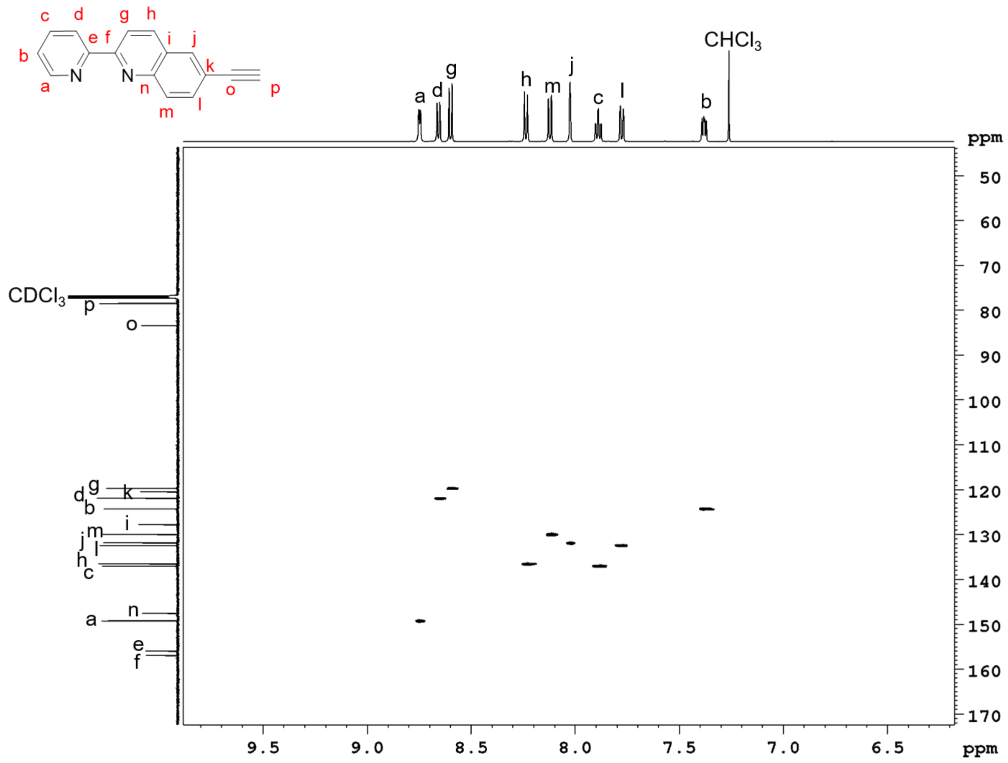

Figure S25. ^{1}H-^{13}C HSQC NMR spectrum (600 MHz/151 MHz, $CDCl_3$, 298 K) of 6-ethynyl-2-(pyridin-2’-yl)quinoline.

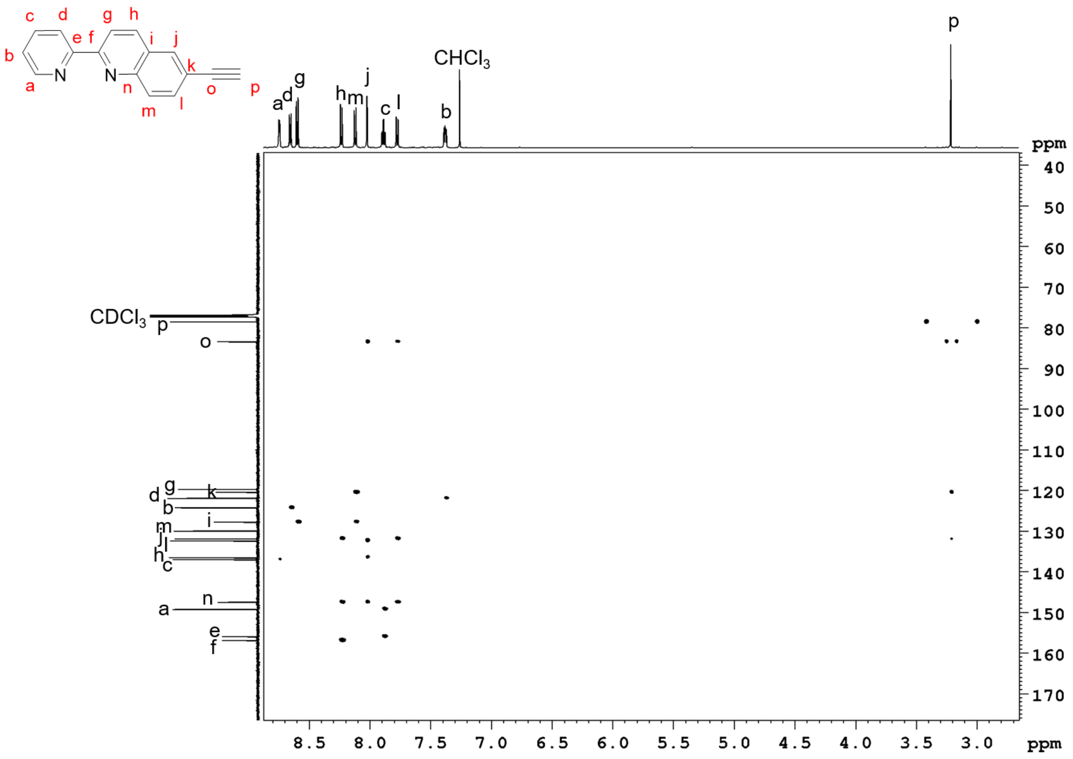

Figure S26. ^{1}H-^{13}C HMBC NMR spectrum (600 MHz/151 MHz, $CDCl_3$, 298 K) of 6-ethynyl-2-(pyridin-2'-yl)quinoline.

2.3.3 6-Phenyl-2-(pyridin-2'-yl)quinoline

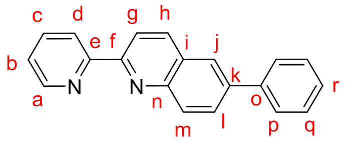

6-Bromo-2-(pyridin-2'-yl)quinoline (100 mg, 352 µmol), phenylboronic acid (42.7 mg, 352 µmol) and K_2CO_3 (122 mg, 880 µmol) were added to a three-neck flask and dissolved in a mixture of THF (8 mL), water (4 mL) and ethanol (6.5 mL). The mixture was degassed by three freeze-pump-thaw cycles. $Pd(PPh_3)_4$ (20.2 mg, 17.5 µmol, 5 mol%) was added and the solution was stirred at 90 °C for 24 h under a nitrogen atmosphere. After cooling to room temperature dichloromethane (30 mL) was added and the mixture was washed with water (30 mL), half-concentrated K_2CO_3 solution (15 mL sat. K_2CO_3 solution in 15 mL H_2O) and water (30 mL). The organic phase was separated and the aqueous phase was extracted twice with dichloromethane (40 mL). The combined organic extracts were dried over $MgSO_4$ and the solvent was removed *in vacuo*. The crude product was purified using column chromatography (silica gel, 6-10% EtOAc/cyclohexane) to give a colourless powder.

Yield: 64 mg (0.227 mmol, 64%)

R_f (cyclohexane/EtOAc 4:1): 0.16

^{1}H NMR (500 MHz, $CDCl_3$, 298 K) δ (ppm): 8.75 (ddd, ^{3}J = 4.8 Hz, ^{4}J = 1.7 Hz, ^{5}J = 0.9 Hz, 1H, H_a), 8.68 (d, ^{3}J = 7.7 Hz, 1H, H_d), 8.59 (d, ^{3}J = 8.6 Hz, 1H, H_g), 8.33 (d, ^{3}J = 8.6 Hz, 1H, H_h), 8.25 (d, ^{3}J = 8.7 Hz, 1H, H_m), 8.04 (d, ^{4}J = 2.0 Hz, 1H, H_j), 8.01 (dd, ^{3}J = 8.7 Hz, ^{4}J = 2.0 Hz, 1H, H_l), 7.89 (td, ^{3}J = 7.7 Hz, ^{4}J = 1.7 Hz, 1H, H_c), 7.76 – 7.73 (m, 2H, H_p),

7.53 – 7.49 (m, 2H, H_q), 7.43 – 7.39 (m, 1H, H_r), 7.37 (ddd, ^3J = 7.7 Hz, ^3J = 4.8 Hz, ^4J = 0.9 Hz, 1H, H_b).

^{13}C NMR (125 MHz, $CDCl_3$, 298 K) δ (ppm): 156.2 (C_e), 156.1 (C_f), 149.2 (C_a), 147.3 (C_n), 140.4 (C_o), 139.5 (C_k), 137.0 (C_h, C_c), 130.2 (C_m), 129.3 (C_l), 129.0 (C_q), 128.4 (C_i), 127.8 (C_r), 127.4 (C_p), 125.3 (C_j), 124.1 (C_b), 121.9 (C_d), 119.4 (C_g).

HRMS (EI, 70 eV) *m/z*: 282.11566 [M]$^+$ (calculated: 282.11570 for $C_{20}H_{14}N_2$).

M. p.: 136 °C

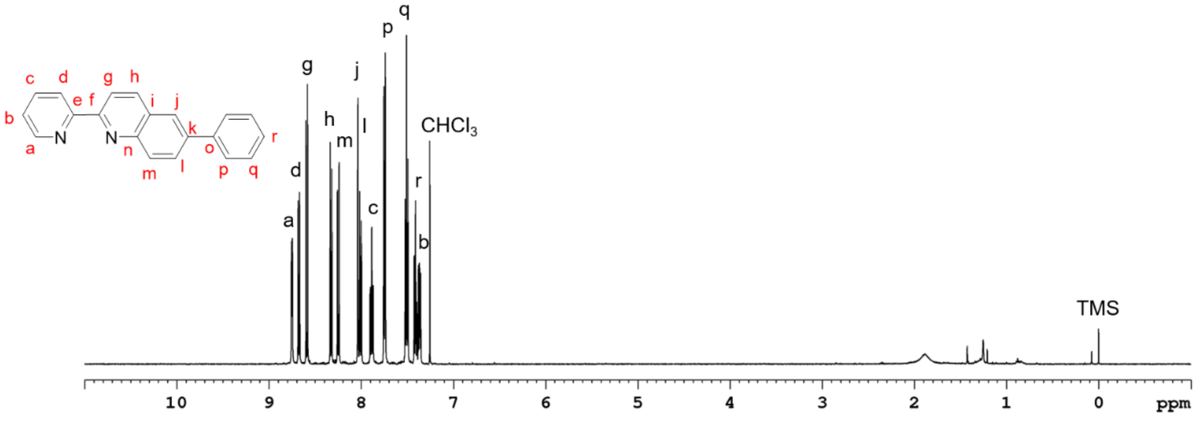

Figure S27. ^{1}H NMR spectrum (500 MHz, $CDCl_3$, 298 K) of 6-phenyl-2-(pyridin-2’-yl)quinoline.

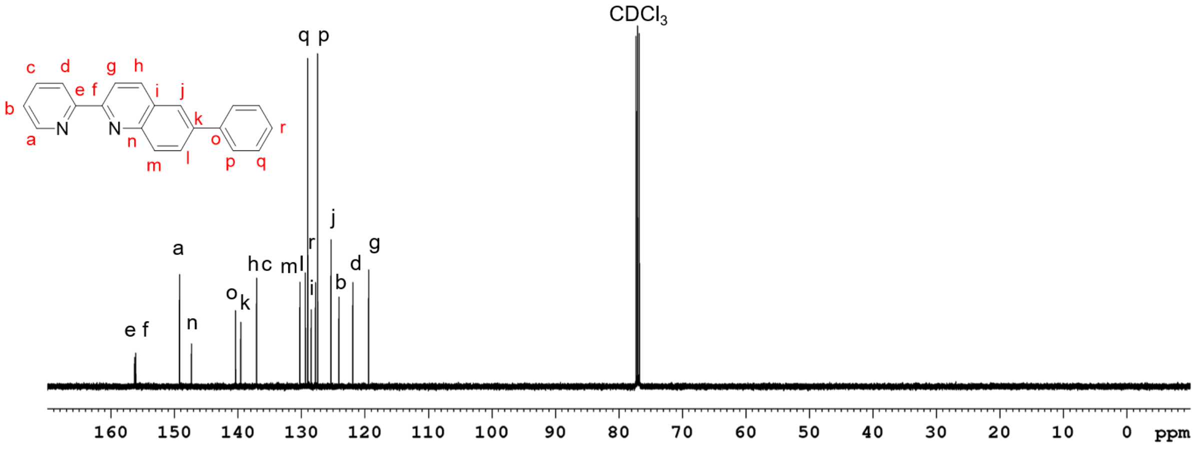

Figure S28. ^{13}C NMR spectrum (125 MHz, $CDCl_3$, 298 K) of 6-phenyl-2-(pyridine-2’-yl)quinoline.

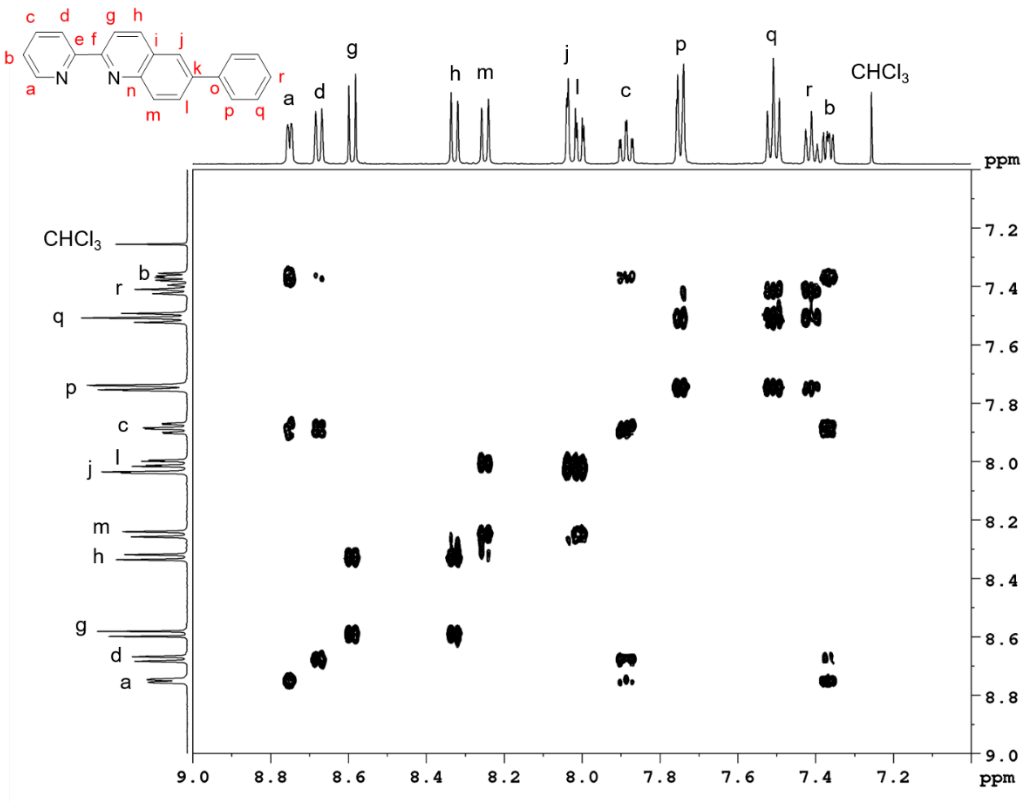

Figure S29. ^{1}H-^{1}H COSY NMR spectrum (500 MHz, $CDCl_3$, 298 K) of 6-phenyl-2-(pyridine-2'-yl)quinoline.

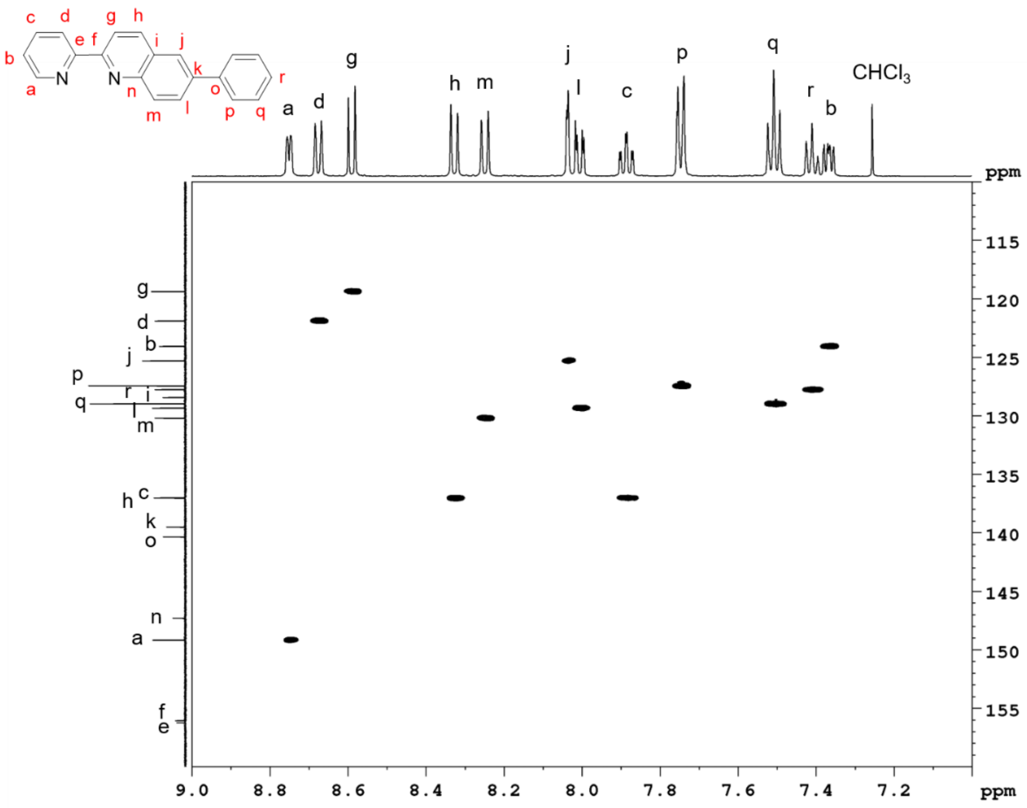

Figure S30. ^{1}H-^{13}C HSQC NMR spectrum (500 MHz/125 MHz, $CDCl_3$, 298 K) of 6-phenyl-2-(pyridin-2'-yl)quinoline.

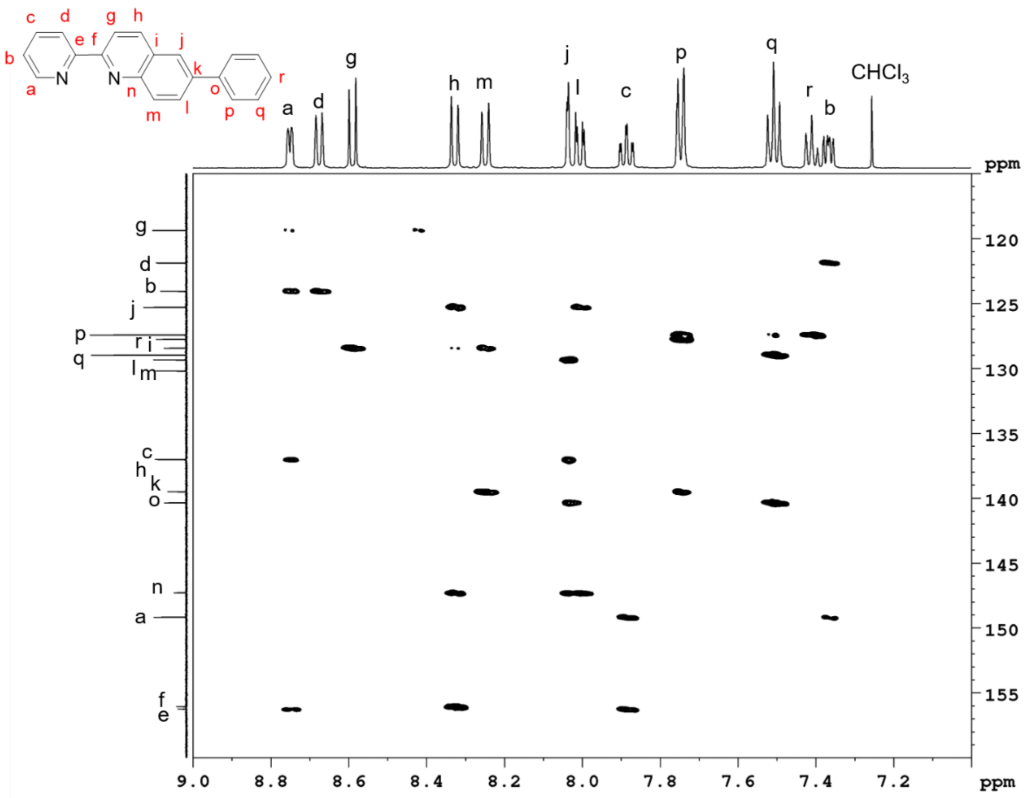

Figure S31. ^{1}H-^{13}C HMBC NMR spectrum (500 MHz/125 MHz, $CDCl_3$, 298 K) of 6-phenyl-2-(pyridin-2’-yl)quinoline.

2.4 Ligand for Cage 8

The ligand was prepared according to Scheme S9; 5-bromo-2,2’-bipyridine was prepared according to a literature procedure.[8] 5-Ethynyl-2,2’-bipyridine was prepared using an adapted literature procedure[9] where the TMS-protected alkyne was synthesised and then deprotected *in situ* using TBAF. The target ligand was prepared from 5-bromo-2,2’-bipyridine and 5-ethynyl-2,2'-bipyridine using a literature procedure where benzene was substituted for THF as the solvent.[10]

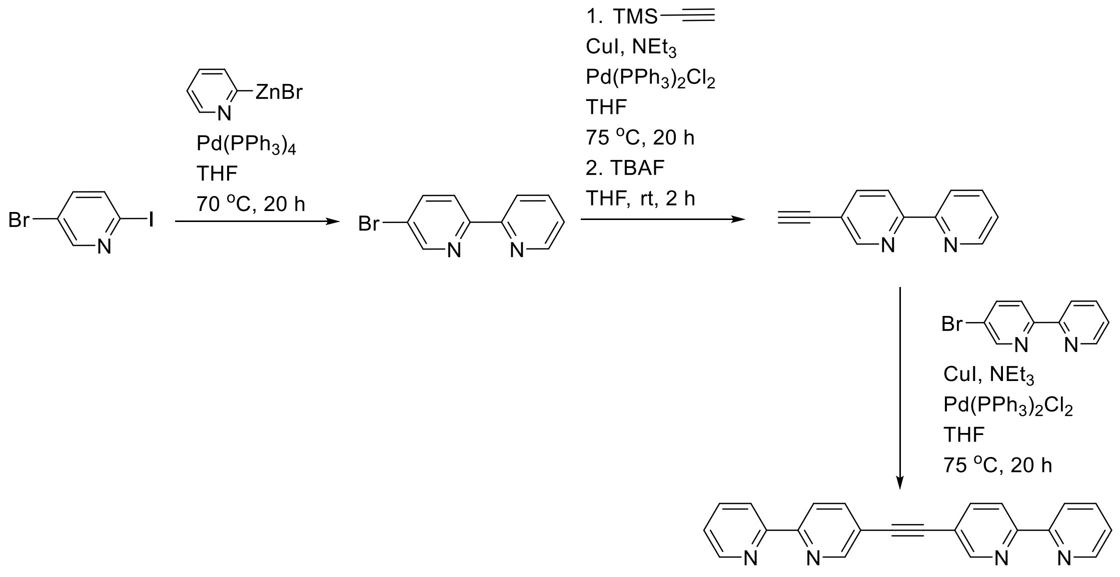

Scheme S9. Synthesis of the ligand for cage **8**.

2.4.1 5-Ethynyl-2,2'-bipyridine

5-Bromo-2,2'-bipyridine (300 mg, 1.28 mmol), CuI (24.4 mg, 128 µmol, 10 mol%) and $Pd(PPh_3)_2Cl_2$ (44.9 mg, 64 µmol, 5 mol%) were added to a three-neck flask. Under a nitrogen atmosphere anhydrous THF (20 mL), TMSA (0.2 mL, 1.40 mmol) and NEt_3 (2.0 mL) were added. The reaction mixture was heated under reflux overnight. After the solution was cooled to room temperature, TBAF (1.4 mL, 1 M in THF) was added and the solution was stirred for another 2 h. Dichloromethane (20 mL) was added and the mixture was washed with water (50 mL). The organic phase was separated and the aqueous phase was extracted twice with dichloromethane (20 mL). The combined organic extracts were dried over $MgSO_4$ and the solvent was removed *in vacuo*. The crude was purified by flash column chromatography (silica gel, 5-40% EtOAc/cyclohexane) to give a beige crystalline solid.

Yield: 145 mg (0.81 mmol, 63%)

R_f (cyclohexane/EtOAc 4:1): 0.33

The analytical data was consistent with literature data.[9]

3 Mononuclear Complexes

3.1 Mononuclear Cobalt Complexes

3.1.1 *mer*-$[Co(pq)_3](BF_4)_2$ (**1a**)

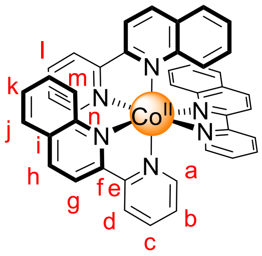

In situ preparation:

$Co(BF_4)_2 \bullet 6H_2O$ (17.4 mg, 51.1 µmol) and 2-(2-pyridyl)quinoline (31.6 mg, 153 µmol) were dissolved in dry CD_3CN (0.5 mL) in an NMR tube and the solution was heated to 50 °C for 12 h to reach thermal equilibrium.

Crystals:

A solution of $Co(BF_4)_2 \bullet 6H_2O$ (50 mg, 0.15 mmol) in ethanol (1 mL) was added to a solution of 2-(2-pyridyl)quinoline (130 mg, 0.63 mmol) in ethanol (1 mL). The reaction mixture was heated up to 50 °C for 30 min. The crystals were isolated and washed three times with diethyl ether before they were redissolved in CD_3CN for NMR analysis.

^{1}H NMR (600 MHz, CD_3CN, 298 K) δ (ppm): 97.1 (s, 1H, H_{d1}), 92.0 (s, 1H, H_{d2}), 87.5 (s, 1H, H_{g1}), 83.3 (s, 1H, H_{g2}), 82.4 (s, 1H, H_{d3}), 71.9 (bs, H_a), 67.9 (s, 1H, H_{b3}), 58.0 (s, 1H, H_{g3}), 57.3 (bs, H_a), 48.0 (s, 1H, H_{b1}), 40.4 (s, 1H, H_{b2}), 36.4 (s, 1H, H_{j1}), 27.3 (s, 1H, H_{j2}), 25.8 (s, 1H, H_{j3}), 21.5 (s, 1H, H_{h2}), 19.0 (s, 1H, H_{h1}), 15.6 (s, 1H, H_{k1}), 14.9 (s, 1H, H_{h3}), 14.7 (s, 1H, H_{l1}), 12.4 (s, 1H, H_{c3}), 11.7 (s, 1H, H_{c1}), 10.5 (s, 1H, H_{c2}), 7.1 (bs, H_a), -1.2 (s, 1H, H_{k2}), -7.7 (s, 1H, H_{k3}), -13.2 (s, 1H, H_{l2}), -16.2 (s, 1H, H_{l3}), -23.1 (bs, H_m), -142.8 (bs, H_m), -166.5 (bs , H_m).

^{13}C NMR (151 MHz, CD_3CN, 298 K) δ (ppm): 617.9 (d, ^1J = 180 Hz, C_{b2}), 585.1 (s, $C_{i/n}$), 544.2 (d, ^1J = 168 Hz, C_{b3}), 541.5 (d, ^1J = 169 Hz, C_{b1}), 499.1 (s, $C_{i/n}$), 483.5 (s, $C_{i/n}$), 458.1 (d, ^1J = 176 Hz, C_{g2}), 415.1 (d, ^1J = 158 Hz, C_{d2}), 368.1 (d, ^1J = 163 Hz, C_{g1}), 355.7 (d, ^1J = 162 Hz, C_{d1}), 352.1 (d, ^1J = 153 Hz, C_{d3}), 305.5 (s, $C_{i/n}$), 288.2 (d, ^1J = 173 Hz, C_{g3}),

272.3 (s, $C_{i/n}$), 237.5 (d, ^1J = 166 Hz, C_{c1}), 232.6 (s, $C_{i/n}$), 226.7 (d, ^1J = 165 Hz, C_{j1}), 217.9 (b, C_m), 216.7 (d, ^1J = 170 Hz, C_{c3}), 209.8 (d, ^1J = 163 Hz, C_{j2}), 194.3 (d, ^1J = 165 Hz, C_{c2}), 191.2 (d, ^1J = 158 Hz, C_{j3}), 185.4 (d, ^1J = 158 Hz, C_{h3}), 177.4 (d, ^1J = 155 Hz, C_{h1}), 174.6 (b, C_m), 161.6 (b, C_m), 143.6 (d, ^1J = 158 Hz, C_{k1}), 126.5 (d, ^1J = 178 Hz, C_{k3}), 122.5 (d, ^1J = 160 Hz, C_{l1}), 122.1 (d, ^1J = 166 Hz, C_{k2}), 115.0 (d, ^1J = 160 Hz, C_{h2}), 78.4 (d, ^1J = 166 Hz, C_{l2}), 49.6 (d, ^1J = 153 Hz, C_{l3}), 48.3 (b, C_a), 42.2 (b, C_a), 36.2 (b, C_a), -23.4 (s, $C_{e/f}$), -33.4 (s, $C_{e/f}$), -44.0 (s, $C_{e/f}$), -84.7 (s, $C_{e/f}$), -118.4 (s, $C_{e/f}$), -225.2 (s, $C_{e/f}$).

HRMS (ESI) *m/z*: 558.1032 $[Co(pq)_2 + BF_4]^+$, 338.5921 (calculated for $C_{42}H_{30}N_6Co$: 338.5926) $[Co(pq)_3]^{2+}$, 235.5499 $[Co(pq)_2]^{2+}$.

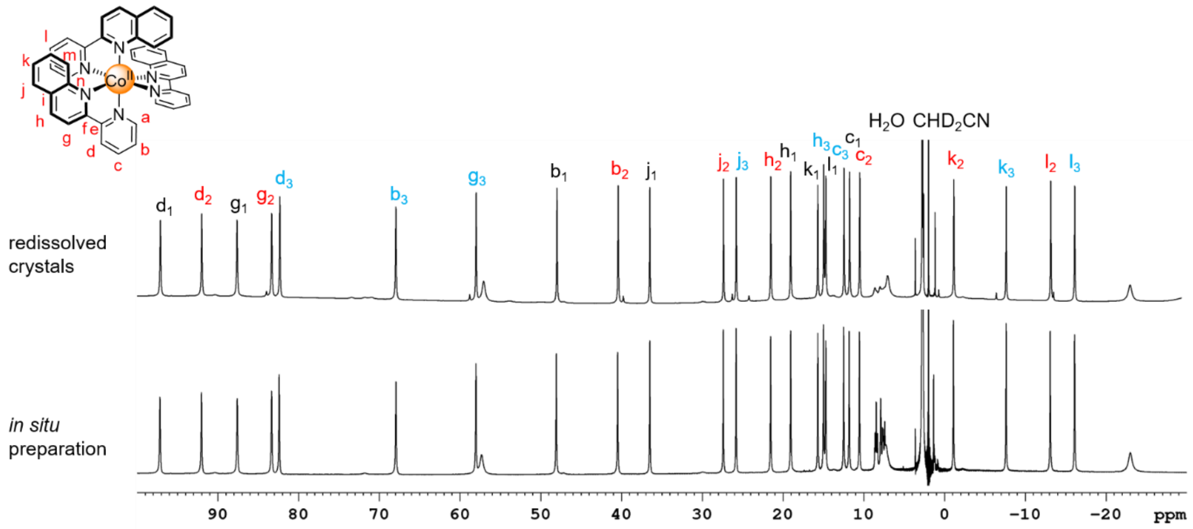

Figure S32. Comparison of ^1H NMR spectrum (600 MHz, CD_3CN, 298 K) of *mer*-$[Co(pq)_3](BF_4)_2$ prepared *in situ* in CD_3CN and by redissolving crystals.

3.1.1.1 X-ray crystal structure determination of $[Co(pq)_3](BF_4)_2$ (**1a**)

Data collection was performed with an Imaging Plate Diffraction System (IPDS-2) from STOE & CIE, Darmstadt, Germany using $MoK\alpha_1$ radiation. Structure solution was performed with SHELXT[1] and structure refinement was done with SHELXL-2018.[2] A numerical absorption correction was performed using programs X-RED and X-SHAPE of the program package X-Area.[3] All non-hydrogen atoms were refined anisotropic. The C-H H atoms were positioned with idealised geometry and were refined isotropic with $U_{iso}(H) = 1.2\ U_{eq}(C)$ using a riding model. One of the two tetrafluoroborate anions is disordered and was refined using a split model with restraints for the bond lengths and angles (SAME) as well as for the anisotropic displacement parameters (SIMU and DELU). Even in this case large components of the displacement factors are observed indicating for a strong rotational disorder that cannot be resolved. This is similar for the second crystallographically independent anion but in this case refinement with a split model does not lead to a better structure model. Therefore, the highest residual electron density peaks are close to this anion. Selected crystal data and details of the structure refinements can be found in Table S2 and an Ortep plot with labelling and a table with selected bond lengths and angles are presented in Figure S33 and Table S3.

CCDC 1987819 contains the supplementary crystallographic data for this paper. These data can be obtained free of charge from the Cambridge Crystallographic Data Centre via http://www.ccdc.cam.ac.uk/data_request/cif.

Table S2. Selected crystal data and details of the single crystal structure refinements for $[Co(pq)_3](BF_4)_2$ (**1a**).

**compound**	**1**
formula	$C_{42}H_{30}B_2CoF_8N_6$
MW / g mol^{-1}	851.27
crystal system	orthorhombic
space group	*Pbca*
a / Å	21.3497(5)
b / Å	15.0262(3)
c / Å	24.9012(4)
a / °	90
β / °	90
γ / °	90
V / Å^3	7988.4(3) Å3
T / K	200(2)
Z	8
D_{calc} / g cm^{-3}	1.416
μ / mm^{-1}	0.506
θ_{max} / deg	27.004
measured refl.	69081
unique refl.	8700
refl. $F_0>4\sigma(F_0)$	7372
R_{int}	0.0521
parameter	568
R_1 [F_0 > 4σF_0)]	0.0673
wR_2 [all data]	0.1912
GOF	1.081
$\Delta\rho_{max/min}$ / e Å^{-3}	1.112/ -0.486

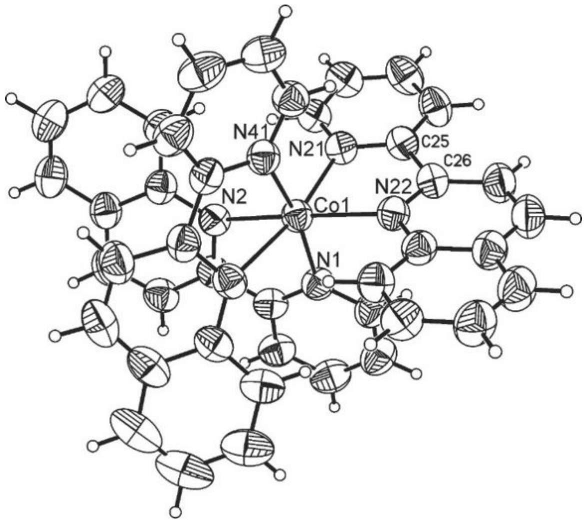

Figure S33. Crystal structure of $[Co(pq)_3](BF_4)_2$ (**1a**) with view of the Co coordination, labelling and displacement ellipsoids drawn at the 50% probability level. The BF_4^- anions are omitted for clarity.

Table S3. Selected bond lengths [Å] and angles [°] for $[Co(pq)_3](BF_4)_2$ (**1a**).

Co(1)-N(41)	2.089(2)	Co(1)-N(22)	2.174(2)
Co(1)-N(21)	2.101(2)	Co(1)-N(2)	2.222(2)
Co(1)-N(1)	2.110(2)	Co(1)-N(42)	2.301(2)
N(41)-Co(1)-N(21)	98.94(10)	N(1)-Co(1)-N(2)	76.20(9)
N(41)-Co(1)-N(1)	168.79(10)	N(22)-Co(1)-N(2)	167.83(9)
N(21)-Co(1)-N(1)	92.16(10)	N(41)-Co(1)-N(42)	74.10(9)
N(41)-Co(1)-N(22)	89.45(9)	N(21)-Co(1)-N(42)	169.30(10)
N(21)-Co(1)-N(22)	76.99(9)	N(1)-Co(1)-N(42)	94.69(10)
N(1)-Co(1)-N(22)	94.58(10)	N(22)-Co(1)-N(42)	110.56(9)
N(41)-Co(1)-N(2)	101.09(9)	N(2)-Co(1)-N(42)	78.53(9)
N(21)-Co(1)-N(2)	95.19(9)		

3.1.1.2 Proton Assignment through T_1 Measurements

Given the availability of the crystal structure for *mer*-$[Co(pq)_3](BF_4)_2$, assignment of the ^1H NMR spectrum was attempted using the Solomon equation, which states T_1 is inversely proportional to $\Sigma(r_{ij})^{-6}$ where r_{ij} is the distance from the paramagnetic center to the proton.[11] Since the T_1 values for all of the complex's signals could not be determined simultaneously in the 250 ppm range of the paramagnetic ^1H NMR spectrum and there were a large number of proton signals, the T_1 relaxation time for each signal (Table S4) was estimated by finding the null point in an inversion recovery experiment (t1ir1d pulse program) since $T_1 = T_{null}/\ln 2$. The three Co^{II}-proton distances in the crystal structure were used for calculating $\Sigma(r_{ij})^{-6}$ for each proton. These values were normalised to the measured T_1 for proton *k* since this was the longest T_1 and the proton assignment was unambiguous; it was not possible to calculate the relative T_1 values normalised to the smallest measured T_1 value since protons *d* and *g* had similar T_1 values and they could not be distinguished on the basis of Co^{II}-proton distances/T_1 values alone. Furthermore, the three different ligand environments for each proton could not be distinguished using this assignment approach. The proton assignments in Table S4 were made using the NMR spectra in Section 3.1.1.3 and these show good agreement with the normalised $[\Sigma(r_{ij})^{-6}]^{-1}/T_{1meas}$ values.

Table S4. T_1 relaxation measurements and proton assignments for **1a**.

Proton assignment	δ (ppm)	Estimated T_1 (ms)	Normalised $[\Sigma(r_{ij})^{-6}]^{-1}$[a]	Normalised $[\Sigma(r_{ij})^{-6}]^{-1}/T_{1meas}$
a	57.3	1.0	0.8	0.8
	[b]			
	[b]			
b	67.8	10.1	15.6	1.5
	47.9	16.7	15.6	0.9
	40.3	15.2	15.6	1.0
c	12.3	39.1	33.9	0.9
	11.7	39.1	33.9	0.9
	10.5	39.1	33.9	0.9
d	96.9	13.3	13.6	1.0
	91.8	15.2	13.6	0.9
	82.1	15.9	13.6	0.9
g	87.4	10.6	13.1	1.2
	83.1	11.6	13.1	1.1
	57.9	10.9	13.1	1.2
h	21.4	40.6	34.7	0.9
	19.0	40.6	34.7	0.9
	14.9	40.6	34.7	0.9
j	36.4	62.3	65.1	1.0
	27.3	62.3	65.1	1.0
	25.7	62.3	65.1	1.0
k	15.6	79.7	79.7	1.0
	-1.1	79.7	79.7	1.0
	-7.6	79.7	79.7	1.0
l	14.7	21.7	21.1	1.0
	-13.1	23.5	21.1	0.9
	-16.1	23.5	21.1	0.9
m	-22.9	0.9	0.8	0.9
	-1428	0.9	0.8	0.9
	-168.5	0.7	0.8	1.2

[a] Calculated from the X-ray crystal structure of $[Co(pq)_3]^{2+}$ by using the three Co^{II}-proton distances for each type of proton.[12] The relative T_1 value was calculated by normalising $[\Sigma(r_{ij})^{-6}]^{-1}$ to the measured T_1 value for the *k* signals. [b] The chemical shift or T_1 value could not determined as the signal was too broad.

3.1.1.3 Characterisation using Paramagnetic NMR Spectroscopy and Mass Spectrometry

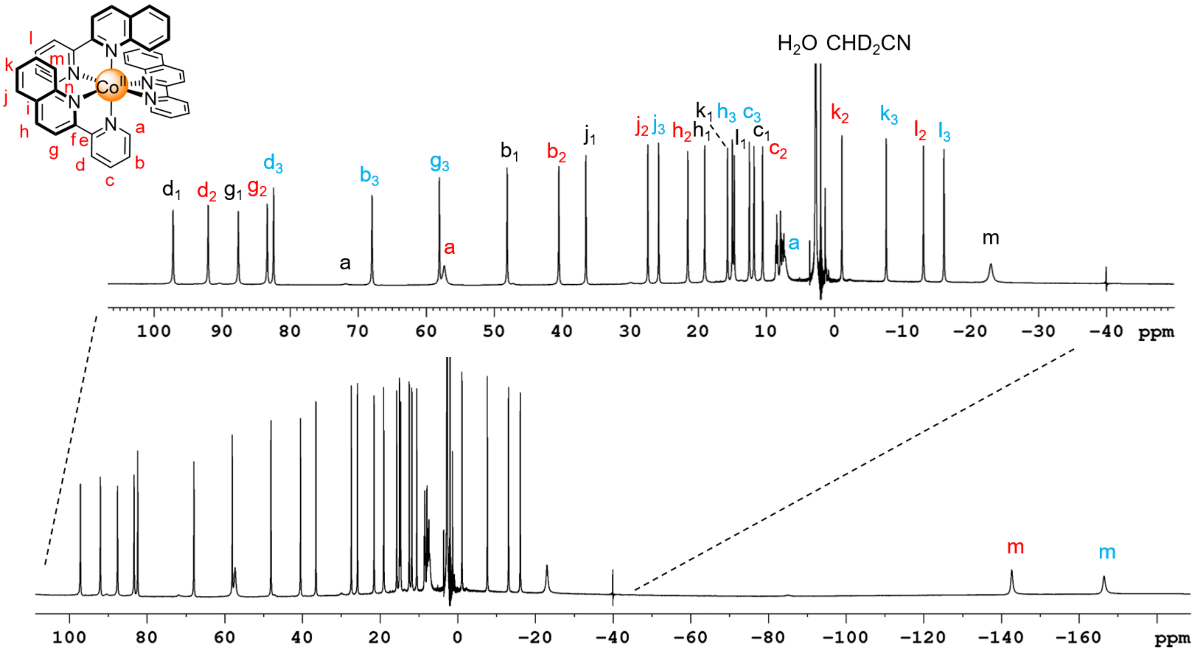

Figure S34. ^{1}H NMR spectrum (600 MHz, CD_3CN, 298 K) of $[Co(pq)_3](BF_4)_2$. Note: the absence of NOE cross-peaks between protons *h* and *j* in the COSY spectrum prevented assignment of these spin systems to a particular ligand environment and therefore, spin system *j*-*l* was arbitrarily labelled with black, red and blue labels in decreasing chemical shift order of proton *j* to represent the three ligand environments. The three proton *m* signals were labelled in a similar manner.

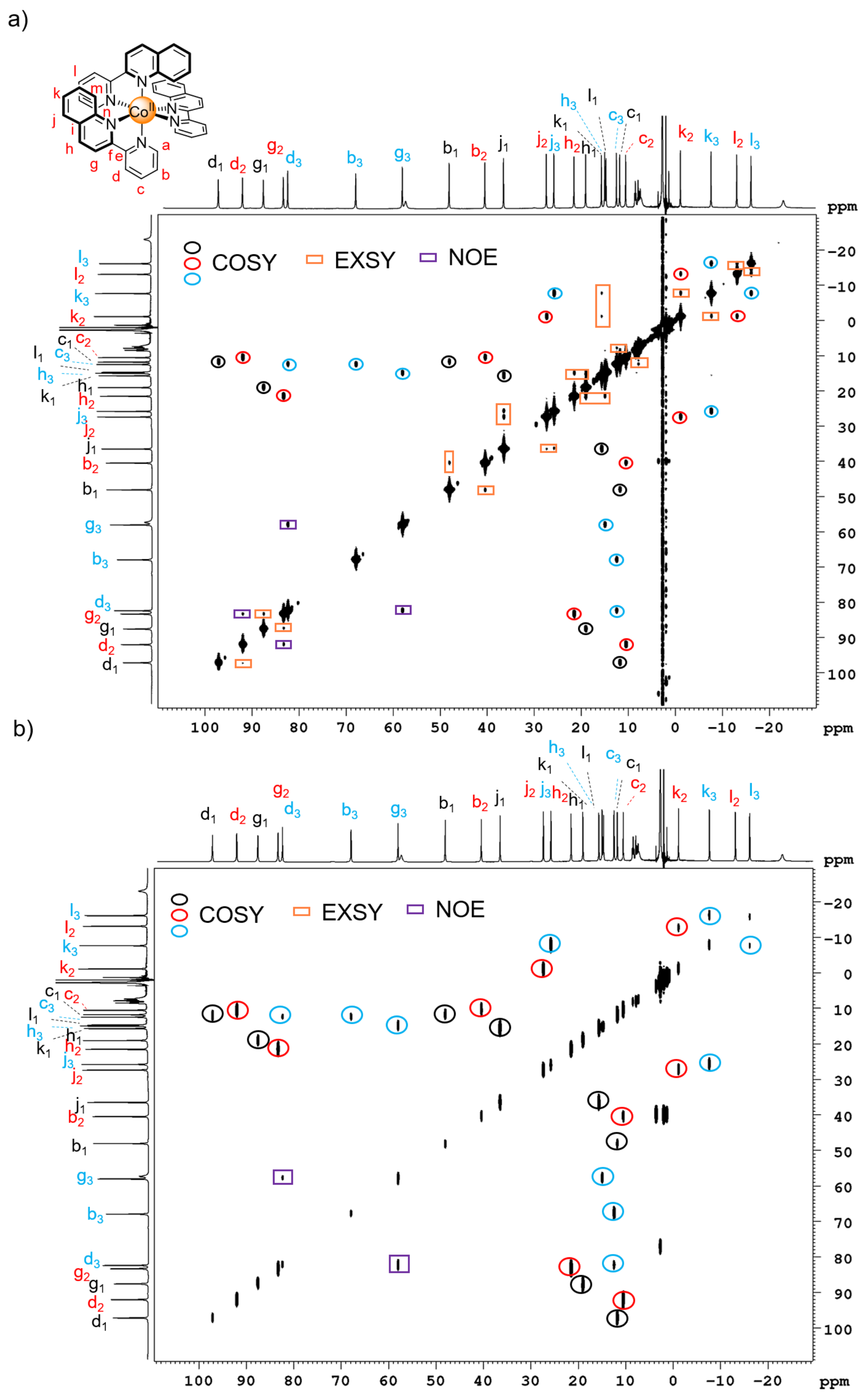

Figure S35a. Comparison of ^{1}H-^{1}H COSY NMR spectra (600 MHz, CD_3CN, 298 K) of $[Co(pq)_3](BF_4)_2$ using different pulse programs: a) cosygpqf; b) cosygpmfqf; c) cosyqf90.

c)

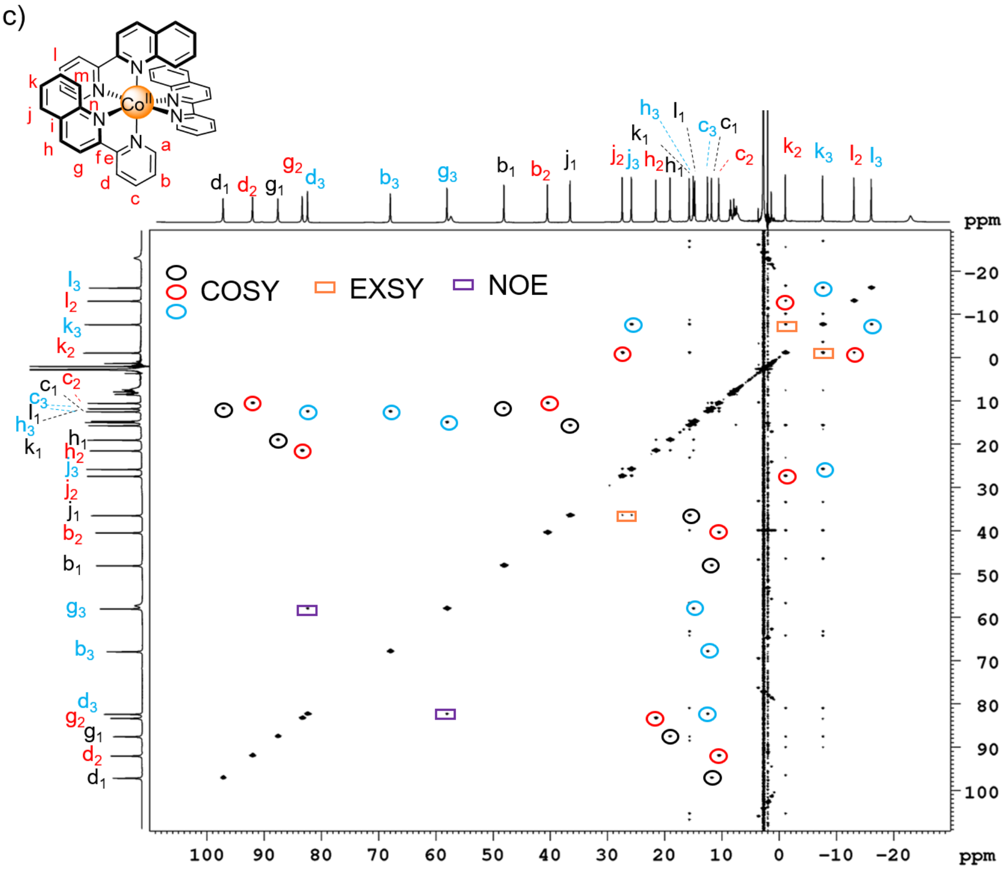

Figure S35b. Comparison of ^{1}H-^{1}H COSY NMR spectra (600 MHz, CD_3CN, 298 K) of $[Co(pq)_3](BF_4)_2$ using different pulse programs: a) cosygpqf; b) cosygpmfqf; c) cosyqf90. Through-space (COSY) cross-peaks within the three ligand environments are represented by the red, black and blue circles, respectively while exchange (EXSY) cross-peaks are represented by the orange squares. The cross-peaks in the purple squares are attributed to through-space (NOE) correlations.

Integration of exchange (EXSY) cross-peaks:

A series of ^{1}H-^{1}H NOESY NMR spectra (600 MHz, CD_3CN, 298 K) of $[Co(pq)_3](BF_4)_2$ was measured where the mixing time D8 was varied from 0.001 s up to 0.02 s. The resulting exchange cross-peaks for each set of protons in the three different ligand environments were integrated. The spectrum recorded with D8 = 0.001 s was used to normalise the integrals since it showed, as expected, no exchange cross-peaks. The exchange cross-peaks integrals in the spectra recorded with D8 = 0.002, 0.003, 0.004, 0.006, 0.008, 0.001, 0.012, 0.016 and 0.02 s were normalised by subtracting the respective integrals in the background spectrum with D8 = 0.001 s. The resulting exchange cross-peak integral for each proton in the different ligand environments was plotted against the acquisition time D8 as shown in Figure S36.

The initial rate of exchange between the three ligand environments was similar for each set of exchanging protons as there was a linear increase in the exchange integral for up to 4 ms. For protons with T_1 relaxation times significantly longer than the mixing time, the exchange integral approached a maximum as the mixing time increased. However, for protons with shorter T_1 relaxation times (e.g. protons *d* and *g*), the exchange integral reached a maximum

before decreasing as relaxation began competing with exchange when the mixing time increased. A mixing time of 10 ms was found to be a good compromise for maximising the exchange cross-peak of all protons despite their differing T_1 relaxation times.

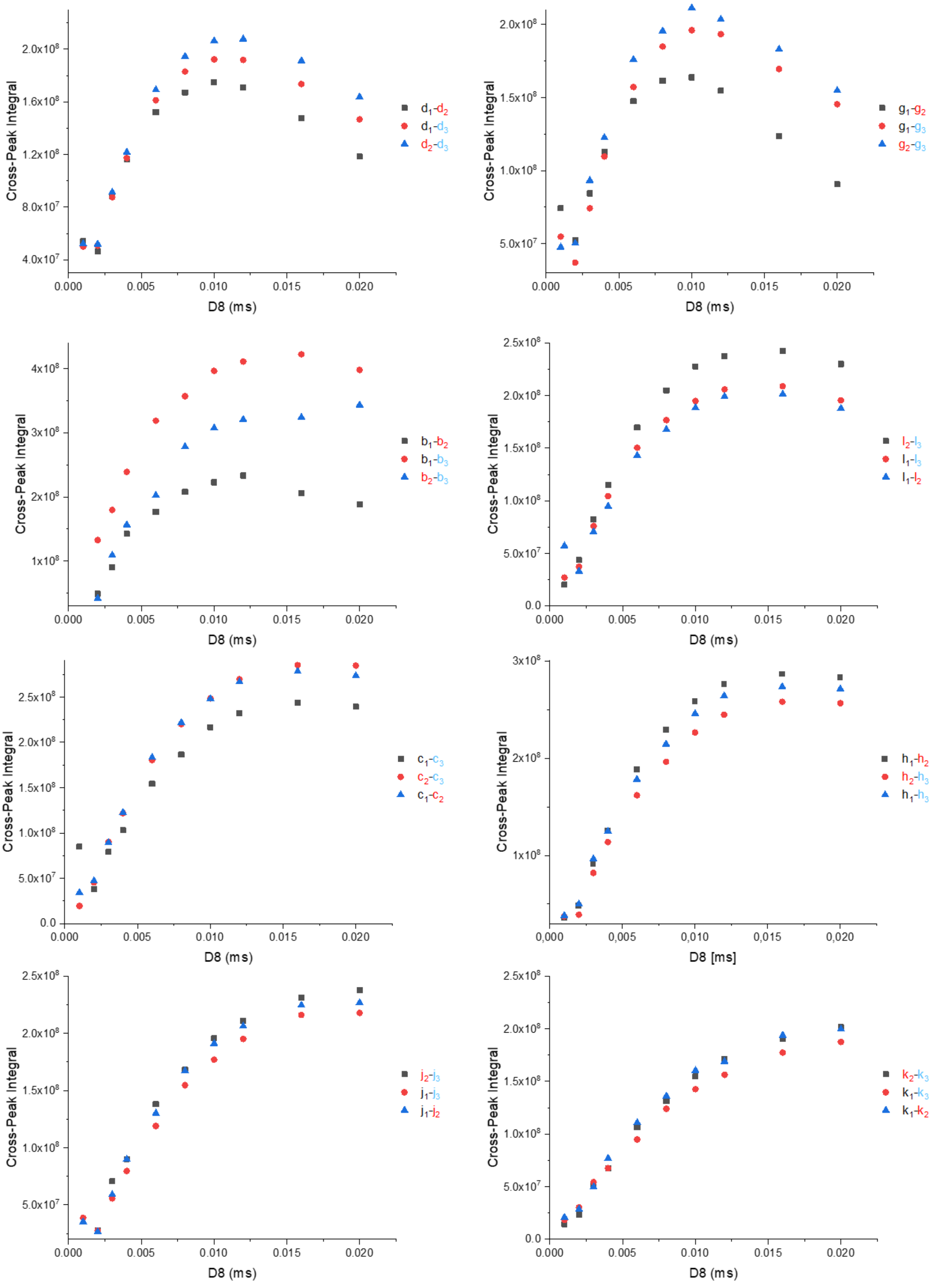

Figure S36. Comparison of integrated cross-peak versus mixing time for ^1H-^1H NOESY NMR spectrum (600 MHz, CD_3CN, 298 K) of $[Co(pq)_3](BF_4)_2$.

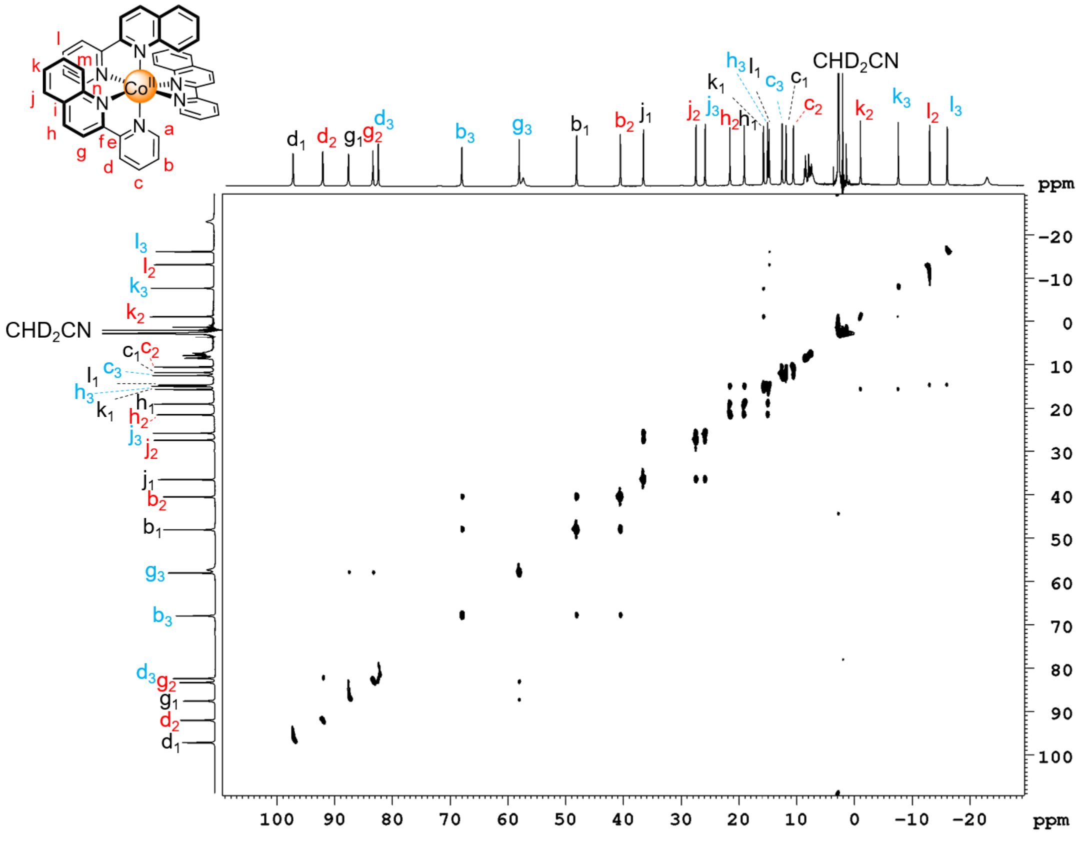

Figure S37. ^1H-^1H TOCSY NMR spectrum (600 MHz, CD_3CN, 298 K) of $[Co(pq)_3](BF_4)_2$.

Steady-state NOE experiments were carried out to investigate whether NOEs between protons in different spin systems (e.g. *d*/*g* and *h*/*j*) could be observed. In contrast to the single relaxation allowed NOE cross-peaks observed in the COSY spectrum (Figure S35), there were three NOE cross-peaks peaks in the steady-state NOE experiments due to competing exchange between the three ligand environments. Furthermore, the NOE cross-peak intensities were very small compared to the EXSY cross-peaks. While more intense cross-peaks consistent with NOEs were observed for irradiation of protons d_3 and g_3, these are likely the result of cross-excitation rather than NOEs due to the proximity of signals d_3 and g_3 (Figure S38). The effect of cross-excitation was more evident in the irradiation of h_3 as k_1, l_1 and c_3 were also excited and exchange cross-peaks were observed for these protons as well (Figure S39).

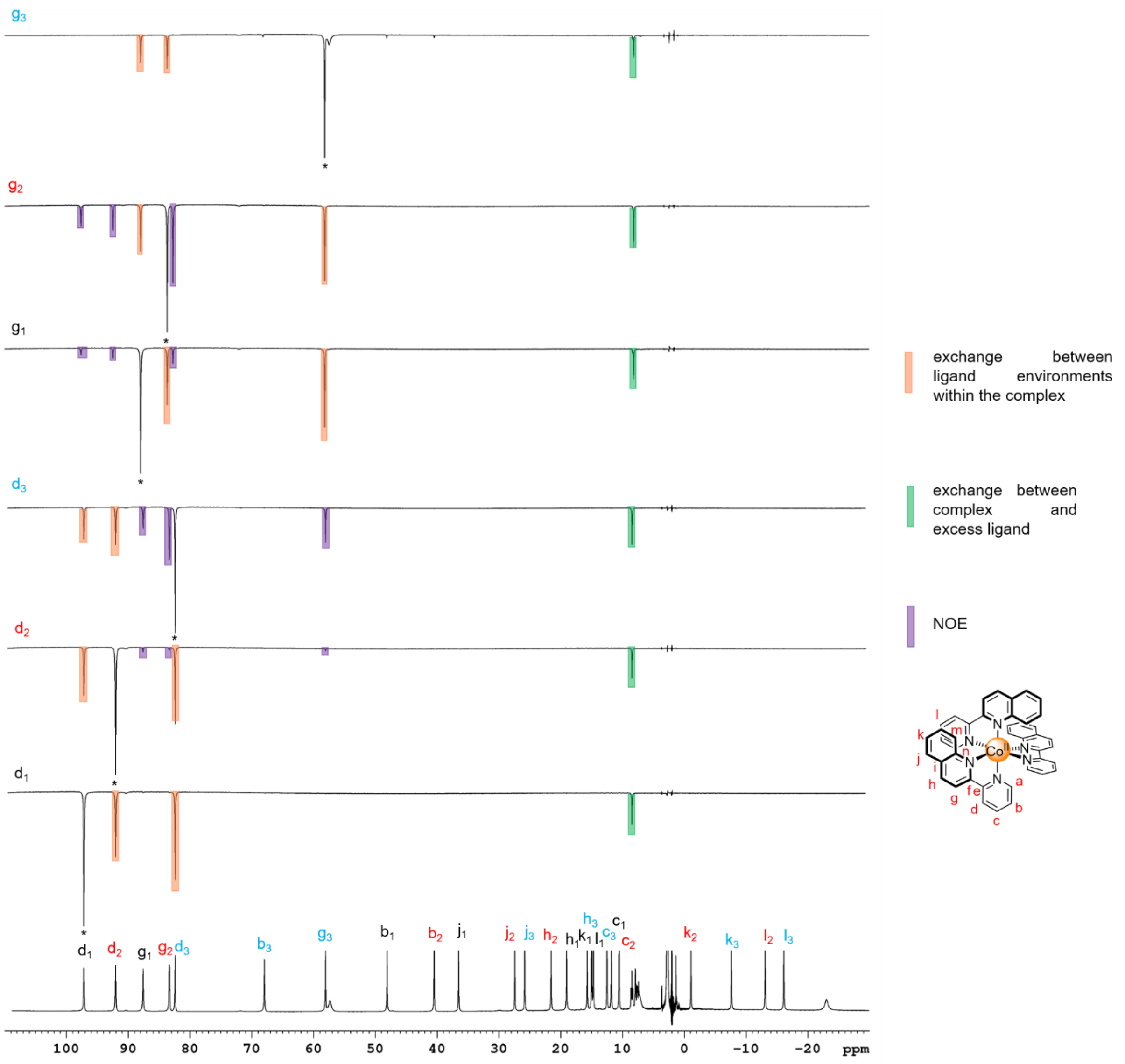

Figure S38. Steady-state NOE experiments (600 MHz, CD_3CN, 298 K) where the signals of protons *d* and *g* were sequentially irradiated (indicated by *) and exchange cross-peaks (orange) and NOE cross-peaks (purple) within $[Co(pq)_3](BF_4)_2$ were observed as well as exchange cross-peaks between the complex and excess ligand (green).

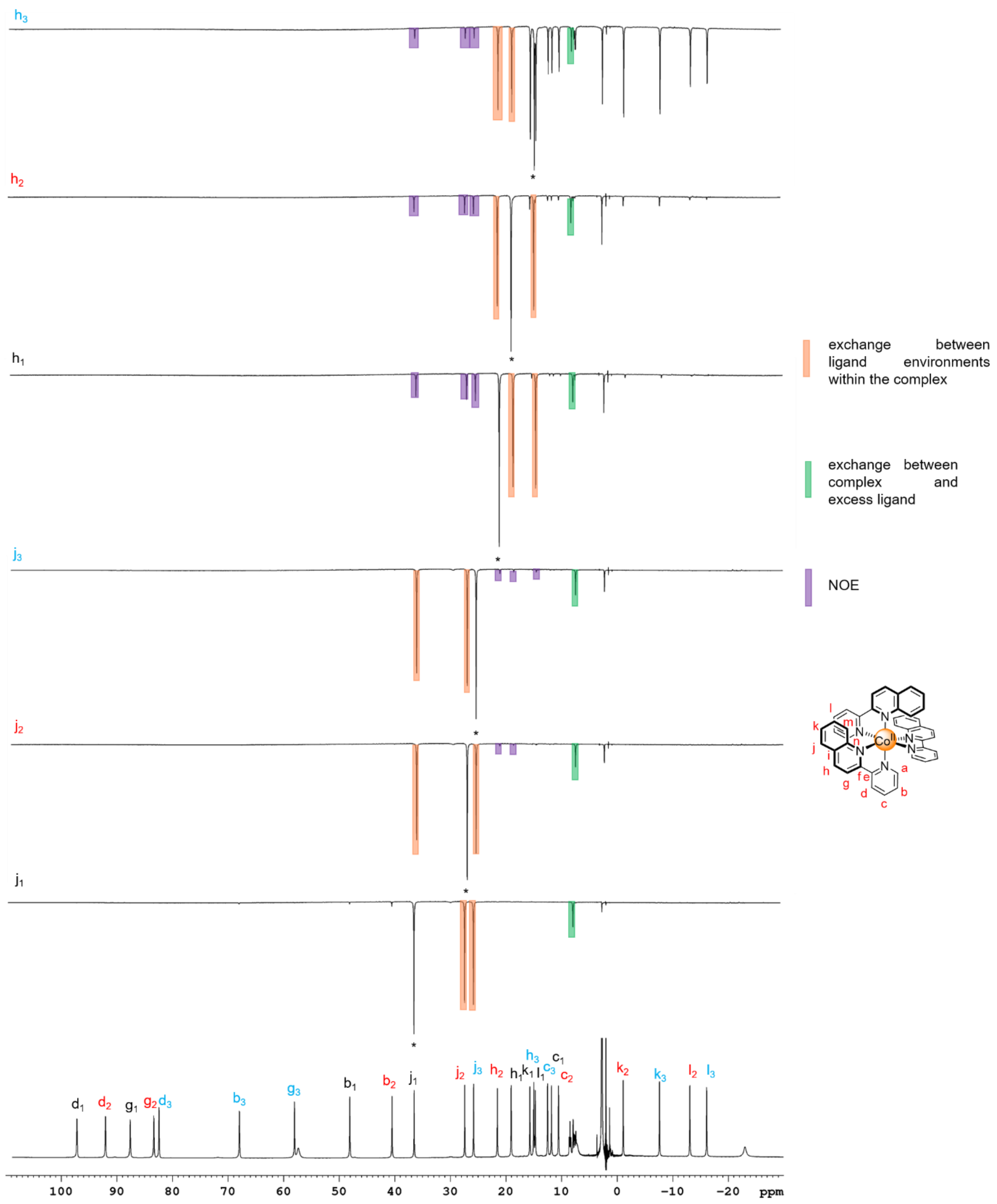

Figure S39. Steady-state NOE experiments (600 MHz, CD_3CN, 298 K) where the signals of protons *h* and *j* were sequentially irradiated (indicated by *) and exchange cross-peaks (orange) and NOE cross-peaks (purple) within $[Co(pq)_3](BF_4)_2$ were observed. However, cross-excitation was also observed upon irradiation of proton h_3 due to its proximity to other proton signals.

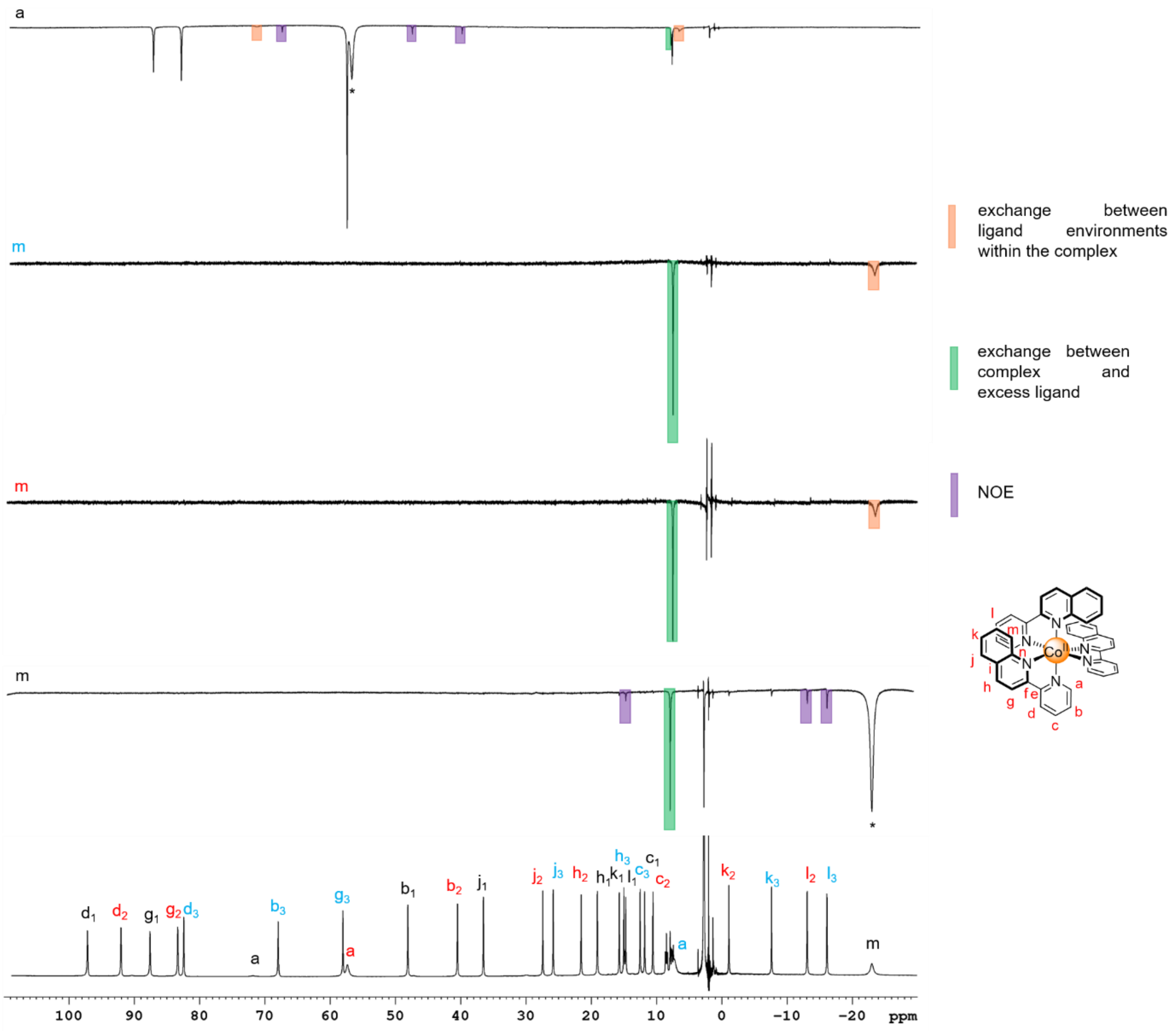

Figure S40. Steady-state NOE experiments (600 MHz, CD_3CN, 298 K) where the signals of protons *a* and *m* were identified by irradiation of the broad peaks. Exchange cross-peaks (orange) within $[Co(pq)_3](BF_4)_2$ as well as those between the complex and excess ligand (purple) were observed. For irradiation of proton *a*, cross-excitation of g_3 was also observed (unlabelled signals) due to its similar chemical shift to proton *a*. Protons *a* labelled in black and blue are tentatively assigned due to the weak exchange cross-peaks observed upon irradiation of proton *a* with the red label.

The assignments of quaternary carbons *l, n, e, f* in Figure S41 were made based on comparison to the reference complex $[Co(bpy)_3](BF_4)_2$ (Section 3.1.9) since TOCSY and HMBQ paramagnetic methods were not available. While doublets are expected for protons *a* and *m*, broad signals were observed due to the proximity to the Co^{II} paramagnetic centre. These carbon signals were distinguished from the quaternary carbon signals on the basis of their broader linewidths and the *a* carbons were assigned by comparison to the reference complex $[Co(bpy)_3](BF_4)_2$ (Section 3.1.9).

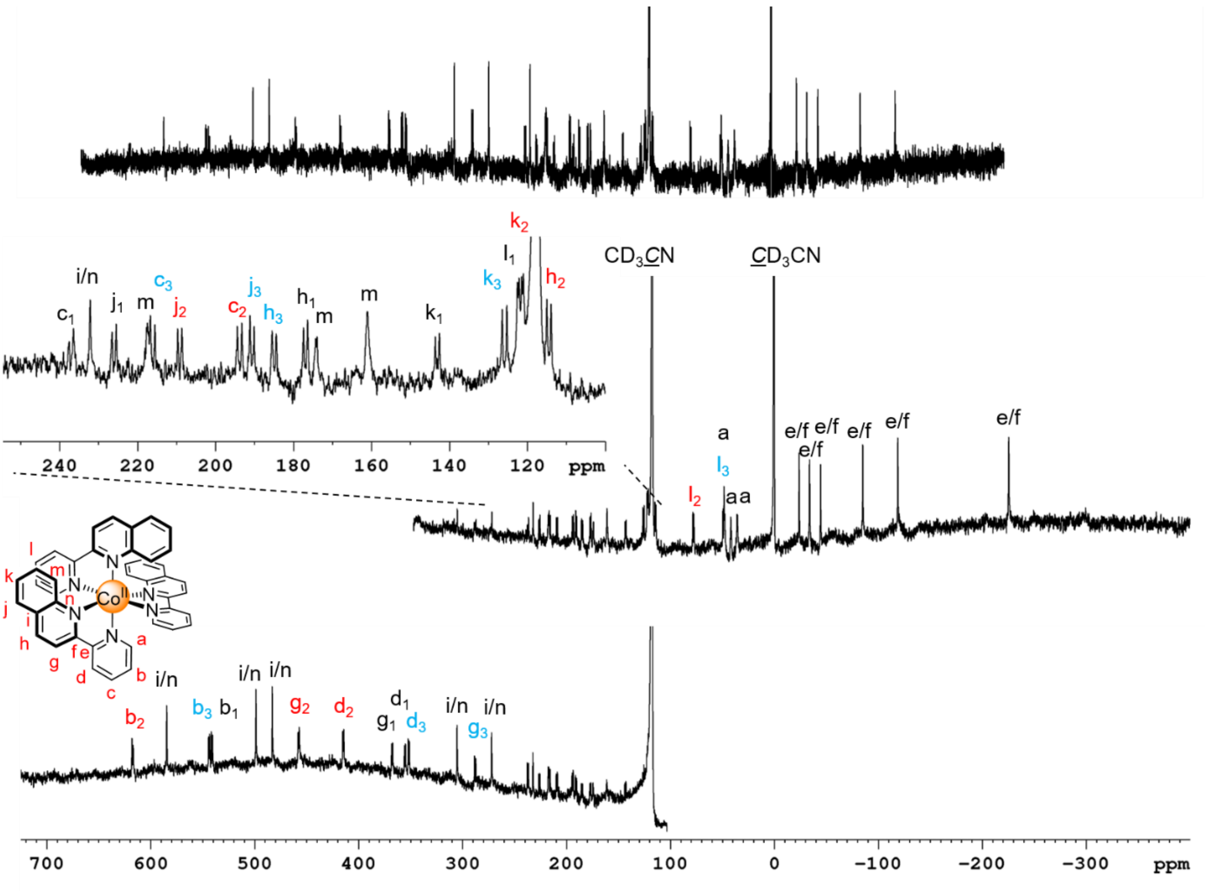

Figure S41. ^{13}C NMR spectrum (151 MHz, CD_3CN, 298 K) of $[Co(pq)_3](BF_4)_2$.

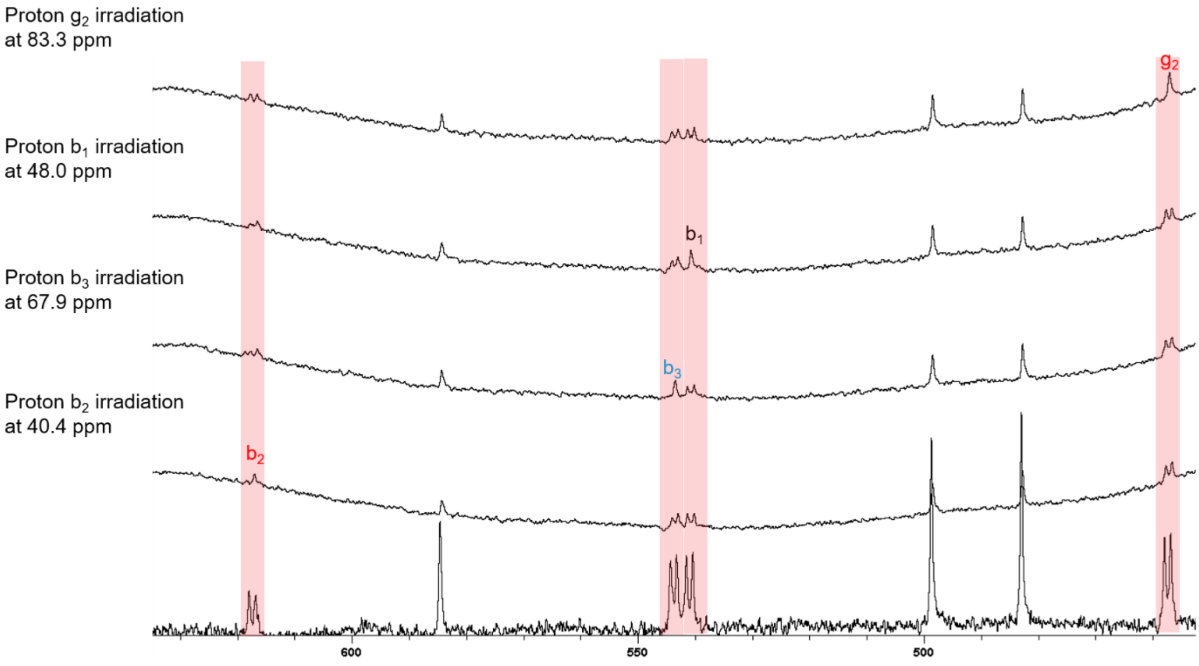

Figure S42. Selective ^1H-decoupling ^{13}C spectra (151 MHz, CD_3CN, 298 K) of $[Co(pq)_3](BF_4)_2$ for identifying $^1J_{CH}$ coupling in the region from 400 – 650 ppm.

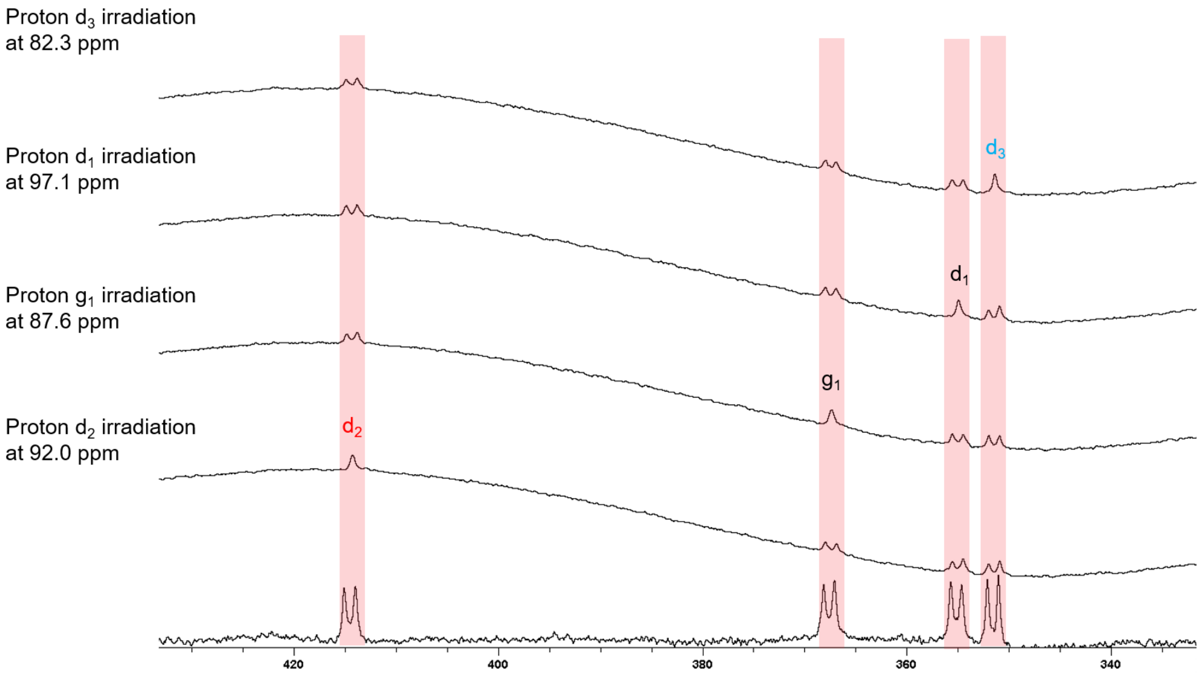

Figure S43. Selective ^{1}H-decoupling ^{13}C spectra (151 MHz, CD_3CN, 298 K) of $[Co(pq)_3](BF_4)_2$ for identifying $^{1}J_{CH}$ coupling in the region from 340 – 420 ppm.

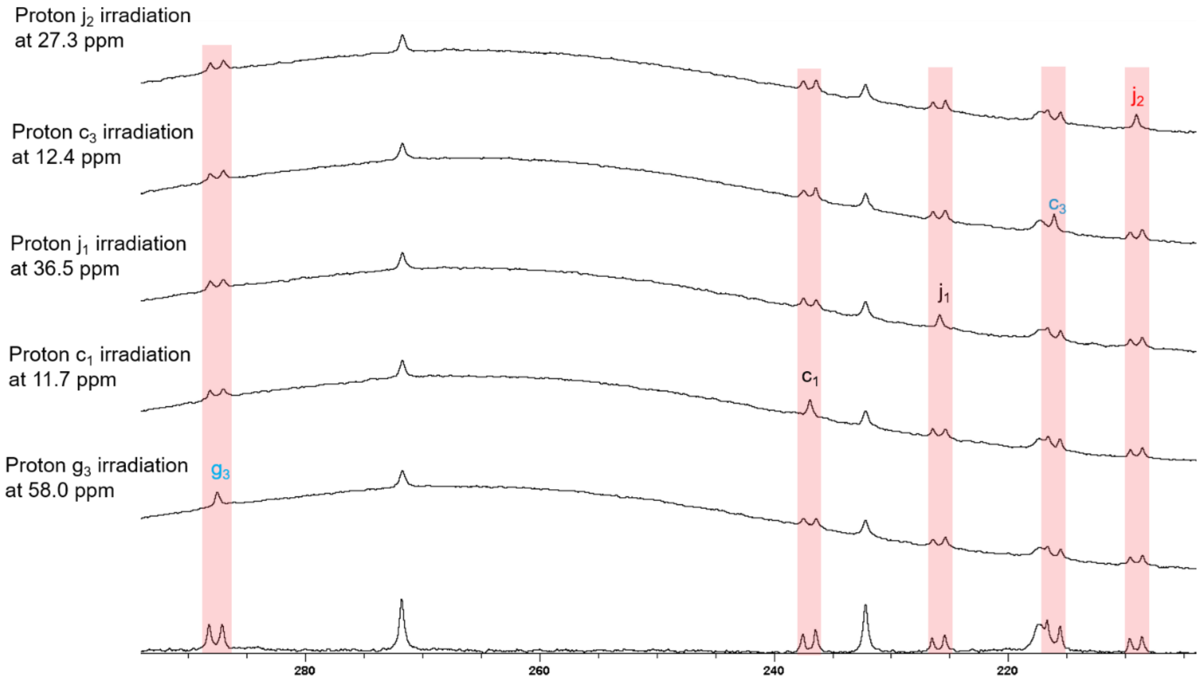

Figure S44. Selective ^{1}H-decoupling ^{13}C spectra (151 MHz, CD_3CN, 298 K) of $[Co(pq)_3](BF_4)_2$ for identifying $^{1}J_{CH}$ coupling in the region from 200 – 300 ppm.

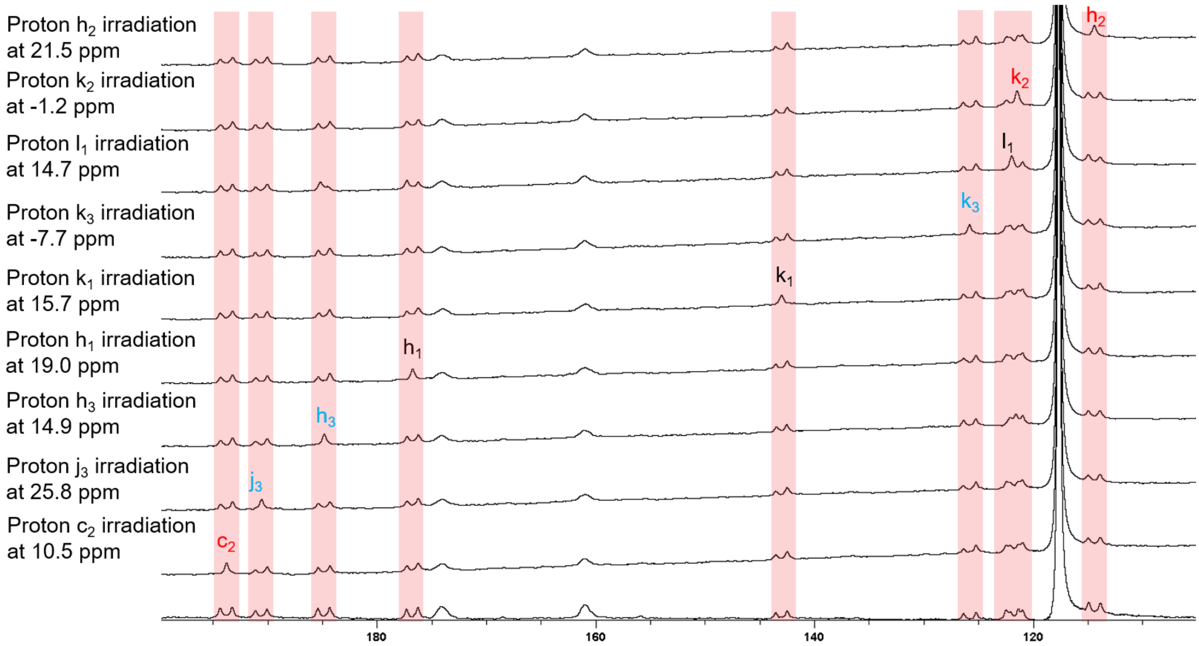

Figure S45. Selective ^{1}H-decoupling ^{13}C spectra (151 MHz, CD_3CN, 298 K) of $[Co(pq)_3](BF_4)_2$ for identifying $^1J_{CH}$ coupling in the region from 100 – 200 ppm.

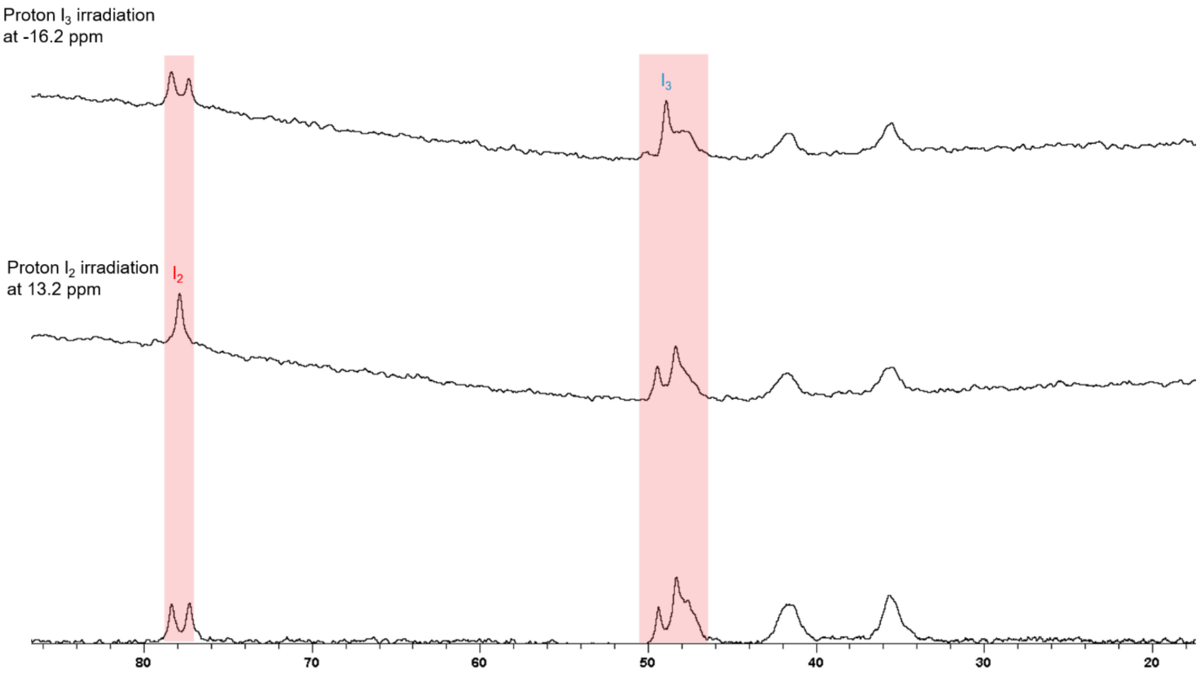

Figure S46. Selective ^{1}H-decoupling ^{13}C spectra (151 MHz, CD_3CN, 298 K) of $[Co(pq)_3](BF_4)_2$ for identifying $^1J_{CH}$ coupling in the region from 20 – 80 ppm.

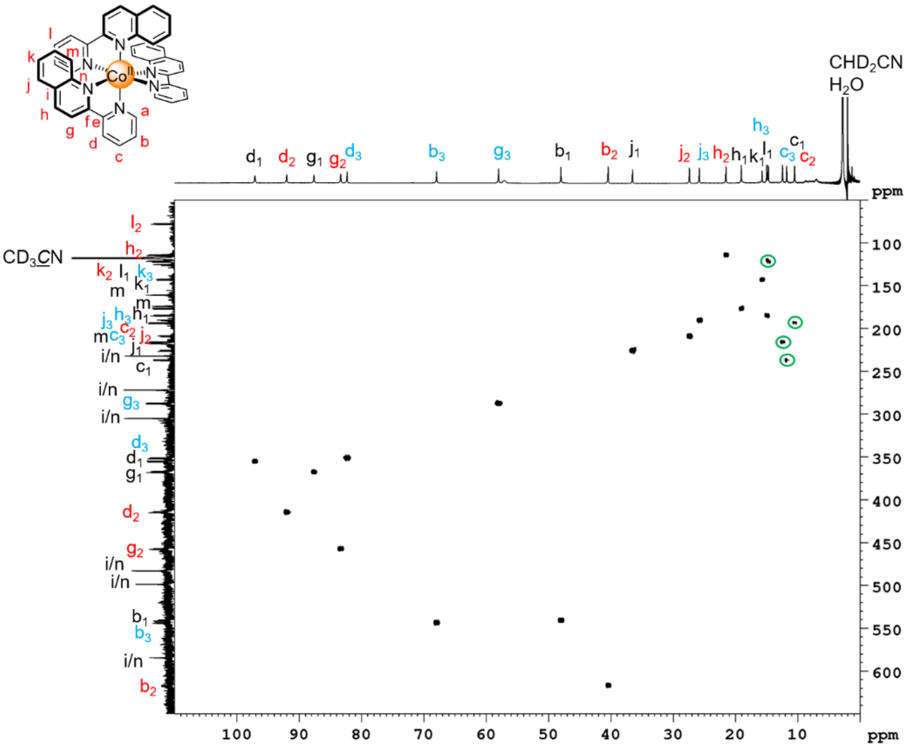

Figure S47. Downfield inset of ^{1}H-^{13}C HMQC NMR spectrum (600 MHz/151 MHz, CD_3CN, 298 K) of $[Co(pq)_3](BF_4)_2$. The decreased intensity of the cross-peaks in the green circles is attributed to non-uniform excitation over the large spectral range.

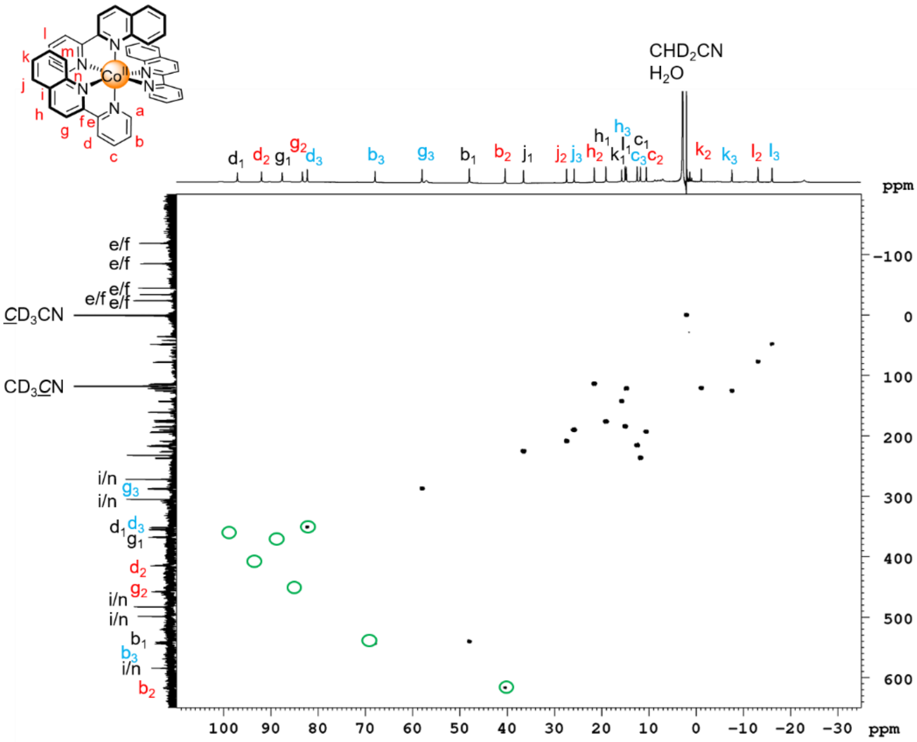

Figure S48. Upfield inset of ^{1}H-^{13}C HMQC NMR spectrum (600 MHz/151 MHz, CD_3CN, 298 K) of $[Co(pq)_3](BF_4)_2$ showing the absence of expected cross-peaks (green circles) for protons in the downfield region as a result of non-uniform excitation of the large spectral range. These cross-peaks are visible in the downfield inset in Figure S47. For assignments of the carbons between 0-200 ppm see Figure S49.

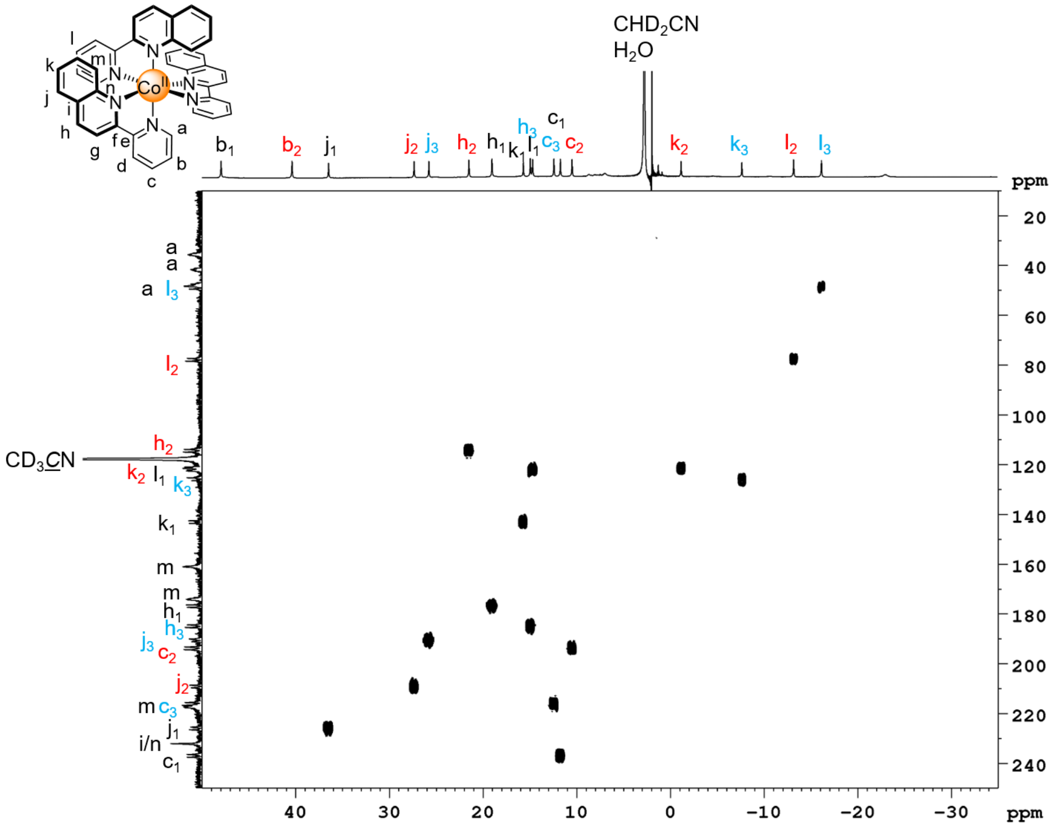

Figure S49. Inset of ^{1}H-^{13}C HMQC NMR spectrum (600 MHz/151 MHz, CD_3CN, 298 K) of $[Co(pq)_3](BF_4)_2$.

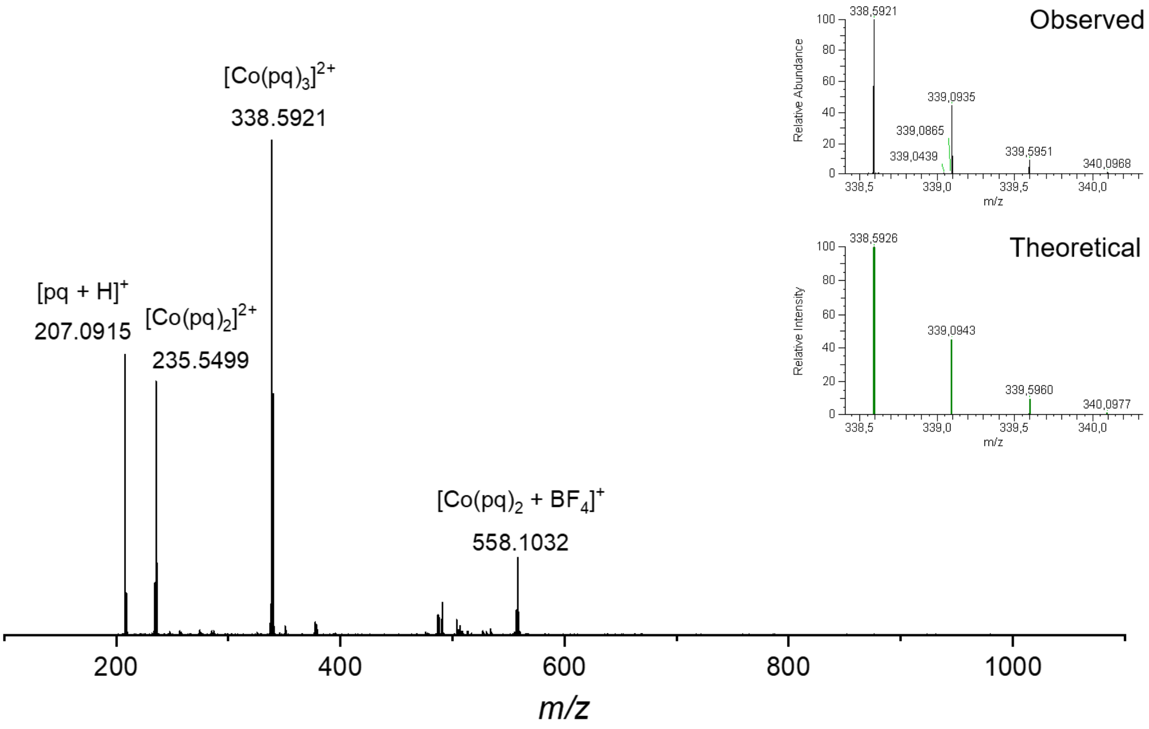

Figure S50. High resolution ESI mass spectrum of $[Co(pq)_3](BF_4)_2$ showing inset the observed (top) and theoretical (bottom) isotope patterns.

3.1.2 *mer*-[Co(pq-5'-Br)$_3$](BF$_4$)$_2$ (**2a**)

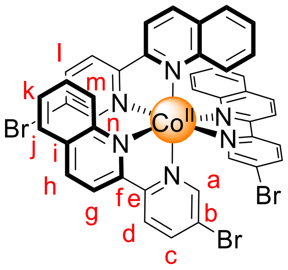

Co(BF$_4$)$_2$•6H$_2$O (3.00 mg, 8.81 µmol) and 2-(5'-bromopyridin-2'-yl)quinoline (7.51 mg, 26.4 µmol) were dissolved in CD$_3$CN (0.5 mL) and the solution was heated to 50 °C for 68 h. A ^{1}H NMR spectrum was measured before an additional equivalent of 2-(5'-bromo-pyridin-2'-yl)quinoline (2.50 mg, 8.81 µmol) was added and the solution was heated to 50 °C for 24 h.

^{1}H NMR (600 MHz, CD$_3$CN, 298 K) δ (ppm): 106.1 (s, 1H, H_d), 99.7 (s, 1H, H_d), 93.4 (s, 1H, H_g), 92.9 (s, 1H, H_g), 82.6 (s, 1H, H_d), 60.2 (s, 1H, H_g), 59.7 (bs, H_a), 39.1 (s, 1H, H_j), 31.2 (s, 1H, H_j), 28.8 (s, 1H, H_j), 25.7 (s, 1H, H_h), 23.6 (s, 1H, H_h), 17.6 (s, 1H, H_h), 16.3 (s, 1H, H_k), 11.3 (s, 1H, H_l), 10.5 (s, 1H, H_c), 6.7 (s, 1H, H_c), 5.9 (s, 1H, H_c), 0.7 (s, 1H, H_k), -6.0 (s, 1H, H_k), -14.5 (s, 1H, H_l), -15.4 (s, 1H, H_l), -43.0 (bs, H_m), -154.6 (bs, H_m), -156.3 (bs, H_m).

HRMS (ESI) *m/z*: 715.9205 [Co(pq-5'-Br)$_2$ + BF$_4$]$^+$, 456.4559 (calculated for $C_{42}H_{27}N_6Br_3Co$: 456.4574) [Co(pq-5'-Br)$_3$]$^{2+}$, 314.4588 [Co(pq-5'-Br)$_2$]$^{2+}$.

3.1.2.1 Characterisation using Paramagnetic NMR Spectroscopy and Mass Spectrometry

Characterisation of *mer*-[Co(pq-5'-Br)$_3$](BF$_4$)$_2$ was carried out using the sample in the presence of excess ligand (4 equiv.) since in the presence of 3 equivalents of ligand a second species, attributed to a Co(pq-Br)$_2$-based species (purple boxes), was also present. Addition of a fourth equivalent of ligand changed the speciation and the major species in solution became *mer*-[Co(pq-5'-Br)$_3$](BF$_4$)$_2$ (Figure S51). While the protons of the three ligand environments of the *mer* complex were identified, assignment to a particular ligand environment was not possible due to the absence of cross-peaks in the COSY spectrum (Figure S53). This is most likely due to the broadness of the signals and the presence of additional species, such as a Co(pq-Br)$_2$-based species. The signals in the ^{13}C NMR spectrum (Figure S55) were also broad despite recording 102,400 scans and only cross-peaks relating to the free ligand were observed in the HMQC spectrum (Figure S56). For this reason, the ^{13}C NMR data for the complex is not reported above.

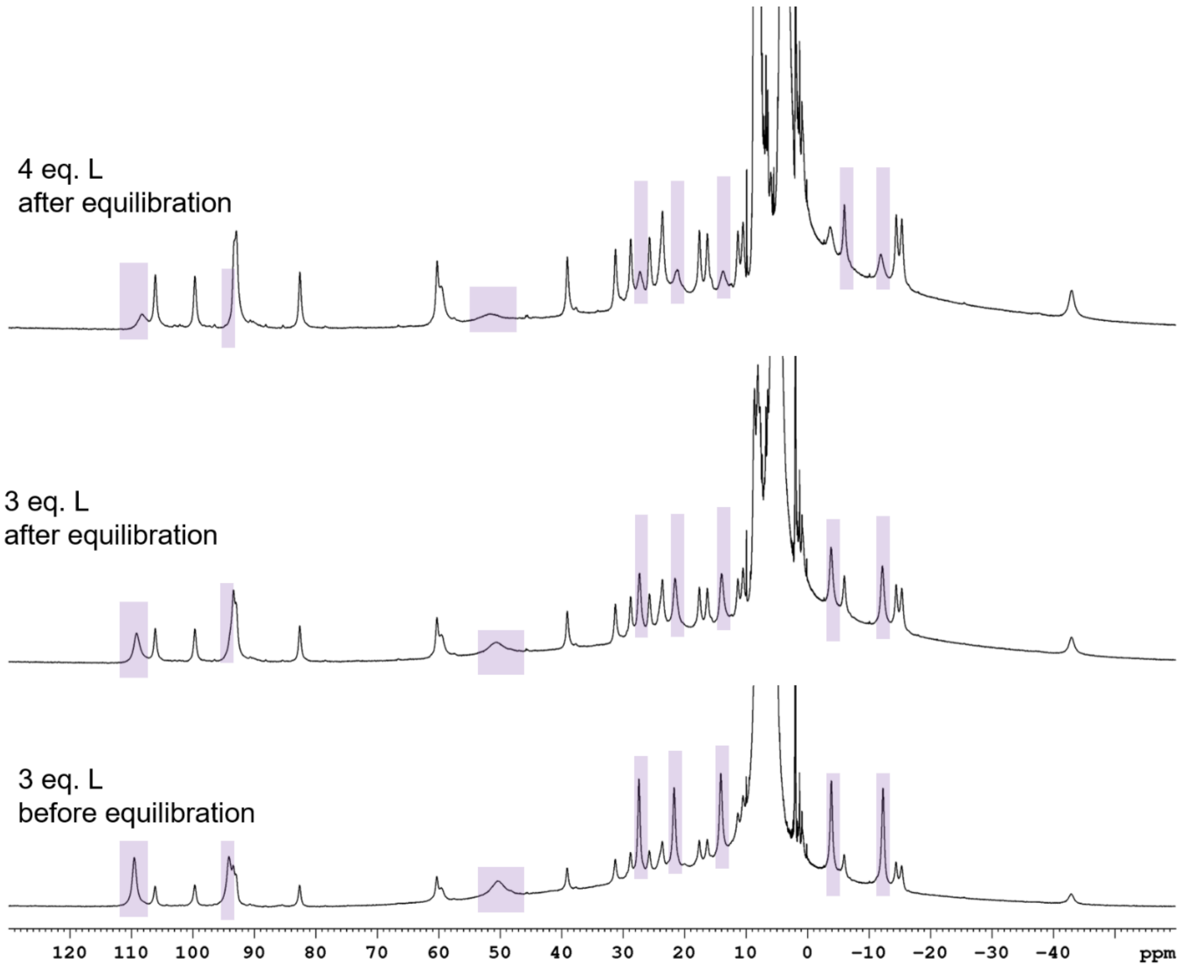

Figure S51. ^{1}H NMR spectra (500 MHz, CD_3CN, 298 K) of the complex mixture showing the change in speciation as the ligand dissolved following equilibration and upon addition of a 4th equivalent of the ligand. The signals in the purple boxes are attributed to a Co(pq-Br)$_2$-based species and the remaining signals are attributed to *mer*-Co(pq-Br)$_3$ (see Figure S52 for their assignment).

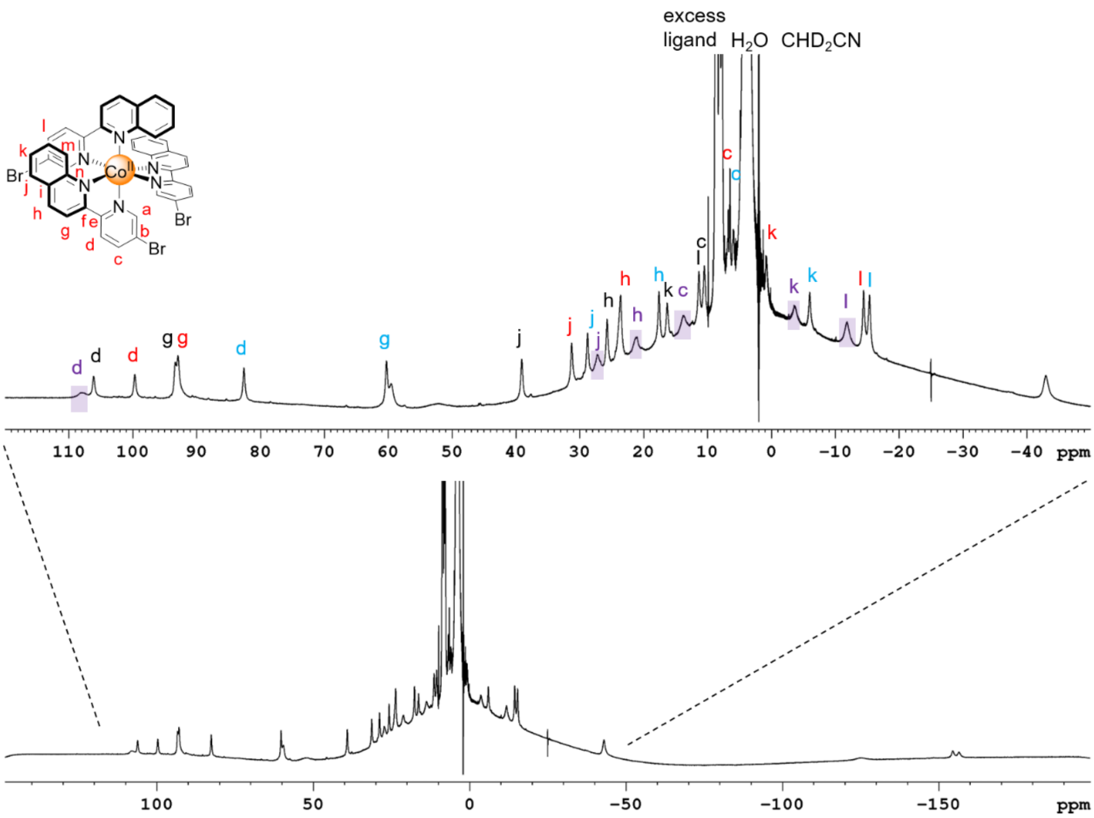

Figure S52. ^1H NMR spectrum (600 MHz, CD_3CN, 298 K) of $[Co(pq\text{-}5'\text{-}Br)_3](BF_4)_2$. The signals with black, red and blue labels correspond to the three ligand environments of the major species, *mer*-$Co(pq\text{-}5'\text{-}Br)_3$. Since the spin system for each ligand environment could not be identified by COSY (Figure S53), the three sets of signals are arbitrarily labelled with black, red and blue labels according to their decreasing chemical shift. Purple signals are attributed to a $Co(pq\text{-}5'\text{-}Br)_2$ based species.

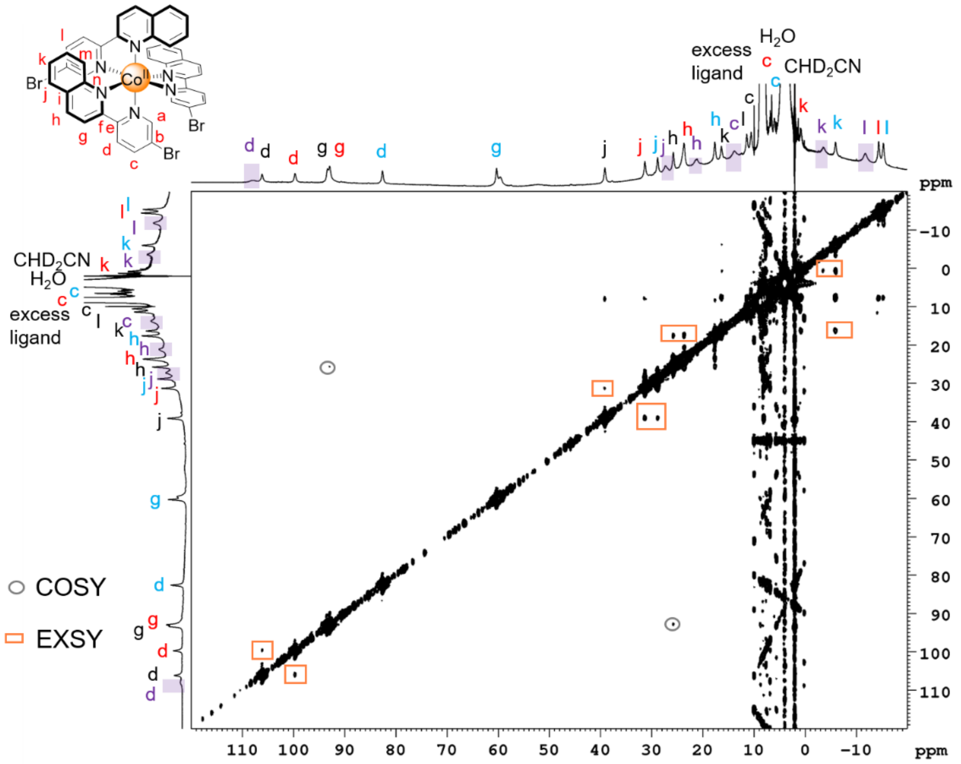

Figure S53. ^{1}H-^{1}H COSY NMR spectrum (600 MHz, CD_3CN, 298 K) of $[Co(pq\text{-}5'\text{-}Br)_3](BF_4)_2$. Grey circles represent through-bond cross-peaks (COSY) but due to overlapping signals they cannot be assigned to a particular spin system of the three ligand environments. The orange squares represent exchange (EXSY) cross-peaks.

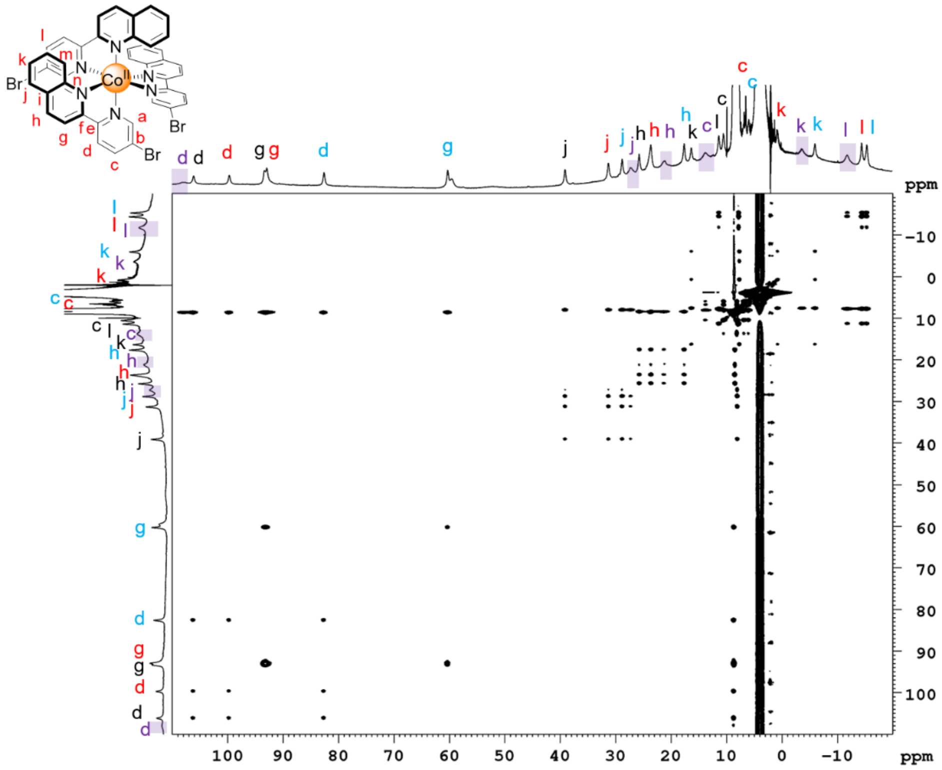

Figure S54. ^{1}H-^{1}H NOESY NMR spectrum (600 MHz, CD_3CN, 298 K) of $[Co(pq\text{-}5'\text{-}Br)_3](BF_4)_2$.

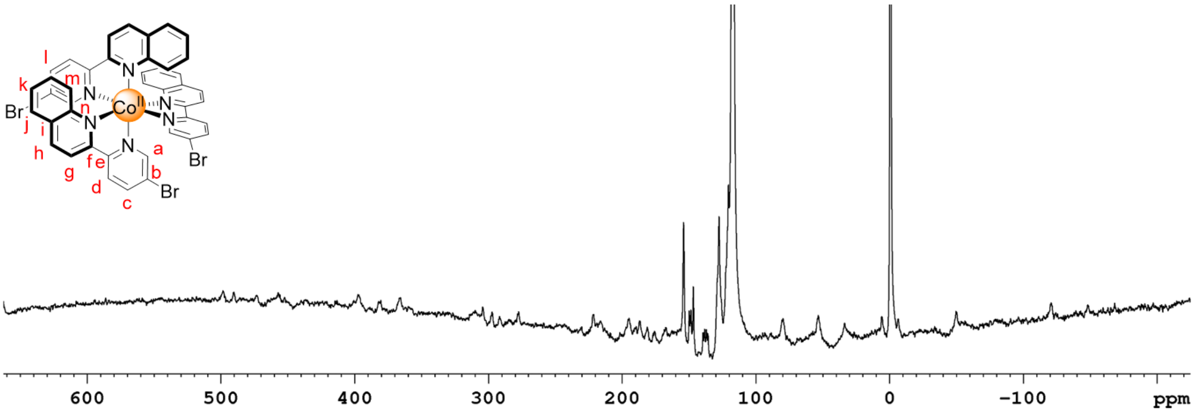

Figure S55. ^{13}C NMR spectrum (151 MHz, CD_3CN, 298 K) of $[Co(pq\text{-}5'\text{-}Br)_3](BF_4)_2$.

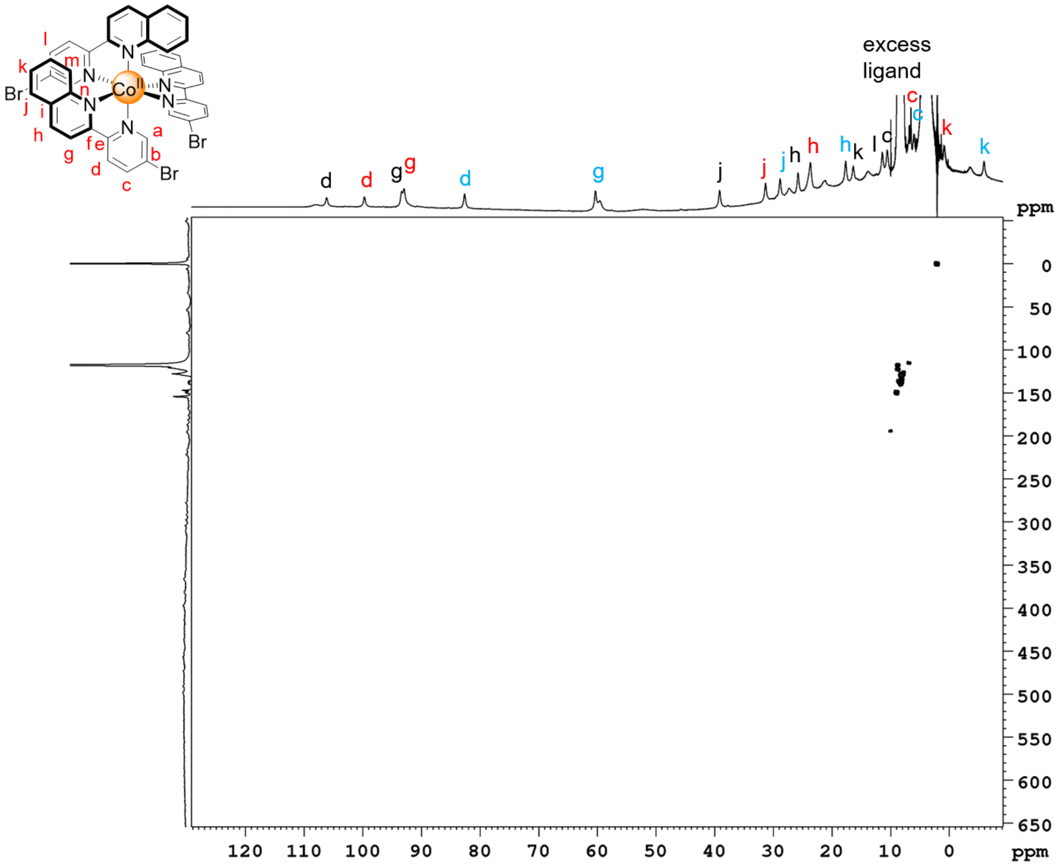

Figure S56. ^{1}H-^{13}C HMQC NMR spectrum (600 MHz/151 MHz, CD_3CN, 298 K) of $[Co(pq\text{-}5'\text{-}Br)_3](BF_4)_2$ showing only cross-peaks for excess ligand.

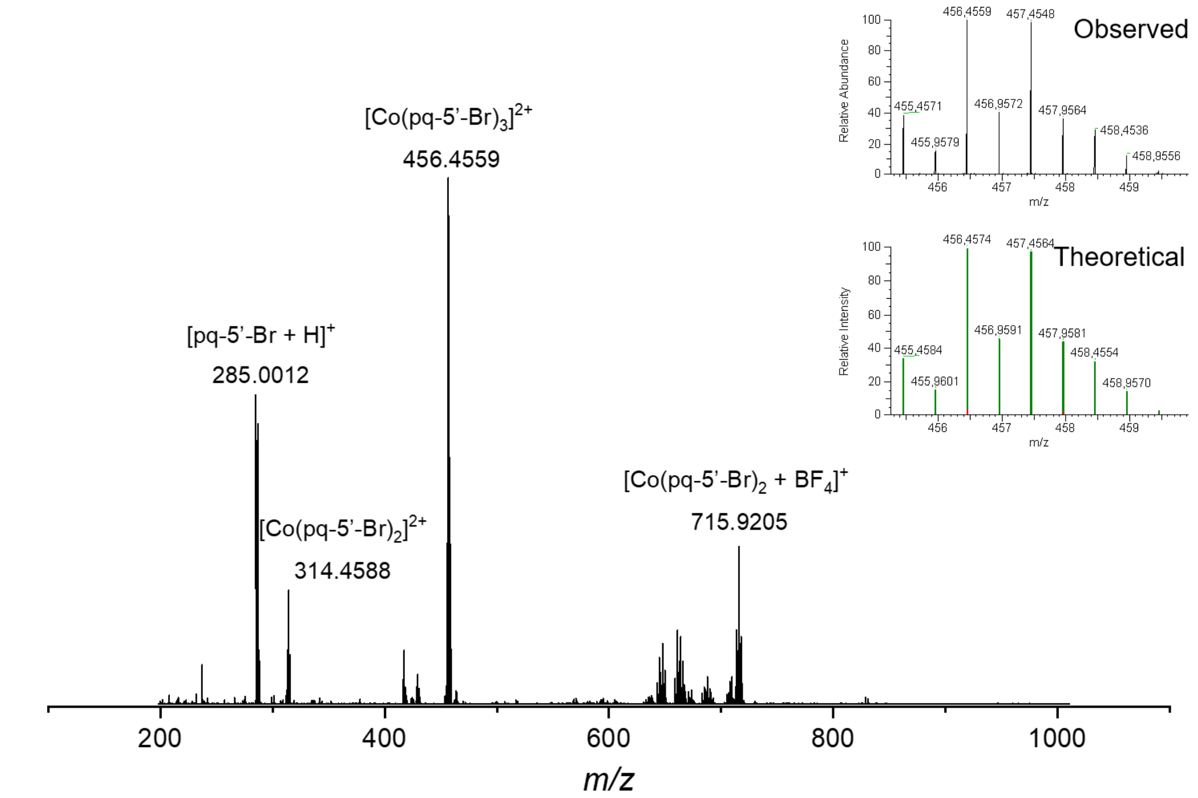

Figure S57. High resolution ESI mass spectrum of $[Co(pq\text{-}5'\text{-}Br)_3](BF_4)_2$ showing in the inset the observed (top) and theoretical (bottom) isotope patterns.

3.1.3 *mer*-$[Co(pq\text{-}5'\text{-}CCH)_3](BF_4)_2$ (**3a**)

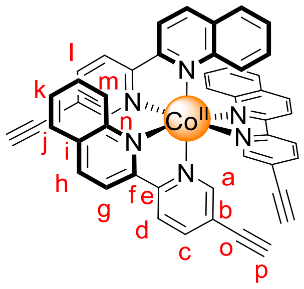

$Co(BF_4)_2$•$6H_2O$ (3.01 mg, 8.84 µmol) and 2-(5'-ethynylpyridin-2'-yl)quinoline (6.09 mg, 26.4 µmol) were dissolved in CD_3CN (0.5 mL) and the solution was heated to 50 °C for 24 h.

^{1}H NMR (600 MHz, CD_3CN, 298 K) δ (ppm): 95.8 (s, 1H, H_{d1}), 92.5 (s, 1H, H_{d2}), 91.4 (s, 1H, H_{g1}), 90.3 (s, 1H, H_{g2}), 76.1 (s, 1H, H_{d3}), 59.6 (s, 1H, H_{g3}), 57.2 (bs, H_a), 38.6 (s, 1H, H_{j1}), 30.3 (s, 1H, H_{j2}), 28.0 (s, 1H, H_{j3}), 24.8 (s, 1H, H_{h2}), 22.3 (s, 1H, H_{h1}), 17.2 (s, 1H, H_{h3}), 16.1 (s, 1H, H_{k1}), 12.8 (s, 1H, H_{c1}), 11.7 (s, 1H, H_l), 9.9 (s, 1H, H_{c2}), 9.1 (s, 1H, H_{c3}), 7.7 (s, 1H, H_p), 0.3 (s, 1H, H_{k2}), -2.0 (s, 1H, H_p), -6.5 (s, 1H, H_{k3}), -7.2 (s, 1H, H_p), -14.9 (s, 2H, H_l), -41.0 (bs, H_m), -152.3 (bs, H_m), -159.1 (bs, H_m).

HRMS (ESI) *m/z*: 374.5914 (calculated for $C_{48}H_{30}N_6Co$: 374.5926) $[Co(pq\text{-}5'\text{-}CCH)_3]^{2+}$, 259.5497 $[Co(pq\text{-}5'\text{-}CCH)_2]^{2+}$.

3.1.3.1 Characterisation using Paramagnetic NMR Spectroscopy and Mass Spectrometry

Despite the broader linewidths in comparison to the spectrum of $[Co(pq)_3](BF_4)_2$ (Table S5, Section 3.1.8), cross-peaks were observed in the COSY and NOESY spectra of $[Co(pq\text{-}CCH)_3](BF_4)_2$ (Figures S59-S60) allowing assignment of the ^{1}H NMR spectrum

(Figure S58). As well as the major species *mer*-[Co(pq-CCH)$_3$](BF_4)$_2$, signals attributed to a Co(pq-CCH)$_2$-based species (purple signals assigned from the NOESY spectrum) and *fac*-[Co(pq-CCH)$_3$](BF_4)$_2$ (green signals) were identified. Assignment of the signals for *fac*-[Co(pq-CCH)$_3$](BF_4)$_2$ below 40 ppm was complicated by overlapping signals and the presence of artefacts; the species is estimated to constitute less than 1% of the complex mixture necessitating from increasing the intensity of the NOESY spectrum to see any cross-peaks (Figure S61). However, closer inspection of the region between 40 and 110 ppm showed cross-peaks for protons *d* and *g* (green signals) and cross-peaks for a fourth yet unidentified species (orange squares) were also observed even though these signals were not observable in the ^{1}H NMR spectrum (Figure S58). This highlights the sensitivity of NOESY for assigning mixtures of exchanging complexes.

Assignment of the ^{13}C NMR spectrum (Figure S62) was not possible due to the broadness of signals and poor HMQC data, most likely due to presence of multiple species at equilibrium. In the HMQC no cross-peaks were observed in the downfield spectrum and only weak cross-peaks for the complex were observed in the upfield spectrum (Figure S63). Stronger cross-peaks were observed for the free ligand.

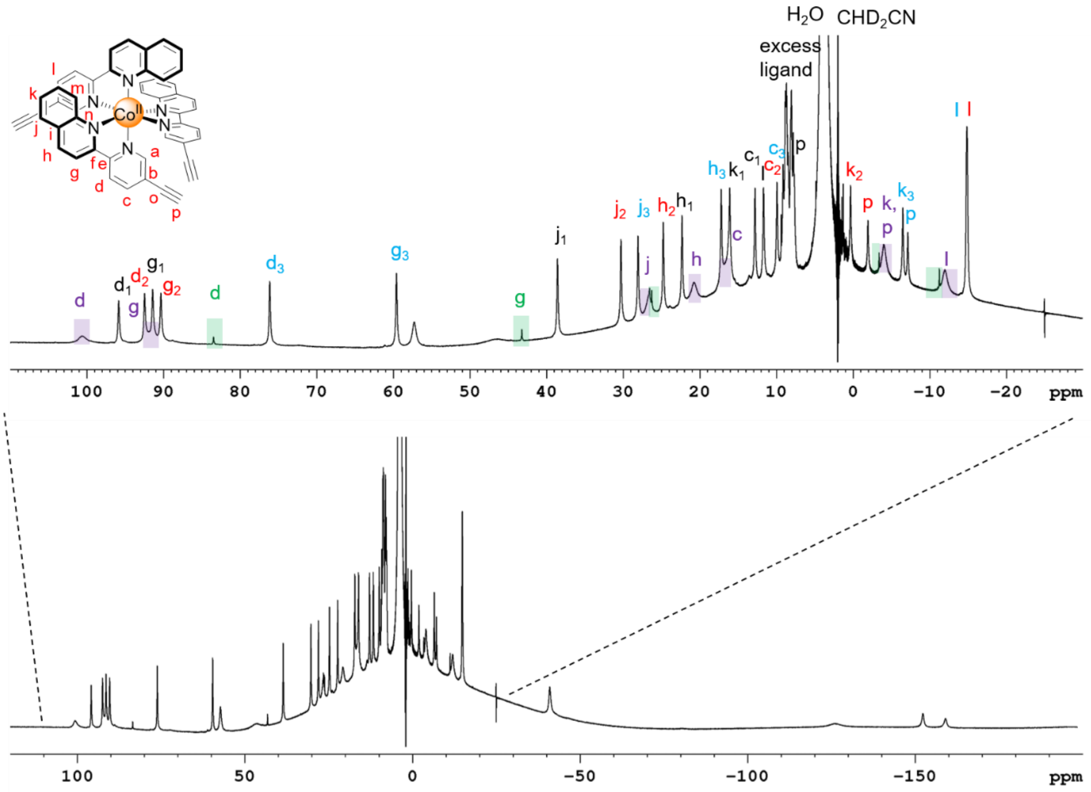

Figure S58. ^{1}H NMR spectrum (600 MHz, CD_3CN, 298 K) of [Co(pq-5'-CCH)$_3$](BF_4)$_2$. The signals with black, red and blue labels correspond to the three ligand environments of the major species, *mer*-Co(pq-5'-CCH)$_3$. Where present, the numbers indicate signals belonging to the same spin system as established by COSY NMR spectroscopy, however, different spin systems (i.e. protons *b*-*d* and protons *g*-*h*) could not be correlated using NOESY spectroscopy. Therefore, the three spin systems were arbitrarily labelled with black, red and blue labels in decreasing chemical shift order of protons *d*, *g* and *j*, respectively, to represent the three ligand environments. Protons *l* and *p* were arbitrarily labelled with black, red and blue labels according to their decreasing chemical shift. Purple signals are attributed to a Co(pq-5'-CCH)$_2$ based species whereas the green signals are attributed to *fac*-Co(pq-5'-CCH)$_3$.

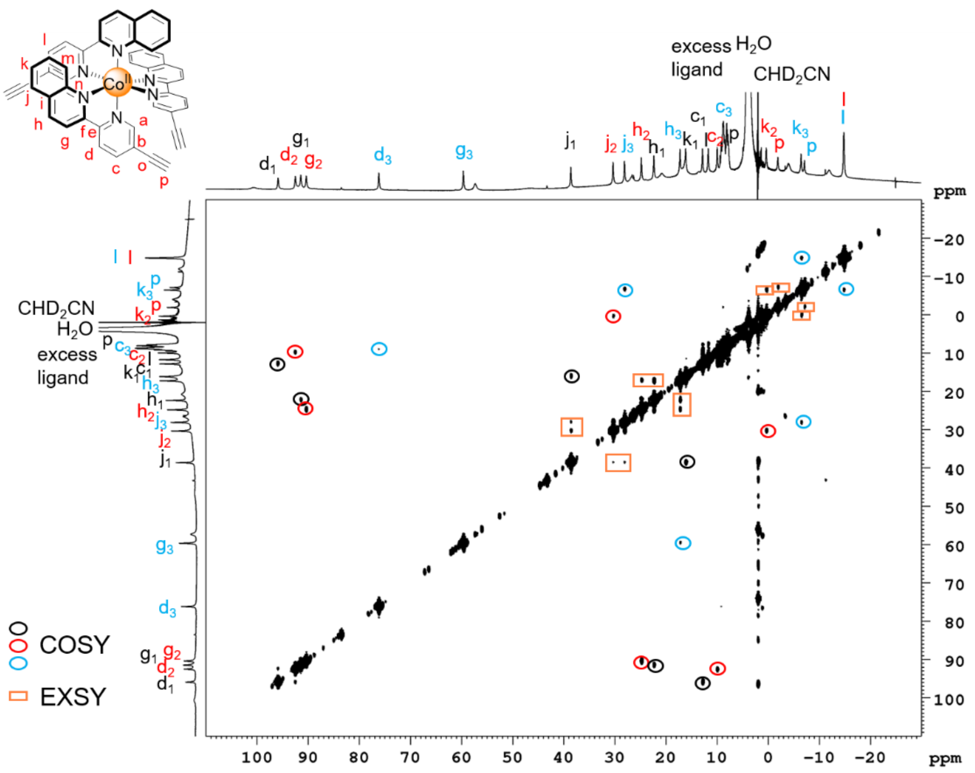

Figure S59. ^{1}H-^{1}H COSY NMR spectrum (600 MHz, CD_3CN, 298 K) of $[Co(pq\text{-}5'\text{-}CCH)_3](BF_4)_2$. The black, red and blue circles represent through-bond (COSY) cross-peaks within the three ligand environments whereas orange squares represent exchange (EXSY) cross-peaks between the three ligand environments.

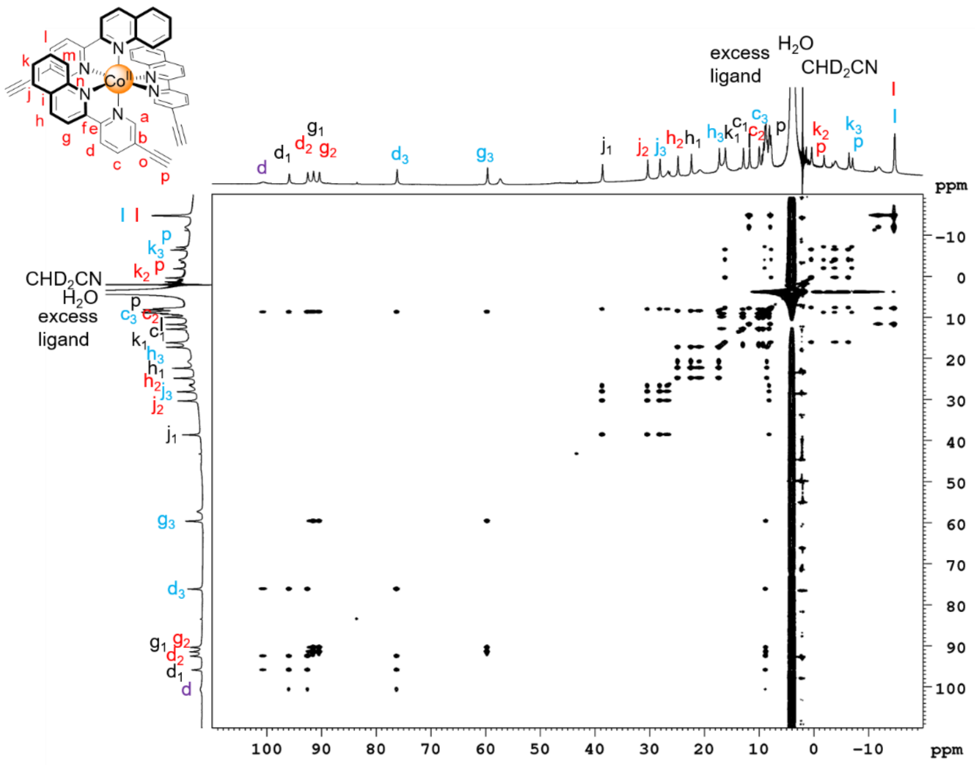

Figure S60. ^{1}H-^{1}H NOESY NMR spectrum (600 MHz, CD_3CN, 298 K) of $[Co(pq\text{-}5'\text{-}CCH)_3](BF_4)_2$.

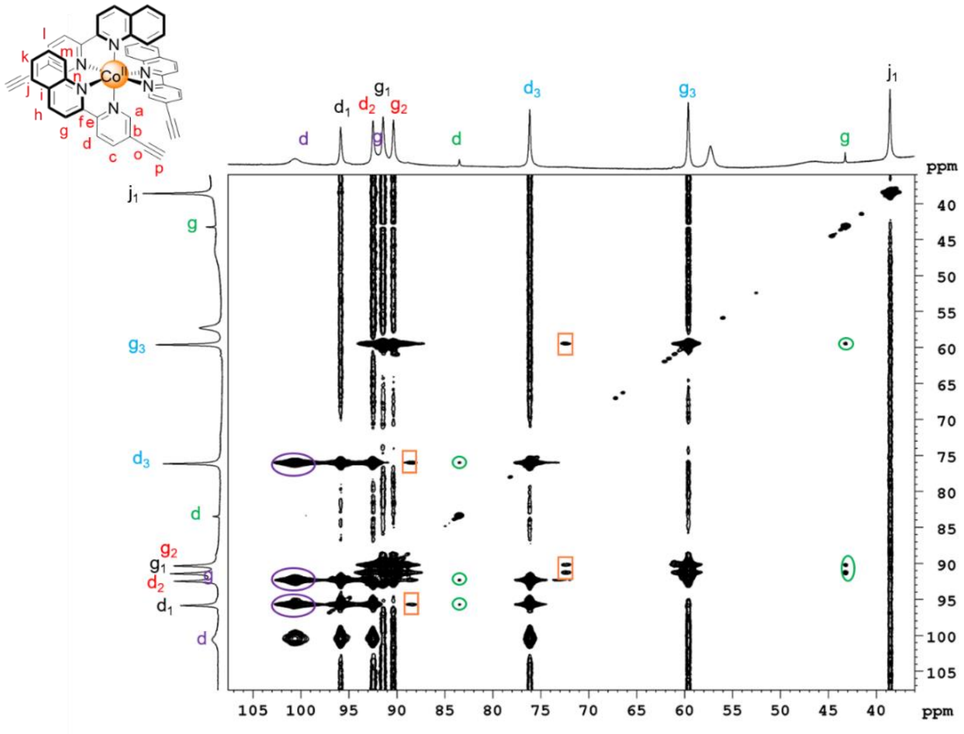

Figure S61. Inset of the ^{1}H-^{1}H NOESY NMR spectrum (600 MHz, CD_3CN, 298 K) of $[Co(pq\text{-}5'\text{-}CCH)_3](BF_4)_2$ showing exchange cross-peaks between the major species, *mer*-$Co(pq\text{-}5'\text{-}CCH)_3$, and minor species attributed to a $Co(pq\text{-}5'\text{-}CCH)_2$ based species (purple circles) and *fac*-$Co(pq\text{-}5'\text{-}CCH)_3$ (green circles) and an unidentified species not visible in the ^{1}H NMR spectrum (orange squares).

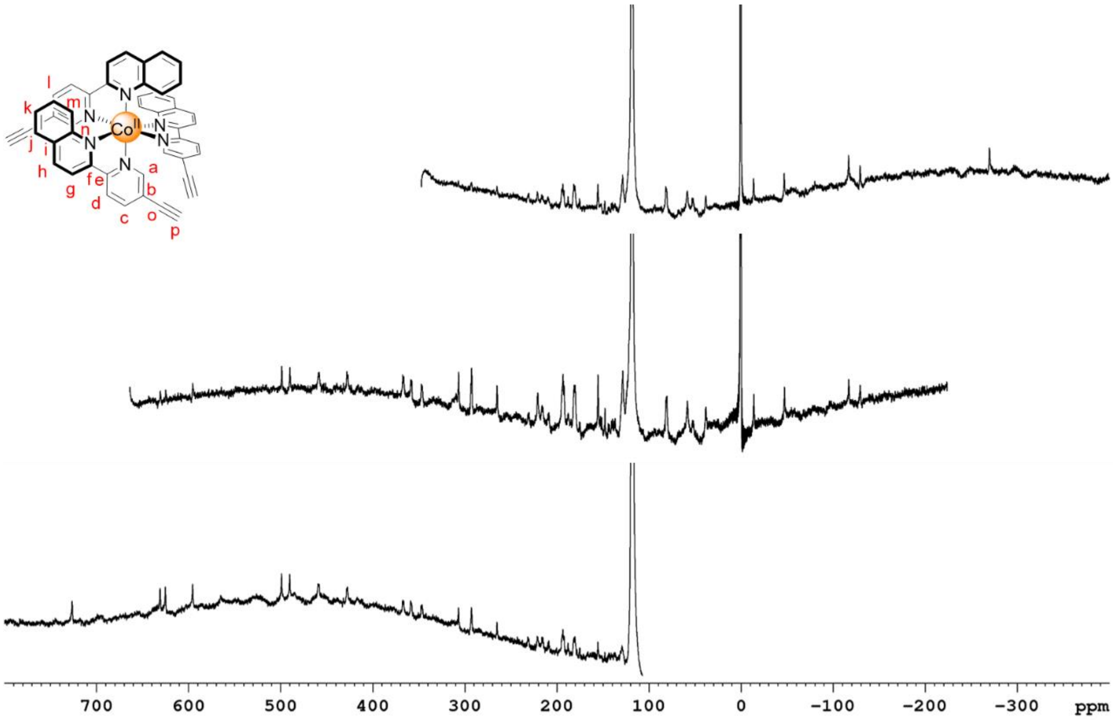

Figure S62. ^{13}C NMR spectrum (151 MHz, CD_3CN, 298 K) of $[Co(pq\text{-}5'\text{-}CCH)_3](BF_4)_2$. Assignment was not possible due to the absence of cross-peaks in the HMQC spectra and the number of overlapping signals from the four species present (*mer*-$Co(pq\text{-}5'\text{-}CCH)_3$, *fac*-$Co(pq\text{-}5'\text{-}CCH)_3$, a $Co(pq\text{-}5'\text{-}CCH)_2$-based species and free ligand).

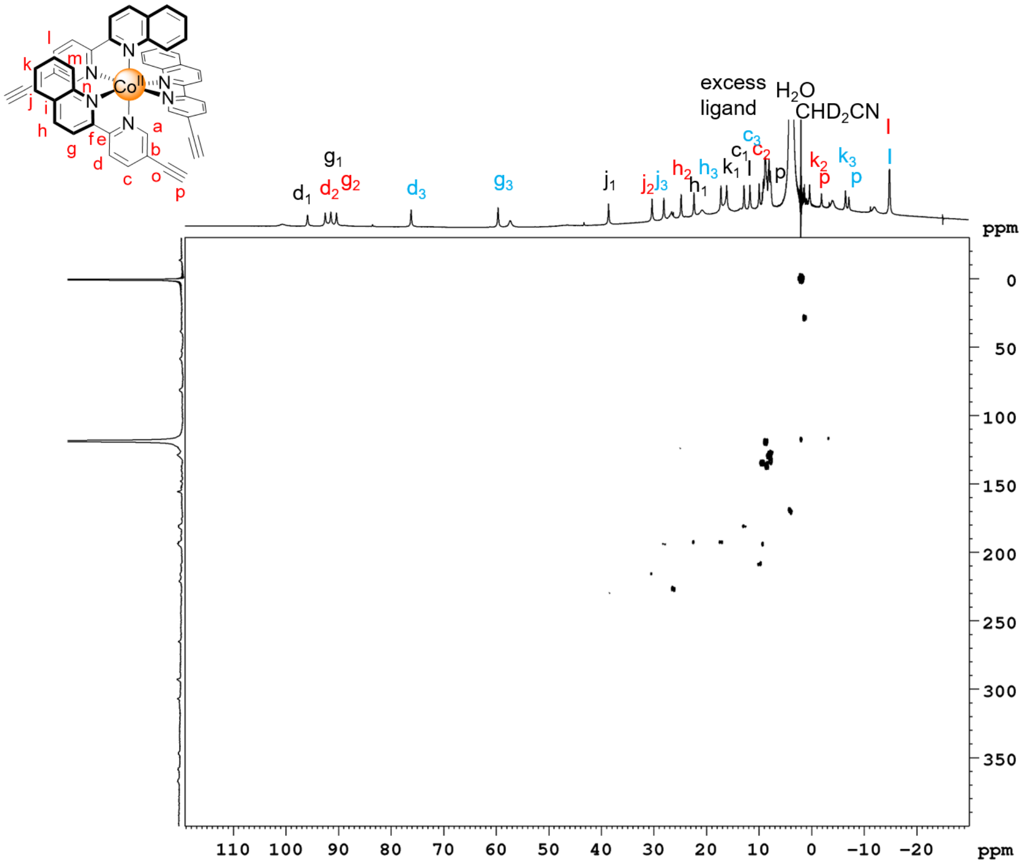

Figure S63. ^{1}H-^{13}C HMQC NMR spectrum (600 MHz/151 MHz, CD_3CN, 298 K) of $[Co(pq\text{-}5'\text{-}CCH)_3](BF_4)_2$.

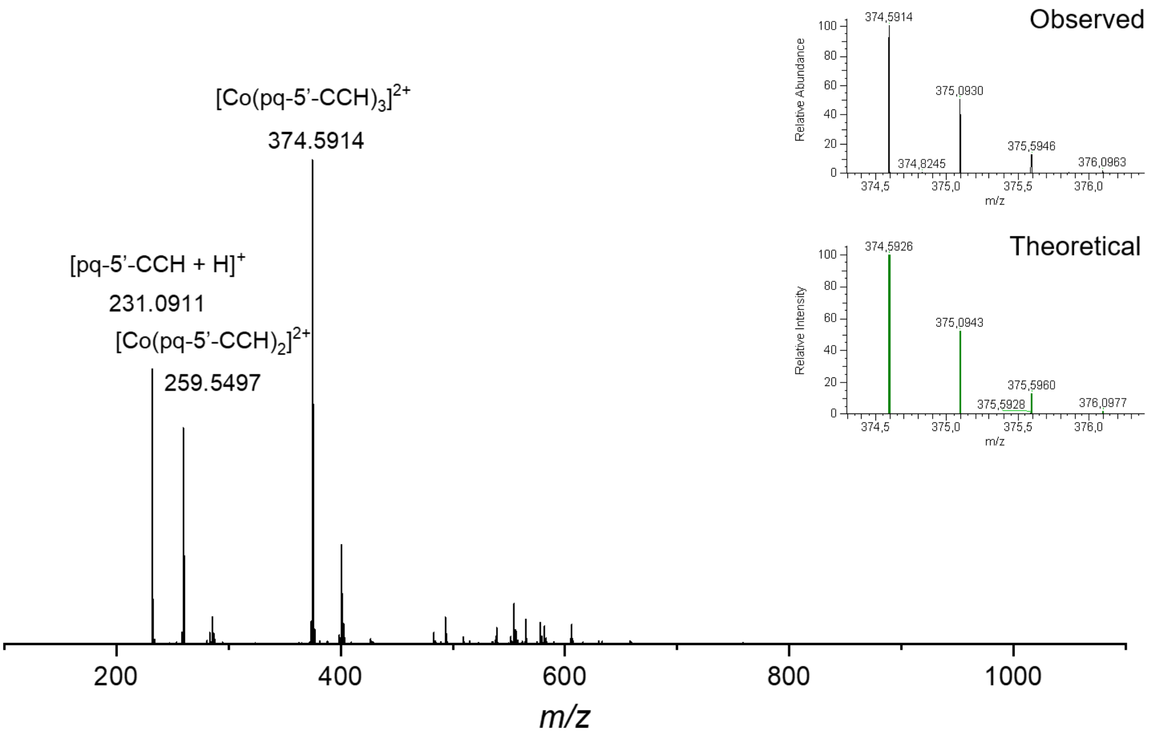

Figure S64. High resolution ESI mass spectrum of $[Co(pq\text{-}5'\text{-}CCH)_3](BF_4)_2$ showing in the inset the observed (top) and theoretical (bottom) isotope patterns.

3.1.4 *mer*-[Co(pq-5'-Ph)$_3$](BF$_4$)$_2$ (**4a**)

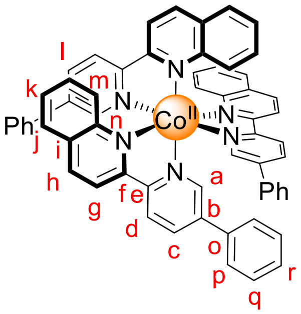

Co(BF$_4$)$_2$•6H$_2$O (3.06 mg, 8.98 µmol) and 2-(5'-phenylpyridin-2'-yl)quinoline (7.46 mg, 26.4 µmol) were dissolved in CD$_3$CN (0.5 mL) in an NMR tube and the solution was heated to 50 °C for 24 h.

^{1}H NMR (600 MHz, CD$_3$CN, 298 K) δ (ppm): 96.8 (s, 1H, H_{d1}), 95.0 (s, 1H, H_{d2}), 90.4 (s, 1H, H_{g1}), 84.4 (s, 1H, H_{g2}), 79.3 (s, 1H, H_{d3}), 57.0 (s, 1H, H_{g3}), 56.0 (bs, H_a), 37.4 (s, 1H, H_{j1}), 28.5 (s, 1H, H_{j2}), 25.3 (s, 1H, H_{j3}), 23.2 (s, 2H, H_{p1}), 21.4 (s, 1H, H_{h2}), 21.2 (s, 1H, H_{h1}), 16.5 (s, 1H, H_{k1}), 15.8 (s, 1H, H_{l1}), 15.3 (s, 1H, H_{h3}), 14.5 (s, 2H, H_{q1}), 12.0 (s, 1H, H_r), 11.5 (s, 1H, H_{c2}), 11.0 (s, 1H, H_{c1}), 10.8 (s, 1H, H_{c3}), 4.6 (s, 3H, H_q, H_r), 1.2 (s, 1H, H_r), 0.3 (s, 4H, H_p, H_q), -0.8 (s, 1H, H_{k2}), -8.4 (s, 1H, H_{k3}), -10.5 (s, 2H, H_p), -14.0 (s, 1H, H_{l2}), -17.3 (s, 1H, H_{l3}), -18.9 (bs, H_m), -145.6 (bs, H_m), -167.8 (bs, H_m).

HRMS (ESI) *m/z*: 710.1647 [Co(pq-5'-Ph)$_2$ + BF$_4$]$^+$, 452.6380 (calculated for C$_{60}$H$_{42}$N$_6$Co: 452.6398) [Co(pq-5'-Ph)$_3$]$^{2+}$, 311.5809 [Co(pq-5'-Ph)$_2$]$^{2+}$.

3.1.4.1 Characterisation using Paramagnetic NMR Spectroscopy and Mass Spectrometry

Despite the broader linewidths in comparison to the spectrum of [Co(pq)$_3$](BF$_4$)$_2$ (Table S5, Section 3.1.8), cross-peaks were observed in the COSY and NOESY spectra of [Co(pq-Ph)$_3$](BF$_4$)$_2$ (Figures S66-S67) allowing assignment of the ^{1}H NMR spectrum (Figure S65). As well as the major species *mer*-[Co(pq-CCH)$_3$](BF$_4$)$_2$, signals attributed to a Co(pq-Ph)$_2$-based species (purple signals) were assigned from the NOESY spectrum.

Assignment of the ^{13}C NMR spectrum (Figure S68) was not possible since no cross-peaks were observed in the downfield spectrum of the HMQC spectrum and only weak cross-peaks for the complex were observed in the upfield spectrum (Figure S69).

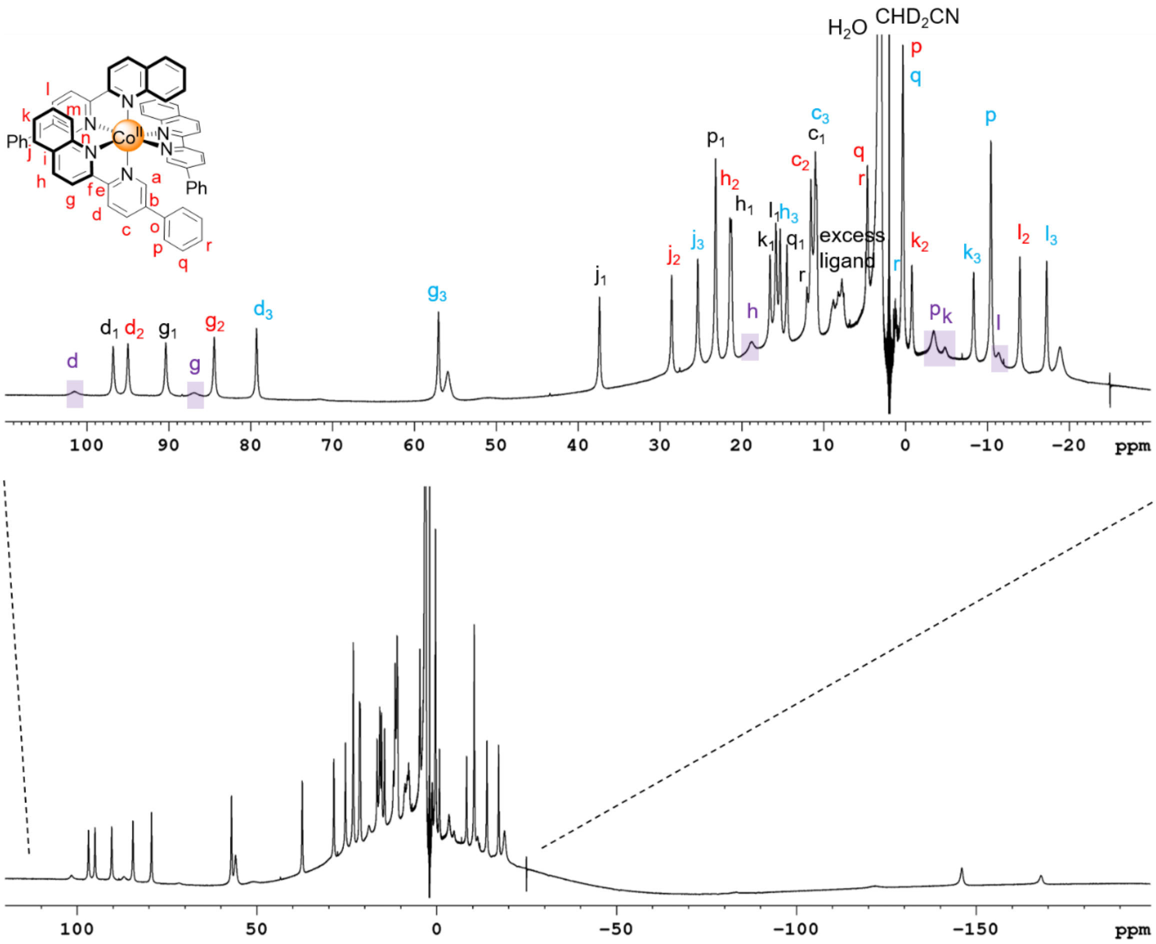

Figure S65. ^{1}H NMR spectrum (600 MHz, CD_3CN, 298 K) of $[Co(pq\text{-}5'\text{-}Ph)_3](BF_4)_2$. The signals with black, red and blue labels correspond to the three ligand environments of the major species, *mer*-$Co(pq\text{-}5'\text{-}Ph)_3$. Where present, the numbers indicate signals belonging to the same spin system as established by COSY NMR spectroscopy, however, different spin systems (i.e. protons *b*-*d* and protons *g*-*h*) could not be correlated using NOESY spectroscopy. Signals without numbers were arbitrarily labelled with black, red and blue labels according to their chemical shift. Purple signals are attributed to a $Co(pq\text{-}5'\text{-}Ph)_2$ based species.

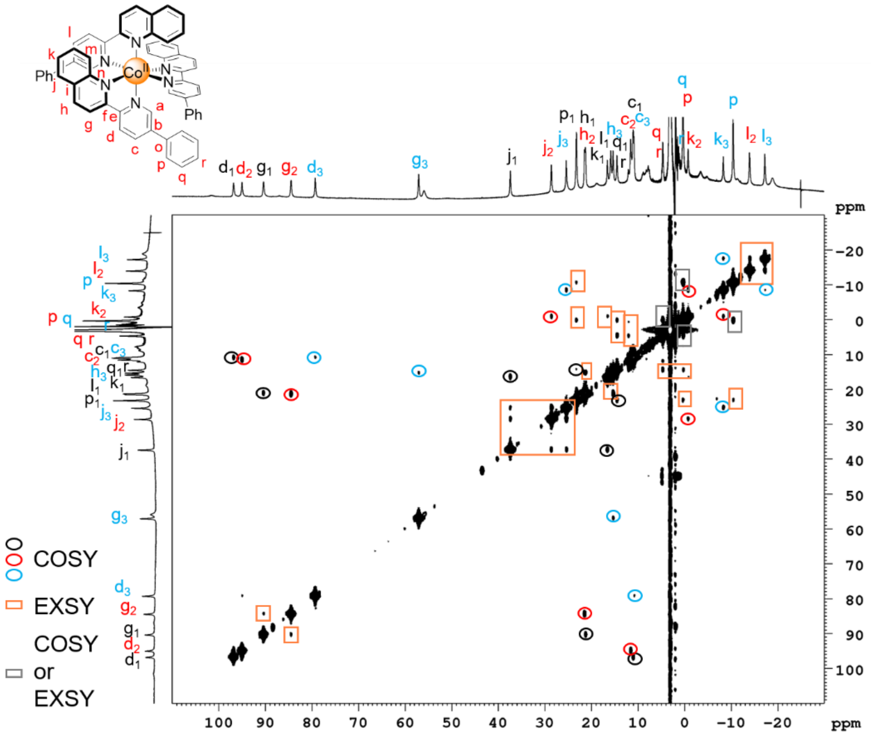

Figure S66. ^{1}H-^{1}H COSY NMR spectrum (600 MHz, CD_3CN, 298 K) of $[Co(pq\text{-}5'\text{-}Ph)_3](BF_4)_2$. The black, red and blue circles represent through-bond (COSY) cross-peaks within the three ligand environments, whereas orange squares represent exchange (EXSY) cross-peaks between the three ligand environments. Cross-peaks in grey squares could not be assigned to through-bond or exchange cross-peaks due to the overlapping peaks.

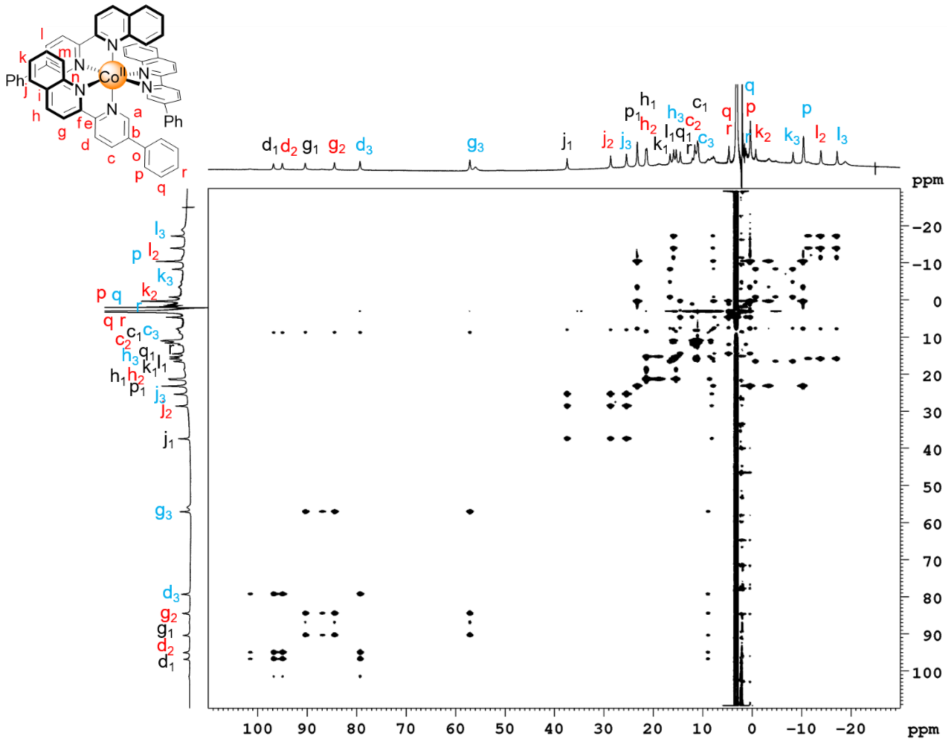

Figure S67. ^{1}H-^{1}H NOESY NMR spectrum (600 MHz, CD_3CN, 298 K) of $[Co(pq\text{-}5'\text{-}Ph)_3](BF_4)_2$.

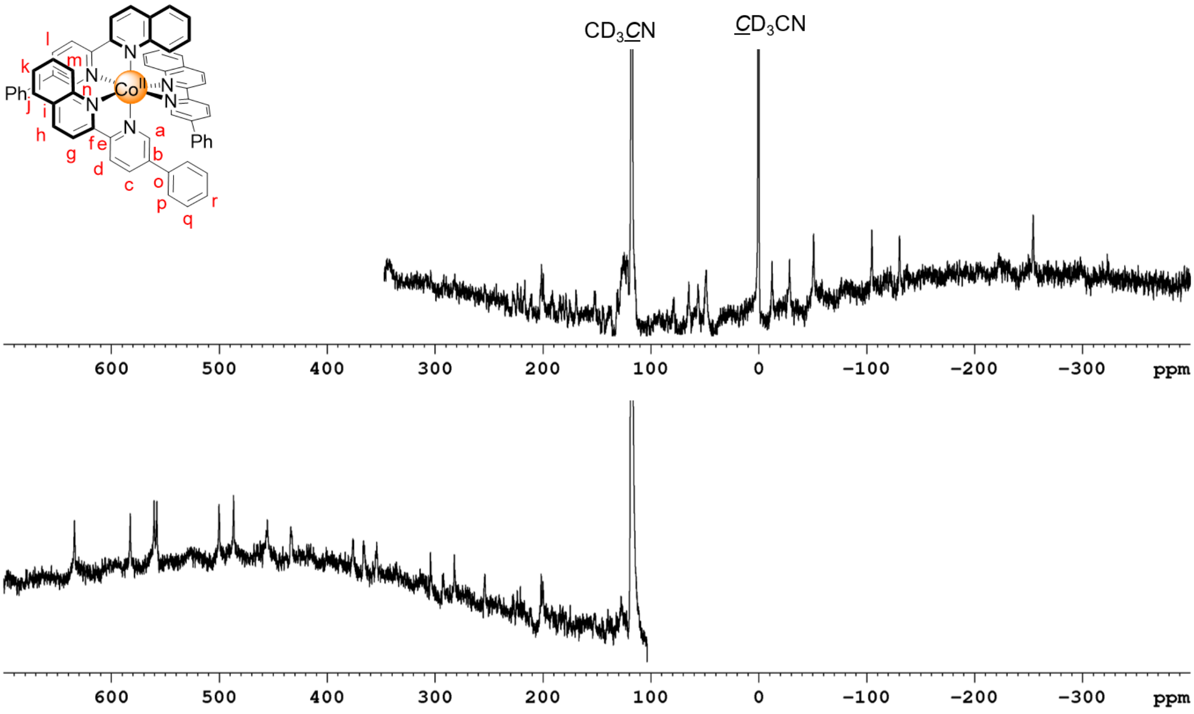

Figure S68. ^{13}C NMR spectrum (151 MHz, CD_3CN, 298 K) of $[Co(pq\text{-}5'\text{-}Ph)_3](BF_4)_2$. Assignment of the spectrum was not possible due to the absence of many cross-peaks in the HMQC spectra.

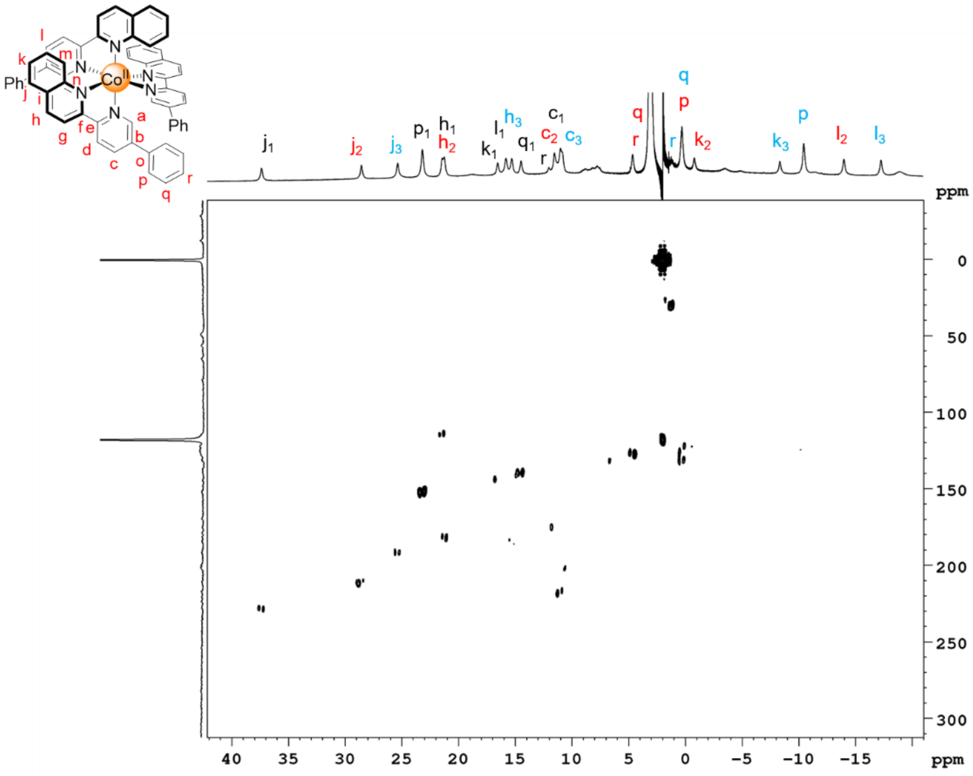

Figure S69. ^1H-^{13}C HMQC NMR spectrum (600 MHz/151 MHz, CD_3CN, 298 K) of $[Co(pq\text{-}5'\text{-}Ph)_3](BF_4)_2$.

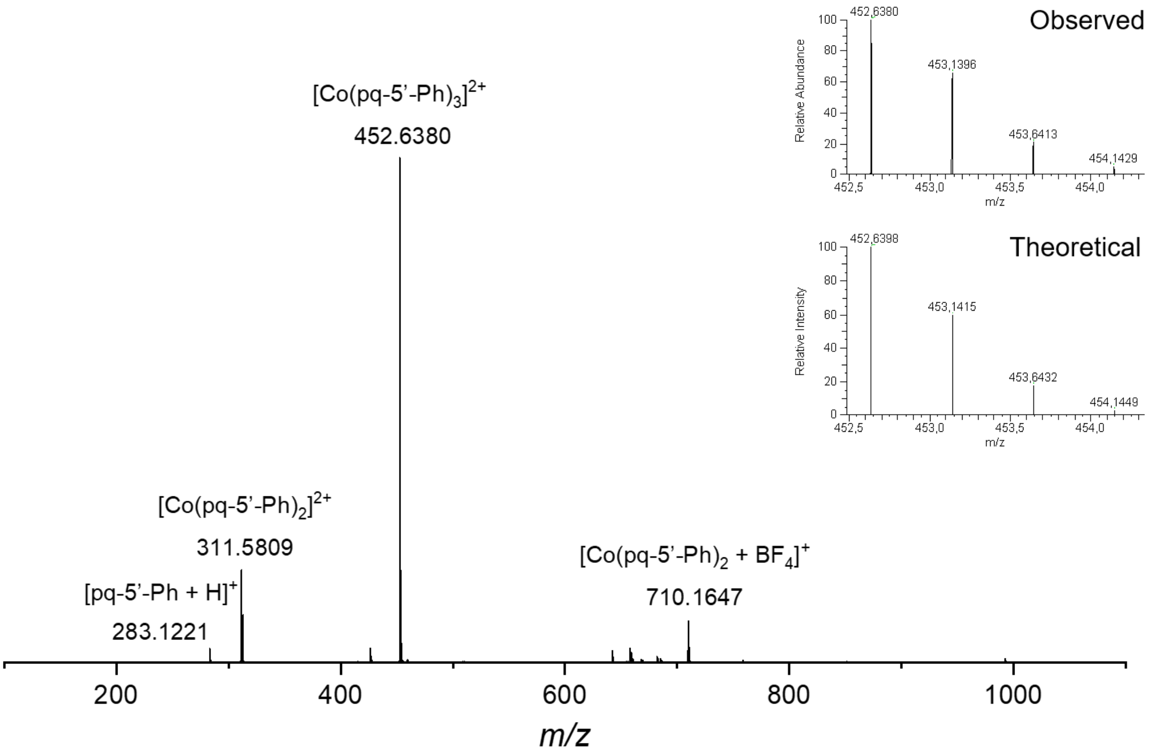

Figure S70. High resolution ESI mass spectrum of $[Co(pq\text{-}5'\text{-}Ph)_3](BF_4)_2$ showing in the inset the observed (top) and theoretical (bottom) isotope patterns.

3.1.5 *mer*-$[Co(pq\text{-}6\text{-}Br)_3](BF_4)_2$ (**5a**)

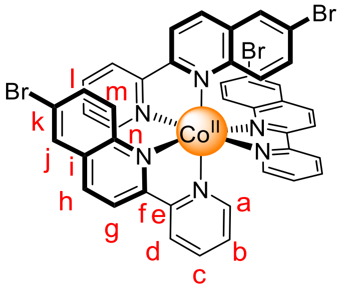

$Co(BF_4)_2{\bullet}6H_2O$ (3.02 mg, 8.87 µmol) and 6-bromo-2-(pyridin-2'-yl)quinoline (7.53 mg, 26.5 µmol) and were dissolved in CD_3CN (0.5 mL) and the solution was heated at 50 °C for 24 h.

^{1}H NMR (600 MHz, CD_3CN, 298 K) δ (ppm): 98.4 (s, 1H, H_{d1}), 90.3 (s, 1H, H_{d2}), 86.4 (s, 1H, H_{d3}), 84.1 (s, 1H, H_{g1}), 78.8 (s, 1H, H_{g2}), 69.1 (s, 1H, H_{b3}), 65.3 (bs, H_a), 58.4 (s, 1H, H_{g3}), 51.1 (s, 1H, H_{b1}), 41.3 (s, 1H, H_{b2}), 34.2 (s, 1H, H_j), 25.2 (s, 1H, H_j), 23.7 (s, 1H, H_j), 19.1 (s, 1H, H_{h2}), 16.7 (s, 1H, H_{h1}), 14.7 (s, 1H, H_l), 14.1 (2H, H_{c3}, H_{h3}), 12.4 (s, 1H, H_{c1}), 9.7 (s, 1H, H_{c2}), -11.5 (s, 1H, H_l), -15.6 (s, 1H, H_l), -21.4 (bs, H_m), -135.3 (bs, H_m), -163.1 (bs, H_m).

HRMS (ESI) *m/z*: 457.4552 (calculated for $C_{42}H_{27}N_6Br_3Co$: 457.4564) $[Co(pq\text{-}6\text{-}Br)_3]^{2+}$, 314.4592 $[Co(pq\text{-}6\text{-}Br)_2]^{2+}$.

3.1.5.1 Characterisation using Paramagnetic NMR Spectroscopy and Mass Spectrometry

Only ^{1}H NMR data assigned to the *mer* isomer are reported above. The ^{13}C NMR data are not reported since it was not possible to fully assign the spectrum (Figure S74) from the HMQC spectra (Figures S75-S76).

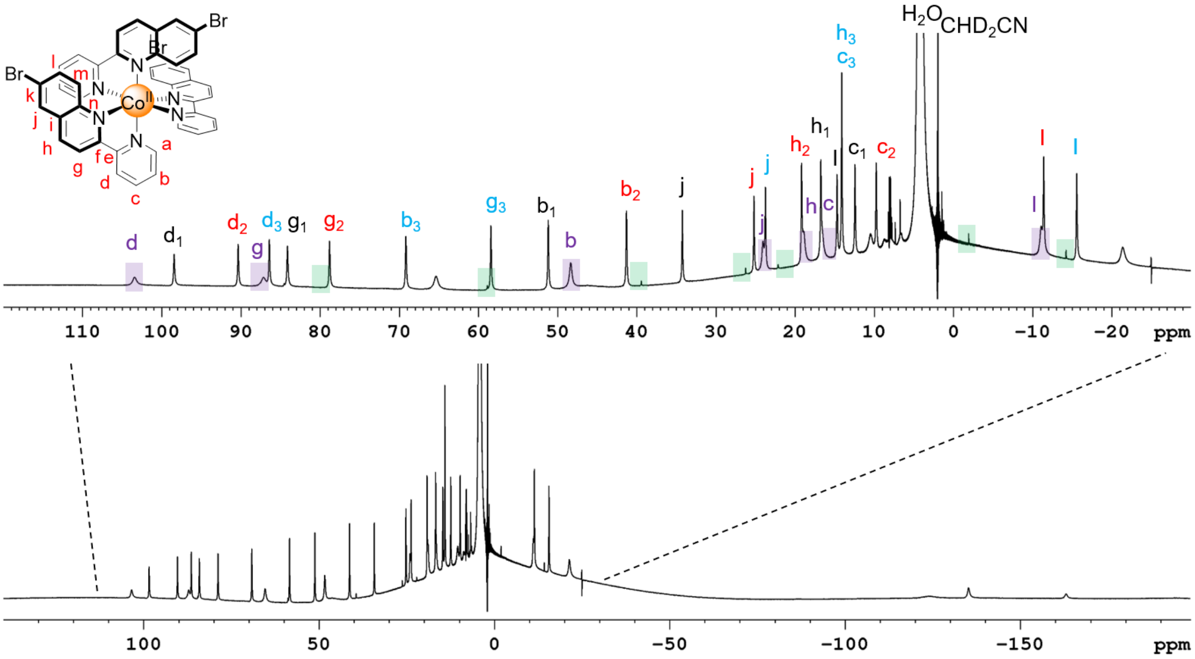

Figure S71. ^{1}H NMR spectrum (600 MHz, CD_3CN, 298 K) of $[Co(pq\text{-}6\text{-}Br)_3](BF_4)_2$. The signals with black, red and blue labels correspond to the three ligand environments of the major species, *mer*-$Co(pq\text{-}6\text{-}Br)_3$. Where present, the numbers indicate signals belonging to the same spin system as established by COSY NMR spectroscopy, however, different spin systems (i.e. protons *b-d* and *g-h*) could not be correlated to a particular ligand environment using NOESY spectroscopy. Therefore, the three sets of signals were arbitrarily labelled with black, red and blue labels according to their decreasing chemical shift for protons *d*, *g*, *j* and *l*. Purple signals are attributed to a $Co(pq\text{-}6\text{-}Br)_2$ based species whereas the green signals are attributed to *fac*-$Co(pq\text{-}6\text{-}Br)_3$.

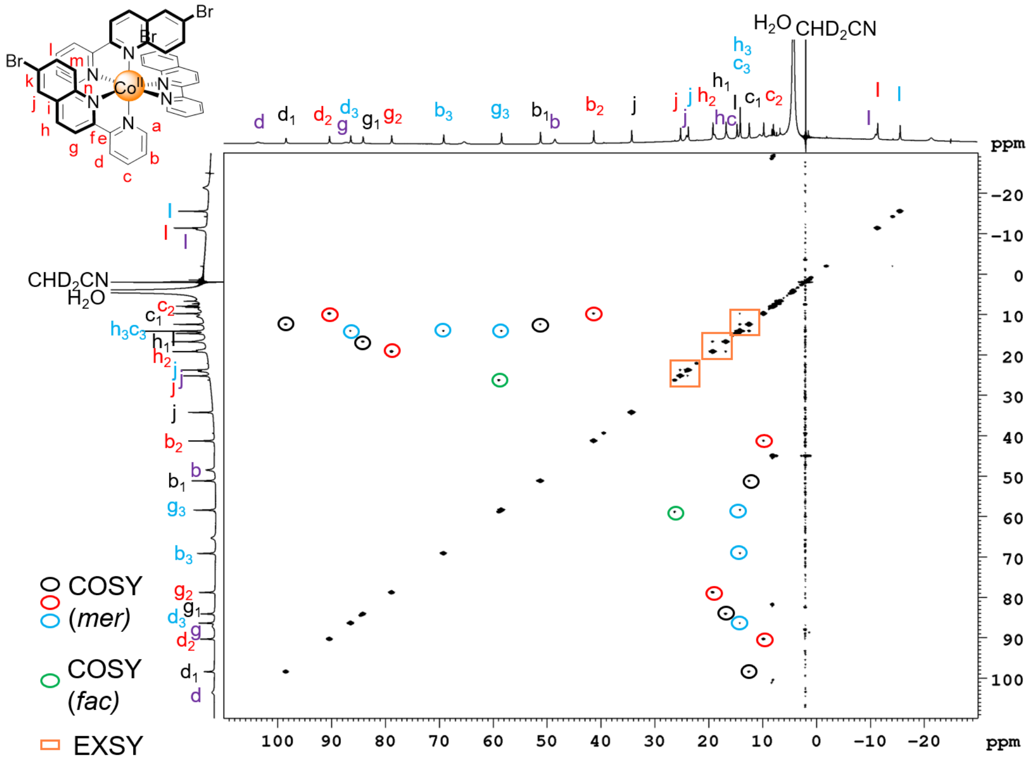

Figure S72. ^{1}H-^{1}H COSY NMR spectrum (600 MHz, CD_3CN, 298 K) of $[Co(pq\text{-}6\text{-}Br)_3](BF_4)_2$. Circles represent through-bond cross-peaks for the *mer* isomer (black, red blue for the three different ligand environments) and *fac* isomer (green) whereas orange squares represent exchange cross-peaks.

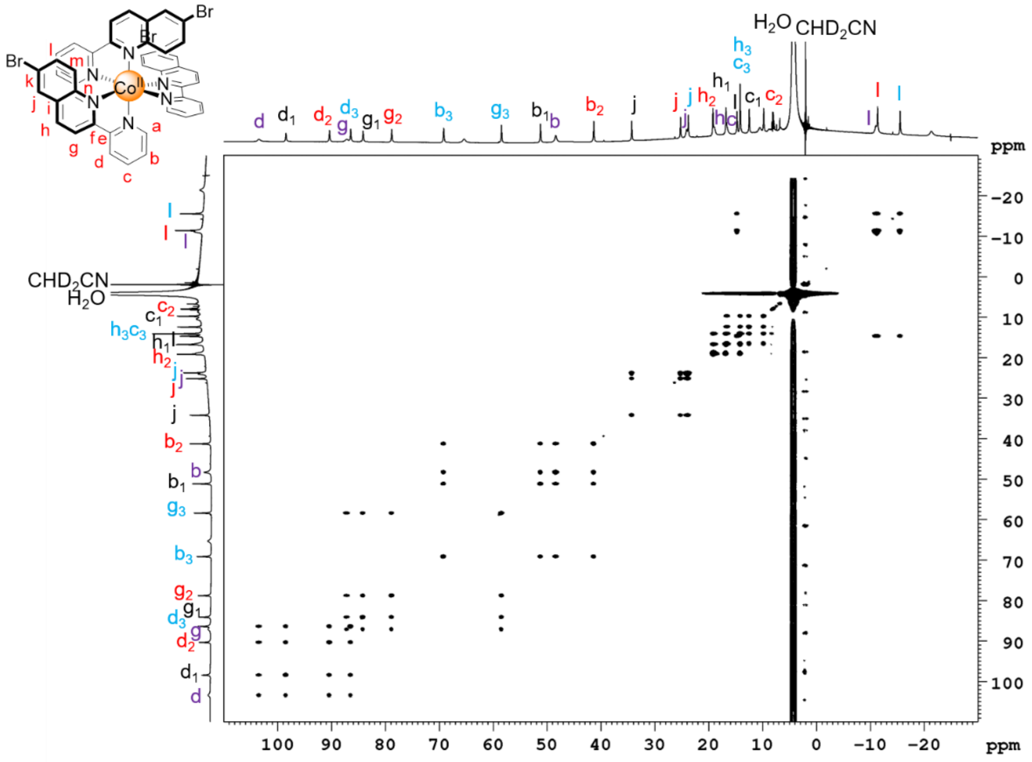

Figure S73. ^{1}H-^{1}H NOESY NMR spectrum (600 MHz, CD_3CN, 298 K) of $[Co(pq\text{-}6\text{-}Br)_3](BF_4)_2$.

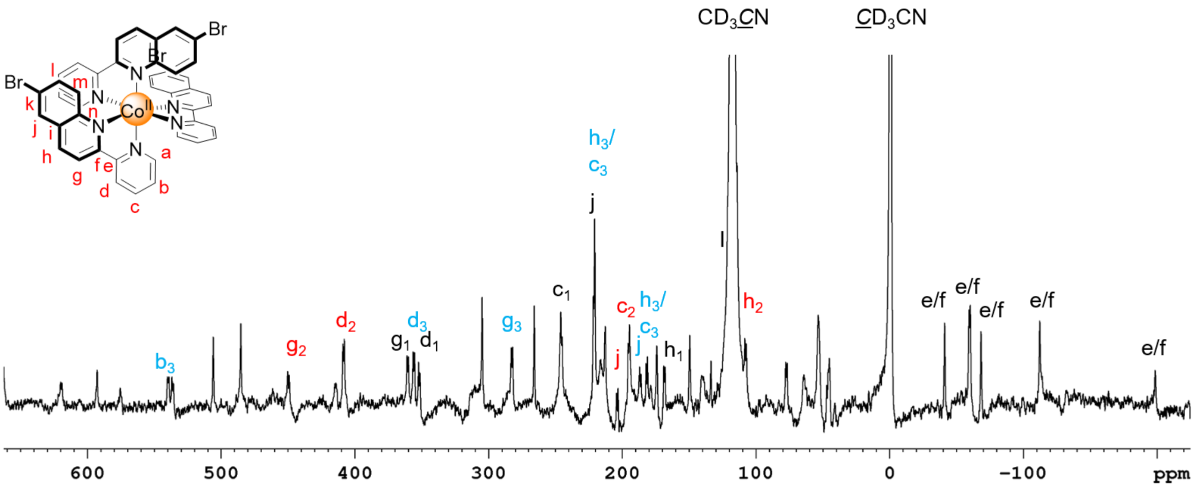

Figure S74. ^{13}C NMR spectrum (151 MHz, CD_3CN, 298 K) of $[Co(pq\text{-}6\text{-}Br)_3](BF_4)_2$. Only partial assignment of the spectrum was possible due to the absence of some cross-peaks in the HMQC spectra and the number of overlapping signals from the four species present (*mer*-$Co(pq\text{-}6\text{-}Br)_3$, *fac*-$Co(pq\text{-}6\text{-}Br)_3$, a $Co(pq\text{-}6\text{-}Br)_2$ based species and free ligand).

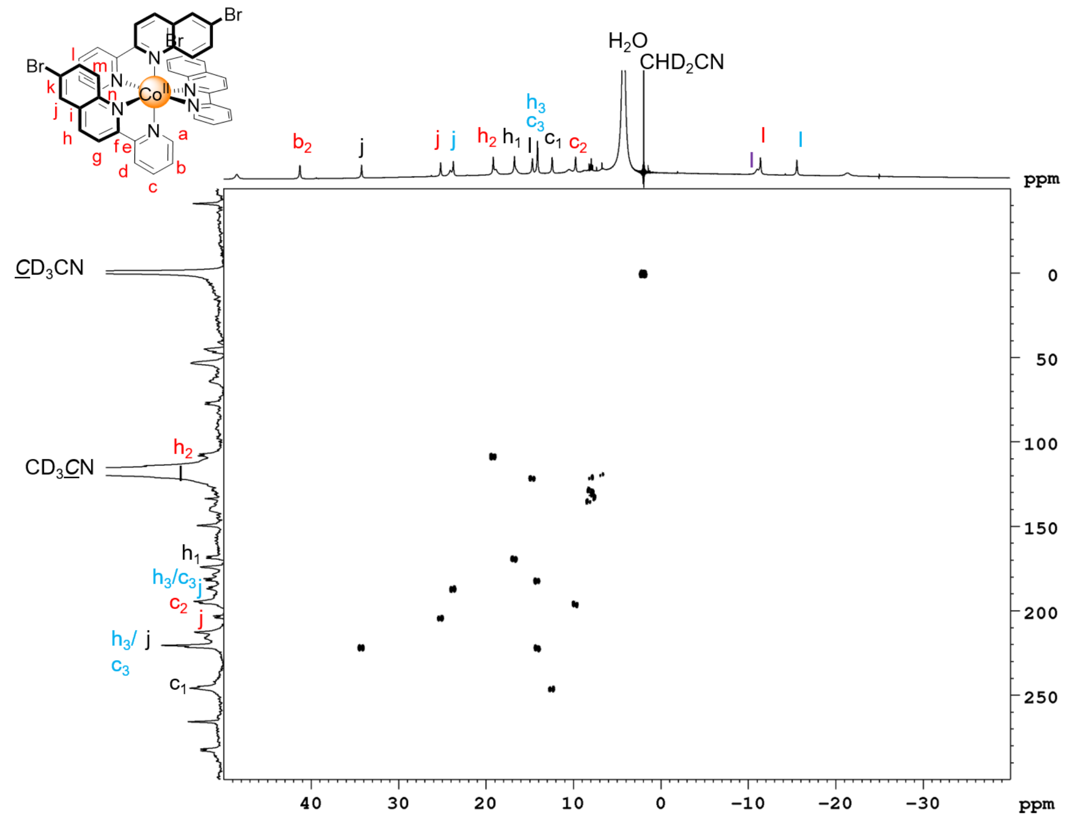

Figure S75. ^{1}H-^{13}C HMQC NMR spectrum (600 MHz/151 MHz, CD_3CN, 298 K) of $[Co(pq\text{-}6\text{-}Br)_3](BF_4)_2$.

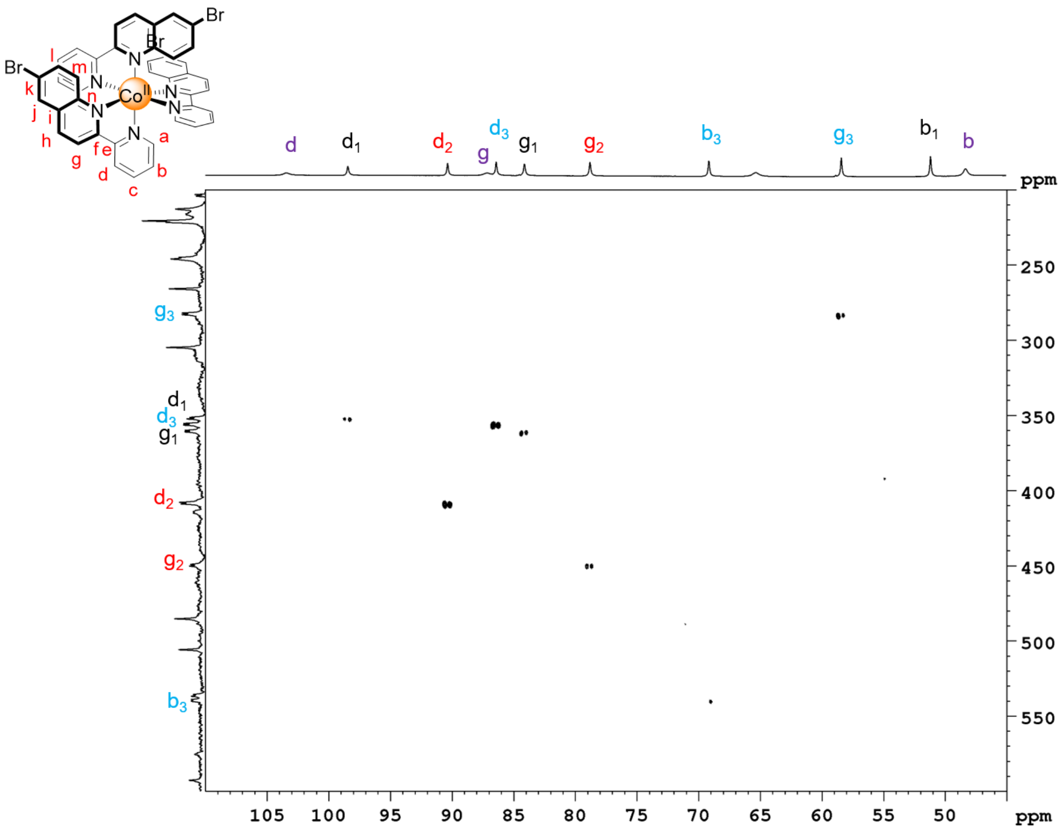

Figure S76. ^{1}H-^{13}C HMQC NMR spectrum (600 MHz/151 MHz, CD_3CN, 298 K) of $[Co(pq\text{-}6\text{-}Br)_3](BF_4)_2$.

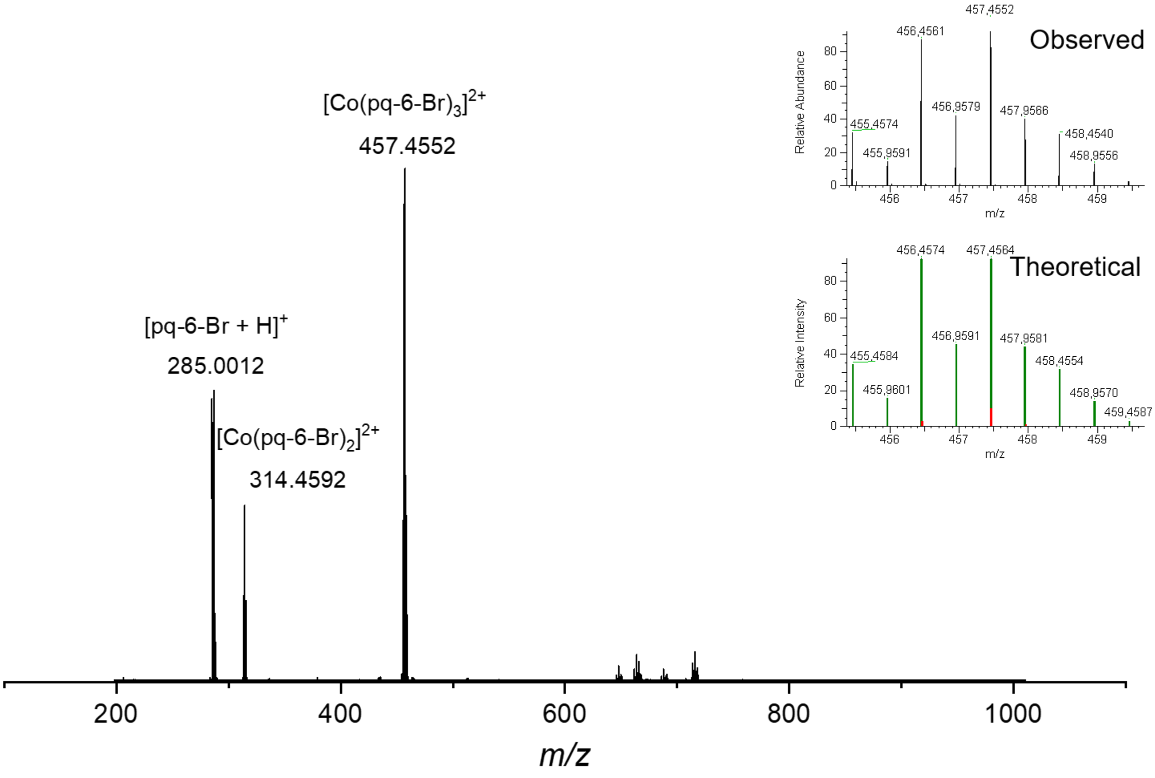

Figure S77. High resolution ESI mass spectrum of $[Co(pq\text{-}6\text{-}Br)_3](BF_4)_2$ showing in the inset the observed (top) and theoretical (bottom) isotope patterns.

3.1.6 *mer*-[Co(pq-6-CCH)$_3$](BF$_4$)$_2$ (**6a**)

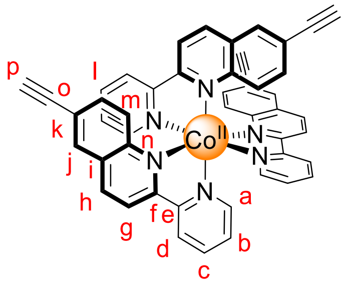

Co(BF$_4$)$_2$•6H$_2$O (3.02 mg, 8.81 µmol) and 6-ethynyl-2-(pyridin-2'-yl)quinoline (6.08 mg, 26.4 µmol) were dissolved in CD$_3$CN (0.5 mL) and the solution was heated at 50 °C for 24 h.

^{1}H NMR (600 MHz, CD$_3$CN, 298 K) δ (ppm): 98.1 (s, 1H, H_{d1}), 90.7 (s, 1H, H_{d2}), 85.6 (s, 1H, H_{d3}), 84.6 (s, 1H, H_{g1}), 79.7 (s, 1H, H_{g2}), 68.8 (s, 1H, H_{b3}), 64.3 (bs, H_a), 58.3 (s, 1H, H_{g3}), 50.5 (s, 1H, H_{b1}), 41.1 (s, 1H, H_{b2}), 35.3 (s, 1H, H_j), 26.1 (s, 1H, H_j), 24.7 (s, 1H, H_j), 19.6 (s, 1H, H_{h2}), 17.2 (s, 1H, H_{h1}), 14.5 (s, 1H, H_{h3}), 14.4 (s, 1H, H_l), 13.6 (s, 1H, H_{c3}), 12.3 (s, 1H, H_{c1}), 10.0 (s, 1H, H_{c2}), 6.7 (s, 1H, H_p), 0.1 (s, 1H, H_p), -2.7 (s, 1H, H_p), -11.9 (s, 1H, H_l), -15.8 (s, 1H, H_l), -22.2 (bs, H_m), -136.4 (bs, H_m), -163.5 (bs, H_m).

^{13}C NMR (151 MHz, CD$_3$CN, 298 K) δ (ppm): 620.8 (d, ^1J = 165 Hz, C_{b2}), 588.3 (s, $C_{i/n}$), 541.5 (d, ^1J = 160 Hz, C_{b3}), 538.6 (d, ^1J = 182 Hz, C_{b1}), 502.7 (s, $C_{i/n}$), 484.4 (s, $C_{i/n}$), 452.8 (d, ^1J = 176 Hz, C_{g2}), 411.5 (d, ^1J = 161 Hz, C_{d2}), 362.3 (d, ^1J = 154 Hz, C_{g1}), 357.3 (d, ^1J = 166 Hz, C_{d3}), 355.1 (d, ^1J = 172 Hz, C_{d1}), 307.3 (s, $C_{i/n}$), 285.6 (d, ^1J = 164 Hz, C_{g3}), 269.3 (s, $C_{i/n}$), 244.9 (d, ^1J = 158 Hz, C_{c1}), 225.5 (d, ^1J = 174 Hz, C_j), 223.3 (s, $C_{i/n}$), 221.4 (d, ^1J = 162 Hz, C_{c3}), 216.5 (s), 208.6 (d, ^1J = 160 Hz, C_j), 196.2 (d, ^1J = 161 Hz, C_{c2}), 190.1 (d, ^1J = 162 Hz, C_j), 183.1 (d, ^1J = 177 Hz, C_{h3}), 177.1 (s), 171.4 (d, ^1J = 160 Hz, C_{h1}), 121.9 (unresolved d, C_l), 110.5 (d, ^1J = 172 Hz, C_{h2}), 87.5 (unresolved d, C_p), 79.3 (C_l, 2 x C_p), 53.6 (s, C_a), 50.3 (s, C_a), 48.2 (d, ^1J = 170 Hz, C_l), 42.7 (s, C_a), -36.6 (s, $C_{e/f}$), -51.3 (s, $C_{e/f}$), -55.9 (s, $C_{e/f}$), -70.9 (s, $C_{e/f}$), -113.4 (s, $C_{e/f}$), -202.6 (s, $C_{e/f}$).

Note: not all quaternary carbons are reported above as they could not be distinguished from excess ligand carbon signals and carbons *m* could not be assigned.

HRMS (ESI) *m/z*: 374.5923 (calculated for $C_{48}H_{30}N_6Co$: 374.5926) [Co(pq-6-CCH)$_3$]$^{2+}$, 259.5497 [Co(pq-6-CCH)$_2$]$^{2+}$.

3.1.6.1 Characterisation using Paramagnetic NMR Spectroscopy and Mass Spectrometry

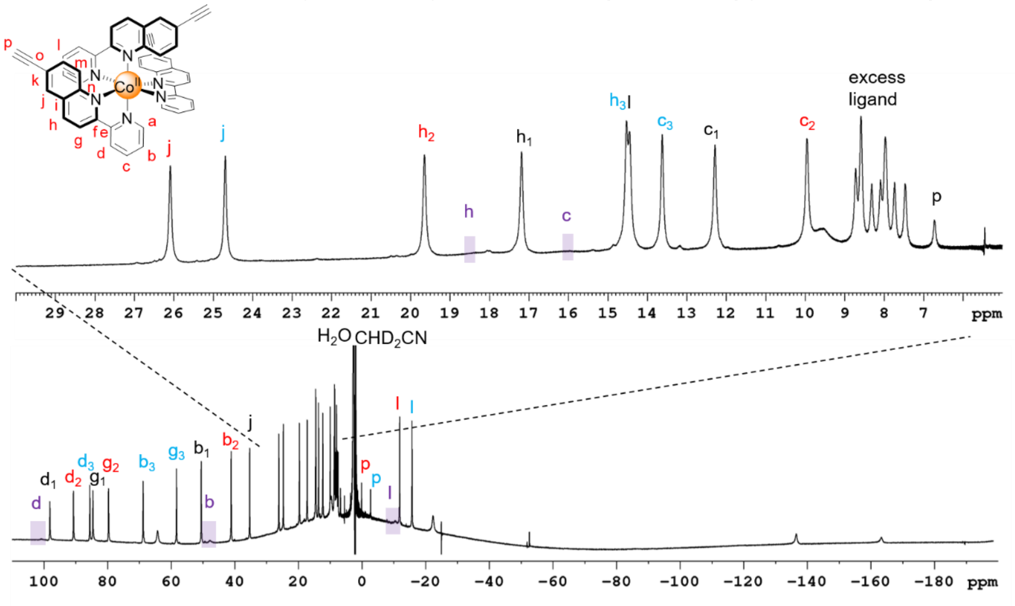

Figure S78. ^{1}H NMR spectrum (600 MHz, CD_3CN, 298 K) of $[Co(pq\text{-}6\text{-}CCH)_3](BF_4)_2$. The signals with black, red and blue labels correspond to the three ligand environments of the major species, *mer*-Co(pq-6-CCH)$_3$. Where present, the numbers indicate signals belonging to the same spin system as established by COSY NMR spectroscopy, however, different spin systems (ie protons *b-d*, *g-h* and *p*) could not be correlated to a particular ligand environment using NOESY spectroscopy. Therefore, the three sets of signals were arbitrarily labelled with black, red and blue labels according to their decreasing chemical shift. Purple signals are attributed to a Co(pq-6-CCH)$_2$ based species.

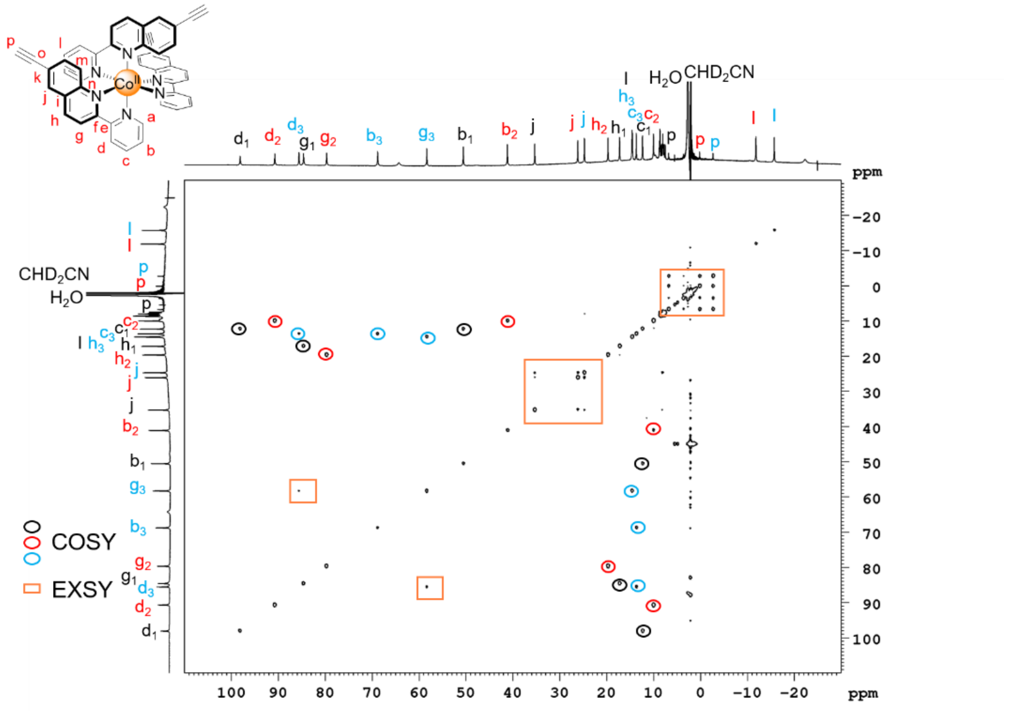

Figure S79. ^{1}H-^{1}H COSY NMR spectrum (600 MHz, CD_3CN, 298 K) of $[Co(pq\text{-}6\text{-}CCH)_3](BF_4)_2$. Black, red and blue circles represent through-bond (COSY) cross-peaks peaks within the three ligand environments whereas orange squares represent exchange (EXSY) cross-peaks between the three ligand environments.

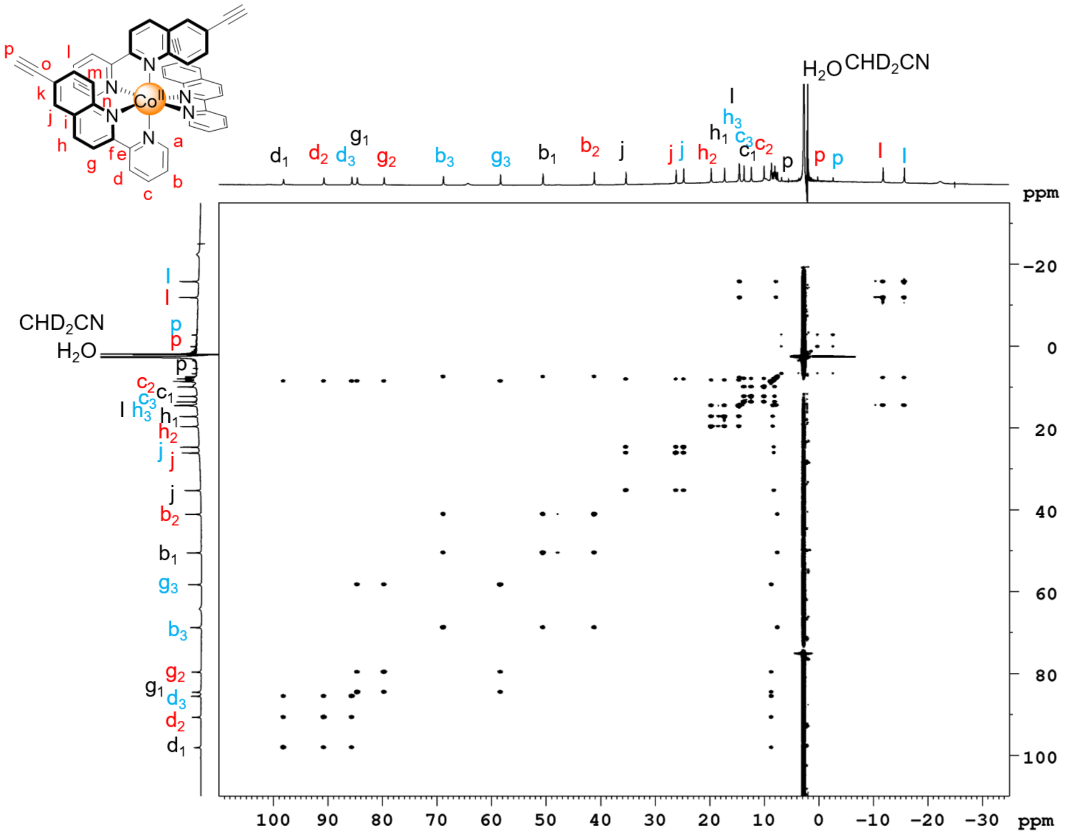

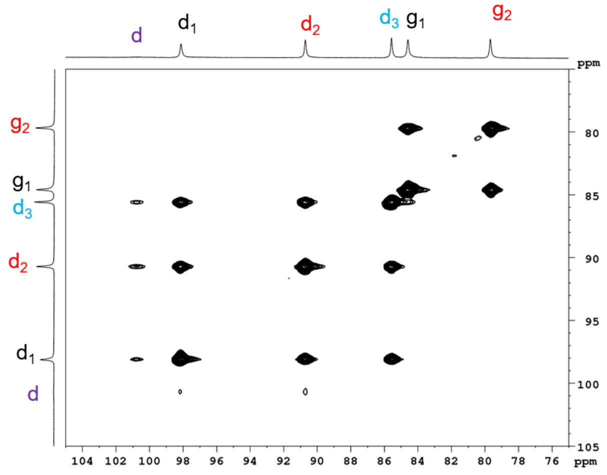

Figure S80. ^1H-^1H NOESY NMR spectrum (600 MHz, CD_3CN, 298 K) of $[Co(pq\text{-}6\text{-}CCH)_3](BF_4)_2$ with inset (below) showing exchange cross-peaks to a second species (purple label), proposed to be a $Co(pq\text{-}6\text{-}CCH)_2$-based species.

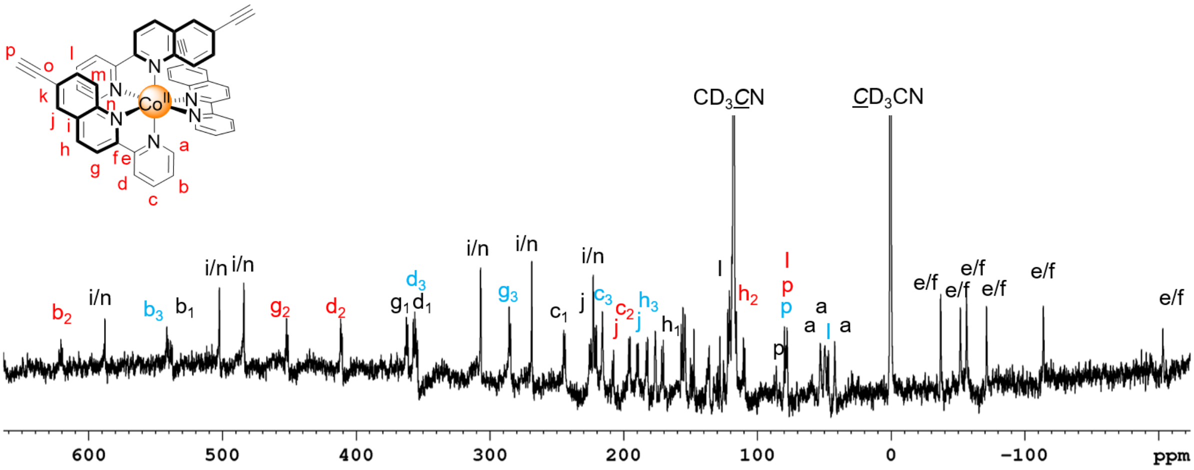

Figure S81. ^{13}C NMR spectrum (151 MHz, CD_3CN, 298 K) of $[Co(pq\text{-}6\text{-}CCH)_3](BF_4)_2$. Note: carbons *k*, *m* and *o* could not be assigned.

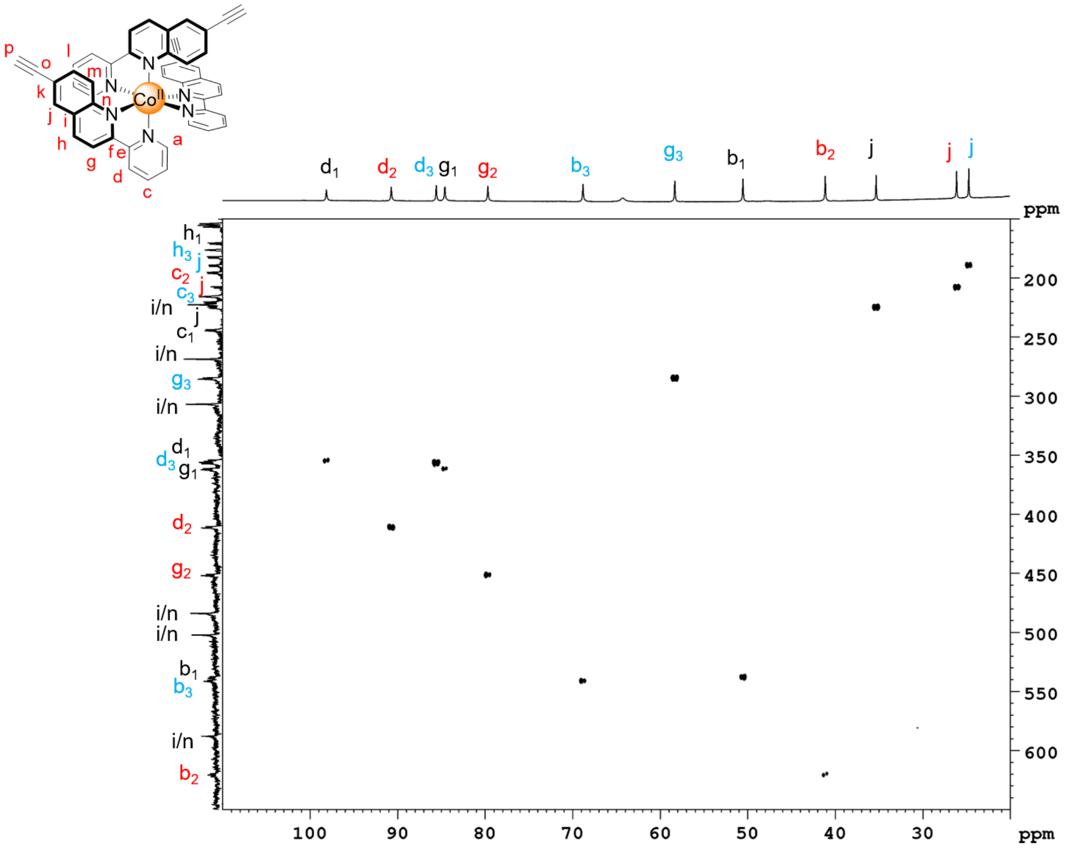

Figure S82. ^{1}H-^{13}C HMQC NMR spectrum (600 MHz/151 MHz, CD_3CN, 298 K) of $[Co(pq\text{-}6\text{-}CCH)_3](BF_4)_2$.

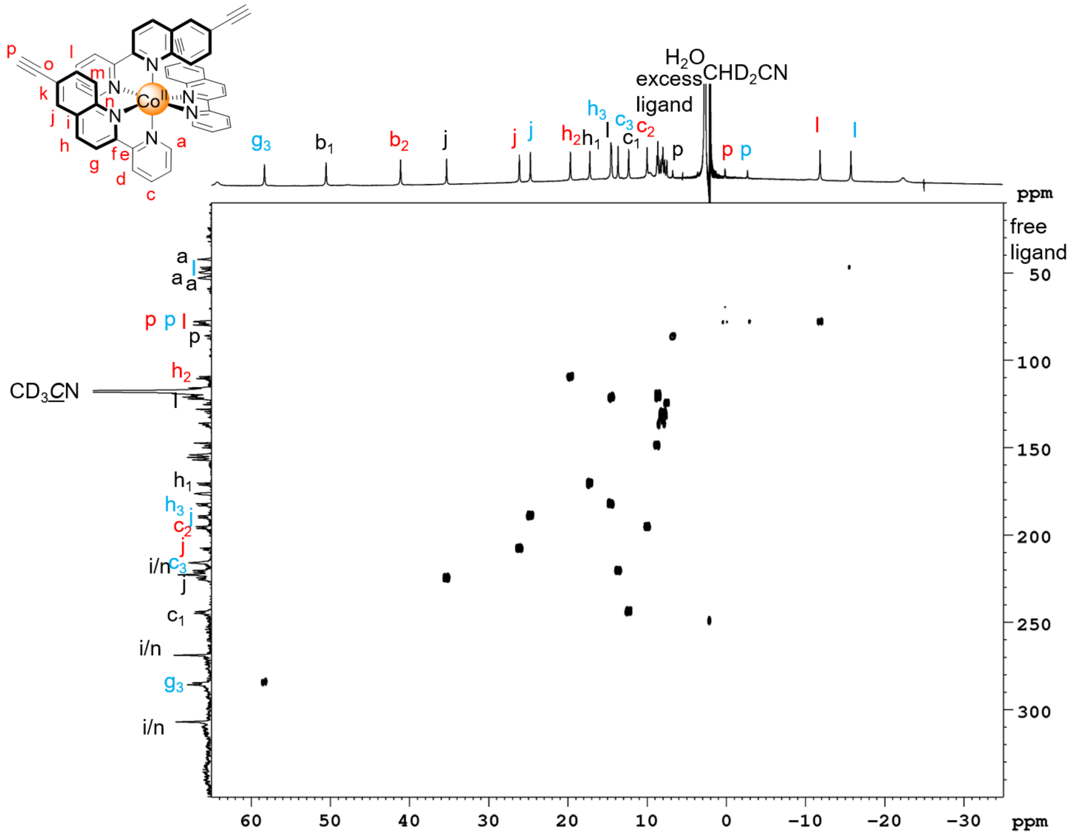

Figure S83. ^{1}H-^{13}C HMQC NMR spectrum (600 MHz/151 MHz, CD_3CN, 298 K) of $[Co(pq\text{-}6\text{-}CCH)_3](BF_4)_2$.

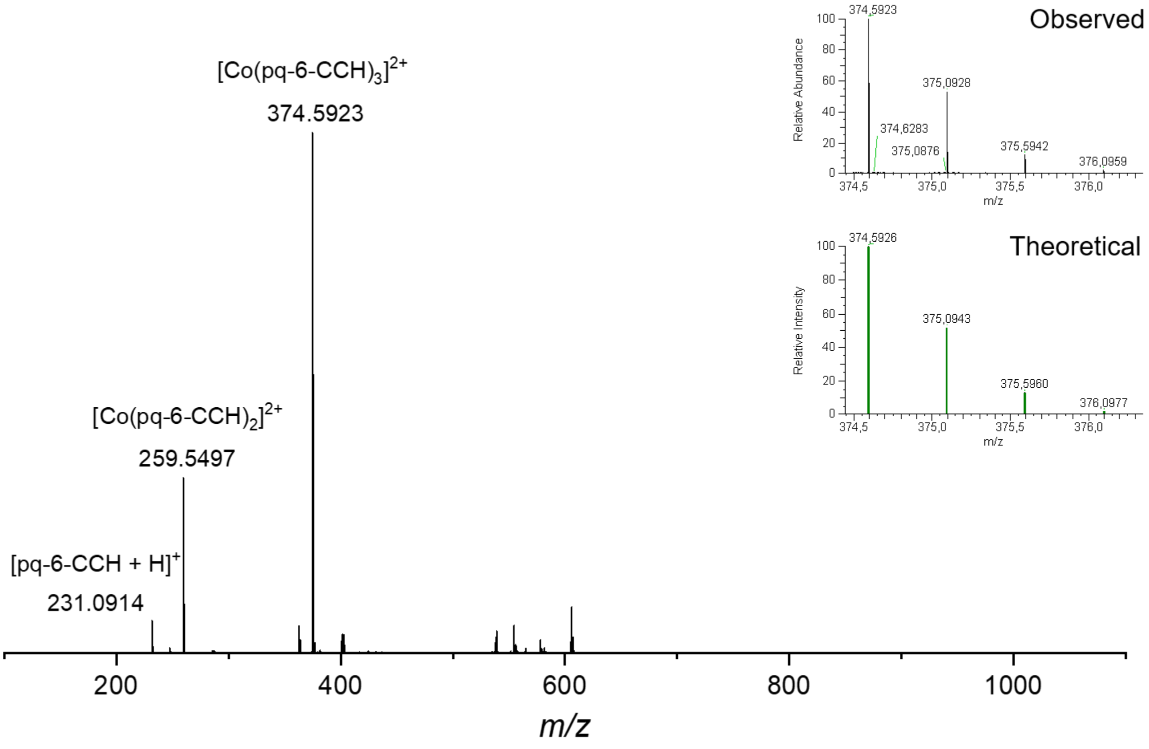

Figure S84. High resolution ESI mass spectrum of $[Co(pq\text{-}6\text{-}CCH)_3](BF_4)_2$ showing in the inset the observed (top) and theoretical (bottom) isotope patterns.

3.1.7 *mer*-[Co(pq-6-Ph)$_3$](BF$_4$)$_2$ (**7a**)

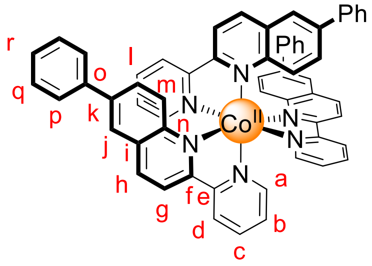

Co(BF$_4$)$_2$•6H$_2$O (3.06 mg, 8.98 µmol) and 6-phenyl-2-(pyridin-2'-yl)quinoline (7.45 mg, 26.4 µmol) were dissolved in CD$_3$CN (0.5 mL) and the solution was equilibrated at room temperature.

^{1}H NMR (600 MHz, CD$_3$CN, 298 K) δ (ppm): 97.2 (s, 1H, H_{d1}), 91.7 (s, 1H, H_{d2}), 86.8 (s, 1H, H_{g1}), 83.0 (s, 1H, H_{d3}), 82.8 (s, 1H, H_{g2}), 67.9 (s, 1H, H_{b3}), 59.5 (bs, H_a), 58.3 (s, 1H, H_{g3}), 48.7 (s, 1H, H_{b1}), 40.7 (s, 1H, H_{b2}), 36.4 (s, 1H, H_j), 27.6 (s, 1H, H_j), 25.8 (s, 1H, H_j), 21.1 (s, 1H, H_{h2}), 18.5 (s, 1H, H_{h1}), 15.4 (s, 1H, H_l), 14.7 (s, 1H, H_{h3}), 13.3 (s, 2H, H_{p1}), 12.7 (s, 1H, H_{c3}), 12.0 (s, 1H, H_{c1}), 10.4 (s, 1H, H_{c2}), 10.3 (s, 2H, H_{q1}), 9.7 (s, 1H, H_r), 5.6 (s, 1H, H_r), 5.3 (s, 2H, H_{q2}), 3.9 (s, 1H, H_r), 3.5 (s, 2H, H_{q3}), 2.7 (s, 2H, H_{p2}), -1.0 (s, 2H, H_{p3}), -12.1 (s, 1H, H_l), -15.6 (s, 1H, H_l), -21.0 (bs, H_m), -138.0 (bs, H_m), -165.2 (bs, H_m).

^{13}C NMR (151 MHz, CD$_3$CN, 298 K) δ (ppm): 619.3 (d, ^1J = 154 Hz, C_{b2}), 587.5 (s, $C_{i/n}$), 545.0 (d, ^1J = 154 Hz, C_{b3}), 541.9 (d, ^1J = 174 Hz, C_{b1}), 498.4 (s, $C_{i/n}$), 483.3 (s, $C_{i/n}$), 455.9 (d, ^1J = 164 Hz, C_{g2}), 415.2 (d, ^1J = 170 Hz, C_{d2}), 365.6 (d, ^1J = 134 Hz, C_{g1}), 356.7 (d, ^1J = 146 Hz, C_{d1}), 354.2 (d, ^1J = 176 Hz, C_{d3}), 304.4 (s, $C_{i/n}$), 288.4 (d, ^1J = 176 Hz, C_{g3}), 272.4 (s, $C_{i/n}$), 238.8 (d, ^1J = 164 Hz, C_{c1}), 230.7 (s, $C_{i/n}$), 221.3 (d, ^1J = 134 Hz, C_j), 217.1 (unresolved d, C_{c3}), 204.2 (d, ^1J = 146 Hz, C_j),194.3 (d, ^1J = 170 Hz, C_{c2}), 185.1 (overlapping d, C_j, C_{h3}), 177.1 (d, ^1J = 164 Hz, C_{h1}), 173.8, 161.1, 154.7, 145.2, 137.9, 133.6 (unresolved d, C_{p1}), 132.4 (unresolved d, C_{q1}), 132.2 (unresolved d, C_r), 126.4 (C_{q2}, 2 x C_r), 124.7 (unresolved d, C_{p2}), 123.5 (unresolved d, C_{q3}), 122.9 (unresolved d, C_{p3}), 120.3 (unresolved d, C_l), 114.5 (d, ^1J = 162 Hz, C_{h2}), 77.0 (d, ^1J = 160 Hz, C_l), 46.7 (C_l, C_a), 43.8 (s, C_a), 38.7 (s, C_a), -23.7 (s, $C_{e/f}$), -38.4 (s, $C_{e/f}$), -49.1 (s, $C_{e/f}$), -83.8 (s, $C_{e/f}$), -115.2 (s, $C_{e/f}$), -220.0 (s, $C_{e/f}$).

Note: not all quaternary carbons and carbons *m* are reported and/or assigned above.

HRMS (ESI) *m/z*: 710.1656 [Co(pq-6-Ph)$_2$ + BF$_4$]$^+$, 452.6386 (calculated for $C_{60}H_{42}N_6Co$: 452.6396) [Co(pq-6-Ph)$_3$]$^{2+}$, 311.5812 [Co(pq-6-Ph)$_2$]$^{2+}$.

3.1.7.1 Characterisation using Paramagnetic NMR Spectroscopy and Mass Spectrometry

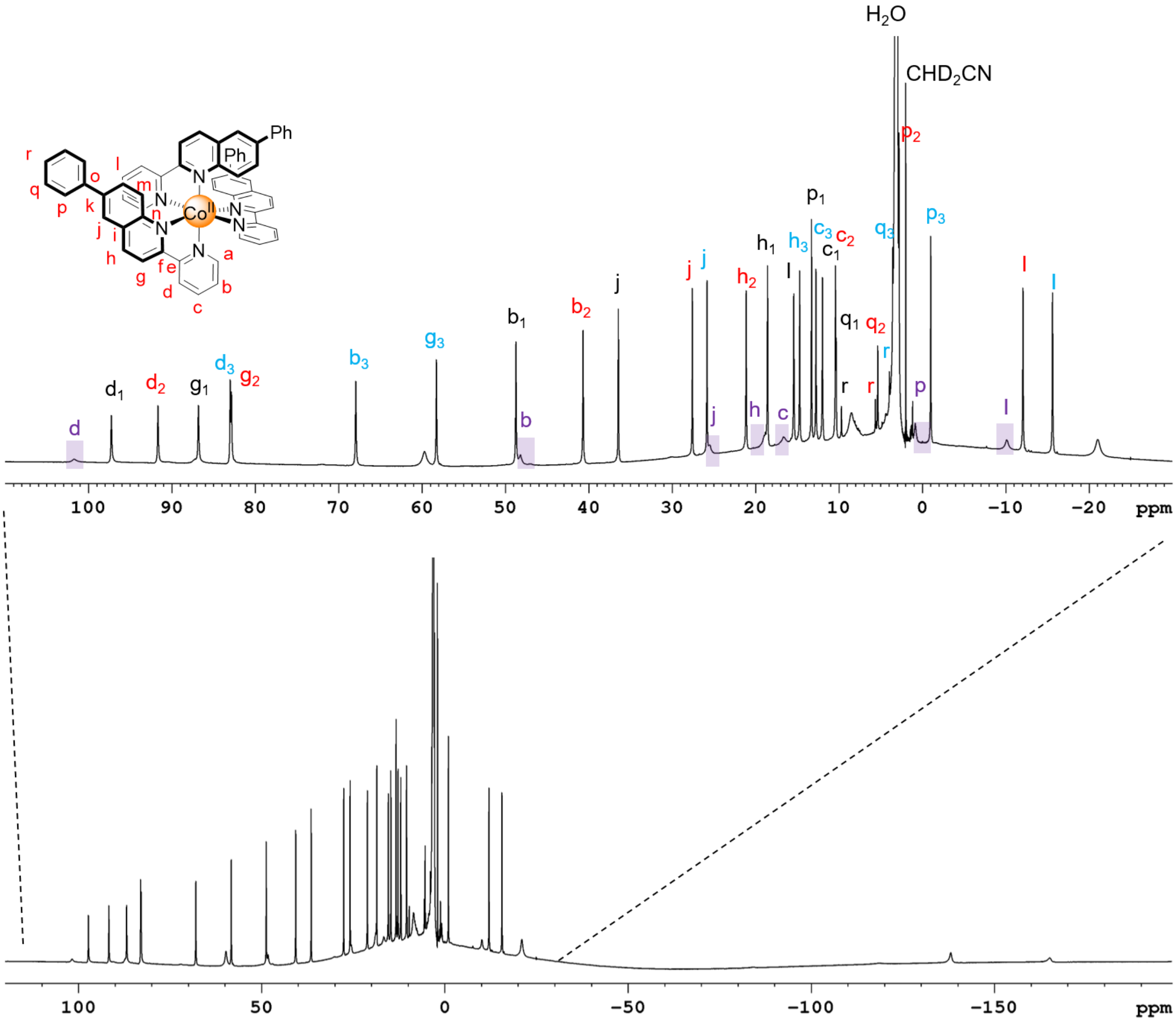

Figure S85. ^{1}H NMR spectrum (600 MHz, CD_3CN, 298 K) of $[Co(pq\text{-}6\text{-}Ph)_3](BF_4)_2$. The signals with black, red and blue labels correspond to the three ligand environments of the major species, *mer*-$Co(pq\text{-}6\text{-}Ph)_3$. Where present, the numbers indicate signals belonging to the same spin system as established by COSY NMR spectroscopy, however, different spin systems (i.e. protons *b*-*d*, *g*-*h* and *p*-*r*) could not be correlated to a particular ligand environment using NOESY spectroscopy. Therefore, the three sets of signals were arbitrarily labelled with black, red and blue labels according to their decreasing chemical shift for protons *d*, *g*, *j* and *l*. Purple signals are attributed to a $Co(pq\text{-}6\text{-}Ph)_2$ based species.

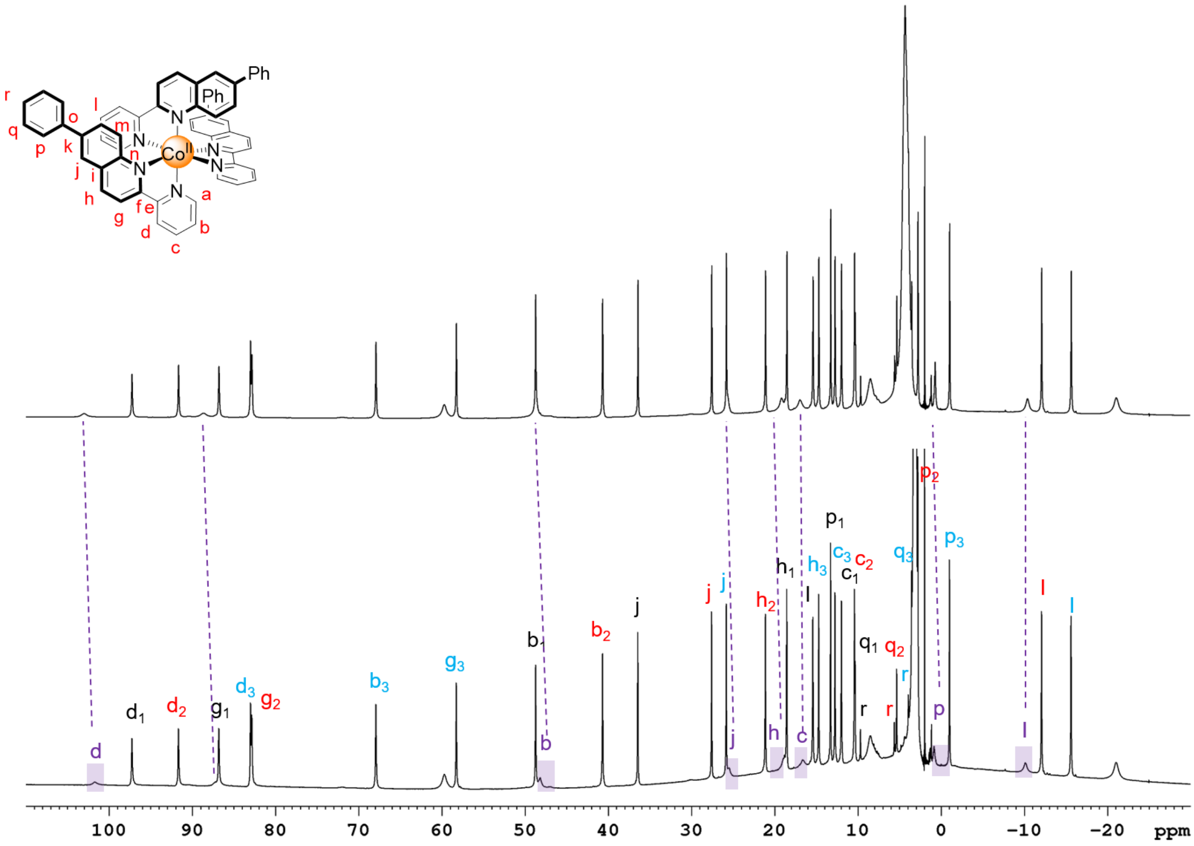

Figure S86. Comparison of ^{1}H NMR spectra (600 MHz, CD_3CN, 298 K) of two samples of $[Co(pq\text{-}6\text{-}Ph)_3](BF_4)_2$ with different water content. The signals attributed to *mer*-$Co(pq\text{-}6\text{-}Ph)_3$ (black, red and blue labels) remain unchanged but the signals attributed to a $Co(pq\text{-}6\text{-}Ph)_2$-based species (purple labels) shift depending on the water content of the sample.

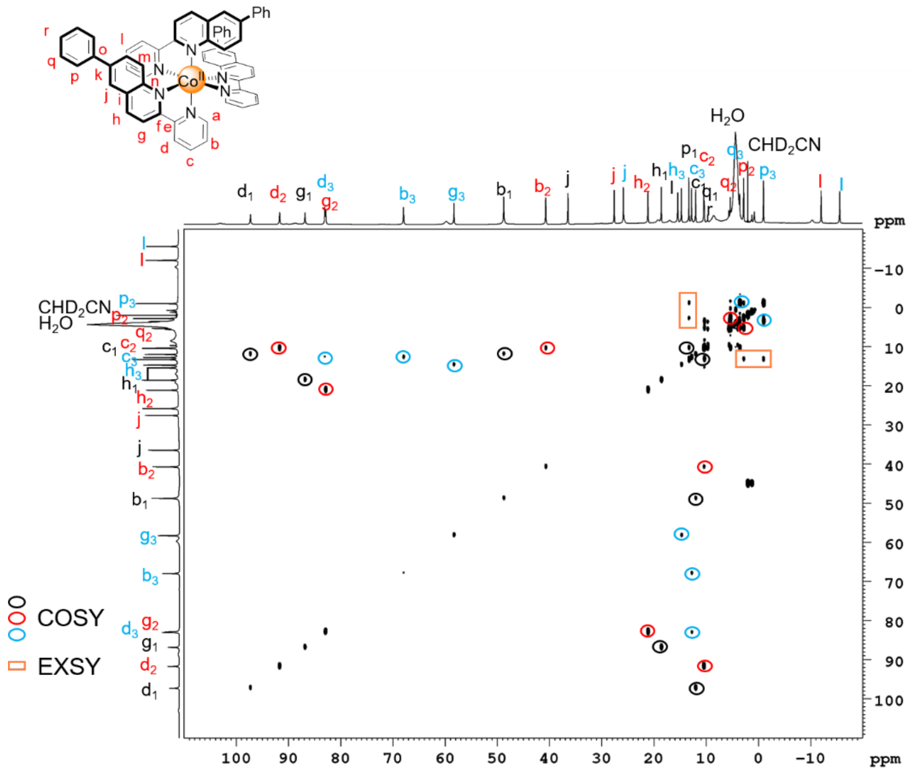

Figure S87. ^{1}H-^{1}H COSY NMR spectrum (600 MHz, CD_3CN, 298 K) of $[Co(pq\text{-}6\text{-}Ph)_3](BF_4)_2$. The black, red and blue circles represent through-bond (COSY) cross-peaks within the three ligand environments whereas orange squares represent exchange (EXSY) cross-peaks between the three ligand environments.

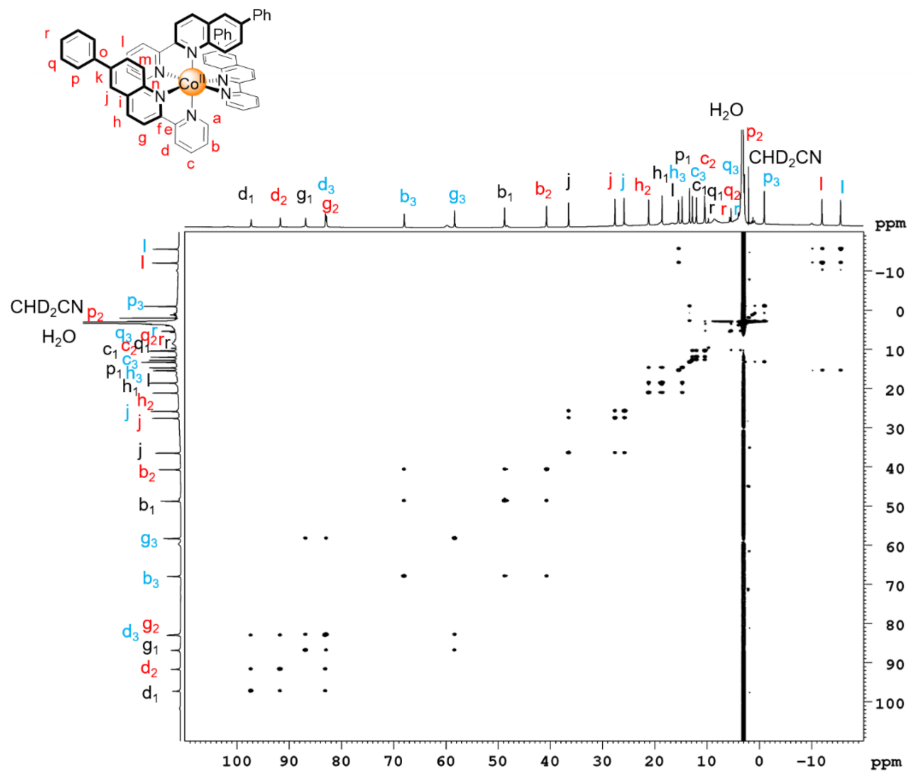

Figure S88. ^{1}H-^{1}H NOESY NMR spectrum (600 MHz, CD_3CN, 298 K) of $[Co(pq\text{-}6\text{-}Ph)_3](BF_4)_2$.

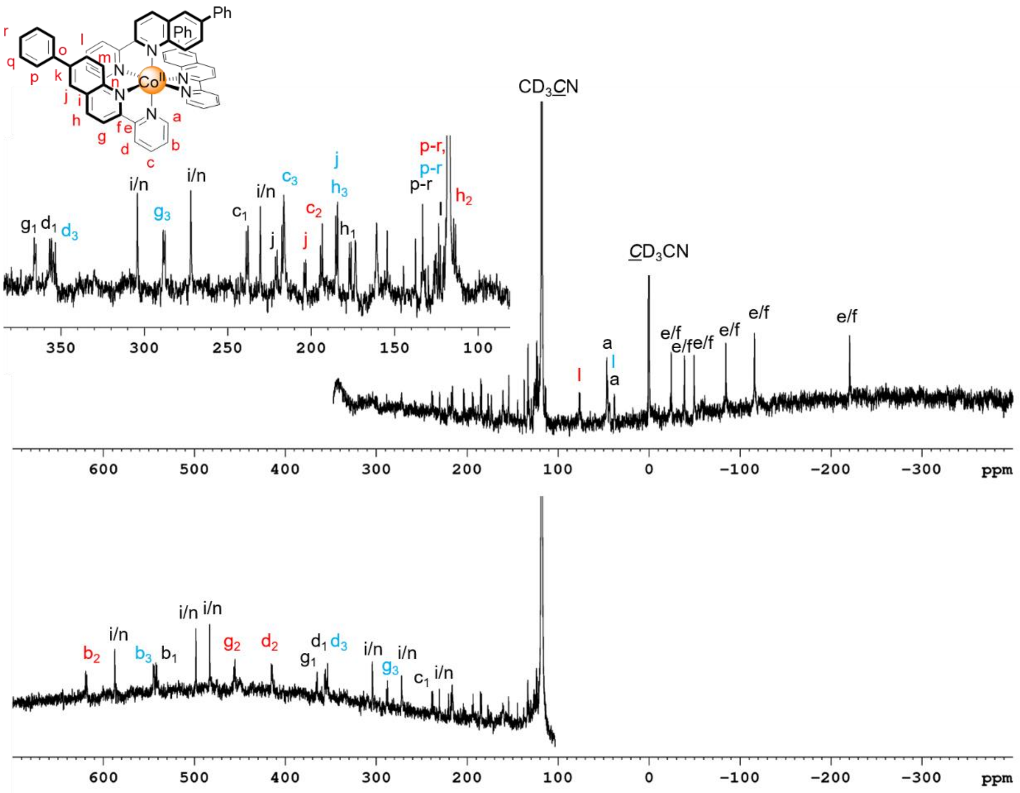

Figure S89. ^{13}C NMR spectrum (151 MHz, CD_3CN, 298 K) of $[Co(pq\text{-}6\text{-}Ph)_3](BF_4)_2$. Three spectra were measured to cover the entire 950 ppm range. Note: carbons *k*, *m* and *o* could not be assigned.

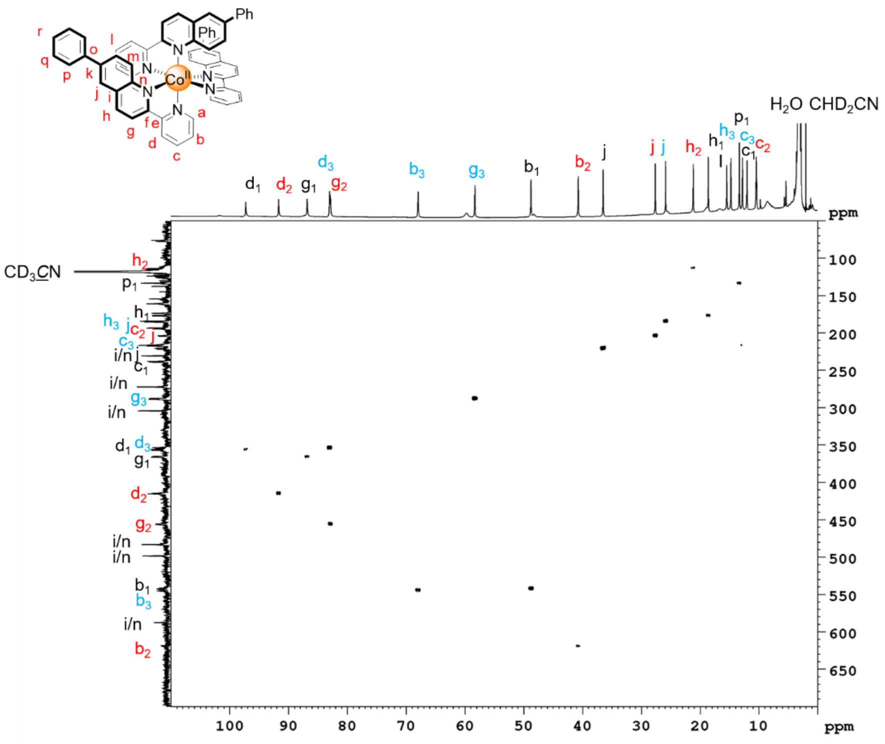

Figure S90. ^{1}H-^{13}C HMQC NMR spectrum (600 MHz/151 MHz, CD_3CN, 298 K) of $[Co(pq\text{-}6\text{-}Ph)_3](BF_4)_2$.

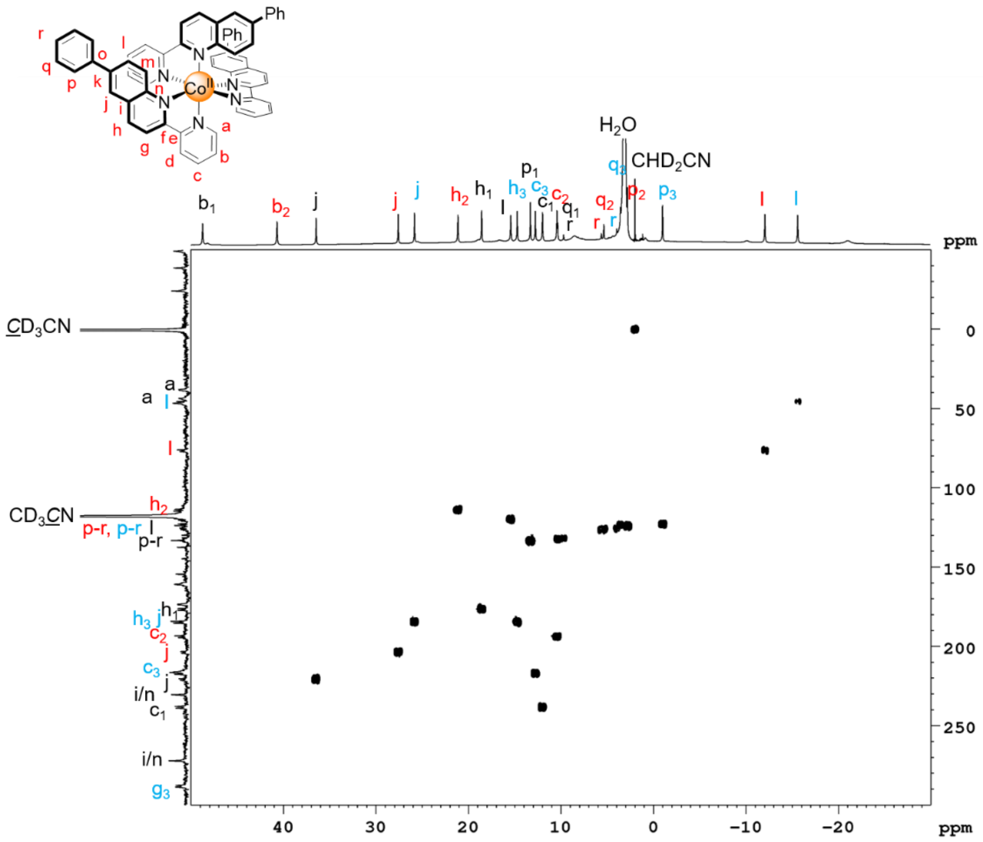

Figure S91. ^{1}H-^{13}C HMQC NMR spectrum (600 MHz/151 MHz, CD_3CN, 298 K) of $[Co(pq\text{-}6\text{-}Ph)_3](BF_4)_2$.

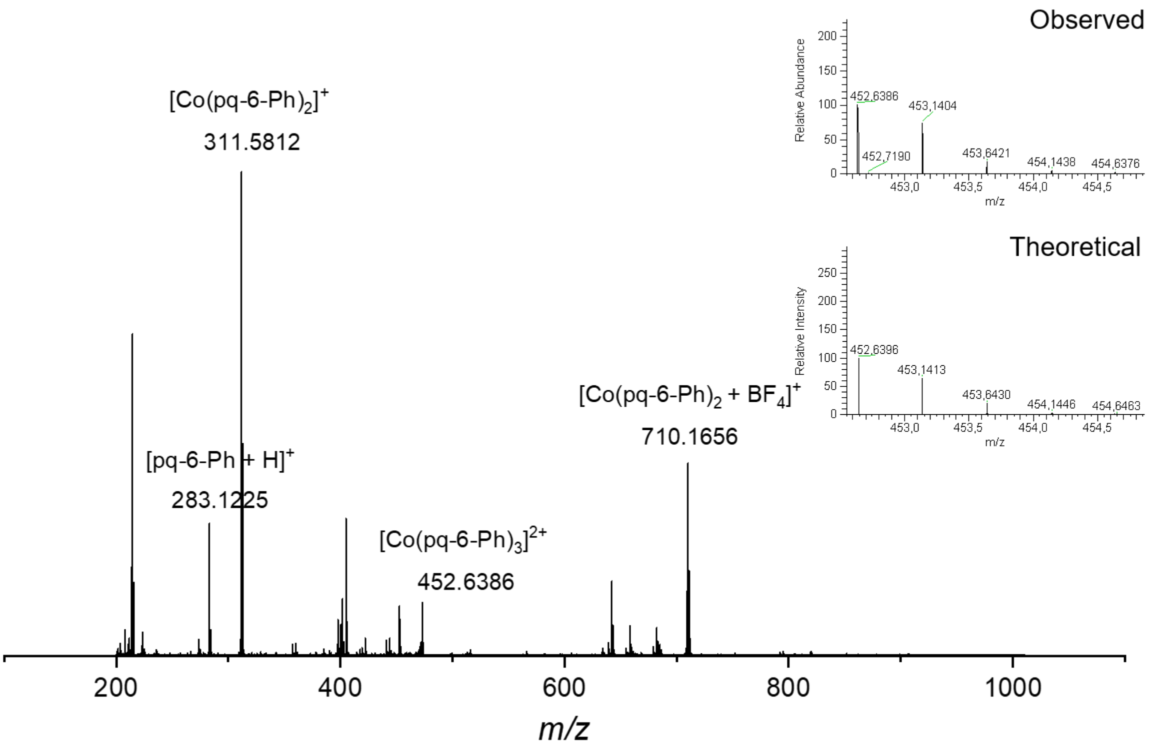

Figure S92. High resolution ESI mass spectrum of $[Co(pq\text{-}6\text{-}Ph)_3](BF_4)_2$ showing in the inset the observed (top) and theoretical (bottom) isotope patterns.

3.1.8 Comparison of Complexes **1a**-**7a**

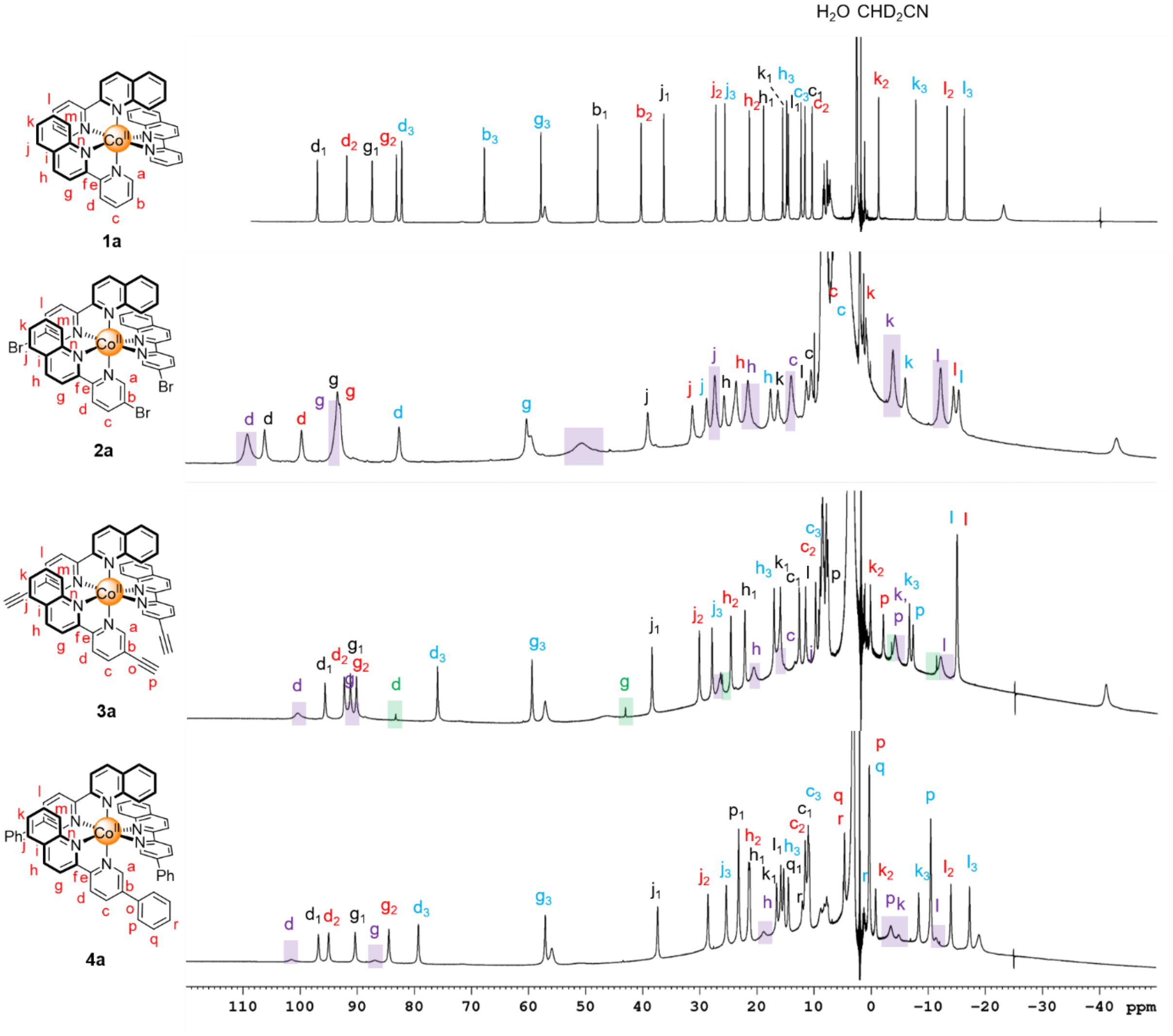

Figure S93. Comparison of the ^{1}H NMR spectra of complexes **1a**-**4a** with 5'-substituted ligands.

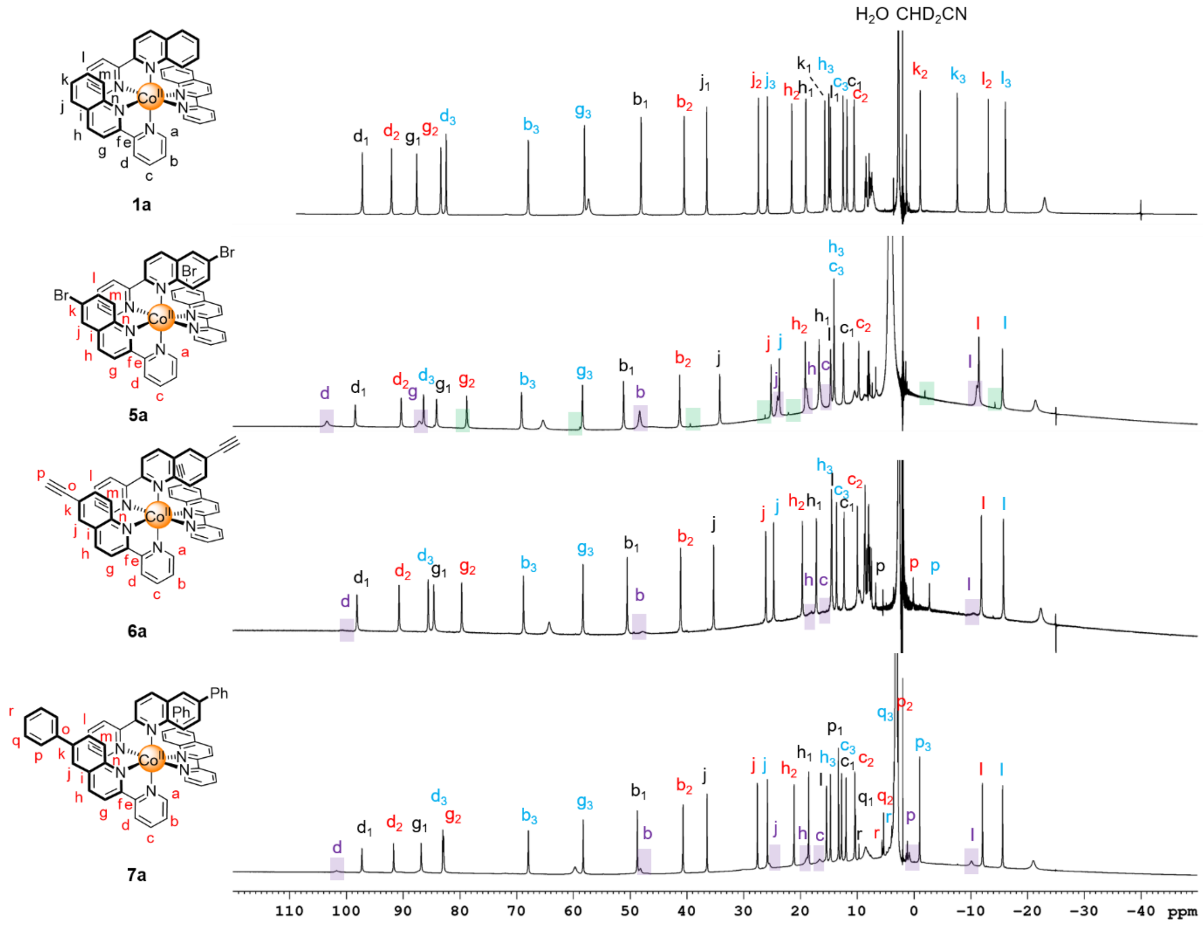

Figure S94. Comparison of the ^{1}H NMR spectra of complexes **1a, 5a-7a** with 6-substituted ligands.

The linewidths of Co^{II} complexes **1a**-**7a** and Co$^{II}{}_4$L$_6$ cage **8** were compared to investigate the relationship between linewidth and the observation of cross-peaks in the 2D NMR spectra (Figures S93-S94, Sections 3.1.1-3.1.7, 4). As the signals for protons *a* and *m* were broad due to their proximity to the paramagnetic Co^{II} and cross-peaks were not observed in any 2D NMR spectra, only the linewidths for protons *b-l* of the 2-(2-pyridyl)quinoline backbone and protons *p-r* of substituents in either the 5'- or 6-position are reported.

Table S5. Comparison of linewidth ranges (Hz) for each proton in the three different ligand environments for Co^{II} complexes **1a**-**7a** as well as cage **8.**

	b	c	d	g	h	j	k	l	p	q	r
**1a**	46-58	41-43	52-67	52-69	42-43	38-41	37-40	43-46	-	-	-
**2a**	-	*	>200	>200	>200	>200	>200	>200	-	-	-
**3a**	-	118	114-147	115-161	163-176	120-147	178	186	91	-	-
**4a**	-	*	91-137	142-172	137-146	142-183	106-120	90-110	*	*	*
**5a**	75-83	96-109	85-97	75-95	96-126	68-87	-	99-116	-	-	-
**6a**	56-67	56-72	64-80	58-80	58-61	48-50	-	55-61	17-32	-	-
**7a**	48-60	42-49	62-72	53-86	41-43	35-37	-	45-49	34-37	*	*
**8**	52	37	68	61	34	-	-	-			

* Could not be determined due to overlapping signals. - Not applicable

The ^{1}H NMR spectra of the ligands were measured in CD_3CN to determine the chemical shift for each proton for NOESY and steady-state NOE experiments.

Table S6. Chemical shifts of the ligand protons (600 MHz, CD_3CN, 298 K).

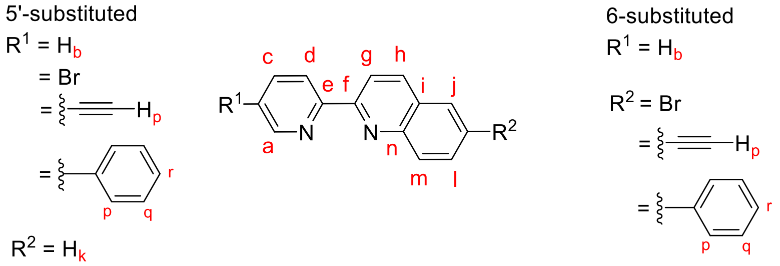

R	a	b	c	d	g	h	j	k	l	m	p	q	r
H	8.72	7.44	7.95	8.65	8.60	8.39	7.96	7.61	7.78	8.12	-	-	-
5’-Br	8.81	-	8.12	8.58	8.54	8.40	7.96	7.79	7.63	8.12	-	-	-
5’-CCH	8.82	-	8.03	8.66	8.58	8.41	7.97	7.62	7.80	8.12	3.69	-	-
5’-Ph	9.02	-	8.22	8.75	8.64	8.44	7.99	7.64	7.81	8.18	7.79	7.55	7.47
6-Br	8.72	7.46	7.95	8.63	8.62	8.32	8.16	-	7.86	8.02	-	-	-
6-CCH	8.72	7.46	7.96	8.64	8.62	8.36	8.12	-	7.79	8.08	3.56	-	-
6-Ph	8.74	7.45	7.97	8.67	8.63	8.45	8.22	-	8.09	8.19	7.83	7.54	7.44

3.1.9 $[Co(bpy)_3](BF_4)_2$

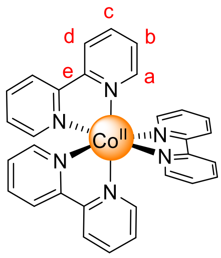

A solution of $Co(BF_4)_2$•$6H_2O$ (50 mg, 0.15 mmol) in ethanol (1 mL) was added to a solution of 2,2’-bipyridine (99.0 mg, 0.63 mmol) in ethanol (1 mL). The reaction mixture was heated up to 50 °C for 30 min. The crystals were isolated and washed three times with diethyl ether.

^{1}H NMR (600 MHz, CD_3CN, 298 K) δ (ppm): 87.8 (bs, 6H, H_a), 84.0 (s, 6H, H_d), 46.1 (s, 6H, H_c), 14.5 (s, 6H, H_b).

^{13}C NMR (151 MHz, CD_3CN, 298 K) δ (ppm): 600.3 (d, ^{1}J = 178 Hz, C_b), 416.0 (d, ^{1}J = 164 Hz, C_d), 181.5 (d, ^{1}J = 166 Hz, C_c), 33.6 (d, ^{1}J = 119 Hz, C_a), -137.2 (s, C_e).

HRMS (ESI) *m/z*: 458.0721 $[Co(bpy)_2 + BF_4]^+$, 263.5688 (calculated for $C_{30}H_{24}N_6Co$: 263.5692) $[Co(bpy)_3]^{2+}$.

3.1.9.1 Characterisation using Paramagnetic NMR Spectroscopy and Mass Spectrometry

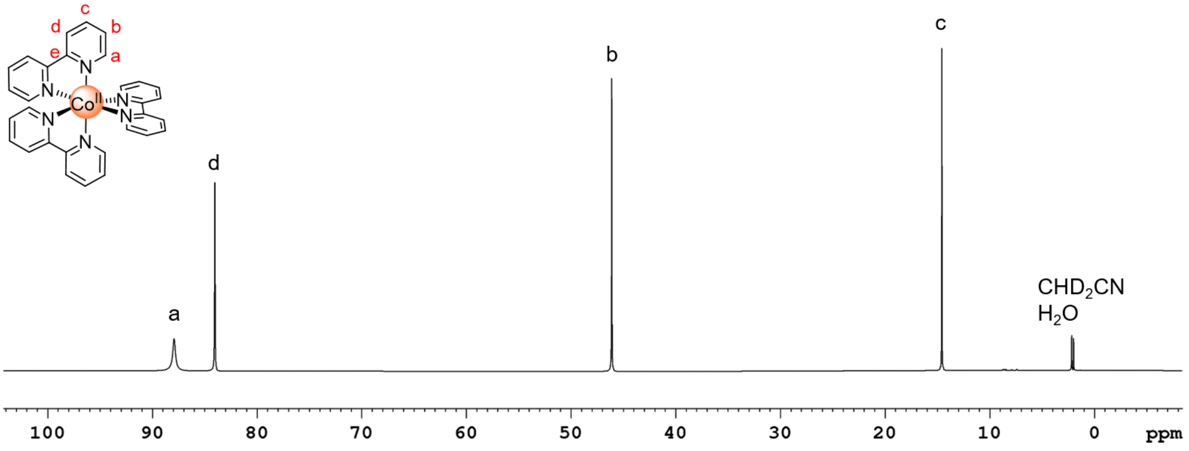

Figure S95. ^{1}H NMR spectrum (600 MHz, CD_3CN, 298 K) of $[Co(bpy)_3](BF_4)_2$.

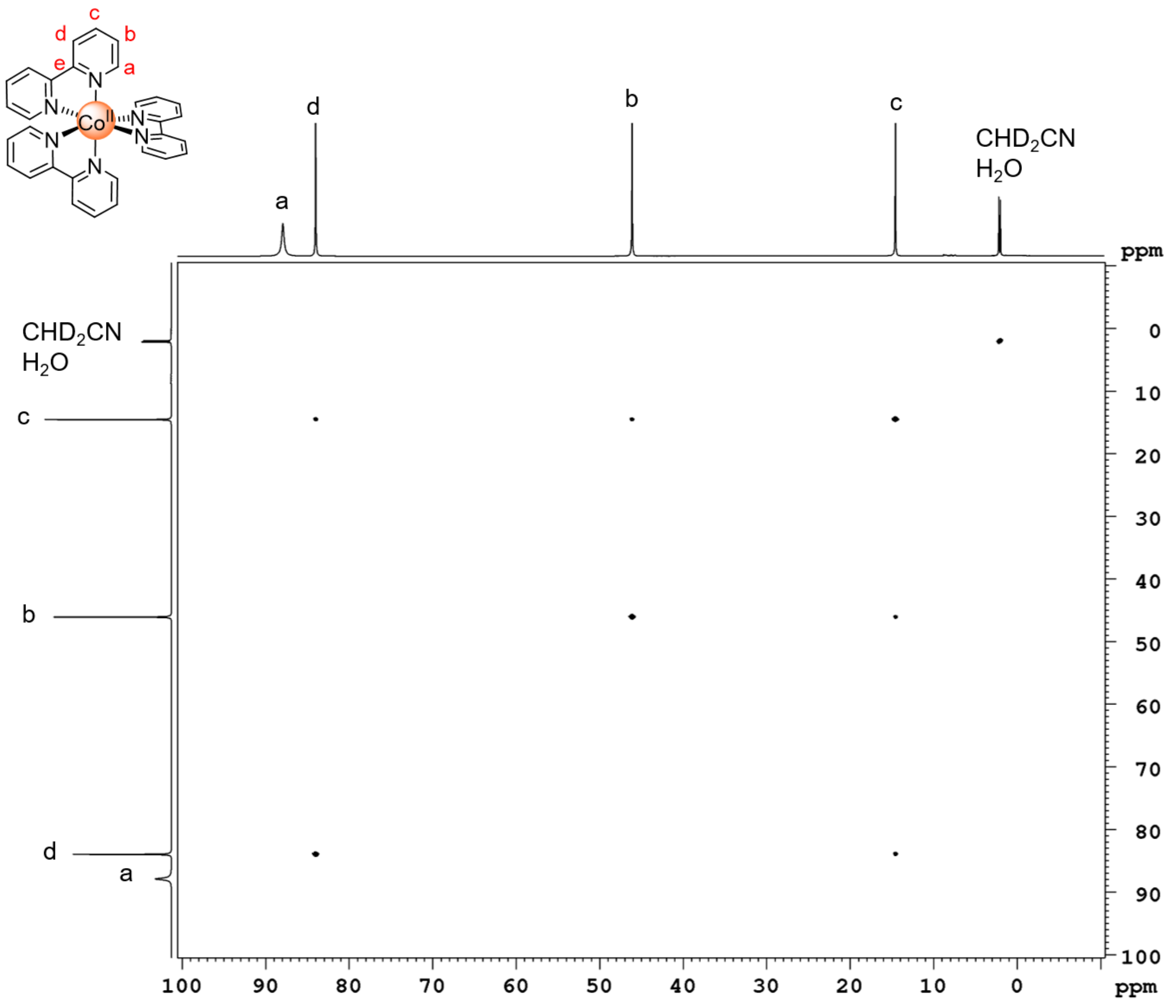

Figure S96. ^{1}H-^{1}H COSY NMR spectrum (500 MHz, CD_3CN, 298 K) of $[Co(bpy)_3](BF_4)_2$.

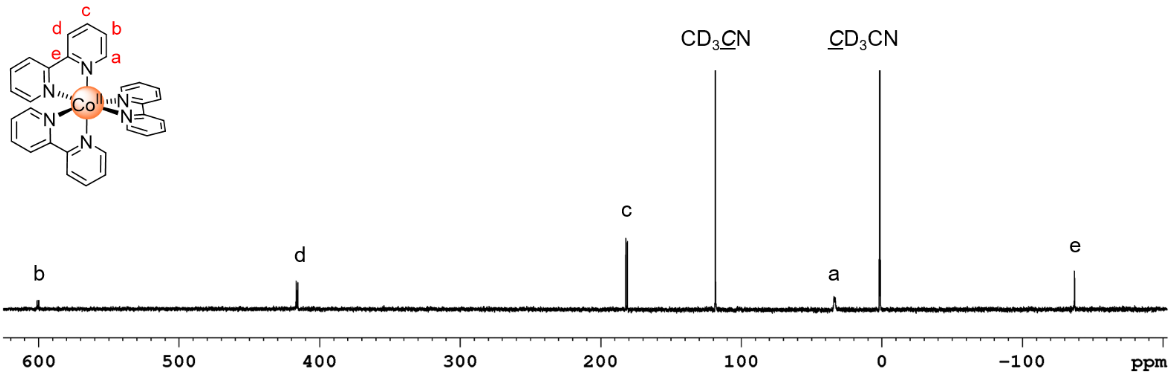

Figure S97. ^{13}C NMR spectrum (151 MHz, CD_3CN, 298 K) of $[Co(bpy)_3](BF_4)_2$.

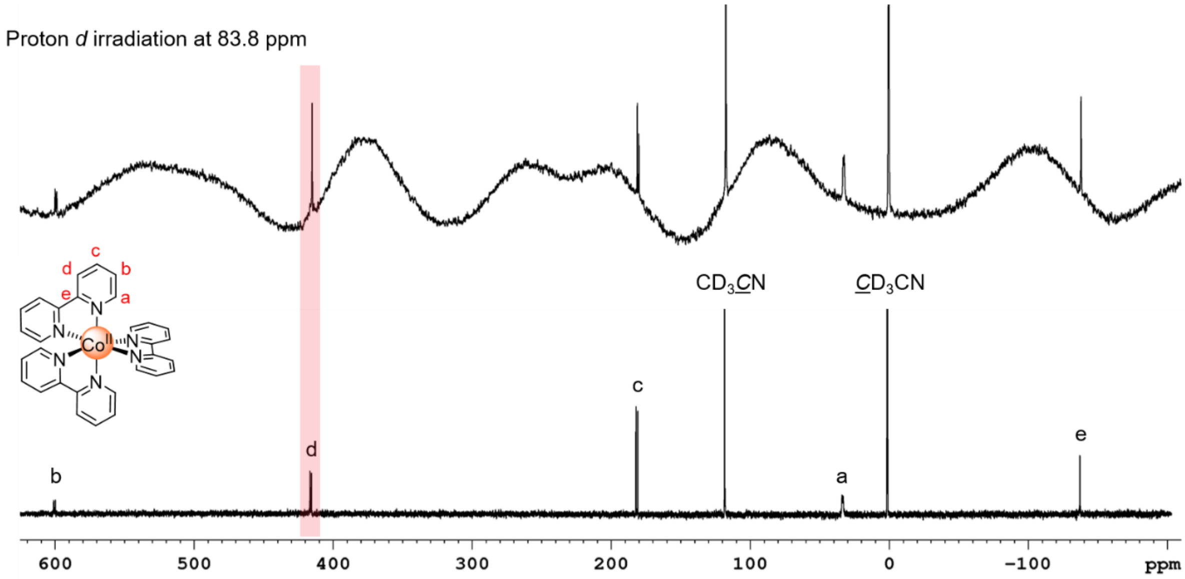

Figure S98. Comparison of ^{1}H coupled (below) and decoupled (above) ^{13}C NMR spectra (151 MHz, CD_3CN, 298 K) of $[Co(bpy)_3](BF_4)_2$ showing the loss of the doublet for carbon *d* when proton *d* is irradiated (83.4 ppm).

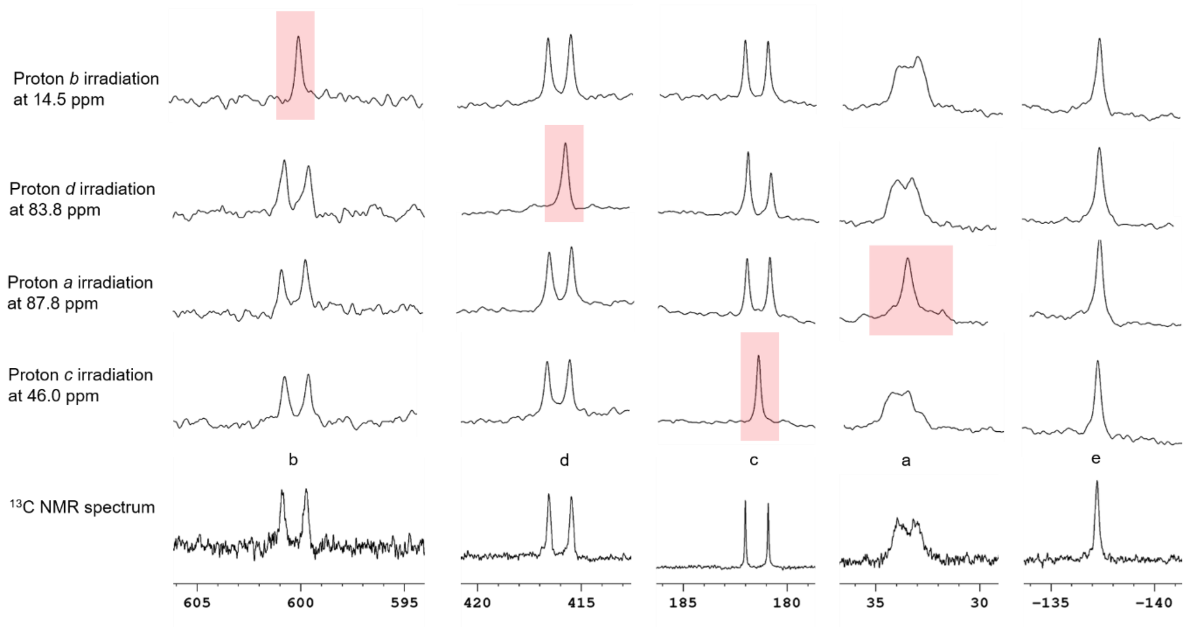

Figure S99. Comparison of selective ^{1}H decoupled ^{13}C NMR spectra (151 MHz, CD_3CN, 298 K) of $[Co(bpy)_3](BF_4)_2$ when different proton signals are irradiated.

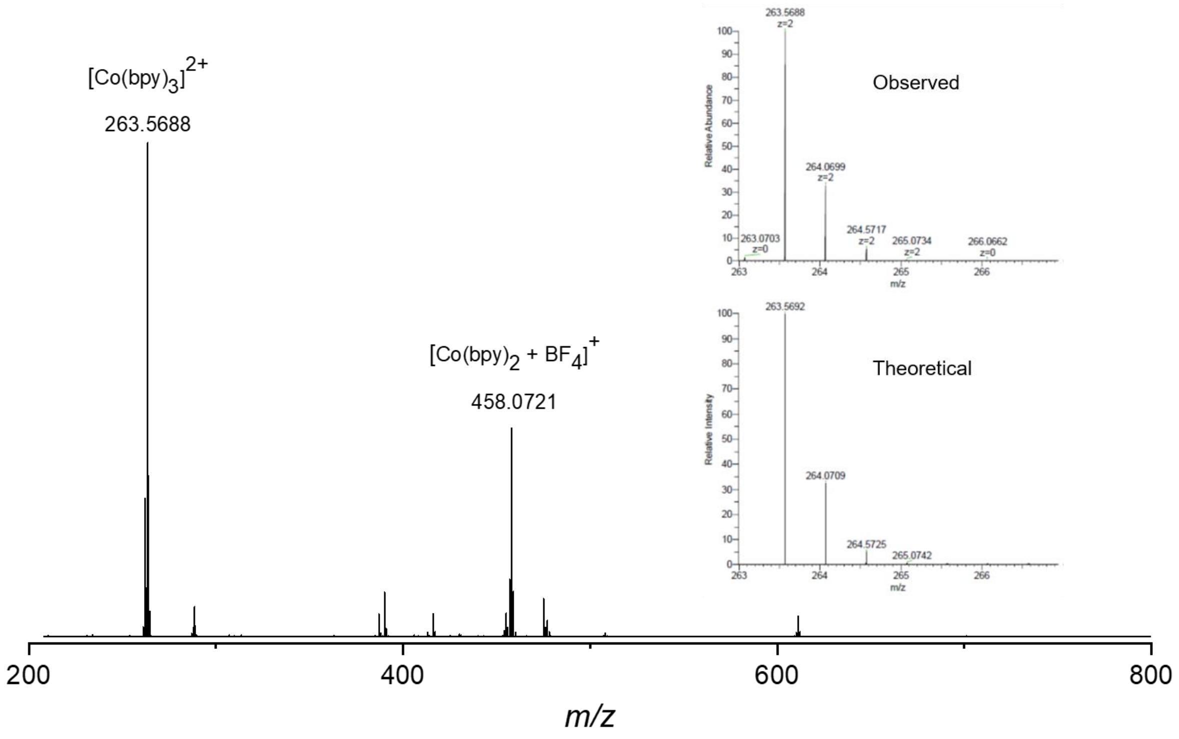

Figure S100. High resolution ESI mass spectrum of $[Co(bpy)_3](BF_4)_2$ showing in the inset the observed (top) and theoretical (bottom) isotope patterns.

3.2 Mononuclear Iron Complex

Although complex **1b** is literature-known as its tetrafluoroborate and perchlorate salts,[13] we prepared the triflate salt since: i) $Fe(OTf)_2$ is commercially available as a salt without waters of crystallisation unlike the tetrafluoroborate salt; ii) triflate salts are safer alternatives to perchlorates.

3.2.1 *mer*-$[Fe(pq)_3](OTf)_2$ (**1b**)

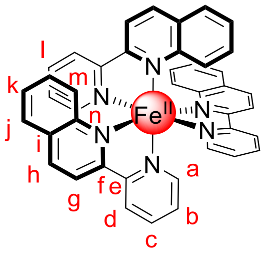

In a glovebox under a nitrogen atmosphere, $Fe(OTf)_2$ (4.96 mg, 14.0 µmol) and 2-(2-pyridyl)quinoline (9.1 mg, 44.1 µmol) were dissolved in dry CD_3CN (0.5 mL) in an NMR tube.

^{1}H NMR (500 MHz, CD_3CN, 248 K) δ (ppm): 119.8 (s, 1H, H_{d1}), 98.0 (s, 1H, H_{d2}), 95.7 (s, 1H, H_{d3}), 92.4 (s, 1H, H_{g1}), 79.1 (s, 1H, H_{g2}), 71.5 (s, 1H, H_{g3}), 63.3 (s, 1H, H_{b2}), 62.7 (s, 1H, H_{b3}), 54.6 (s, 1H, H_{b1}), 27.3 (s, 1H, H_{j1}), 26.8 (s, 1H, H_{j2}), 23.9 (s, 1H, H_{j3}), 9.1 (s, 1H, H_{h2}), 4.2 (s, 1H, H_{k2}), 3.1 (s, 1H, H_{k3}), 2.0 (s, 1H, H_{c1}), 1.0 (s, 1H, H_{k1}), 0.5 (s, 1H, H_{h1}), -1.5 (s, 1H, H_{c3}), -1.8 (s, 2H, H_{l1}, H_{h3}), -3.3 (s, 1H, H_{c2}), -9.3 (s, 1H, H_{l2}), -19.0 (s, 1H, H_{l3}), -59.1 (s, 1H, H_m), -126.4 (s, 1H, H_m), -137.6 (s, 1H, H_m).

3.2.1.1 Characterisation at 248 K using Paramagnetic NMR Spectroscopy

As shown in variable temperature experiments (Section S3.2.1.2), the ^{1}H NMR spectrum of $[Fe(pq)_3](OTf)_2$ contained broad signals at 298 K, precluding detailed analysis. The signals sharpened considerably upon cooling to 248 K and therefore, characterisation of the complex was carried out at 248 K using the paramagnetic NMR toolbox (Figures S101-S105).

The signals with black, red and blue labels correspond to the three ligand environments of the major species, *mer*-$Fe(pq)_3$. However, different spin systems (i.e. protons *b*-*d*, *g*-*h*, *j*-*l*) could not be correlated to a particular ligand environment using NOESY spectroscopy and therefore, each spin-system was arbitrarily labelled with black, red and blue labels according to decreasing chemical shift of protons *d*, *g* and *j* to represent the three different ligand environments. Where present, the numbers indicate signals belonging to the same spin system as established by COSY NMR spectroscopy.

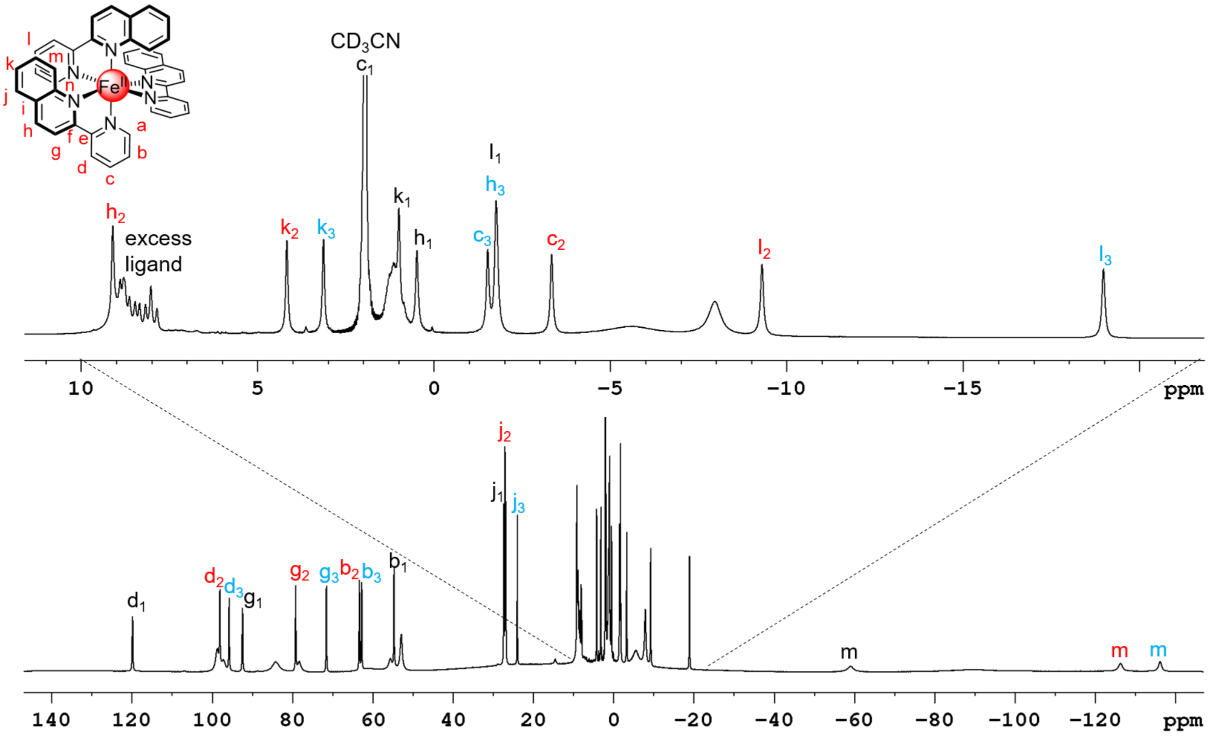

Figure S101. ^{1}H NMR spectrum (500 MHz, CD_3CN, 248 K) of *mer*-$[Fe(pq)_3](OTf)_2$. The signals with black, red and blue labels correspond to the three ligand environments of the major species, *mer*-$Fe(pq)_3$. However, different spin systems (i.e. protons *b*-*d*, *g*-*h*, *j*-*l*) could not be correlated to a particular ligand environment using NOESY spectroscopy. Therefore, the three sets of signals were arbitrarily labelled with black, red and blue labels according to the decreasing chemical shift of protons *d*, *g* and *j*. Where present, the numbers indicate signals belonging to the same spin system as established by COSY NMR spectroscopy.

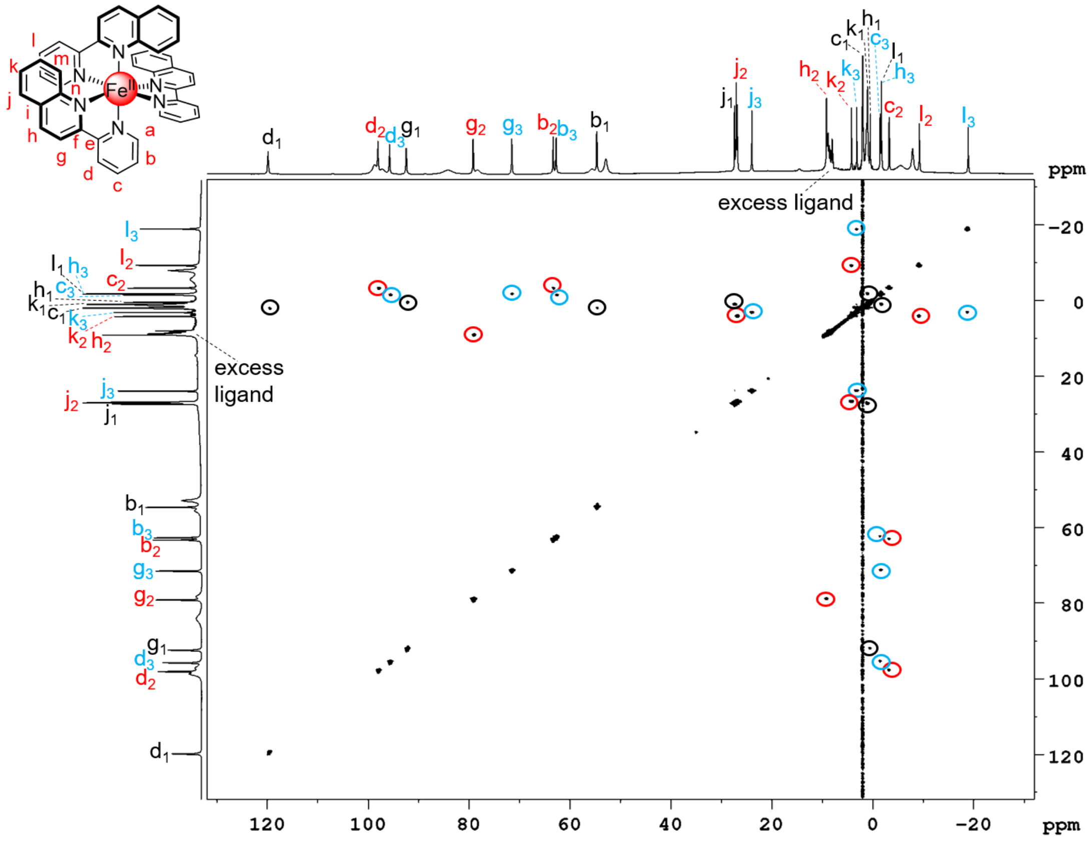

Figure S102. ^1H-^1H COSY spectrum (500 MHz, CD_3CN, 248 K) of *mer*-[Fe(pq)$_3$](OTf)$_2$.

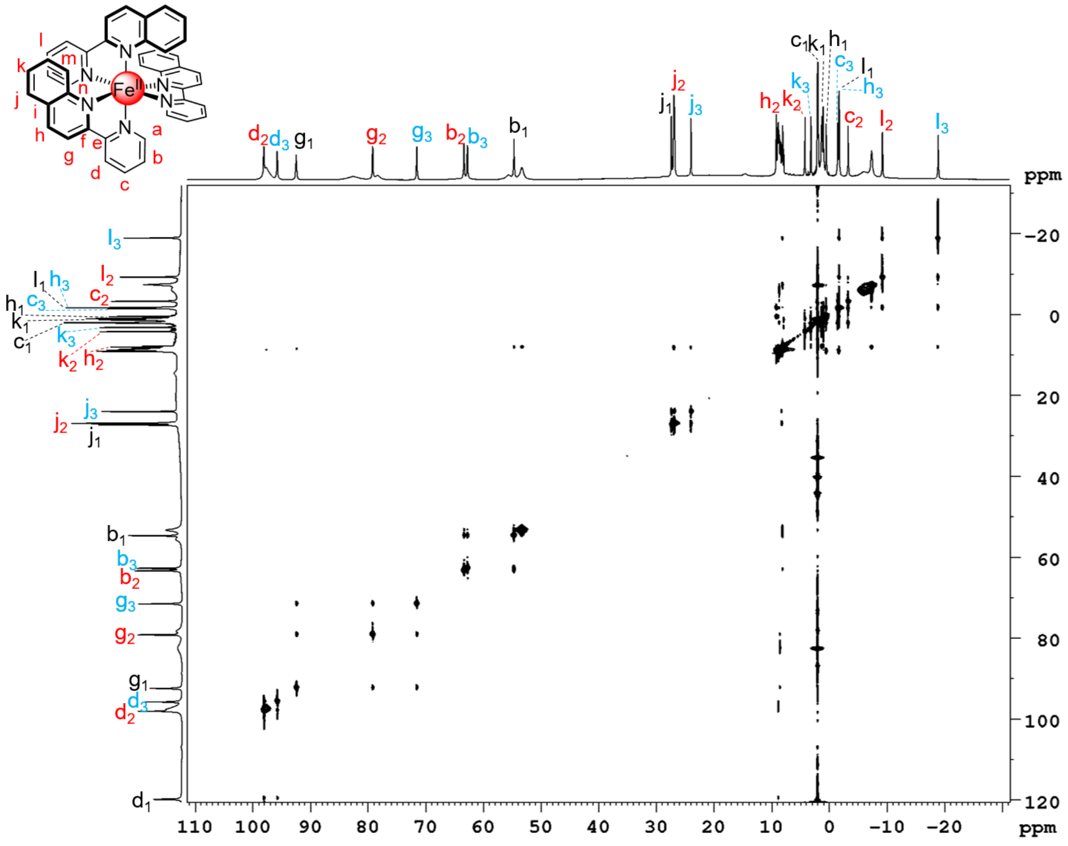

Figure S103. ^1H-^1H NOESY spectrum (500 MHz, CD_3CN, 248 K) of *mer*-[Fe(pq)$_3$](OTf)$_2$.

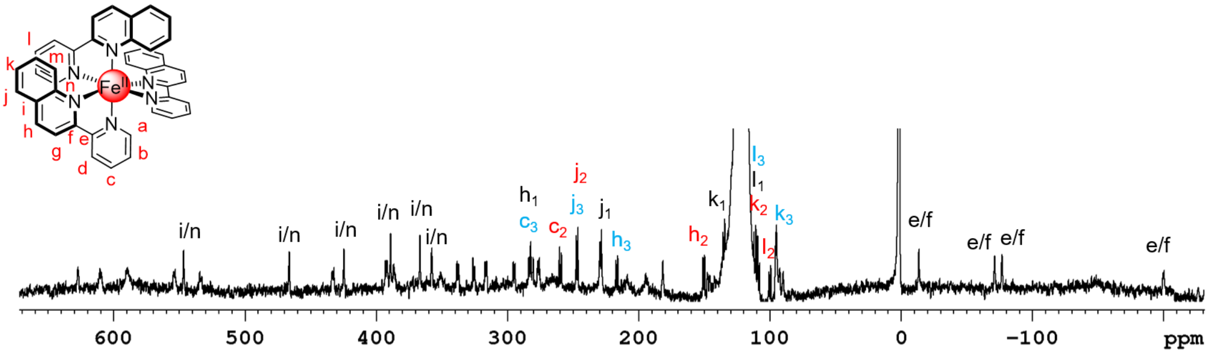

Figure S104. ^{13}C NMR spectrum (500 MHz, CD_3CN, 248 K) of *mer*-$[Fe(pq)_3](OTf)_2$. Assignment of the complete spectrum was not possible due to the absence of some cross-peaks in the HMQC spectra.

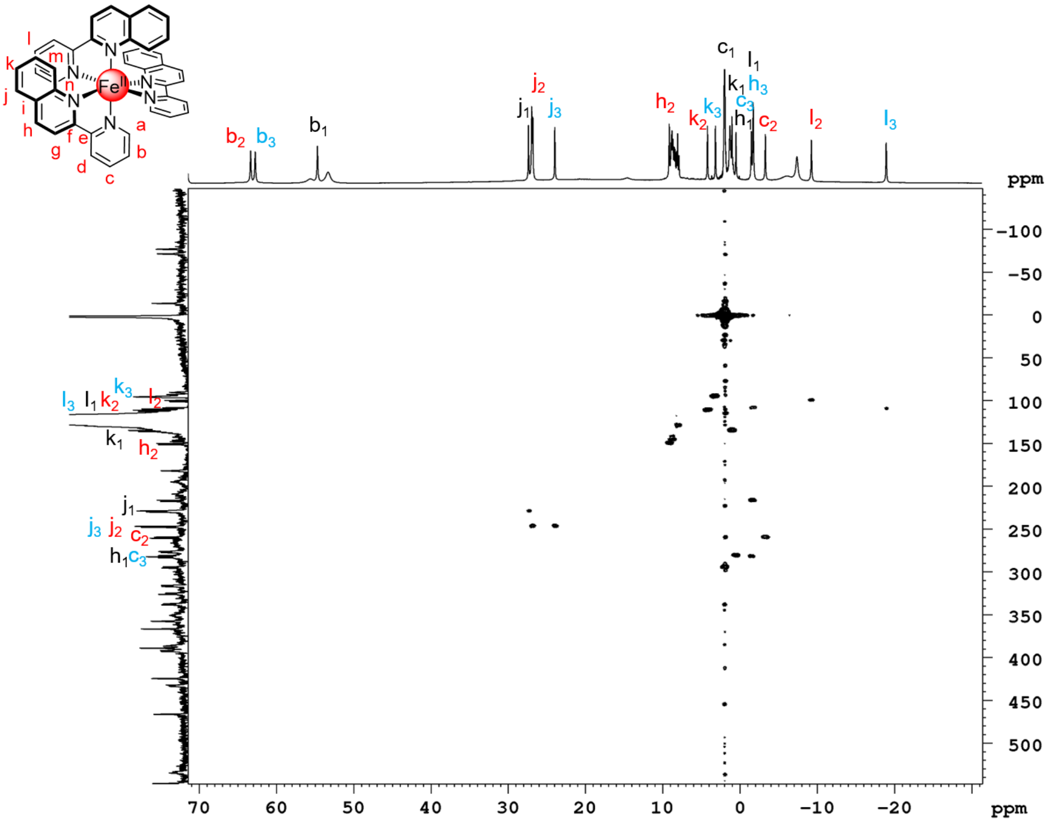

Figure S105. ^{1}H-^{13}C HMQC NMR spectrum (500 MHz/125 MHz, CD_3CN, 248 K) of *mer*-$[Fe(pq)_3](OTf)_2$. Two additional HMQC spectra were measured to cover the entire spectral range but the remaining carbon signals could not be assigned due to missing or very weak cross-peaks, which could not be distinguished from noise.

3.2.1.2 Variable Temperature Studies

Variable temperature ^{1}H NMR studies (Figures S106-109) showed that the ^{1}H NMR spectrum contained broad signals at 298 K but the signals sharpened as the temperature was lowered from 298 K to 248 K, enabling assignment at 248 K using the paramagnetic NMR toolbox. Variable temperature COSY, NOESY and HMQC spectra were also measured over this temperature range to investigate the effect of linewidth (Table S7) on the observation of cross-peaks (Figures S110-S112). While cross-peaks were observed in the NOESY spectra (Figure S111) irrespective of linewidth, the number of observable cross-peaks in the COSY (Figure S110) and HMQC (Figure S112) decreased as the linewidth increased (Table S7) as the temperature was raised to 266 K.

Table S7. Comparison of linewidth ranges (Hz) for each proton in the three different ligand environments for complex **1b** at different temperatures

Temp. (K)	b	c	d	g	h	j	k	l
248	62-64	46-50	79-101	63-88	47-52	38-43	37-58	50-60
257	67-75	61	84-91	70-82	67-85	51-53	47-56	60-63
266	91-119	94-97	105-117	93-102	99-137	88-90	93-94	87-102
272	120-126	140	129-140	119-126	144	128-144	>100	118-136
283	> 200	> 200	> 200	> 200	> 200	> 200	> 200	140-150

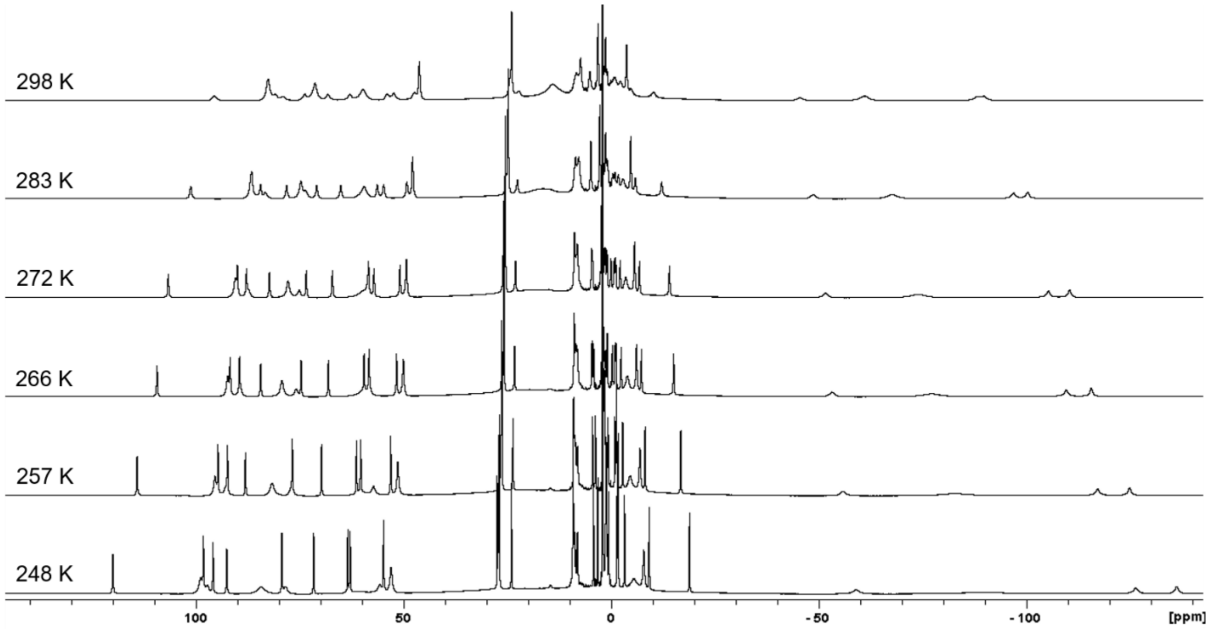

Figure S106. ^{1}H NMR (500 MHz, CD_3CN) spectra of $[Fe(pq)_3](OTf)_2$ at different temperatures. See Figures S107-S108 for proton assignments.

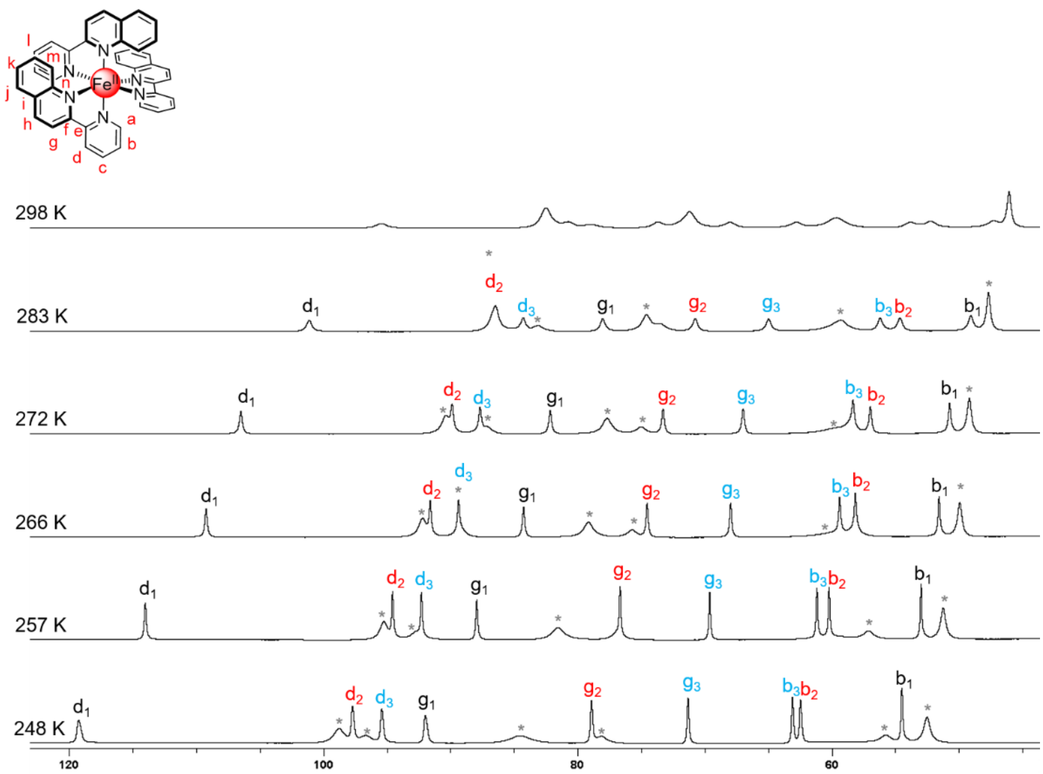

Figure S107. Downfield inset of the ^1H NMR (500 MHz, CD_3CN) spectra of $[Fe(pq)_3](OTf)_2$ at different temperatures. Signals marked with * correspond to additional species present at equilibrium that could not be assigned due to the broadness of the signals.

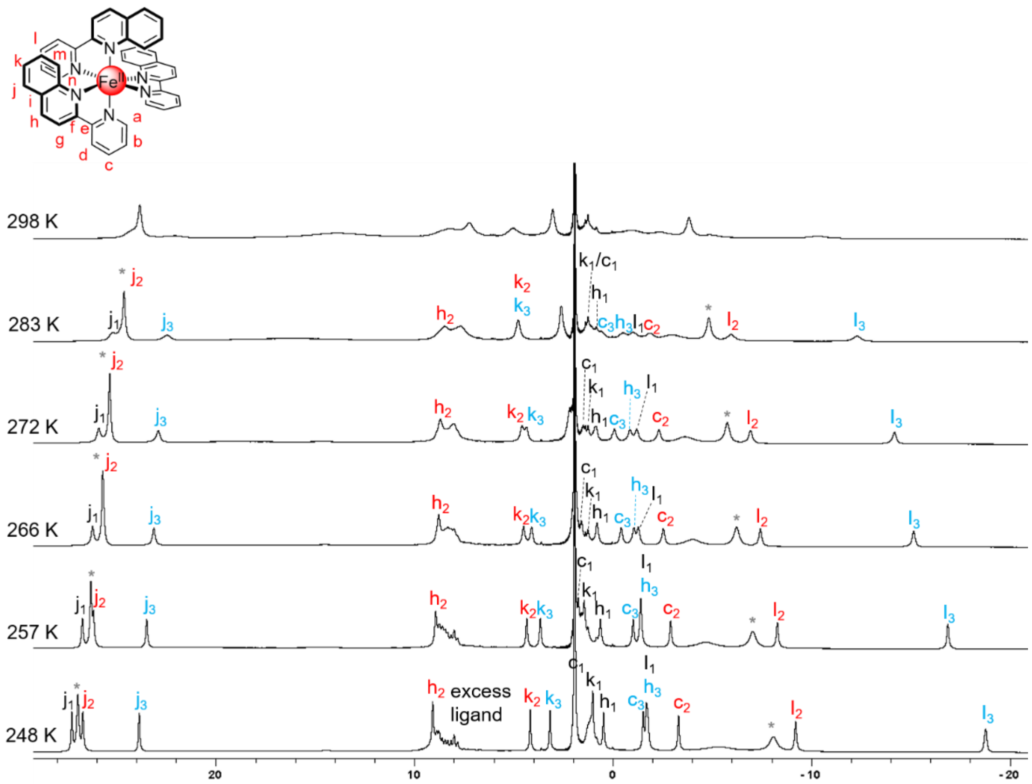

Figure S108. Upfield inset of the ^1H NMR (500 MHz, CD_3CN) spectra of $[Fe(pq)_3](OTf)_2$ at different temperatures. Signals marked with * correspond to additional species present at equilibrium that could not be assigned due to the broadness of the signals.

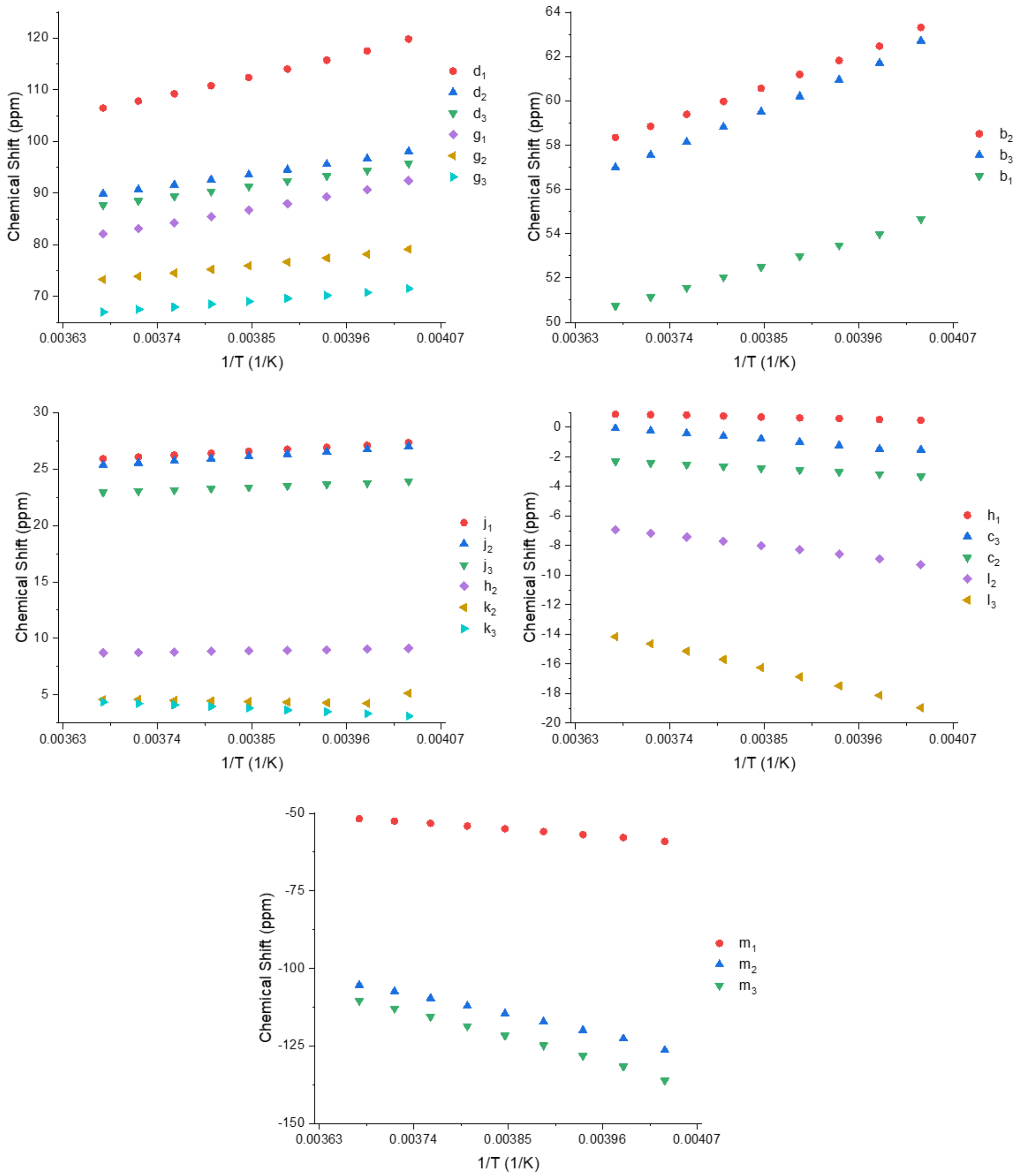

Figure S109. Curie-Weiss plot showing the chemical shift changes of $[Fe(pq)_3](OTf)_2$ as a function of 1/T. The linear relationship between chemical shift and 1/T indicates the complex shows Curie-Weiss behaviour over this temperature range.

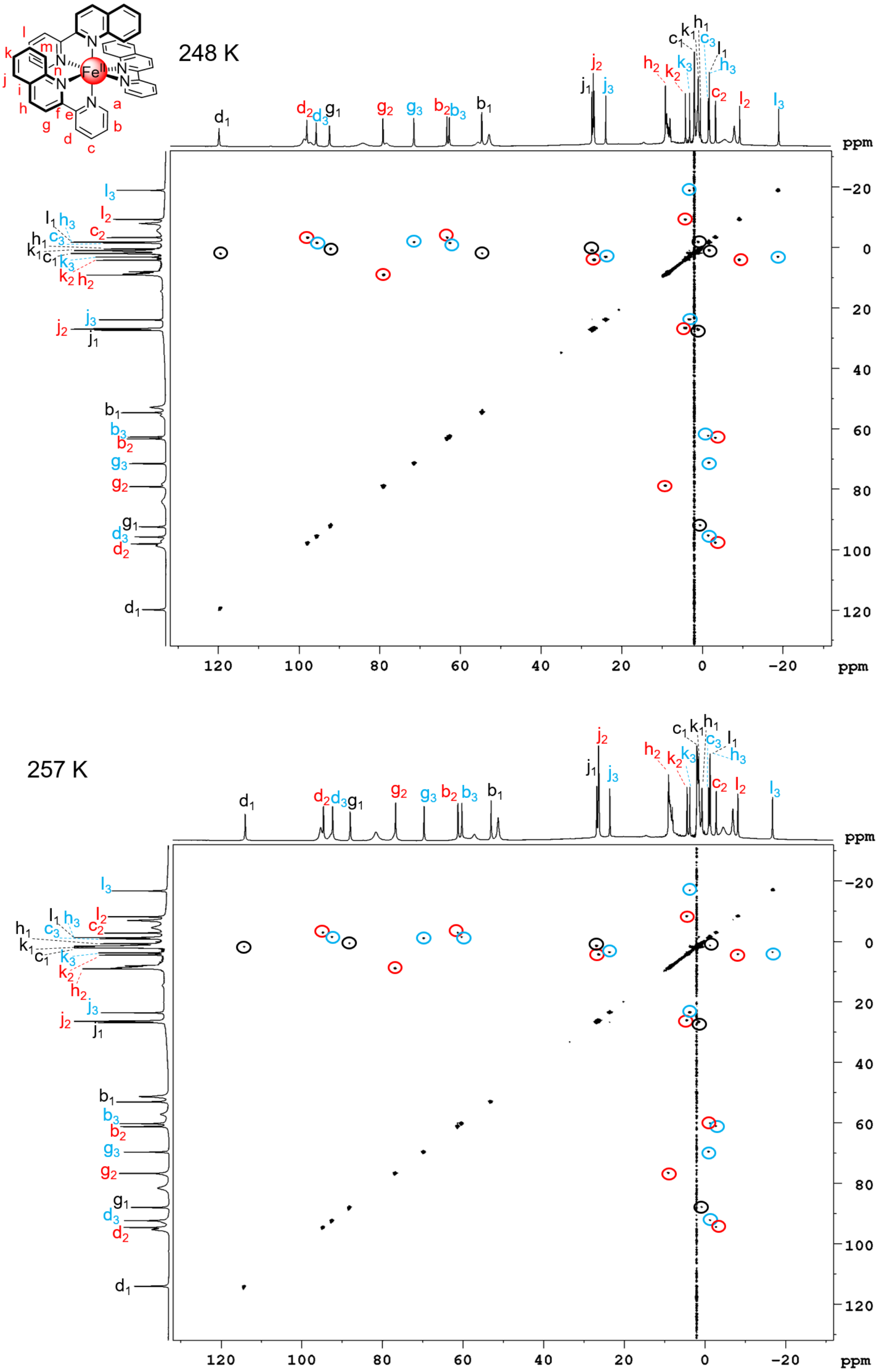

Figure S110a. Comparison of ^{1}H-^{1}H COSY (500 MHz, CD_3CN) spectra of $[Fe(pq)_3](OTf)_2$ at 248 K, 257 K and 266 K showing the loss of cross-peaks as the temperature increases. At 272 K no cross-peaks were observed.

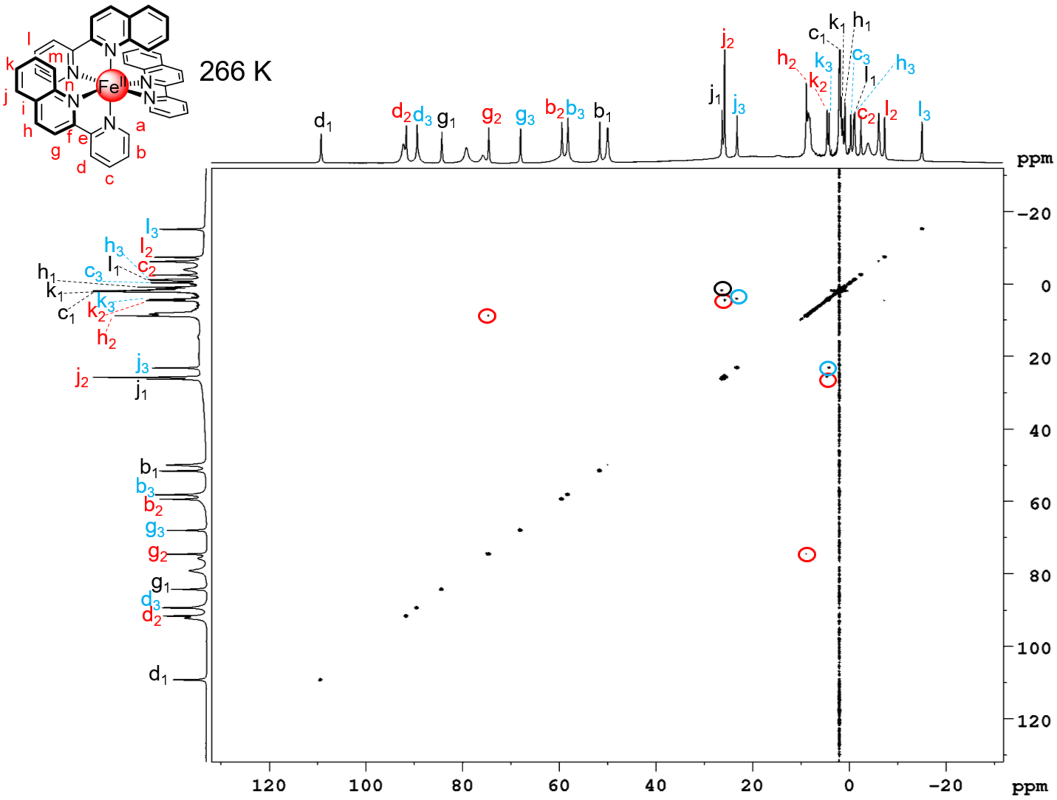

Figure S110b. Comparison of ^{1}H-^{1}H COSY (500 MHz, CD_3CN) spectra of $[Fe(pq)_3](OTf)_2$ at 248 K, 257 K and 266 K showing the loss of cross-peaks as the temperature increases. At 272 K no cross-peaks were observed.

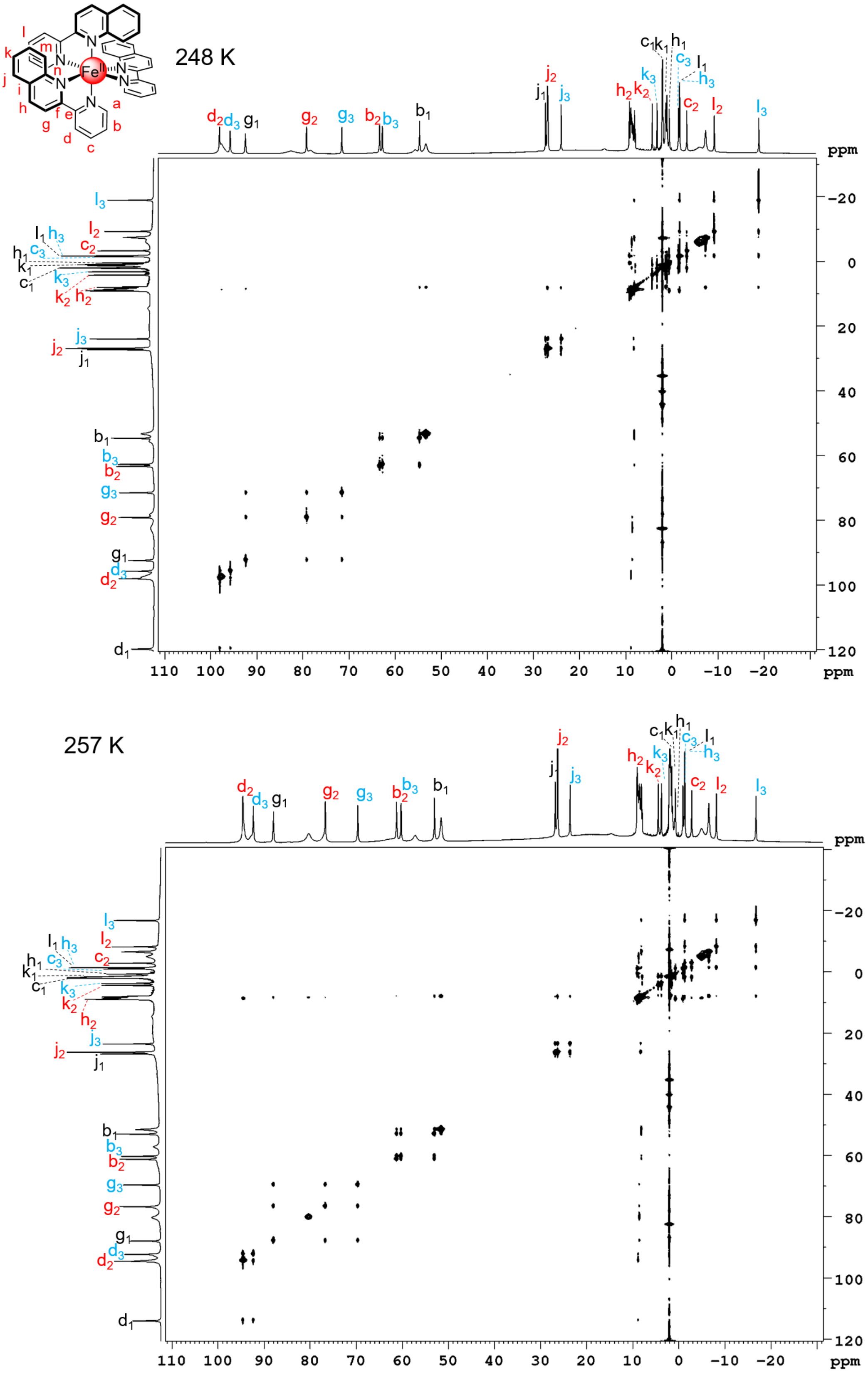

Figure S111a. Comparison of ^{1}H-^{1}H NOESY (500 MHz, CD_3CN) spectra of $[Fe(pq)_3](OTf)_2$ at 248 K, 257 K, 266 K and 272 K.

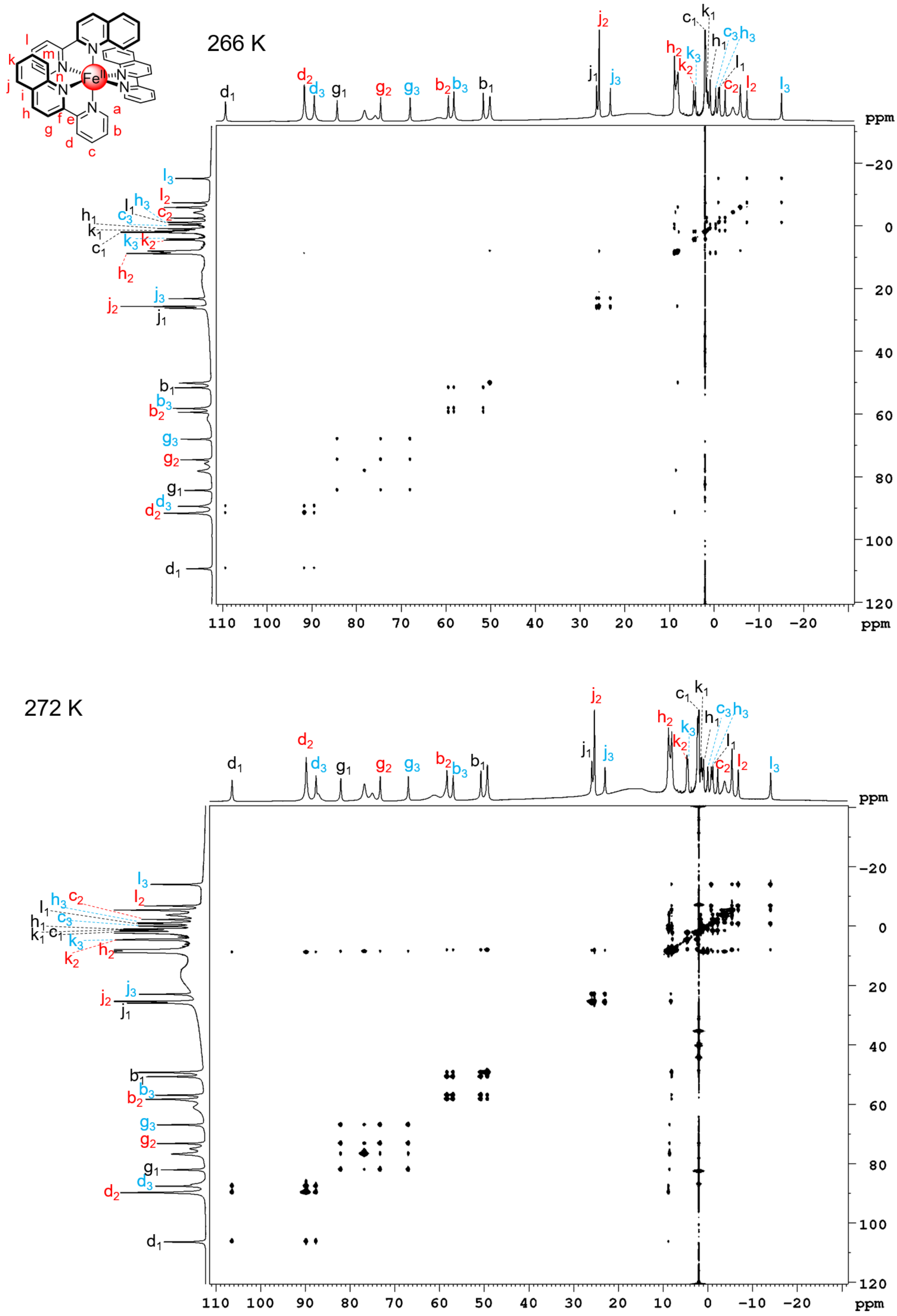

Figure S111b. Comparison of ^1H-^1H NOESY (500 MHz, CD_3CN) spectra of $[Fe(pq)_3](OTf)_2$ at 248 K, 257 K, 266 K and 272 K.

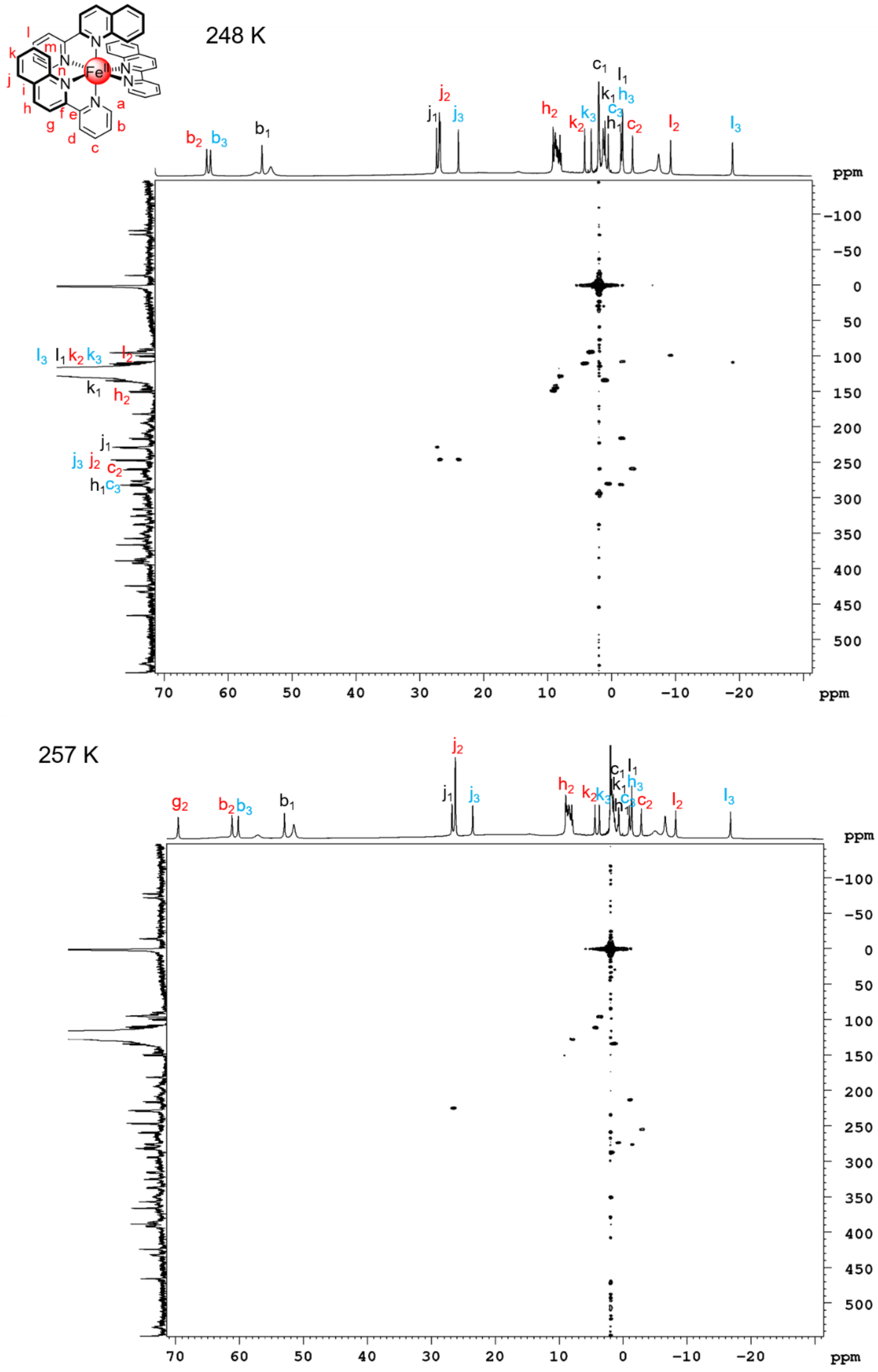

Figure S112a. Comparison of ^{1}H-^{13}C HMQC (500 MHz/125 MHz, CD_3CN) spectra of $[Fe(pq)_3](OTf)_2$ at 248 K, 257 K and 266 K. While additional HMQC spectra were recorded to cover the spectral range, additional cross-peaks were observed in the second spectral range at 257 K only (Figure S112b, top).

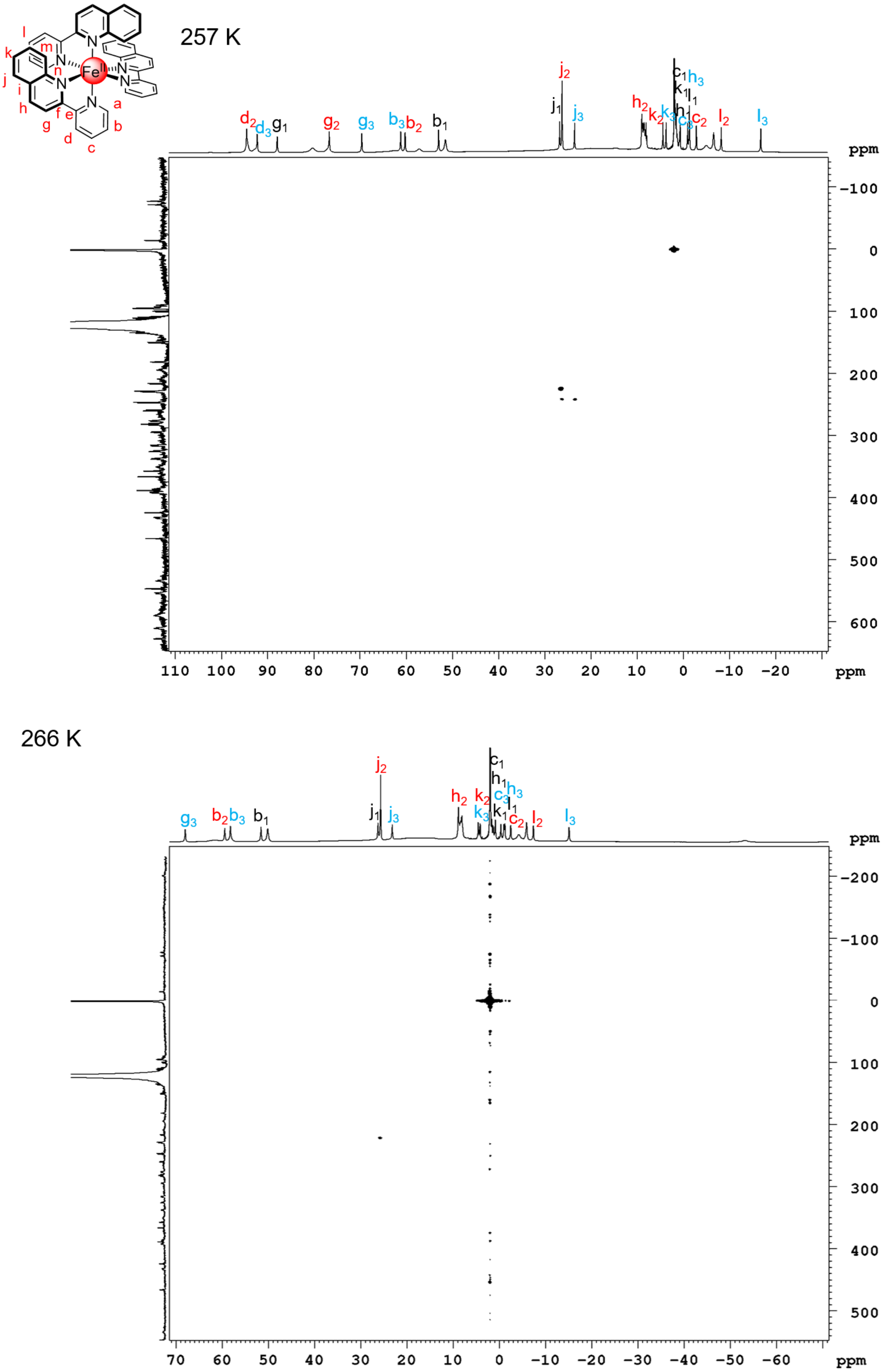

Figure S112b. Comparison of ^{1}H-^{13}C HMQC (500 MHz/125 MHz, CD_3CN) spectra of $[Fe(pq)_3](OTf)_2$ at 248 K, 257 K and 266 K. While additional HMQC spectra were recorded to cover the spectral range, additional cross-peaks were observed in the second spectral range at 257 K only (top).

4 Co_4L_6 Cage 8

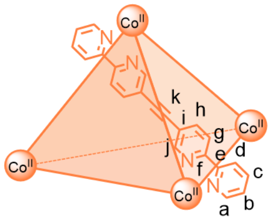

Cobalt bis(trifluoromethylsulfonyl)imide (10.9 mg, 17.6 µmol) and 1,2-di([2',2"-bipyridin]-5'-yl)ethyne (8.88 mg, 26.6 µmol) were dissolved in acetonitrile (1.5 mL) and heated at 50 °C for 22.5 h. After cooling to room temperature, the solution was added dropwise to diethyl ether (10 mL) and centrifuged. The organic layer was decanted and the residue washed with diethyl ether (10 mL) before drying the brown solid under air.

Yield: 13.3 mg (2.97 µmol, 68%)

^{1}H NMR (600 MHz, CD_3CN, 298 K) δ (ppm): 85.3 (br, 12H, $H_{a/j}$), 85.0 (s, 12H, H_d), 81.1 (s, 12H, H_g), 79.7 (br, 12H, $H_{a/j}$), 45.0 (s, 12H, H_b), 14.8 (s, 12H, H_c), 11.1 (s, 12H, H_h).

^{13}C NMR (151 MHz, CD_3CN, 298 K) δ (ppm): 680.4 (s, C_i), 611.2 (d, ^{1}J = 172 Hz, C_b), 423.3 (d, ^{1}J = 164 Hz, C_d), 408.8 (d, ^{1}J = 171 Hz, C_g), 226.9 (s, C_k), 179.5 (d, ^{1}J = 166 Hz, C_h), 176.7 (d, ^{1}J = 166 Hz, C_c), 67.0 (d, ^{1}J = 107 Hz, $C_{a/j}$), 31.2 (d, ^{1}J = 120 Hz, $C_{a/j}$), -139.2 (s, $C_{e/f}$), -146.5 (s, $C_{e/f}$).

^{19}F NMR (471 MHz, CD_3CN, 298 K) δ (ppm): -80.6 (NTf_2^-).

HRMS (ESI): *m/z* = 1960.9854 [**8** + $6NTf_2]^{2+}$, 1214.0164 [**8** + $5NTf_2]^{3+}$, 840.5322 [**8** + $4NTf_2]^{4+}$, 616.2422 [**8** + $3NTf_2]^{5+}$, 466.7155 [**8** + $2NTf_2]^{6+}$.

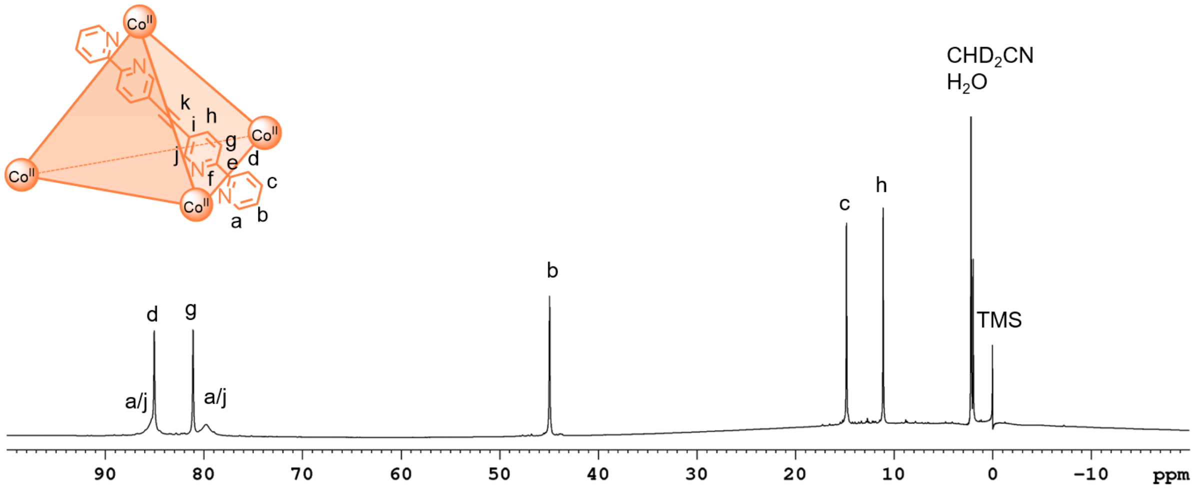

Figure S113. ^{1}H NMR spectrum (600 MHz, CD_3CN, 298 K) of cage **8**.

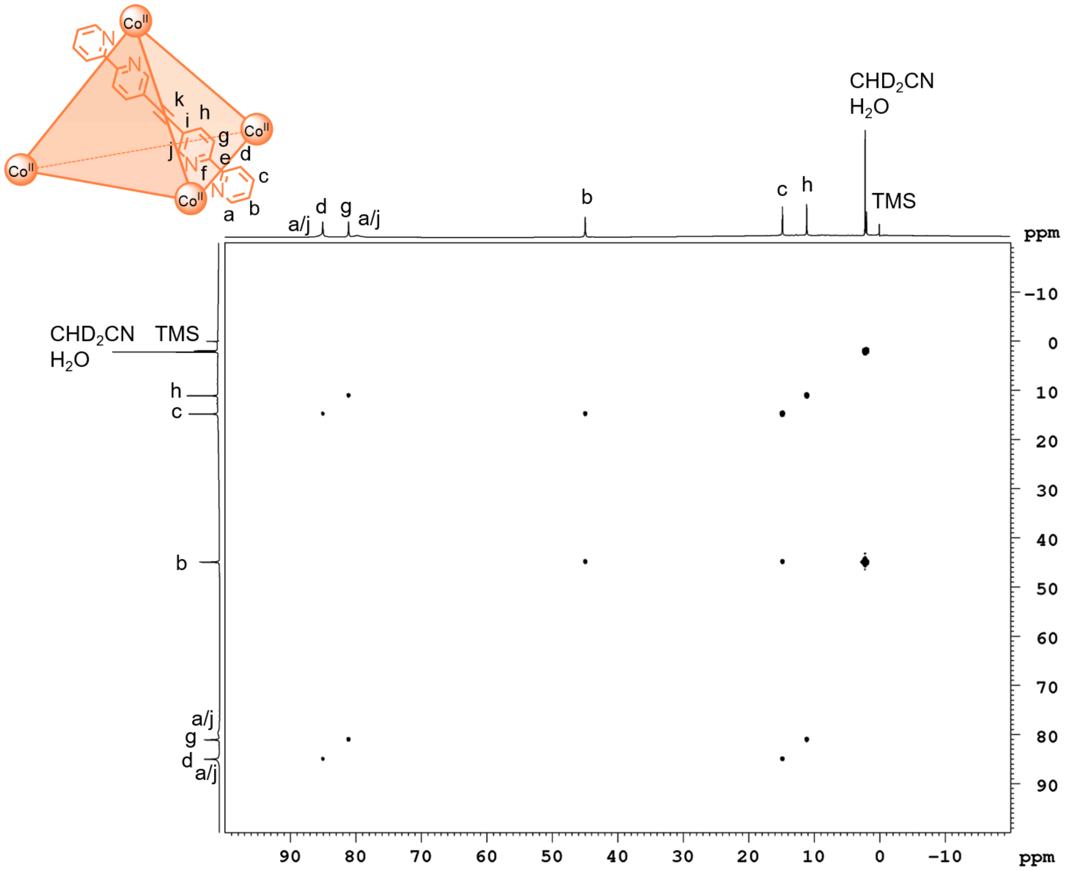

Figure S114. ^{1}H-^{1}H COSY NMR spectrum (600 MHz, CD_3CN, 298 K) of cage **8**.

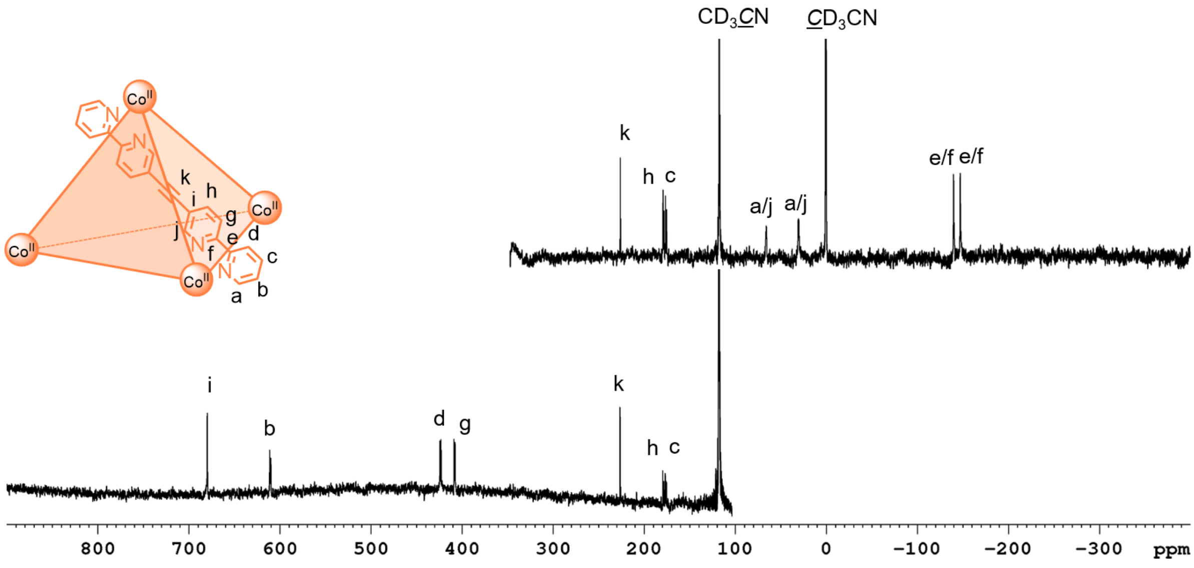

Figure S115. ^{13}C NMR spectrum (151 MHz, CD_3CN, 298 K) of cage **8**.

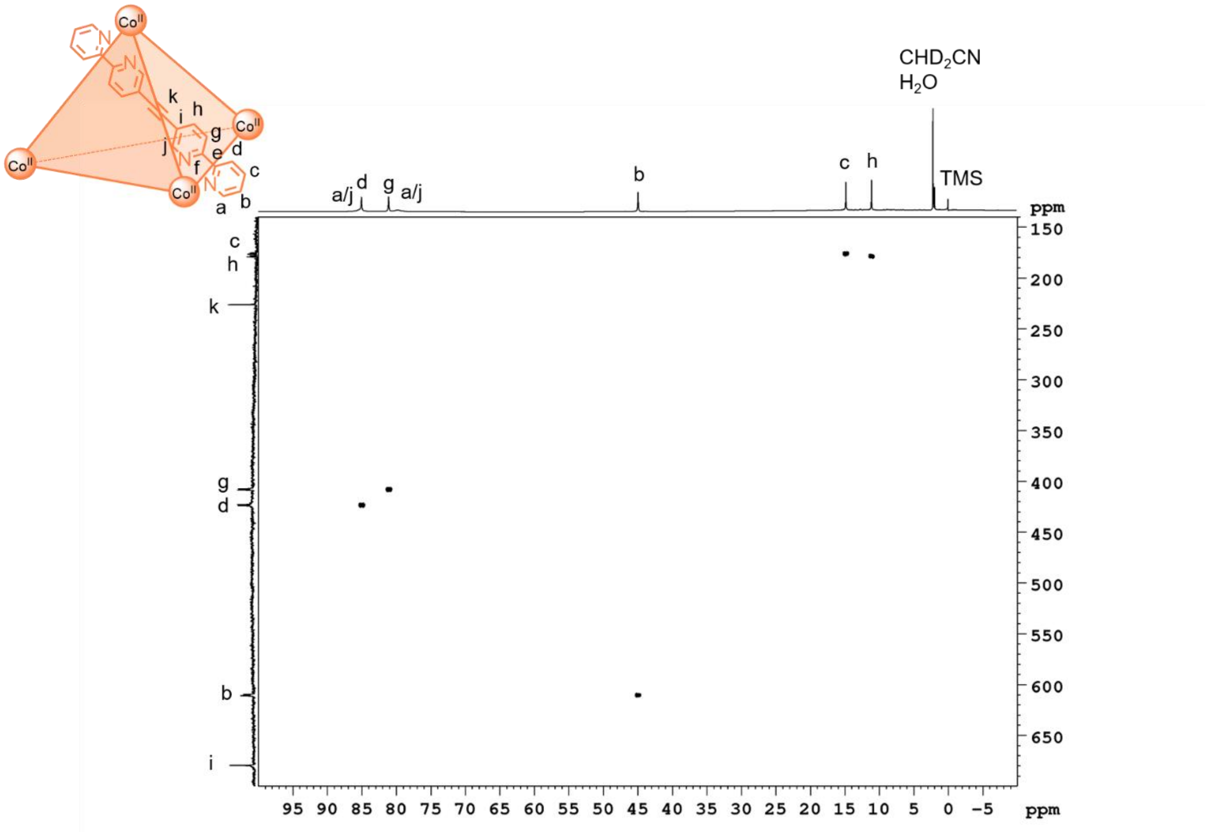

Figure S116. ^{1}H-^{13}C HMQC NMR spectrum (600 MHz/151 MHz, CD_3CN, 298 K) of cage **8**.

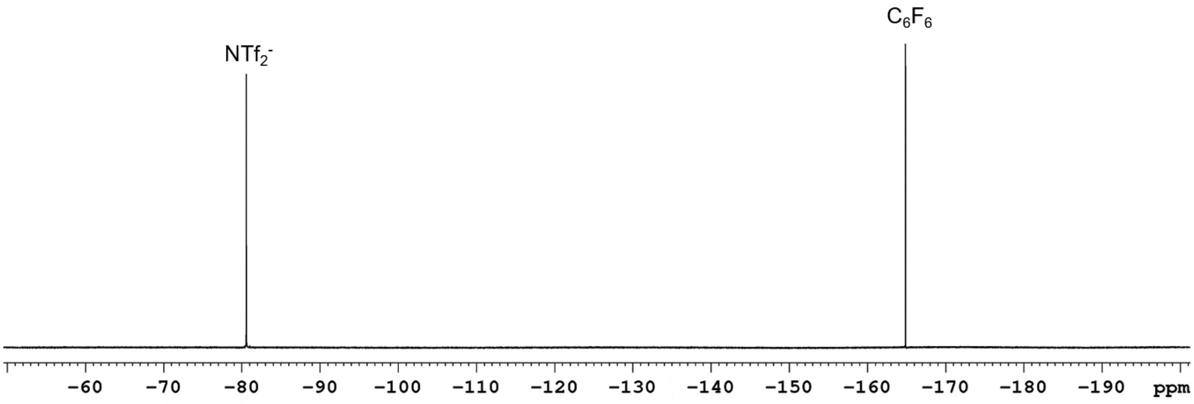

Figure S117. ^{19}F NMR spectrum (471 MHz, CD_3CN, 298 K) of cage **8**.

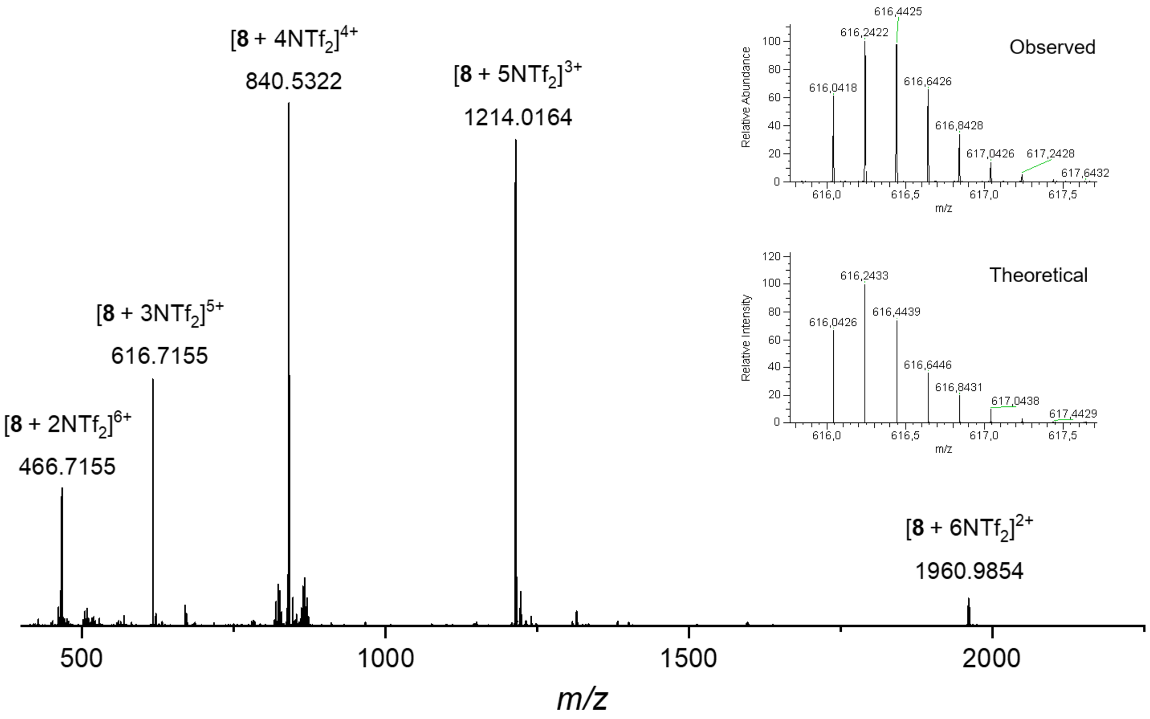

Figure S118. High resolution ESI mass spectrum of cage **8** showing in the inset the observed (top) and theoretical (bottom) isotope patterns.

5 References

1. Sheldrick, G., *Acta Crystallograph., Sect. A: Found. Adv.* **2015,** *71*, 3-8.
2. Sheldrick, G., *Acta Crystallograph., Sect. C: Struct. Chem.* **2015,** *71*, 3-8.
3. Stoe&Cie *X-AREA*, 1.44; STOE & CIE GmbH: Darmstadt (Germany), **2008**.
4. Cho, C. S.; Ren, W. X.; Shim, S. C., *Tetrahedron Lett.* **2006,** *47*, 6781-6785.
5. Wang, R.; Fan, H.; Zhao, W.; Li, F., *Org. Lett.* **2016,** *18*, 3558-3561.
6. Abarca, B.; Ballesteros, R.; Ballesteros-Garrido, R.; Colobert, F.; Leroux, F. R., *Tetrahedron* **2008,** *64*, 3794-3801.
7. Hu, Y.-Z.; Zhang, G.; Thummel, R. P., *Org. Lett.* **2003,** *5*, 2251-2253.
8. Bonakdarzadeh, P.; Pan, F.; Kalenius, E.; Jurček, O.; Rissanen, K., *Angew. Chem. Int. Ed.* **2015,** *54*, 14890-14893.
9. Ma, L.; Guo, S.; Sun, J.; Zhang, C.; Zhao, J.; Guo, H., *Dalton Trans.* **2013,** *42*, 6478-6488.
10. Kim, M.; Kang, C. H.; Hong, S.; Lee, W.-Y.; Kim, B. H., *Inorg. Chim. Acta* **2013,** *395*, 145-150.
11. Solomon, I., *Phys. Rev.* **1955,** *99*, 559-565.
12. (a) Amouri, H.; Mimassi, L.; Rager, M. N.; Mann, B. E.; Guyard-Duhayon, C.; Raehm, L., *Angew. Chem. Int. Ed.* **2005,** *44*, 4543-4546; (b) Tidmarsh, I. S.; Taylor, B. F.; Hardie, M. J.; Russo, L.; Clegg, W.; Ward, M. D., *New J. Chem.* **2009,** *33*, 366-375.
13. (a) Onggo, D.; Hook, J. M.; Rae, A. D.; Goodwin, H. A., *Inorg. Chim. Acta* **1990,** *173*, 19-30; (b) Jahro, I. S.; Onggo, D.; Ismunandar; Rahayu, S. I.; Muñoz, M. C.; Gaspar, A. B.; Seredyuk, M.; Gütlich, P.; Real, J. A., *Inorg. Chim. Acta* **2008,** *361*, 4047-4054; (c) Harris, C.; Kokot, S.; Patil, H.; Sinn, E.; Wong, H., *Aust. J. Chem.* **1972,** *25*, 1631-1643.
